# Supplementary material for: Barriers and limitations to the development of a telemental health service for workers in Peru- A user-centered approach
Source: PLoS One. 2025 Apr 9;20(4):e0321401. doi: 10.1371/journal.pone.0321401 (PMC11981184; doi:10.1371/journal.pone.0321401)
Supplement: S1 File — Questionnaires used for the semi-structured interviews with external users, internal users, and decision-makers. (DOCX) [file pone.0321401.s001.docx]

**Interview Guide for Decision Makers**

**Profile: ___________ Code: ___________**

**Date: ___________ Gender:___________**

**Age: ___________**

Warm-up

- What is your opinion on the use of digital interventions, specifically those related to telehealth, for addressing mental health issues?

Norms and Policies (Access and Coverage)

- What policies, regulations, or guidelines exist that guide the implementation or use of digital interventions in mental health? Are there any particularly important policies, regulations, or guidelines that have influenced your work?
- In your opinion, what factors influence the capacity for coverage and care that digital mental health services, such as teleconsultations or telecare, can provide?

Experiences with These Interventions

- What experiences have you had with the implementation or use of digital interventions in mental health, such as teleconsultations?
- What strategies have you applied or observed being implemented to encourage users of the health system to adopt digital mental health interventions, such as teleconsultations? How have these experiences succeeded in retaining users within the health system?
- What communication or dissemination strategies are used to promote the use of teleconsultation systems, telecare, or other digital mental health interventions?

Barriers, Facilitators, and Needs

- What problems have you encountered or what barriers have you identified in the implementation or use of teleconsultation services? What has been the greatest barrier/difficulty, and why do you think it is the most significant? Why do you think this occurs?
- What facilitators or catalysts for the implementation or use of digital platforms for teleconsultations, telecare, or digital interventions have you found within the health system?

**Interview Guide for Health Workers (Psychologists and Psychiatrists)**

**Profile: ___________ Code: ___________**

**Date: ___________ Gender:___________**

**Age: ___________**

Warm-up

- Briefly describe how long you have been providing mental health services through telehealth, and how has your experience been in delivering mental health services via telehealth?

Norms and Policies (Access and Coverage)

- Do you have any guidelines, protocols, or manuals that you use to conduct teleconsultations? If so, please describe your experience using them. If not, what elements should this guide or manual cover (e.g., steps to follow in teleconsultations, psychological instruments for assessment)?

Experiences with These Interventions

- Through which platforms, programs, or media have you provided telehealth services? How has your experience been with these platforms? (If you have used more than one, describe all and mention which you prefer and why.)
- What do you like about using these platforms to provide teleconsultation services? What do you like the most? What do you dislike about using these platforms? What do you dislike the most?
- How do you find the interaction with patients using telehealth platforms? How has the use of digital means for consultations affected patient adherence?
- What communication and dissemination strategies have you observed the health system using to promote the adoption of teleconsultations by health personnel or users?

Barriers, Facilitators, and Needs

- What difficulties have you encountered while providing telehealth services? What has been the most significant challenge? How would you address it?
- What facilitators or benefits have you found in using teleconsultation services?
- Please provide some suggestions: What additional features should the telehealth platform you use have to facilitate your work?

**Interview Guide for Users (Health Sector Workers, Police, and Education)**

**Profile: ___________ Code: ___________**

**Date: ___________ Gender:___________**

**Age: ___________**

Experiences with These Interventions

- How has your experience been throughout the teleconsultation process? This includes seeking care, scheduling an appointment, and receiving the service.
- Were you asked to meet any prerequisites before receiving teleconsultations or online care? If so, what were they, and how was your experience with the process of meeting these requirements?
- How many teleconsultations have you received? What did you like most about receiving care via teleconsultation? What did you like least?
- Through which medium did you receive teleconsultations? (e.g., phone, computer, tablet?) What program did you use for the teleconsultation, such as Zoom, Meet, or a specialized software? How did you find the program you used for the teleconsultation?

Barriers, Facilitators, and Needs

- What barriers or problems have you encountered in receiving teleconsultation services? (If needed, provide examples such as “issues with the teleconsultation platform or difficulties scheduling appointments.”)
- Are there benefits or conveniences to receiving teleconsultations compared to in-person care?
  What features or improvements should the teleconsultation program have to make you feel more comfortable during your teleconsultation?
