# Supplementary material for: Barriers and limitations to the development of a telemental health service for workers in Peru- A user-centered approach
Source: PLoS One. 2025 Apr 9;20(4):e0321401. doi: 10.1371/journal.pone.0321401 (PMC11981184; doi:10.1371/journal.pone.0321401)
Supplement: S2 File — Software requirements and procedures identified in the study. (DOCX) [file pone.0321401.s002.docx]

**Recommendations based on Telehealth/Teleconsultation User Codes**

1. **Experiences with these interventions:**

- The response to calls for scheduling appointments is problematic due to long wait times.
- Telephone consultations are sometimes very impersonal (lacking satisfaction/empathy for the user).
- Telephone consultations are occasionally not answered because the number is mistaken for an automated call (from banks, service offers, etc.).
- The numbers used for calls change with each attempt, which generates distrust among users.
- Every time users contact doctors by phone, they have to recount their issues due to changing doctors with each call. It doesn’t feel the same as having a personal medical record.
- There should be more follow-up, which is not the same as having more calls, but rather keeping up with the user's current situation.
- Being attentive to calls is difficult when one is in poor health.
- Users sometimes felt that the calls were made out of obligation (without a genuine interest in their well-being).
- There is a delay in sending the meet/zoom link, which causes user concern.
- Questions in the mental health area did not allow users to open up and build more trust.

1. **Description of barriers or problems in receiving care**

- The barrier is related to the type of care: the service provides a prescription, listens, and gives a list of medications, which is considered impersonal.
- Calls are made at times that are inconvenient for users, who are often busy. There are no fixed hours.
- Users have to take their own notes because there is no medical record maintained with the telehealth service, which is frustrating for them.
- Users report that often there are no available appointments in the telehealth service, even when doctors provide referrals. There are no specialist appointments available.
- The requirement to receive calls when users are in poor health.
- Scheduled appointments do not always adhere to the agreed date.

1. **Suggestions for the future**

- Users prefer medical consultations to be virtual to avoid wasting time.
- If a virtual appointment application is developed, it should consider that not all adults are well-versed in technology.
- It is preferred that the first appointment be in person, followed by teleconsultation for follow-ups.
- For psychological care, video calls should be prioritized.
- Some users prefer telecare to be conducted via video call on Zoom rather than a phone call.
- Specialists should be trained to build rapport with users.
- Develop an emotional support program for teachers.
- It is necessary for the EPS and telecare services to use the same system.
- Appointments should be punctual and better coordinated.
- Increase the range of specialties available in telecare.
- Train public and private sector workers on using Zoom.
- Implement an electronic medical record so that if another doctor is attending the user, there are no issues or inconveniences with starting from scratch.
- Provide a known contact number for calls that is not the personal number of the doctors.
