# Supplementary material for: Barriers and limitations to the development of a telemental health service for workers in Peru- A user-centered approach
Source: PLoS One. 2025 Apr 9;20(4):e0321401. doi: 10.1371/journal.pone.0321401 (PMC11981184; doi:10.1371/journal.pone.0321401)
Supplement: S3 File — Transcripts of the interviews by topic, in Spanish. (DOCX) [file pone.0321401.s003.docx]

**D1: Tomador_Decision**

**E:**Buenas tardes, en día de hoy estamos con —----, él pertenece a la dirección del Ministerio de Reconocimiento al Docente del Ministerio de Educación. Buenas tardes, —------.

**e:**Hola, Liliana, ¿cómo estás? Es la dirección de promoción del Ministerio de Reconocimiento.

**E:**Correcto, gracias, —---. Dirección de promoción, reconocimiento al docente. Alfredo, ¿podrías presentarte, por favor, tus nombres completos?

**e:**soy —----, coordinador de Bienestar al Docente.

**E:**Gracias, —---. En esta oportunidad, —----ha decidido participar voluntariamente como parte de uno de los gestores que ha hecho anteriormente uso de estas plataformas digitales y en el marco del estudio que es documento de diseño y evaluación de la usabilidad, aceptabilidad, y satisfacción de un servicio de telesalud a tamizaje, manejo inicial y derivación oportuna en salud mental para trabajadores de grupos ocupacionales vulnerables con antecedentes de COVID 19 en Lima. Bueno, —------, gracias. Ahora voy a proceder a hacerte unas cuantas preguntas. Primero quisiera conocer un poco sobre tu opinión en cuanto al uso de intervenciones digitales. Sabemos que anteriormente has podido trabajar con un programa de escucho docente que también fue una intervención digital enmarcado un poco en la atención. ¿Podría darnos un poco tu opinión sobre las intervenciones?

**e:**Mira, la mayoría de estas intervenciones a distancia o digitales, como señalas, que se desarrollaron en temas orientados a promover el bienestar socio emocional de las personas, salieron con mucha fuerza en este contexto de pandemia, ¿no? Porque lo regular, que en principio es lo que viene siendo ahorita reclamo de parte de muchos usuarios, es el trabajo de manera más presencial. Sin embargo, las intervenciones remotas a distancia también han permitido acercarse a lugares a los cuales muchas veces era difícil poder acercarse, ¿no? Además, también te permiten una inmediatez de reducción en tiempos de desplazamiento, etcétera, determinados momentos específicos para poder asistir, por ejemplo, a una cita, sea con un psicólogo o recibir un taller por parte de un otro especialista. Entonces, creo que han habido muchas cosas interesantes respecto a las posibilidades que brindan este tipo de espacios, tanto favoreciendo el acceso, no solo por llegar a otros lugares, como te decía, sino también por la inmediatez

y la posibilidad de que alguien esté en su propio hogar, incluso en su lugar de trabajo. Pero también tiene algunas limitaciones, sobre todo cuando quieres ahondar o profundizar con algunos temas en particular.

**E:**Correcto, sí, sabemos que las intervenciones nos han permitido acercarnos quizás a más gente, a más población que está bastante distante del lugar donde trabajamos, y ese quizás ha sido el plus. Ahora, en esa línea, Alfredo, tú que has podido trabajar de cerca, o has podido tener esta experiencia de cerca, ¿estabas al tanto o conocías quizás un poco sobre las políticas, las normas o guías que existen o quizás guían un poco este proceso de implementación o el uso de esas intervenciones digitales, o tú, o como parte del equipo quizás, que estuvieron gestionando toda esta idea, este movimiento, ¿pudieron conocer de cerca o empaparse del tema en este proceso?

**e:**Sí, de hecho nos tocó ver desde varias cosas, desde el tema referido a todo lo que tiene que ver con el manejo de protocolos de trabajo en atención a distancia, que en este caso lo recibimos desde la Federación Internacional Cruz Roja, que ya tenían una serie de herramientas validadas en los diversos congresos de emergencia en los que ellos ya han trabajado, pero igual tuvimos que hacer una adecuación, porque, como sabes, siempre toca adecuar algunos elementos, algunos instrumentos, a las dinámicas propias de la población con la que nos toca atender. Entonces, ahí desde el elemento respectivo al procedimiento y a la propia estrategia, como lo queríamos hacer, están también las propias normativas que el MINSA estuvo sacando y actualizando en el contexto de pandemia. Un año antes creo, había estado un documento referido a temas de salud mental. En el 2020 tuvieron que hacer actualización referida al Plan Nacional de Salud Mental en el contexto COVID, por ejemplo. Entonces nosotros revisamos eso también para enseñarnos y alinearnos frente a normas específicas sobre la estrategia que implementábamos. Las definiciones operacionales provenían de los documentos del MINSA, ¿no? Pero además tienes otro tipo de normativas, por ejemplo, referidas a el uso de bases de datos. Porque siempre en este tipo de procesos terminas gestionando bases de datos con información confidencial de las y los diversos usuarios que requieren este tipo de servicios. Entonces ahí, en este caso particular, por ejemplo, no solo es conocer la información, sino es también suscribir un... realizar una serie de gestiones para inscribirlos me parece que es el Ministerio de Justicia el que gestiona todo esto. Entonces hay que participar, hay que determinar qué es el punto focal, cómo se gestiona la base de datos, etc. Y cómo se protege también, cuáles son todos los elementos de protección de la base de datos de los usuarios que tienen la estrategia. Entonces, hay toda una serie de elementos mucho más allá que solo el pensar en lo que tienes que hacer para desarrollar tu servicio como dices ahí. Hay que tener una lectura de cuáles son los parámetros que están establecidos para llevarlo con determinados criterios de calidad.

**E:**Sobre todo porque manejamos información sensible, ¿no? Información muy personal que tiene que ser tratada de manera cautelosa y de manera reservada también.  Alfredo, durante esta experiencia que tú tuviste, ¿quizás qué factores podrías identificar o has observado que pueden tener algún impacto en la capacidad de cobertura? Porque entiendo que este programa te escucho, docente, fue dirigido a nivel nacional, ¿es correcto? Y si en ese sentido, en todo caso, ¿qué factores tú pudiste observar que propiciaran como miren, una mayor cobertura, ¿no? Y en todo caso, ¿qué otros no?

**e:**El primer tema clave para la cobertura es la difusión, ¿no? O sea, la cobertura va asociada a tu capacidad de atención versus tu difusión, porque tú puedes tener una capacidad de atención muy alta, por decirlo así, un equipo de 20, 30, 40, 50 profesionales, pero si no tienes una estrategia de difusión muy efectiva, vas a tener a todo ese grupo sin atenciones, ¿no? Por lo pronto, y la atención es siempre bajo. Entonces, un tema clave es que uno identifique que tiene los elementos para poder desarrollar estrategias de difusión dirigidas específicamente al grupo que quieres llegar, así como también que tienes la capacidad para dar respuestas. Ahora, en nuestro caso, por ejemplo, al inicio había mucha presión de decirla que no tenía capacidad para pedir ese contrato de 20, 30 psicólogos. Bueno, no dijeron eso. No había plata, pero querían que se contratara 20, 30 psicólogos. Pero sabiendo que este primero que nosotros hacemos en el sector, somos el sector de educación, entonces no es que hay una especial expertis en el desarrollo y en la provisión de este tipo de servicios. Lo que hicimos fue más en mirar mucho la experiencia de otros que en el mismo momento estaban implementando su propia estrategia como propiedad de cooperación, por ejemplo, acnologías, etc. Estaban desarrollando sus propias estrategias. Entonces era mirar qué es lo más conveniente en este caso. Lo más conveniente en este caso fue jugar con un número mágico, que era el primer grupo mínimo de seis personas, seis especialistas y un supervisor. Y con ese grupo nos quedamos 100, porque también era como reconocer quién era nuestro interés de desarrollar un servicio permanente, sino más bien iniciar una línea que luego transite con lo que sirve nuestra competencia. Promover y favorecer a los sectores que desde las regiones identifiquen eso como un problema y tomen medidas para ello. Entonces ahí era donde cambiaban un poco los roles. Pero para asegurar, volviendo un poco a la parte inicial de esa pregunta, para asegurar el llegar a la mayor cantidad de gente, es tener claro cuáles son tus mecanismos de difusión. En nuestro caso el mecanismo por excelencia fue en los paisajes de texto, en tanto que en el sector de educación, si logramos contar con base de datos de teléfonos celulares de los docentes y directivos, entonces se les podía enviar esa información. Y había mucha diferencia, por ejemplo, cuando tú habías mandado al fin de semana mensaje de textos a cuando habías posteado en redes o habías mandado correos u otro tipo de cosas. Nada era tan efectivo como el envío de mensaje de texto.

**E:**¿Podríamos decir quizá que los mensajes de texto son el medio de difusión digamos preferido o quizás más usado por la población docente?

**e:**Sí, creo que es lo más efectivo definitivamente. Claro, y tu mensaje de texto con un link dentro del mismo, un mensaje sencillo con pocos caracteres, pero que venga con tu link para que puedas acceder de manera directa a tu plataforma o a donde vas a gestionar la atención. Hasta llamadas hicimos.

**E:**¿Qué tal fue la experiencia con las llamadas? O sea, ¿con cuál tuvieron más recepción o mayor acogida? ¿Siempre fue mensajes?

**e:**No, mensajes. Porque además las llamadas tienen un inconveniente. Además de invertir mucho tiempo, terminan distrayéndote con otros temas. Entonces es como que las personas que reciben una llamada empiezan a tratar, a explorar otro tipo de cosas y se les pierde el sentido. Entonces, en el espacio que haces una llamada ya pueden llegar miles de mensajes de texto. Entonces, sí, los mensajes son los más repetidos.

**E:**Entonces, los mensajes tienen, digamos, mayor acogida quizás por parte de los docentes. Ahora, Alfredo, una consulta. Ustedes, al momento de que ya ofrecían el servicio, que ya tenían activo, digamos, su plataforma de Te Escucho Docente, ¿ustedes contaban con medios de agendamiento? ¿O ustedes a través del mensaje brindaban los horarios? ¿El participante los escogía? ¿Cómo era ese mecanismo de uso ya propiamente?

**e:**Se gestionó en una tabla tipo Wordpress, o sea, en una página web, una plantilla de tipo Wordpress, se incorporó un Google Form para que puedan ellos ingresar y solicitar su horario. Si bien la plataforma luego se complejizó, se hizo más sofística esa parte que quisimos en algún punto desarrollar un sistema de citas tipo clínica en la cual tú ves quiénes están disponibles, por decirlo. Entonces, tú vas viendo en tiempo real si se va copando o no se va copando la capacidad de acción de cierta persona, los horarios, etc. Esa parte de la programación no fue posible hacer porque quien nos apoyó con eso ya no contaba con recursos, así que siempre lo manejamos como un Wordpress. Y finalmente como un Google Form, no, un Office Form, uno de esos formularios de Office. Entonces, sí, era, o sea, lo que hacía el mensaje textual era el vínculo, y el vínculo finalmente era donde tú ya empezabas a acceder a llenar un formulario de Office. Ese formulario luego lo revisaba el supervisor que tenía que estar disponible, quien distribuía la carga. Cuántos están llegando por tal fecha, veía la carga que tenía el equipo asignado y iba distribuyendo. También para poder hacer la distribución, al igual que lo que escuché en la reunión anterior con ustedes, me parece, también había un sistema de semáforización para poder determinar cuál era el nivel de necesidad que podía tener.

**E:**Claro, quizás como una clasificación digamos, en quiénes tienen mayor riesgo, por decirlo así, o en quienes son más propensos a...

**e:**Así es, un triaje que te permitía decir ya esta persona necesita asistir ya. Entonces sería una persona con esa indicación. Pero eran muy pocos. Mira, entre el 2000, el 20, el 22, los rojos fueron ni siquiera el 1% de las personas. Los amarillos fueron más del 11% y los verdes fueron el 87%. Entonces ahí, como para que veas que realmente no eran tantas las situaciones así, muy complejas que llegaban.

**E:**Ok. Ahora, Alfredo, un poco más siguiendo ya quizás a las barreras, facilitadores y otras necesidades que pudiese identificar. De manera general, ya sea tanto en el diseño, en el uso o quizás en costos también, ¿qué barreras podrías haber identificado durante este proceso? Sobre todo en implementación de uso de esos servicios de teleconsulta. ¿Cuál dirías tú que fue la mayor dificultad, barrera? ¿Y por qué esa sería importante?

**e:**La primera creo que fue más institucional. O sea, a nivel del propio ministerio. Y tenía que ver con la relevancia que ya se le entregaba a este tipo de servicios. En cuanto a esta pandemia, de hecho, tampoco fue fácil, pero fue un poco más sencillo que se identifique el por qué era pertinente este tipo de servicios. Pero ya habiendo pasado algo en tiempo, ya no se le da tanta importancia. Cuando por el contrario, desde los usuarios

se le da mucha más importancia. Entonces, institucionalmente es como que dicen, bueno, ya pasó esto, yo ya no veo por qué tengo que importar tanto, pero no sé. Ahora, los primeros meses del año, si te acuerdas, tuvimos este tema del ciclón llano. Eso trajo toda una serie de situaciones, sobre todo hacia el norte del país. Era lo que principalmente estaban requiriendo de apoyo para los docentes y en general la comunidad educativa de esas zonas. Soporte socioemocional. Pero no tenías quien lo brinde, no tenías equipos, no tenías estrategias para poder desarrollar. Y ahí es donde voy también, el hecho de que lo que fue una barrera al inicio, que también no siempre había mucha costumbre desde las personas, desde los docentes de recibir este tipo de servicios, ahora ya no lo es. Ahora más bien hay mucha demanda. Si vieras el momento en el que conversábamos sobre nuestras estadísticas de demandas, o sea, que tanto subía la demanda de atención el año pasado. ¿Tengo una idea? El año pasado teníamos a 6.800 docentes. ¿Ya? Que fue el histórico más alto. Pero eso se atendió con un equipo de 6. Y es súper interactual. Aunque no fue un año entero, porque dieron como 3, 4 meses que no tuvimos financiamiento. Pero ya, mira, 6.800 docentes. Este año, con dos psicólogos, solo atendiendo de mayo a agosto, estamos pasando 2.500 docentes. Porque todas han sido demandas de atención grupales. ¿Qué ha cambiado en este tiempo? Que hay mucha mejor disposición de participar.

**E:**Quizá ocurre algo paradójico, porque institucionalmente quizás al inicio hay mucho soporte, pero luego con el tiempo, bueno, también se acaban los recursos, va bajando el interés, pero en cuanto a la población, el interés de la población, los docentes, más bien va creciendo con el tiempo, ¿no? Ante mayor discusión, tiene mayor acogida también. Entonces hay un efecto inverso, quizás una relación inversa entre esas dos poblaciones.

**e:**Y son mucho más conscientes de cómo se comunica. Y tenías que empezar como 10 minutos, 15 minutos para tratar de ir movilizando el tema, viendo quién habla. No, ahora los espacios son mucho más fluidos. Las personas que van y tratan de aprovechar al máximo, están en esos espacios. Entonces, como dices, hay una mayor disposición desde el usuario en saber, en exigir y en utilizar los servicios que se le ofrecen. Probablemente ya no hay tanta disposición, pero, ¿cómo lo aterrizaría también respecto a este tema institucional? No solo porque haya o no haya un recurso, sino ¿cuál es la apuesta? Y ahorita que vuelvo a pensar en el tema de los organismos de difusión, claro, nosotros nunca hicimos, por ejemplo, una campaña en medios. Salvo al inicio, que si salimos, me parece, en una radio, alguna nota de prensa, en un periódico, una cosa así. Salvo al inicio, que tuvimos por ahí unas dos, tres cosas en medios, después no. Se habla de julio de 2009. Después nunca más hubo nada de eso. Entonces, es muy distinto a lo que por ejemplo, de la media 113 o del MIGS. Entonces, claro, tú tienes la posibilidad en medios bastante potente, pero nosotros jamás hemos tenido la la chance de medir eso como impacto de difusión.

**E:**Correcto. Eso también está relacionado a la importancia de los medios, ¿no? De tener el apoyo quizás de notas de prensa, o difusiones a través de medios masivos, radio, televisión, ¿no? Para tener un poquito más de acogida. Ahora, por el otro lado, en contraste a todo lo que has mencionado, algunas facilidades, o quizás catalizadores que hayas podido identificar para la implementación o el uso de esas plataformas digitales, de teleconsulta, teleatención, interacciones digitales, dentro del propio sistema que tú has usado, que ustedes han usado como Minedu, ¿cuáles son algunos de esos facilitadores?

**e:**El mismo hecho de que sean virtuales, hace que se puedan correr la voz también de manera más fácil entre los mismos usuarios. Creo que eso es un tema tan importante, ¿no? Porque ellos también hacen un boca a boca, ¿no? De poder pasarse la voz, de decir, oye, si esto existe, esto funciona. Otra cosa que creo que también es importante es que hay muchas iniciativas locales también. Ahorita sí no podría decir de cuántas de estas persisten, pero hasta el año pasado había muchas iniciativas en territorio. Entonces, este... Y a nosotros, una de las tareas que nosotros tuvimos fue también de capacitar mucho a los equipos en región. Entonces, creo que sí hubo un momento en el cual hubo un interés genuino desde las autoridades, al menos en territorio, en fortalecer su propia capacidad de brindar respuesta a este tipo de iniciativas. Entonces, era mucho más sencillo que se entienda la demanda ya no solo desde el usuario, sino también desde el que tenía que proveer las condiciones. Eso favorecía mucho el desarrollo de este tipo de trabajos de educación. La presencia de múltiples tipos de plataformas virtuales también. Claro, en nuestro caso, las retenciones individuales eran llamadas, no había mucho que hacer. El hecho de que tú lo contactes, solo siente tener un tema muy potente. No es sentir que es el docente el que está atrás de ti para que lo puedan atender, sino que es como que es práctica regular. O sea, en su trabajo suele ser uno el que está atrás para que le ayuden a resolver algo. Era al revés. Éramos nosotros quienes estábamos atrás de ellos para ayudarles. Entonces, creo que eso es un tema también bien potente para generar un poco la imagen respecto al servicio que se les estaba brindando a las personas. El que hayamos sido también capaces de adaptarnos a diversas plataformas, sobre todo cuando brindábamos los servicios grupales, era también algo importante porque hay regiones donde no funciona el Teams, no están acostumbrados al Teams, hay lugares donde no están acostumbrados al Zoom. Así que nosotros díamos, multiplataformas y gratuitas donde era posible para el caso de las urbanas. Eso es lo que requerían. Otro tema que también creo que ayudó mucho, sobre todo el 21 y el 22, fue que capitalizando lo que salían de los servicios de contención, identificábamos cuáles eran las principales problemáticas que estaban movilizando los centros en ese contexto. Y en base a ello empezamos a generar webinars mensuales. No íbamos a tener una capacidad de llegar a mucha gente, lo que sí hacíamos era, no podemos atener a los miles que estaban. Lo que sí podíamos era, a través de estos webinars, de manera específica, esos temas en los que los estaban movilizando, los que les estaban interesando. Y así también siempre se hacía una difusión del servicio, sacábamos también intereses desde los comentarios que dejaban en los webinars. Así como producir recursos en función a esas temáticas. Entonces hemos hecho herramientas específicas.

**E:**A menos de todo lo que has mencionado, me queda bastante claro que lo que ha facilitado quizás más todo ese proceso, ha sido un poco también la capacidad de adaptación, de poder usar diversas plataformas. Y el tema también de identificar sus necesidades a través de estos webinars, que creo que son espacios bastante útiles porque te permiten interactuar directamente con la población. Y conoces de primera mano sus necesidades. Es un buen espacio para recolectar información.

**e:**Sí, el webinar es un espacio muy interesante para ellos, para tomar temperatura directa de lo que está pasando. También muchas veces te han macheteado horrible.

**E:**Sí, suele suceder. Sobre todo cuando están en vivo.

**e:**Así es, pero es parte de eso. Entonces, digamos, siempre hay que, como dices, seguir tomando de ese lado lo que tú coges de ahí para poder devolverlo.

**E:**Claro. Sobre todo la reflexión. Y justamente ya llegando a la parte final, les entrevisto a Alfredo, nos sería quizás que nos puedas dar una reflexión, una recomendación, digamos, de lo que ha sido tu experiencia y que quizás nos pueda servir a nosotros.

**e:**Yo creería que con el servicio que ustedes están pensando implementar, un tema clave es sobre todo porque están abordando tres grupos profesionales muy importantes dentro del país. Entonces creo que es importante un acercamiento más institucional a los propios sectores señalando el trabajo que están haciendo. O sea, que no quede solamente un acercamiento de nosotros, que ya estamos teniendo para aterrizar, por ejemplo, sino que es solo un acercamiento entre la inflación y la paz libre, que estamos teniendo un vínculo por estos temas de seguridad, salud y trabajo, sino que es bueno que se destaque más bien todo el contexto. En este caso, como el Instituto Nacional de Salud, el Censopas se está desarrollando este estudio, está financiado, pero que es una avaliación de una propuesta de servicio, tal tipo que tiene el Censopas. Entonces, creo que es importante evidenciar lo que se hace, que quede claro para todos los sectores que se está haciendo. Y así también tratar de ir generando y jalando mucha más evidencia sobre los impactos positivos de este tipo de intervenciones. En algún punto, me parecería importante que además de estas vinculaciones sectoriales, también puedan haber vinculaciones territoriales. Entonces, de pronto tener vinculaciones con algunos gobiernos regionales que ustedes consideren lo bueno es que educación, salud, están descentralizados. Entonces, tienes una dirección y una dirección regional de educación en todos lados. No me queda muy claro cómo es en el caso de las fuerzas policiales. Creo que eso no está descentralizado. Descentralizado como si los otros, ¿no? Entiendo que debe estar desconcentrado, pero no sé si descentralizado. Y eso puede servir también para señalar cuál es el trabajo que se está desarrollando. Yo creo que un gran aprendizaje de eso es hacer explícito como diríamos en catalán, la calidad. O sea, ser muy obvio sobre lo que se está haciendo. Que se sepa lo que se está haciendo y que se sepa los impactos. Yo me acuerdo que la primera vez que conversamos, ustedes me explicaron que ibas a poder salir con un informe. Entonces, eso es algo que realmente es como que un quiebre para muchas personas. Y que saben que eso es algo en lo que no van a poder llegar muchos casos, ¿no? Sobre todo, por ejemplo, quienes acceden al Polisalud. Porque si quiere llegar una atención especializada en salud demora mucho tiempo. Entonces, ese me parece que es un tema que podría ayudar y que además va a poder igual beneficiar también a los mismos grupos poblacionales que ustedes están señalando. Porque va a poder volver a meter el énfasis en el porqué se apuesta por esos grupos poblacionales. Profesionales. ¿Cuál es la importancia, lo clave de este grupo y porqué se tiene que seguir apostando por eso? Porque ese me parece que sería un tema importante. El otro es si existe el el algunos de esas cosas también en coordinación con el Instituto de Opinión Pública, con Pulso PUCP. Este se desplome en la serie de artículos todavía se está trabajando algunos. Jonathan lo conoce bien en el topo de ese tema. Entonces, se abrió eso como un campo para tratar de promover la investigación y ahondar en la problemática del impacto en los temas de bienestar socioemocional en los docentes. En particular, en este contexto con la data de lo que tenía en nuestras atenciones, la data de concentración de pacientes, otro tipo de información. Profundizar en temas de género, profundizar en diversas cosas que están ocurriendo. Y ese creo que es un tema que es muy importante. Claro, en el caso de ustedes ya nace una organización de contribución académica además, ¿no? Pero me parece que se puede ampliar muchos otros elementos. Que también implica el identificar con quienes en esta primera parte de articulación, también implica eso, identificar cuáles pueden ser estos otros actores para articular. Porque ustedes no tienen que resolver todo, pero sí pueden sugerir, ¿no? Y señalar, mira, acá hay un, este tipo de situación. O oportunidad, no tanto problemática, pero puede haber una oportunidad para que este otro lo aproveche. Y así sigan ahondando, profundizando en otro tipo de cambios. Y eso sería de momento algunas primeras reflexiones.

**E:**Bien, muchas gracias —-----. Ha sido bastante enriquecedora toda su experiencia que has tenido con la administración de la plataforma Te Escucho Docente. Un poco las barreras, los facilitadores, ¿no? Los contras que siempre hay de cualquier actividad. Yo creo que esta propuesta que haces en CENSOPAS nos va a servir bastante para conocer, para impulsar. Es un servicio y también poder conseguir aliados estratégicos de otras instituciones. Y por qué, tal vez, no, quizás algo más interterritorial, como mencionabas. Muchas gracias Alfredo. Gracias por toda tu experiencia. Y bueno, por hoy terminamos la entrevista.

**D2: Tomador_Decision**

**E:**Muy buenas tardes doctora, muchísimas gracias por haber aceptado su participación en el estudio de investigación. Díganos por favor su nombre y su cargo para comenzar con la entrevista.

**e:**Mi nombre es —------------, yo soy directora ejecutiva de la dirección de hiposalud, de la dirección de telesalud, referencia y urgencias del Ministerio de Salud y tengo a cargo la línea 113.

**E:**Muchas gracias. Bueno, vamos a comenzar con una serie de preguntas. La primera se va a relacionar con ¿cuál es su opinión respecto al uso de intervenciones digitales específicamente relacionadas con telesalud para la atención de problemas de salud mental? En general, su opinión.

**e:**En realidad ha facilitado mucho el uso de todas las tecnologías de la información y comunicación para llegar a la población. Hay mucha población dispersa, hay mucha población lejos que durante todo el tiempo no tiene acceso a los servicios de salud y tienen mucho temor al acceso justo en salud mental. Entonces, sí nos ha facilitado el tener canales de información, ya sea telefónicos o virtuales, para poder llegar a esa población que de cierta forma está un poco olvidada. Entonces, le estamos dando oportunidad de atenderse que antes no la tenían.

**E:**Respecto a las normas y políticas, específicamente sobre acceso y cobertura, ¿qué políticas, normas o guías existen que guían la implementación o el uso de intervenciones digitales en salud mental actualmente? ¿Alguna política o norma o guía en particular que considere importante que haya incluido en su trabajo?

**e:**Tenemos en realidad dos. El reglamento propiamente de telesalud, donde te facilita todas las reglas del juego para utilizar la telesalud en todos sus ámbitos. Y tenemos también el reglamento de salud mental, en el cual te pide que cuando promociones por lo menos lo que es intento de suicidio, promociones también el que pueda acceder telefónicamente a un servicio gratuito, como es el 113 en la opción 5. Entonces, esas dos nos están ayudando en intervenir en este problema de salud pública que es la salud mental.

**E:**Y en su opinión, ¿cuáles son los factores que influyen en la capacidad de cobertura y atención que pueden tener los servicios de salud mental digitales como teleconsultas o de alta atención en el marco de normas y políticas?

**e:**Limitaciones. En realidad todavía las tecnologías no son abiertas para todo el público. Tenemos que hay grupos etarios que manejan muy bien las tecnologías, pero tenemos adultos mayores o niños que todavía están en esa evolución de poder utilizar las tecnologías para acceder en este caso a salud. Eso es una limitante. Luego tenemos también la costumbre de las personas. Muchas veces estamos acostumbrados a querer ir a ver al médico o a querer ir a ver al psicólogo cuando esta puerta nos permite que desde nuestra casa poder ser atendidos. Y eso es muy importante en realidad, pero también nos limita porque hasta que nos acostumbremos, la idiosincrasia del peruano y de la gente en general se acostumbre a que si es una puerta factible, una puerta verdadera para tener contacto en salud, eso va a ser limitante. Necesita que las personas al final nos acostumbremos a las tecnologías, que todavía estamos en ese proceso.

**E:**Claro, lo que tú comentas justamente es la brecha de repente de alfabetización digital que pueden tener las personas para poder hacer estos servicios, pero en general o específicamente en relación de repente a normas y políticas, ¿usted ha encontrado alguna limitante que cree que podría mejorar en este aspecto también, que facilite la cobertura?

**e:**Nos facilitaría, por ejemplo, el cambiar un poco lo que es la norma de referencias. ¿Por qué le digo? Porque nosotros atendemos usuarios todos los días para salud mental y cuando necesitamos que se intervenga a otro nivel, llámese ya en una oferta fija, está la limitante de la norma de referencias que le pide que vaya a un establecimiento de salud físicamente antes de ir a un establecimiento especializado. Entonces eso sí es una limitante normativa, delineamiento, que nos está perjudicando en realidad la atención. Entonces hay algunas normas que están hechas para la oferta fija, ¿no? Y la oferta ahorita por telesalud es una oferta nueva que todavía le faltan muchas cosas por normar.

**E:**Probablemente, como mencionas, necesita madurar todavía este proceso, ¿no? De repente hay algunas características nuevas que usted cree que como proceso en la experiencia que ya ha podido tener, cree que podrían incorporarse en estas nuevas normativas. ¿Cuáles serían? No sobre referencias, pero de repente comentanos un poquito más respecto de ello.

**e:**¿A qué se refiere referencia? O sea, cuando un usuario de salud acude a atenderse por primera vez, va a un cierto nivel de atención. ¿Sí? Y para poder recibir una atención especializada requiere un formulario y una derivación que le dice la gente, pero que se llama referencias en realidad. Entonces, ¿qué es lo que pasa? Nosotros lo estamos atendiendo como si estuviera en un primer nivel. Nosotros, como en 113 tenemos una oferta ampliada. Les estamos atendiendo gratis a la población, pero no le damos un formulario para que se atiendan en el siguiente nivel de atención. Entonces, ese es un candado, ¿no? Y nos juega en contra porque ya lo diagnosticamos, ya le dimos su intervención inicial, le hemos hecho un tamizaje y ya requiere una intervención mayor, pero cuando llega a ese establecimiento de salud le piden que retorne al inicio donde empezó. Entonces, eso se tiene que cambiar. Es una norma que hay que cambiarla para que en verdad la oferta que estamos dando tenga el fruto que necesitamos.

**E:**Y ahora cométenos sobre, por ejemplo, las experiencias sobre el uso de estas intervenciones. ¿Qué experiencias ha tenido con implementación o el uso de intervenciones digitales en salud mental como las teleconsultas aquí en la institución?

**e:**Nosotros, desde que empezó la pandemia, además, desde que antes que empezara la pandemia en Perú, hacíamos videollamadas a la China, ¿ya? Porque el consul de allá estaba muy preocupado por los estudiantes peruanos que estaban allá y no estaban aislados, estaban atrapados, entre comillas, y no podían tener contacto con su familia. Entonces, nosotros hacíamos por Skype o por videollamadas. No utilizan WhatsApp, ya porque está prohibido, pero había otro tipo de videollamadas y ellos escapaban por el Skype para poder consultar con nuestros especialistas en salud mental para tranquilidad por el aislamiento que tenían. Entonces, eso, por ejemplo, es algo que no se cuenta porque fue antes para nosotros de la pandemia. Ahora, durante la pandemia también, el aislamiento social, no el COVID propiamente, sino el mismo aislamiento generaba mucha ansiedad, mucho estrés, y era una de las causas principales de consulta. Por eso es que también nuestra demanda en salud mental era alta y teníamos apoyo en ese momento de los psiquiatras, de los psicólogos, de los hospitales que estaban en reboto, que no podían atender, pero estaban atendiendo a través de la línea. Entonces, ahí nosotros ampliamos nuestros servicios y tratamos de disminuir el estrés en la población por el aislamiento, por la muerte de los familiares, por su misma ansiedad de morir, porque el COVID al final eso nos generó mucha ansiedad en nosotros y en nuestros familiares que puedan fallecer. Y sí fue muy importante esa intervención.

**E:**Y coméntenos, ¿qué estrategia ha aplicado usted o ha visto que se ejecute para que los usuarios del sistema de salud adopten el uso de intervenciones digitales en salud mental como teleconsultas? Por ejemplo, ¿qué tipo de intervenciones o estrategias para que estas personas puedan adoptar, de repente personas que no están tan involucradas en herramientas digitales? ¿Cómo estas experiencias han logrado rellenar a los usuarios en este sistema de salud?

**e:**Lo que pasa es que el uso de la tecnología agiliza la atención. ¿Cómo así? Estamos hablando de que un usuario de salud espera a veces un mes, dos meses, tres meses para que lo atienda un especialista X. En cambio, con el uso de las tecnologías, el usuario ha ido aprendiendo de que lo pueden atender más pronto. Una vez al mes, una vez cada dos meses y en el caso de nosotros, cada vez que lo necesite. Y eso va a favor nuestro porque quiere decir que lo van a seguir utilizando. Así es. Quizás, digamos, como conclusión ha sido como una recomendación que una persona que ha vivido la experiencia ha recomendado a otra y así sucesivamente. Sí, exacto. Y es la oportunidad de atención la que ha aumentado. Antes no tenías la oportunidad de ir a sacar una cita. Ahora tienes la oportunidad de solo marcar un número y comunicarte con un solo.

**E:**Correcto. ¿Y qué estrategias de comunicación o difusión se utilizan para promover el uso de los sistemas de teleconsultas, de las atenciones u otras intervenciones digitales de salud mental aquí? ¿De qué manera promovemos que sea más conocido de repente el canal?

**e:**Lo hacemos por televisión, por radio nacional. Estamos en los circuitos cerrados de los Bancos de la Nación, en el metro de Lima. Hay carteles que se están colocando. Desde el inicio de la pandemia no había esa opción de la opción 5. Si no se implementó y se quedó. Y ya ahorita en todos los medios de comunicación, como te digo, que tengan que hablar sobre suicidio, sí o sí tienen que poner el número y la opción para poder abrirle la puerta a la persona que tenga la necesidad.

**E:**Correcto. Y de repente, específicamente para la línea 5 que es salud mental, de repente ¿cuál de todas las formas de difusión ha sido de repente la que usted considera la más exitosa o la que ha tenido más impacto?

**e:**Televisión. Los medios masivos son los que tienen más impacto. A los minutos empieza a llamar la gente. Y empieza a llamar en picos, pero después los que verdaderamente necesitan se mantienen y seguir llamándonos. O sea, primero llaman como para probar, ¿no? Para probar si es que nos están contestando. Pero después ya la gente que sí necesita empieza a seguir llamando. Y hemos tenido casos de poder detectar ideaciones suicidas, por ejemplo, que hemos tenido que intervenir ya sea con policía, con serenazgo, o le hemos pedido la ayuda a SAMU para que se pueda dar una intervención más completa y no quedarnos con el servicio de tele-salud, con la teleorientación telefónica, porque ya llega un momento en que necesitas hacer la atención presencial. Entonces, en ese, nosotros por ejemplo estamos trabajando en hacer esa integración de lo digital con lo presencial.

**E:**Una consulta, afundando en la estrategia, ¿no? De la atención. La teleorientación que se brinda es más que nada como un proceso de tamizaje o de triaje, digamos, en salud mental, o también se afunde en el tratamiento, en el seguimiento y con el cerebro.

**e:**Ya, lo que pasa es que, tele-salud, uno de los ojos de la salud es la telemedicina. Ya, en la telemedicina hay teleorientación donde no das medicamentos. Ya, teleconsulta donde sí ya podrías dar medicamentos y en este caso sería pues un psiquiatra que tendría que dar medicamentos, no un psicólogo. Lo que es telemonitoreo es cuando ya tú le haces una o dos o tres llamadas de seguimiento. Ya, y bueno, ya lo que es teleinterconsulta entre establecimientos de salud. En estos tres primeros que estamos hablando, de esas tres formas estamos interviniendo. ¿No? En ajustar los medicamentos con los médicos que tenemos, en la teleorientación, en darle todo lo que es el sostén emocional del caso, de acuerdo a lo que sea, por ejemplo violencia, el sostén emocional en el caso de violencia. Ese tratamiento inicial y ese seguimiento posterior hasta para ver que lo atiendan o si es que se tuviera que internar o tuviera que recibir medicación de otra manera. ¿No? Hacer ese enlace. No puede quedar la tele-salud sola, tiene que enlazarse con todo el sistema de salud.

**E:**Correcto. Justo nos mencionaba anteriormente sobre la parte de lo que es las referencias. ¿No? Entonces, más o menos como usted nos indica, ustedes acompañan que justamente la persona, a pesar de que regresen nuevamente al primer nivel para que haga la referencia por la manera que existe actualmente, igual le hace el tema del acompañamiento, el seguimiento, ¿no? Para asegurarse que haya habido, digamos, esa referencia bajo la forma actual que se realiza.

**e:**Para que llegue al centro de salud mental comunitario o llegue al hospital de salud mental, de acuerdo sea a la necesidad. Porque hay algunos que no lo quieren, ¿cierto? Pero yo creo que sí, a esos hay que hacer seguimiento.

**E:**También nos contaba un poco sobre otras formas o otros canales que se utilizan en estos tipos de seguimiento monitoreo, intervenciones. Uno de ellos es la oficina del teléfono, otros canales también que nos…

**e:**Tenemos correo electrónico, tenemos mensajería instantánea por WhatsApp y por Telegram. Y ahorita estamos diseñando, como te decía, una aplicación móvil y un website que se va a unir al punto gob.pe, porque el punto gob.pe es una página plana del Estado. Pero estamos haciendo una diseñada más amigable para que justamente sea más accesible para los temas de salud mental. Pero que se tiene que unir al punto gob.pe por indicaciones del gobierno.

**E:**Así es. Y de repente nos comenta un poco de algunos canales que ya vienen usando, si bien tradicionalmente la línea telefónica es la que más se ha hecho conocida, de repente hay otros…

**e:**Correo y WhatsApp son muy conocidos, sí.

**E:**Ah, correcto. Y coméntenos de repente cuáles, digamos, son los públicos objetivos para distintos canales que ha podido observar, ¿no?

**e:**Mira, la mayoría de población que nos llaman, uno es, delinea callado, no te voy a mentir, la mayoría de Lima y Callao, es adultos, ¿no? de etapa adulta. Hay jóvenes, hay adultos mayores, pero de etapa adulta es la mayoría. Y público femenino es el que más se llama, también, ¿no? Ahora, en ese sentido nosotros hemos hecho ciertos convenios, ciertos acuerdos con otras líneas. Por ejemplo, con Sí se ve, que ve bullying, que ve violencia escolar. Entonces, cuando nosotros detectamos un caso de violencia escolar, nosotros le derivamos la llamada para que ellos hagan la intervención del caso. Vayan al colegio, hagan todo lo que ellos tienen que hacer como Ministerio de Educación. Lo mismo ocurre con lo que es el programa Aurora, Aurora la línea 100, violencia contra la mujer. Igual, detectamos un caso, nosotros le damos el soporte emocional, pero para el otro soporte, el que es por abogados, por policía, por intervención, derivamos ese caso al programa, ¿no? Para tratando de complementar toda esa atención en salud mental porque es compleja en realidad. Todo, son estrategias que estamos tratando de emplear para abarcar mayor cantidad de problemas públicos que tenemos.

**E:**¿Existirá algún otro aliado de repente que también usted ha podido observar, o de repente que cree o considere que se debería sumar?

**e:**¿Un aliado? No se cuenta, ¿verdad?

**E:**No se preocupe. Ok, ahora vamos a hablar respecto específicamente de las barreras facilitadoras y necesidades.

**e:**Bueno, uno más, pensión 65. Estamos llamando a los adultos mayores, primero que nos dan una lista, los de pensión 65, sobre todo de las zonas de desastre, de dengue y de lluvias. Entonces, los estamos llamando y de acuerdo a lo que requieren, les estamos haciendo la teleorientación, ya sea por medicina, psicología, en tratamiento de psicología, nutrición, etc. Entonces, ahí también ese es otro aliado más, los de pensión 65.

**E:**Ok, entonces ahora sí vamos a entrar sobre las barreras facilitadoras y necesidades. ¿Qué problemas ha encontrado o qué barreras ha identificado en la implementación o uso de estos servicios de teleconsultas? ¿Cuál ha sido de repente la mayor barrera o dificultad y por qué cree que es la más importante? En general, ¿no? Nos ha contado un poco de las estrategias, nos ha contado los canales, nos ha contado el público objetivo, ¿no? Y de cada una de ellas, ¿cuál es la barrera o dificultad que se le viene a la mente?

**e:**La cobertura del MTC. Porque tenemos que no todo el país tiene fibra óptica, por ejemplo. Si bien es cierto, casi todo el país, cada vez más, todos tienen teléfono, hay sitios donde no hay comunicación todavía, no podemos llegar. No estamos llegando no porque no queramos, sino porque no podemos. Todavía no hay conexión de fibra óptica o prendes de repente un televisor para hacer una charla, una conferencia de salud y le quitas la luz a todo el pueblo. Entonces, esa es una barrera que no está en nosotros. Y es grave.

**E:**Y de repente, fuera de la parte tecnológico-técnica, ¿cuál es otra barrera de repente operativa que usted ha visto, ¿no? O encontrado en el proceso de implementación?

**e:**¿Operativa como barrera? No, al contrario. La telesalud te abarata costos, te acerca la salud al ciudadano. En vez de barrera, son oportunidades. Y aunque no lo creas, el COVID, en vez de ser algo malo, fue una oportunidad para poder trasladar las fronteras. Hemos llegado casi a todo el país y estamos llegando casi a todo el país. Entonces, más que barreras, yo consideraría que es algo que ahorita está en boga y que va a seguir creciendo.

**E:**Nos comentaba también respecto a las articulaciones o las alianzas que tienen, ¿no? ¿Ha encontrado de repente alguna barrera o dificultad al momento de poder generar estos casos de cooperación?

**e:**Siempre hay barreras políticas, pero hay que salvarlas.

**E:**¿Y qué facilitadores o, por el contrario, ¿no? ¿O catalizadores ha encontrado para la implementación o el uso de esas plataformas, ¿no? Dentro de todo este sistema?

**e:**¿Que nos ayuda?

**E:**Sí, ¿qué ha visto, no? De repente una oportunidad, de repente, no sé, tanto en el ámbito tecnológico, en el ámbito, digamos, de estrategia, como en el ámbito operativo.¿Qué usted ha visto y ha dicho, bueno, aquí es, aquí podemos?

**e:**En su momento, la dirección de Secretaría Digital de la PCM fue un catalizador. Reniec también fue un catalizador porque yo estuve en la experiencia y he llegado a todos los sitios. Otros organismos gubernamentales como los que manejan el Banco de la Nación, ¿no? El canal de Televisión Nacional también son catalizadores porque sí son, nos ayudan, no son nuestros, pero nos han estado ayudando. Y sí, nos han ayudado a difundir todo el trabajo que hemos venido haciendo.

**E:**O sea, cada vez que de repente ha encontrado una nueva, o una nueva alianza, de repente se ha podido, digamos, incrementar justamente los procesos de cobertura. Y dentro del público objetivo, de repente, ¿cuál es? Uno de ellos que nos mencionaba es el tema de la alfabetización en herramientas digitales, ¿no? ¿Cuál de repente ha sido otro facilitador que usted ha podido observar en el público objetivo, fuera del aprendizaje tecnológico que uno pueda tener?

**e:**Es que lo más fácil es entenderlo. Ahorita, por eso es que es lo que más se ha masificado. Ahorita tenemos prácticamente dos teléfonos por cada persona. Y eso es lo más fácil. Que no sea smartphone, no importa, pero la señal de voz, eso es lo que nos ha facilitado. Cuando Nación InfoSalud solo recibía llamadas de un 0800, de números fijos y de teléfonos públicos. De Movistar.

**E:**¿En qué año fue eso?

**e:**Estamos hablando del 2002. Y hasta el 2017, antes de que yo entrara a la gestión, era tal cual lo que te estoy diciendo.

**E:**¡Wow!

**e:**Solamente teléfonos públicos, fijos, Movistar y sobre 0800. Por ende, casi nadie, ¿no? Ya nadie tiene eso. Ya no hay teléfonos públicos, casi nadie tiene fijos. Y ya Movistar ya no tiene el monopolio, así que... Ahora todos tienen celular. Todos tienen celular. Y más que los otros canales, la señal de voz ahorita es la que más se va a difundir porque llegas hasta el adulto mayor. Llegas desde el niño, que nos llaman los más pequeños, a veces de 8 años, que están con algún adulto. Aunque necesitan alguna ayuda, no saben qué hacer. Hasta adultos mayores que inclusive hablan quechua o aymara y tenemos algunos operadores que hablan quechua y aymara. O sea, eso es ahorita sobre lo que nos estamos centrando hasta que podamos tener la alfabetización digital. Que eso va a demorar. Ustedes sí, pero los más arriba que yo todavía les falta.

**E:**Un poquito nos gustaría también conocer un poco las características de esas personas ya que desde su posición se han podido observar distintos tipos tanto etarios, aspectos sociodemograficos. Y justamente aquellos usuarios que utilizan la línea 5 de salud mental nos comentan que la mayoría son mujeres. ¿Son mujeres más o menos en qué edades? ¿En qué situación?

**e:**La mayoría son mujeres y como te dije de etapa adulta. No adulto mayor, no jóvenes, sino de la etapa adulta de ese etario. Y habitualmente por lo que ellos buscan la ayuda es por estrés, por ansiedad, por trastornos emocionales. Y obviamente consultan por hijos y familiares. Yo creo que la mujer tiene menos temor a la salud. Por eso son ellas las que nos consultan más. Pero los problemas por los que más consultan son estos que te estoy diciendo. Emocionales, estrés, hay otros casos obviamente de violencia y otras cosas, pero no es el mayor tema de llamada.

**E:**Quizás esto también suceda porque en general los varones de la empresa tampoco escuchan la parte emocional. Y en ese sentido, ¿creería usted que quizás nos podamos dirigir más al sector masculino? Como que romper esa barrera, ese temor que puedan tener ellos a abrir a contar

**e:**Yo creo que con los spots que han estado saliendo, porque justamente son varones los que dicen que estoy preocupada, que estoy pensando en suicidar. Con esos spots estamos tratando de abrir esa barrera. Pero sí hay que trabajar, hay que trabajar. No es algo de ahorita, no es algo que años y años. Pero sí hay que trabajar en los niños, sobre todo a expresar sus emociones. Y eso se va a ver en el transcurso de la evolución de todos estos medios digitales.

**E:**¿Qué otras necesidades cree usted que se deberían, muy aparte del tema de, hemos hablado de las derivaciones, también nos ha hablado de mejorar algunas estrategias con nuevos aliados. ¿Qué otras necesidades cree usted que se deberían realizar en la mejora en este caso del servicio? Nos ha hablado también del alfabetización digital, de las personas. ¿Qué otras necesidades cree usted que debería hacerse?

**e:**Una de las cosas que yo sí estoy convencida que va a funcionar es esa aplicación móvil de la que te estaba hablando. ¿Por qué? Porque habitualmente cuando tú, no sé, tienes alguna duda, si estás estresado o no, y tú entras en cualquier aplicación y dice llena tus datos, llena tus datos. Llegas al final, no, mete tu tarjeta. La clásica, ¿no? O cualquiera, crees que tu índice de masa corporal está mal, ya, mete tus datos, te vamos a ayudar, mete tu tarjeta. Ya, lo que yo quiero es tener una aplicación gratuita, obviamente, por el Estado, pero que te dé ese compañero y ese consejo que no te da ninguna de las aplicaciones porque todas son de paga. Que te pueda dar tranquilidad, que puedas tener tu agenda de citas si es que tienes problemas de salud, que puedas tener juegos dentro de eso, que te brinde paz, los ruidos ambientales que también te ayudan a tranquilizar, pero que todos ahorita, o la gran mayoría, son de paga. Yo creo que ese desarrollo que estamos haciendo nos va a ayudar bastante y también nos va a ayudar a esos otros grupos etareos que no estamos cubriendo. A esos jovencitos que de repente les gustan los videojuegos, pero que de repente con un juego de dibujo de otro tipo, de otro estilo, lo va a ayudar más bien a manejar su ira, a tranquilizarse, con consejos y obviamente con un botón de pánico para llamarnos en caso sea necesario. Yo creo que eso es lo que nos podría ayudar y nos va a ayudar bastante. Esa idea por lo menos que tenemos nosotros ahorita.

**E:**Bueno doctora, le agradecemos muchísimo las preguntas. No sé —----si hubiera otro comentario. No, de parte de que creo que esa amiga ha sido una entrevista tan enriquecedora en realidad.

**e:**Gracias chicos. Muchas gracias.

**D3: Tomador_Decision**

**E:------**, muchísimas gracias. Vamos a comenzar entonces con la entrevista. Le voy a pedir, bueno, le voy a hacer una serie de preguntas, luego de repente mi compañero va a complementar con algunas más. La entrevista va a durar aproximadamente unos 15 o 20 minutos. Le pediría, por favor, que para empezar la entrevista, y habiendo aceptado el consentimiento informado, nos diga su nombre, su cargo, y comenzamos con las preguntas.

**e:**Ok, mi nombre es —------, asesor del despacho ministerial en temas de salud mental.

**E:**Sí, su nombre completo, bueno, y su cargo, ¿no? La institución en la que pertenece, por favor.

**e:**Ministerio de Salud.

**E:**Ok, comenzamos entonces con la entrevista. Coméntanos, doctor, ¿qué opinas sobre, en general, sobre el uso de las intervenciones digitales, específicamente relacionadas con telesalud, para la atención de problemas de salud mental?

**e:**Bueno, que hubo mucha expectativa al inicio de la pandemia, ¿no? Ante las dificultades para mantener los servicios, en general, ¿no? Los servicios de salud. En salud mental no tuvimos tanta dificultad porque en los últimos años habíamos implementado servicios de salud mental en la comunidad, ¿no? Y durante la pandemia también, que permitieron llegar a gran parte del territorio nacional, casi el 80% de las provincias del país. Y eso nos facilitó el que se mantenga el contacto con la población, que ya venía recibiendo servicios. Fue una ventaja el no estar dentro del hospital, ¿no? Como sí para otros servicios que tuvieron que, digamos, cerrar la prestación presencial, dado que los hospitales se dedicaron por completo al COVID, ¿no? Entonces, este, de modo que en ese aspecto, en salud mental hubo un panorama diferente en nuestro país,

como en muchos de los países donde tienen servicios de salud mental comunitaria, que pudieron mantener el contacto con la población, a diferencia de otros servicios, ¿no? El contacto con los usuarios, especialmente. Sin embargo, digamos, los medios digitales

sirvieron mucho más bien para difundir, hacer comunicación a la población en general, por los diferentes canales digitales, de modo que desde los propios servicios de salud mental, ya en cada región, en cada territorio, hicieron mucha prevención, mucha difusión sobre prevención, y también sobre, para poder mantener el contacto con la población, ¿no? Esa fue en general la experiencia, en realidad, los propios servicios de salud mental en cada territorio. Tempranamente, los equipos intentaron implementar la, digamos, la atención digital individual, este, sin embargo, una gran barrera es, pues, que la población no tiene un buen acceso, ¿no?, a servicios, inclusive aquí en Lima, se cae con frecuencia, digamos, la señal, ¿no?, y recién está entrando la, digamos, tecnología de la línea óptica, que hace posible que se pueda mantener de mejor manera la comunicación, ¿no?, por los diferentes canales digitales, pero recién en Lima, ¿no?, y en salud mental, en general, los tiempos de consulta son mucho más largos, ¿no?, e inclusive los tamizajes y las propias intervenciones abarcan más de media hora, ¿no?, para que sean realmente efectivas, y eso demanda que la señal sea buena, aparte de que tenga datos el usuario, ¿no?, que así tuviera datos, que la señal misma sea buena, y en los propios distritos más pudientes acá en Lima, la señal es muy inestable todavía, ¿no?, y entonces son situaciones que se vieron desde tempranamente que limitaron, a pesar de los esfuerzos, ¿no?, de digitalizar las comunicaciones, especialmente la atención individual, que disminuyeron la posibilidad de hacerlo y que realmente reemplace, digamos, la atención presencial, ¿no?

**E:**Sí, justo nos ha dado un panorama general acerca de quizás de todo el contexto, su apreciación, también de repente los procesos que ha involucrado, y algunas barreras y facilitadores que usted nos ha comentado. De hecho, vamos a ir desmenuzando con las preguntas, algunas de ellas, vamos a enfocarnos primero en esta primera parte, que son dos preguntas, nos vamos a enfocar en lo que son normas y políticas, luego vamos a hablar de la experiencia, así de la intervención, y vamos a finalizar con algunas preguntas relacionadas a barrera, facilitadores y necesidades. Para empezar entonces con las preguntas relacionadas a normas y políticas, específicamente relacionadas a acceso y cobertura, la pregunta sería ¿qué políticas, normas o guías existentes, existen o guían la implementación o el uso de estas intervenciones digitales en salud mental? ¿Algunas de estas políticas, normas o guías particularmente importantes considera que haya influido en su trabajo?

**e:**Bueno, la única política que existe en el Ministerio de Salud es la política de salud 2031, o sea, no hay otra política formal, la única política que existe, y no incluye específicamente este tipo de intervenciones, de modo que ahí hay un desarrollo pendiente. En segundo lugar, respecto a normas específicas, son las normas propias de telesalud que fueron emergiendo durante el año 2020 y 2021, que trataron de regular las iniciativas que fueron empezando a emerger en todo el territorio nacional, en los diferentes servicios, para llegar mejor a los ciudadanos usuarios de los servicios. Creo que eso de alguna manera facilitó el tener, primero, el estandarizar un lenguaje, el poder codificar las actividades para poder ser registradas, y también de modo que podamos tener data para evaluar estos procesos también. Entonces, de modo que sí esas digamos fueron normas específicas que me parece que contribuyeron a la implementación de algunas iniciativas, o al inicio del intento de estandarizar estos procesos.

**E:**Y en su opinión, ¿cuáles? Bueno, nos habló un poquito quizás en la introducción que nos dio, pero para consolidar la pregunta respecto a normas y políticas, ¿cuáles serían los factores que influyen en la capacidad de la cobertura y atención que pueden tener los servicios de salud mental digital, como teleconsultas o teleatenciones? Específicamente en relación a normativas y políticas, ¿no?

**e:**Perdón, me podrías volver a preguntar, no escucho bien.

**E:**Sí, no se preocupe. En relación a las normas y políticas, ¿cuáles son aquellos factores que influyeron en la capacidad de cobertura y atención de estos servicios de salud mental digital? En las teleconsultas o teleatenciones.

**e:**Bueno, no vinculados específicamente a las normas, sino a los recursos que se requiere para implementar este tipo de intervenciones. Obviamente que se requiere tener el know-how, y recién el ministerio tenía el know-how al respecto, la dirección correspondiente de telesalud era una dirección todavía, digamos, emergente, y creo que esa fue una limitación que se tuvo, pero no solo en salud mental, sino en general. También los recursos para la implementación, los recursos en los propios servicios, es decir, que todo esté computarizado, que haya una historia clínica, electrónica, que hubiera una buena red de conexión, y no lo hay en el país, que los usuarios tuvieran datos, inclusive celulares para poder, o aparatos para poder llegar. En especial el ministerio de salud tiene esa gran dificultad que atiende a 20 millones de personas que están aseguradas al SIS principalmente, pero que justamente están aseguradas al SIS porque tienen muy pocos recursos económicos. Entonces la proporción de personas, de acuerdo a los reportes del INEI, que no tenían en ese momento, en el 2020-2021, un celular o una computadora, era una proporción muy importante, en algunas regiones del más del 50-60%. Pues podía tenerse todo implementado, pero si los usuarios no hay una red suficiente, y los usuarios no tienen ni los equipos ni los datos para la conexión, entonces en realidad, para instituciones del ministerio de salud que tienen una población objetivo con esas características económicas, resultaba una herramienta importante, pero digamos, no suficiente.

**E:**Claro, doctor. Para cerrar este apartado de normas y políticas, un poco de lo que hemos comprendido de lo que nos comentaba, es de que las soluciones iniciales al contexto de pandemia respecto a salud mental y a través de los centros de salud mental comunitaria, respecto a telesalud, fueron hacer parte del sistema las atenciones, por ejemplo, con los códigos de registro de teleatención, telecapacitación, para los llenados de reportes GIS y FUAS, para el CIS. En ese sentido, luego de esta etapa en la que ya se le dieron las herramientas a los centros de salud mental comunitaria de que ya pueden registrar a estos pacientes que atendían en esta modalidad, luego existió, tal vez no una guía, no un documento formal, pero capacitaciones de parte de DESAME o de otra área del MINSA en coordinación con DESAME respecto a cómo atender en telesalud. ¿Alguna guía para el personal de salud?

**e:**Se recibieron capacitaciones de parte de telesalud a nivel nacional sobre el uso de los codificadores para cada tipo de intervención, en fin, la aplicación de todo este procedimiento de estandarización. Eso se desarrolló a nivel nacional en varias oportunidades, de modo que ese fue un esfuerzo de la dirección de telesalud que coordinamos desde la dirección de salud mental y que sí fluyó. Sin embargo, creo que tardó, la dirección de telesalud tardó buenos meses para poder hacer esta definición normativa. Y entonces, claro, eso hizo que se desperdiciara mucha información. Sin embargo, especialmente del año 2020, no, sin embargo, no significa que no se dio este servicio. O sea, creemos que hemos perdido mucha información porque hubo una reacción espontánea muy importante, ahora que menciona los centros de salud mental comunitaria, del personal de los centros de salud mental comunitaria, por iniciativa propia y dado también la etapa de vida de los operadores de salud de los centros de salud mental comunitaria, porque eso también influye, ¿no? Como son servicios nuevos, el personal que trabaja en estos servicios es personal joven, que está entre los 25 y 35 años y que están muy, o ya venían muy familiarizados con el manejo de, digamos, la tecnología, las herramientas tecnológicas, sin ser profesionales en tecnología. Entonces, este, eso facilitó que rápidamente hubieran iniciativas que a nosotros nos llamó la atención ya desde marzo o abril, ¿no? Que empezaron a implementar sus centrales en cada región y empezaron a hacer las teleconsultas, ¿no? Y nos llamó poderosamente la atención, pero veíamos con mucha frustración la pérdida de la información, porque no podía registrarse todo ese trabajo que iban realizando, inclusive sin horario, ¿no? Porque en esa época se perdió la noción del tiempo, ¿no? Y había que atender las demandas, especialmente, por ejemplo, del Ministerio de la Mujer para los casos de violencia o intentos de feminicidio que no dejaron de darse a pesar de la pandemia, ¿no? Entonces, este, sí, hubo un esfuerzo de capacitación, pero creo que tomó un tiempo y lo importante es que se dio la atención, ¿no? Pero que los usuarios pudieron tener el servicio, pero lastimosamente se perdió mucha información, en mi opinión, ¿no?

**E:**Ahora vamos a pasar justamente a profundizar los temas relacionados a las experiencias. Nos ha contado un poquito ya sobre algunas experiencias que ha podido tener con el equipo, ¿no? Que ha desarrollado diversas iniciativas. Si podías resumir en general cuáles son aquellas experiencias en la implementación del uso de estas intervenciones, ¿cuál sería?

**e:**Fundamentalmente, la implementación de las teleatenciones, ¿no? Desde los centros de salud mental, que fueron, digamos, en una modalidad que facilitó una modalidad de atención híbrida, ¿no? Porque al tener estos servicios en los territorios, y estos ya venían

abastecidos, sus farmacias, ¿no? También en salud mental hay uso de medicamentos, ¿no? Pudo mantenerse la atención y la entrega de manera virtual o presencial, de manera híbrida, ¿no? Y, este, lo cual facilitó que no hubiera pérdida de contacto con los usuarios y que pudiera entregarse inclusive la medicación, ¿no? A diferencia de países donde no tienen este tipo de servicios, y no hubiéramos tenido, solo hubiéramos tenido los hospitales o servicios de psiquiatría o solo los hospitales psiquiátricos en Lima, ¿no? Que están en Lima. No, no, por más que implementáramos este tipo de intervenciones digitales, ¿no? No hubiera sido posible. Pero creo que eso fue, este, muy importante. También el que puedan, estas teleatenciones, no solamente implicar teleatenciones médicas, ¿no? Sino de todas las especialidades del equipo multidisciplinario, ¿no? Tempranamente vimos que empezaron a implementar, por ejemplo, la teleatención en terapia de lenguaje que se da en estos servicios, porque tenemos una gran población,

especialmente de menores de 12 años con problemas del neurodesarrollo, que implican problemas del lenguaje, ¿no? Y que ya venían recibiendo sus terapias y nosotros veíamos con alegría cuando llegaban a los servicios los esfuerzos que hacían los terapeutas ocupacionales, ¿no? Este, desde los servicios de salud mental, específicamente los centros, que a través inclusive de su celular, ¿no? Este, continuaban el protocolo de atención del lenguaje con los niños y sus familiares o sus cuidadores, ¿no? También la implementación temprana de las actividades, este, grupales. En salud mental se utiliza mucho la psicoterapia grupal, los grupos de ayuda mutua, ¿no? Este, y también eso fue posible implementar, este, tempranamente, especialmente para los problemas vinculados a adicciones, ¿no? Este, las terapias grupales son muy, muy eficaces y para la regulación por el propio grupo. Y no se perdió el contacto, ¿no? En muchos lugares se implementaron prontamente este tipo de intervenciones específicamente mediante Zoom o mediante Meet, ¿no? Entonces, con múltiples personas. Y como no había restricciones en el Zoom, digamos, este, pudieron implementar con relativa facilidad. Pero lo que más hubo fueron webinars, ¿no? Entonces, este, que se implementaron también en muchos lugares, este, para sus propias localidades, los webinars y con mucho éxito para el lugar, ¿no?

**E:**Sí. Ahora, pasando a la siguiente pregunta, porque nos ha comentado justamente la pregunta que iba a continuar, que era sobre las estrategias que ha ejecutado para poder, este, retener a estos usuarios. Nos ha comentado un poco del hecho de que los, estos usuarios ha podido integrarse a todo un sistema de atención, la atención integral en salud, para otras especialidades. Y cuéntennos, justamente, entre estas estrategias, qué, qué estrategias de comunicación o difusión ha visto usted que se han utilizado y que han sido exitosas para que justamente estas personas conozcan acerca de estos servicios y puedan, este, y pueda haber demanda, ¿no? En este caso.

**e:**Sí, bueno, fundamentalmente me parece que, y que ocurrió prácticamente en todo sitio, como les comentaba, la ventaja que se tenía desde estos servicios de salud mental era que tenían equipos muy jóvenes y muy familiarizados con los medios de comunicación virtuales, ¿no? Y tempranamente lanzaron sus campañas de comunicación, ¿no? Para difundir los servicios, las líneas de ayuda, a través de, de, de sus líneas de fe. Todos crearon su, su canal de fe, su, su, su dirección de Facebook, ¿no? Este, muchos exploraron otros, otros canales, ¿no? Algunos han creado sus canales de YouTube, ¿no? Este, y para hacer difusiones más o menos continuas, este, luego de unos meses ya también empezaron a utilizar TikTok, ¿no? Para difundir, este, mensajes y, este, y de toda índole, tanto preventivos como para promocionar el servicio de recuperación, ¿no? Entonces, este, creo que eso fue una ventaja que no se tuvo en otros servicios, el que tengamos personal muy joven, ¿no? No vimos lo mismo porque también tenemos servicios de salud mental en centros de salud del primer nivel, o sea, tenemos alrededor de mil doscientos centros de salud que tienen servicios de salud mental con profesionales de psicología, pero buena parte de estos son nombrados, ya es personal, que tienen, digamos, más de quince, veinte años de servicios y que probablemente por la edad no estaban tan familiarizados con, con este tipo de, de, de, de instrumentos y si con ellos inclusive hasta las propias capacitaciones no fluía tan rápido, ¿no? Como, este, con, con el personal de los centros de salud mental comunitarios, ¿no? Que, como a patito, por, por suerte, estaban justo en la edad, porque no fue nada planificado, fue una coyuntura.

**E:**Así es. Justo vamos a ingresar a hablar un poquito acerca de las barreras, los facilitadores y necesidades. Nos ha ido comentando un poco en cada una de las anteriores, de hecho, nos quedan cinco minutos. Lo que sí, por ejemplo, me gustaría puntualizar es que en un servicio de telesalud, de hecho, hay procesos que el paciente pasa por experiencia, ¿no? Desde, por ejemplo, desde encuentro el canal de comunicación, quiero agendar una cita para poder teleatenderme con un profesional de que, por ejemplo, se pueda reservar en tal fecha o tal hora, recibo la comunicación en que pueda recibir la atención y posteriormente, quizás, una recomendación final, una derivación, en ese caso. En todo ese proceso, fuera del alcance tecnológico, porque nos han comentado en múltiples ocasiones de que el tema de la cobertura de internet, de la tecnología, a veces son recursos difíciles de alcanzar por la mayoría de la población. Pero respecto a las operaciones, ¿cuáles son aquellas barreras que usted ha podido encontrar? Nos ha comentado justamente que a veces el personal de telesalud también no tiene, digamos, suficiente habilidad en recursos digitales, probablemente es una transición que va a tener que suceder, pero en tema operativo, ¿ha encontrado alguna barrera o de repente necesidad que cree usted que se debe desarrollar y mejorar en estas operaciones?

**e:**Sí, fundamentalmente la plataforma de citas digitales, ¿no? Entonces, este, en ese momento no se tenía y fueron iniciativas muy locales, ¿no? Para poder empezar a hacer el agendamiento de citas y luego, claro, el Ministerio avanzado y ha ido poniendo las plataformas de citas que ha facilitado, pero al inicio sí fue una barrera muy, muy, muy importante, ¿no?

**E:**Y por el otro contrario, ¿qué facilitadores o catalizadores ha encontrado para que, o sea, estas teleconsultas se realicen eficientemente dentro del sistema de salud? Nos ha comentado justamente que a veces los equipos de personal joven ha ayudado muchísimo en la implementación de muchas iniciativas, ¿no? ¿Qué otras facilitadores o catalizadores ha encontrado de repente en el ecosistema, no? Probablemente, de repente se me ocurren algunas instituciones de apoyo, etcétera. Coméntenos un poquito respecto a ahí.

**e:**Bueno, sí, o sea, en realidad también la teleatención demanda de recursos blandos, ¿no? De el operador, ¿no? Que hace la prestación, ¿no? Más aún si se tratan de intervenciones prolongadas en el tiempo, ¿no? Entonces, este, ¿qué tiene que ver inclusive con el manejo de la cámara, el mantener una conexión emocional a través de la cámara, no? Esos recursos son muy importantes de desarrollarlos para justamente generar fidelidad o adherencia al tratamiento, que puede ser una barrera, eso en, digamos, las intervenciones a través de la tecnología, el no poder sentir cerca emocionalmente a la otra persona, ¿no? Pero creo que fue bien importante que hubiera, que esto también se pusiera en debate tempranamente. Nos pudimos dar cuenta nosotros de que había que desarrollar este tipo de habilidades para asegurarnos el contacto emocional con el usuario, ¿no? Que no solo en salud mental es muy importante, ¿no? Sino en general. Y gracias a que nos dimos cuenta, se puso en debate esto, se pudieron desarrollar una serie de mesas, de capacitaciones para discutir esto, porque tampoco no había nada escrito, ¿no? Entonces, ahí hubieron instituciones que nos ayudaron, como la sociedad psicoanalítica, como algunas agrupaciones o colectivos de profesionales de psicología de la sociedad civil organizada, ¿no? Entonces, que vieron, digamos, este, también poner, digamos, sobre el tapete el hablar sobre quién es el que interviene a través de la cámara y no solamente a quién se interviene, ¿no?

**E:**Bien, doctor. Y ya para finalizar, nuevamente gracias por su tiempo. Tenemos una pregunta final. Entonces, vemos que la casuística en el Perú es que tenemos un buen cuerpo de profesionales de salud mental jóvenes, por un lado, y preparados en su formación propia, ¿no? Como profesional de salud, que le ha permitido, digamos, solucionar esta situación, este contexto de pandemia, tal vez sin una guía política

o necesariamente con un documento a seguir, sino que las estrategias que han utilizado nacen también de su propia expertise como profesional, ¿no? La facilidad tal vez de algunas redes, de los grupos cautivos, ¿no? Como nos mencionaba, de grupos de tratamientos de adicciones, tal vez algunos casos derivados por fiscalía u otros ministerios que sí o sí tendrían que darse, entonces facilitaba el acceso o la permanencia del servicio como tal. Y me queda en duda la última sección que nos comentó, que hubieron otros actores en los que apoyaron en el soporte de salud mental, ¿no? Por ejemplo, el colegio de psicólogos, colegio médico, entre otros centros, brindaron atenciones de salud mental, inicial, tamizaje probablemente también, y de soporte emocional. ¿Cuál es la posibilidad de que una persona que tal vez acuda a un servicio de telesalud, ¿no? Que puede ser de estos grupos como colegios de psicólogos, otros colegios profesionales, otro servicio del MINSA, suponiendo que tal vez en el 113 hubiera un servicio de tamizaje como tal. ¿Cuál es la posibilidad de que esta evaluación con un informe hecho por un profesional de salud mental, no psiquiatra o psicólogo, pueda servir como insumo para ingresar allá al sistema de salud con un centro de salud mental comunitario? ¿Las formas en las que el centro de salud mental comunitario actualmente recibe a sus pacientes para atención de patologías complejas, permitiría que estas personas con un informe puedan ingresar al sistema?

**e:**Bueno, sí, ¿no? Este, o sea, el centro de salud mental comunitario funciona como cualquier otro establecimiento de salud en la red de referencia y contrarreferencia, ¿no? Entonces, este, de modo que, digamos, normativamente tiene que entrar por el centro de salud más cercano a donde está escrita la persona, y luego derivarse. En los centros de salud mental hay una disposición que esté en la norma técnica para que por lo menos la primera atención, de todas maneras, sea acogida aun cuando no es derivado desde un establecimiento de salud, ¿no? Este, y evaluado, y de repente derivado a otro, o poder seguir con el tratamiento ahí según la evaluación. Entonces, esto sí es posible. Es más, tenemos ya una experiencia, algunas experiencias, ¿no? Una fue con socios en salud, ¿no? Que implementó un chatbot, este, que nosotros quisimos implementarlo en el MINSA, pero por los temas normativos, ¿no? Este, no fue posible. Entonces, se implementó en la DIRIS Lima Norte, ¿no? Con socios en salud. Y sí, fluyó, había un tamizaje específico de acuerdo a las normas del MISA, y luego una consejería, y luego una derivación, ¿no? Y hay hasta una publicación científica al respecto, y da cuenta de que, bueno, fluyó el proceso, ¿no? También hay otra experiencia respecto a la línea 113, está la opción 5, ahí hay un pool de psicólogos, ¿no? De profesionales de psicología, que durante la pandemia funcionó también inclusive con apoyo de la sociedad civil, pero ahora ya es personal de... Porque el voluntariado es difícil de mantenerlo eternamente, ¿no? Entonces, este, y ahora ya TeleSalud tiene su equipo, ¿no? Y se atiende un volumen importante. Hacen como una consejería y la orientación, y generalmente se vincula con los servicios correspondientes, ¿no? Entonces sí es posible implementar en nuestra opinión. Hay que ver también un poquito, digamos, el perfil de la población. La población que llama al 113, opción 5, es población que está pasando por alguna situación angustiante, fundamentalmente, ¿no? Y entonces llaman a la opción. Entonces, para un psicólogo inclusive sin aplicar el tamizaje, porque los tamizajes generalmente son aplicados o autoaplicados o por profesional no especializado en salud mental, ¿no? Este, cambio, un psiquiatra o un psicólogo no necesita de eso porque alto que es uno mientras está en la entrevista le evalúan, ¿no? Entonces, pero hay esta opción, la de la... Lo del chatbot de socios en salud es importante tener en cuenta que tuvo que agregarse una estrategia para que funcione, porque se lanzó y no funcionaba tanto. O sea, inclusive pagaron para que esté en las redes y todo, ¿no? Este, aparezca en chatbot, pero no se usaba tanto. Entonces tuvieron que implementarse una estrategia adicional para llevar el chatbot, ¿no? Con orientadores, ¿no? Llevarlo a la población, es decir, con agentes comunitarios y que la población... Hacer un barrido con el chatbot, o sea, era como hacer un tamizaje digital. Este, y eso también es importante tener en cuenta porque sí tuvimos una experiencia donde no nos fue bien, que fue con profesional de salud. Porque teníamos la necesidad de dar el apoyo al profesional de salud, ¿no? Especialmente a los que estaban en servicios, este, altamente sensibles, ¿no? En primera línea. Sí, en primera línea. Y desgraciadamente lanzamos primero, digamos, una línea específica para esos profesionales, ¿no? Y no llamaban, no... Y luego vimos reportes de otros países que nos decían que eso tampoco estaba funcionando, ¿no? Este, y que... Es que el perfil del profesional de salud para salud mental también es bien particular, ¿eh? O sea, y creo que tiene que ver en general con el estigma que hay de hablar sobre los propios problemas de salud mental en el ámbito laboral, como en cualquier otra profesión, porque hay temores a poder ser objeto de, digamos, que la información se filtre y ser objeto de exclusión o algún tipo de reacción del sistema laboral. Entonces, este, eso creo que también, esa sí fue una experiencia adversa que no prendió para nada. Y tuvimos que hacer lo que recomendaba OMS, que era poner equipos de soporte psicosocial ahí en las UCIs y en los servicios de emergencia o en los servicios COVID que acompañaran en la atención, pero que también a la vez estuvieran vigilando, digamos, de alguna manera, el estado emocional de los trabajadores, ¿no? Sí, eso es lo particular.

**E:**Sí, de hecho tuvimos una experiencia muy parecida con personal de salud a nivel nacional, en el que, como usted nos comentó en su caso de socios de salud, el tener que salir a campo y tener facilitadores más cercanos a ellos fue vital, sino realmente que no se hubiera podido cumplir las metas. Sí. Bueno, doctor Yuri, muchas gracias por su tiempo. Ha sido muy valiosa el poder compartir estas experiencias de parte de SAME en el contexto de pandemia, usando sobre todo estas soluciones digitales, que seguramente en el futuro muy cercano ya no serán una novedad, ¿no? Y sino ya parte del proceso normal de atención. De hecho, 113 ya es un primer bosquejo de cómo va a funcionar este sistema. Y de parte de CENSOPAS, muchas gracias por su participación. Y también tenga por seguro que finalizado el estudio de SAME también va a contar con el informe de los resultados de este sistema, que va a estar disponible para utilizar si es que fuera necesario implementarlo para sus propios fines, ¿no?

**e:**Ok, muchas gracias.

**E:**Muchas gracias, doctor.

**e:**Gracias a usted. Hasta luego.

**D4: Trabajador_Profesional_Salud**

**e:**Mi nombre es —------, soy psicóloga, psicoterapeuta. Bueno, en tiempo de pandemia era la responsable del servicio de adicciones, sin embargo en la actualidad soy la jefa del Centro de Salud Mental Comunitario de Los Valles.

**E:**Ok, muchas gracias. Entonces, en esta entrevista, sin exceder, tratamos de explorar un poco el tema de la pandemia, de la situación de la pandemia y a los usuarios de la telemedicina. Estamos viendo como varios dispositivos, no una recomendación. Entonces, cuéntame brevemente, ¿Durante cuánto tiempo las el servicio de adicciones de salud mental se acoplaron a través de teleatención y cuál ha sido su experiencia en la teleatención de medicina?

**e:**Bueno, desde marzo del 20202 cuando inicia la pandemia, el comunitario se acopla a la necesidad que se estaba dando en el tema de la atención virtual. Como estrategia en ese momento se hizo un piquete. Habian usuarios que venian aqui de manera presencial y venían cumpliendo todo el protocolo pero nosotros los atendiamos de manera virtual. Esto es virtual, el protocolo implicaba llamada telefónica, porque algunos todavía no contaban con equipos con mucha tecnología, videollamadas en algunos casos o lo que es un Zoom, un Zoom.

**E:**Ok, entonces, entrando ya un poco en el principio de este proceso formal, en lo que el servicio de adicciones sí se contabilizaban, así lo que se está haciendo aquí, no se ha acudido. Entonces existia algún protocolo con el que se realizara la teleatención?

**e:**De parte de DIRESA Callao se nos enviaron las codificaciones para el GIS y si fue iniciativa del jefe poder hacer un formato donde se hacia la teleatención. Tenemos 4 servicios, el poder especificar el tipo de intervencion y poder detallar la sesion como una hoja de historia

**E:**El contacto con DIRESA fue la guia instructiva mas el reporte...

**e:** Claro un formato estandarizado para los 4 servicios para poder hacer el llenado de la atención virtual

**E: [NO ES INTELIGIBLE LO QUE DICE EL ENTREVISTADOR]**

**e:**Bueno hubo capacitación del parte del MINSA explicando cuál iba a ser el trabajo que se iba a realizar en virtual, pero de inicio porque esto nos cogió por sorpresa. Por eso, cada uno hizo lo mejor que podía en ese momento. Sin embargo, en las reuniones de equipo que teníamos se daba la retroalimentación, porque de hecho no para todos funcionaba el hecho de ser una llamada telefónica. Sino que para niños era necesario una plataforma como esta. 

**E: [NO ES INTELIGIBLE LO QUE DICE EL ENTREVISTADOR]...**¿Qué características debería tener?

**e:**Uno el tema de que debe estar un poco adecuado a la realidad, porque si bien es cierto Callao es provincia, pero sí al nivel económico tiene el mismo acceso de Lima. Entonces, que pueda estar un poco acercado a la realidad. Que pueda ser bastante manejable, porque nosotros tenemos personal SERUMS internos que también no conocen tanto de documentos técnicos, entonces, que sea claro sencillo y que sobre todo permita poder llenar los procedimientos que nosotros realizamos

**E:  [NO ES INTELIGIBLE LO QUE DICE EL ENTREVISTADOR]...**¿Hubo otro medio o otra plataforma que le permitiera hacer eso?

**e:**Desde mi experiencia fueron esos, sin embargo también a veces con algunos usuarios el INSS, el NINSS no dependiendo pero también hay que tener en cuenta que a veces los usuarios de servicios de adicciones contaban con teléfonos básicos. Entonces, no hacer una llamada y tratar de articular con algún familiar para poderles enviar algunas pautas, algunos videos a través de ellos.

**E:**¿Cuáles son las experiencias con cada uno de ellos? Por ejemplo, con llamadas telefónicas, libros, cumbres, ¿qué diferencia sentiste en cada uno?

**e:**En llamadas telefónicas a veces sentía como que era un monólogo, ¿no? Porque a veces el usuario, ajá, sí, o sea, sí. A veces parafraseaba desde su experiencia un poquito de lo que uno le decía, pero quedaba mucho el vacío. Porque si bien es cierto, bueno, era la primera experiencia que yo tenía en una atención virtual y más por teléfono, porque casi siempre se brinda una atención face to face, ¿no? Entonces, era un poco difícil, pero ya conforme uno también va teniendo más experiencia, de repente ya no era tan técnica, ¿no? Y me acomodo un poco más a lo que estaba viviendo en esos momentos, porque a veces hacer psicoterapia como tal por una llamada telefónica es bastante complicado, ¿no? En cuanto al tema del Meet, el tema del Zoom era mucho más fácil para el profesional y también considero que para el usuario, ¿no? Porque compartíamos pantalla, hacíamos material para poder hacer una psicoterapia, porque ahí sí había de repente un encuentro no visual, el usuario también podía un poco ver plasmada la información, entonces era mucho más rico. Ahora, recordemos que en el caso de la Perla, la Perla se considera Perla Alta y Perla Baja. La Perla Alta tiene un nivel adquisitivo mucho más alto, la Perla Baja quizá no. Entonces, el problema eran los datos que tenían que gastar los usuarios y muchos de ellos tenían el tema de que esos datos eran para sus clases, ¿no? O eran un poco limitado, entonces quizá ahí entraba la otra salida, la videollamada, que también ayudaba, ¿no? Porque era un encuentro visual con el usuario, pero no te daba esta facilidad de permitir. Entonces, como estrategia, nosotros hacíamos material muy general, los psicólogos, y lo compartíamos con el usuario como para que tenga una guía o pueda hacer un checklist de cosas que podría hacer en tiempo de pandemia. Muy bien, muy bien, lo que yo comento. Entonces, cuando se refiere a la idea de la perla alta, Sí, así es, estos dos medios.

**E:  [NO ES INTELIGIBLE LO QUE DICE EL ENTREVISTADOR]...**

**e:**En realidad, bueno, yo he trabajado más con adultos y con el servicio de adicciones, entonces, para mí, yo no he tenido ese inconveniente, pero sí cuando teníamos estas reuniones de equipo, compañeros a veces mencionaban eso, ¿no? De, por ejemplo, los usuarios que eran derivados, los casos judicializados o los niños a veces no querían aprender, ¿no? Entonces, ellos sí habían encontrado estas dificultades, si bien es cierto, por lo que escuchaba, les daba como que la primera vez la oportunidad de repente de no aprenderla, pero en una siguiente sí, o hacer aproximaciones sucesivas. Quizá en la siguiente sesión, no directo, pero sí de repente un poco mirando hacia el techo, pero que pueda hacer un poco a poco el acercamiento hacia ellos.

**E:**Ah, muy interesante, entonces, ¿cuáles son las formas o medios utilizados para hacer adicciones? ¿Cuál ha sido la forma en que se está utilizando más de bien?

**e:**Para mí han sido el Zoom, Meet o Teams, no cualquiera de estas, por lo que mencionabas, ¿no? Que puede permitir también el compartir información, que es importante, y la que menos me gustó, de hecho, que ha sido la llamada a teléfono.

**E:**¿Consideras que hay que hacer esta forma, o si quieres, las tramas como diferente, o como usted va a acercarse, incluir en la manera de la comunicación, el tiempo que se utiliza, o incluso en la adherencia a continuar con las adicciones?

**e:**No considero que incluya en la adherencia, pero sí de repente en el tema del rapport. Lo que pasa es que en el caso de los comunitarios, somos centros de referencia, ¿no Entonces, ya vi un primer contacto que es con enfermería. Es muy difícil que psicología tenga este primer encuentro, entonces, ya es como que enfermería quien se lleva, digamos, todo el trabajo de poder hacer que el usuario quiera atenderse en un comunitario. Pero sí considero que influye, ¿no? Porque a veces el hecho de no ver a alguien, la confianza no es tan sencilla, ¿no? Incluso uno puede conocer viendo a la persona, pero la información que le brinda considero que al vernos es más fácil que se pueda dar.

**E:**¿Qué es lo que tiene comunicación y atención? Yo creo que el tema también en su caso sería la violencia, tal vez a nivel nacional y comunista, e incluyo justamente para hacer mayor distinción y para que los usuarios no se atiendan, visten también a los usuarios de la gente que tenemos con nosotros.

**e:**Bueno, desde MINSA hubo lo que es la publicidad en redes sociales, desde su página, hubo otros también, el tema en la televisión que salió, poder discundir a los mentales comunitarios. Desde DIRESA se hicieron volantes o banners, ¿no? Que se colocaban en los establecimientos informando que había la atención virtual. Eso ayudó. Si bien, en cierto, una de las dificultades de los comunitarios de callao es que no contamos con redes sociales propias, pero se hizo como que un flyer interno y le mandábamos a los usuarios. Ahora, si bien es cierto, ayudó mucho, pero recordar que a veces el móvil era del mismo profesional, lo cual también a veces generaba un poquito de incomodidad porque ya no había un horario fijo a veces, ya que el usuario te podía llamar a cualquier hora y todo ello. Lo cual obviamente se logra educando al usuario, poniendo límites, pero sí también generó por ahí a veces algún malestar entre los profesionales.

**E:**¿Y en el caso de las llamadas?

**e:**Ah, si es de llamadas o las videollamadas. Cuando obviamente Zoom era mucho más fácil porque solo quedaba el enlace, ¿no? Que lo podíamos enviar.

**E:**Esto me lleva a otra pregunta. Entonces, bueno, entiendo que el sistema de salud actual, política, laboral, está construido en partir de las normas que se impuesta, en función de la situación, y si en ese nivel de situación podemos tener un problema útil de la salud mental, ¿no es un problema, no? A veces no se lo pueden hacer ni en la salud mental.

**e:**Efectivamente. ¿En pandemia el ingreso, digamos, o el flujo no se respetaba, no? ¿Podía venir cualquier persona informando, por ejemplo, no? Yo tengo depresión, llevo mi tratamiento en la carrera, pero no hay citas, o no me dan información por pandemia, no hay nada. O sea, en pandemia no hubo este requisito de la hoja de referencia ni el filtro. Porque recordemos que muchos colegas con morbididad o porque también el hospital, por ejemplo, aquí en Carrion, todo era COVID. No había otro tipo de atención. No estaba brindando una atención en salud mental. Entonces, los hemos recibido, pero actualmente que ya no estamos con el tema de COVID, sí, pues todo usuario pasa este filtro en el centro de salud de primer nivel.

**E:**¿Y cuál es el proceso para obtener una hoja de referencia? ¿Cómo el paciente que no hace referencia lograba obtener una cita con el doctor para tener atención? ¿Cuál es esa línea de procesos?

**e:**Bueno, en pandemia con la hoja de referencia pasaba primero por acogida. Una vez que pasaba por acogida, lo derivaba de acuerdo, ¿no? Lo que encontraba en este caso la enfermera con especialidad en salud mental o para psiquiatría o para psicología. Ahora, en pandemia éramos en el mismo centro de seis horas. Entonces, las citas en psiquiatría eran lo que más rápido se llenaba y muchas veces pasaba por psicología, ¿no? Lo malo de la pandemia es que no nos permitía hacer este BAI que nosotros sí hacemos ahora en una modalidad regular. Entonces, respondiendo así, era acogida, enfermería y directamente con psicología en la mayoría de casos. Y nosotros coordinábamos con el médico psiquiatra o médico de familia si es que requería urgente la atención con medicina.

**E:**Es decir, la referencia no debía ser una cita de atención, y lo que hacía es que se utilizaba esa referencia que buscaba en la cita con el centro de salud.

**e:**Claro, ahora, sí o de lo contrario, llamaba al teléfono fijo del establecimiento o al móvil que se nos ha brindado, llamaban ahí, dejaban sus datos y Admisión programaba la cita con acogida, ¿no?

**E:**¿Y cómo se entregaba al paciente la cita? Es decir, ¿había un papel o un WhatsApp, mensaje de texto que indicaba a cualquier cliente que era el paciente?

**e:**Había el mensaje de texto o el WhatsApp, ¿no? Porque casi, casi el número de visores que tenían de manera presencial era poco. A ellos sí se les entregaba, en este caso, la fecha y la hora, y se les decía llamada o videollamada, ¿no? Pero no se les especificaba el nombre del profesional. O sea, les decían psicólogo, ¿no? No le daban el nombre y les pedían el del licenciado.

**E:**Ok, pero en esta llamada telefónica que usted está haciendo, ¿no se les solicitaba que le tocara en el proceso, por ejemplo, para hacer una llamada a un asunto? ¿Qué se le solicitaba en la llamada telefónica? No, han sido pocos los casos, pero...

**e:**Eso nos habia pasado mucho en admisiones. Esto era ir al establecimiento y ahi se le facilitaba una tablet y entonces se tenia el registro. Han sido unos casos.

**E:**O sea, el paciente en algunas ocasiones se presentó a un centro, pero la atención fue remota, porque el profesional no tenía la modalidad de ser remota.

**e:**Así es. Ahora, por necesidad de servicio, había, por ejemplo, enfermería y medicina venía a veces interdiario, ¿no? Y psicología, conforme pasaron los meses, ya veníamos una o dos veces por semana. Entonces, si identificábamos usuarios con bajos recursos, ¿no?, para poder tener esta llamada y todo ello, a ellos los priorizábamos y los atendíamos presencialmente con todas las medidas de bioseguridad.

**E:**Ok. ¿En la actualidad se continúa midiendo algún tipo de precaución en los establecimientos hospitalarios?

**e:**Sí, lo hacemos, pero con usuarios que de repente hemos identificado que venir hasta el establecimiento no lo pueden hacer por condiciones físicas o de repente también por el tema de que hay un diagnóstico ya crónico con una comorbilidad física, ¿no? Ahí los estamos atendiendo y también, como te había mencionado ayer por llamada, como tenemos un médico especialista, que es el médico psiquiatra, él está autorizado para hacer las atenciones, ¿no? En este caso, los centros de primer nivel evaluan, bueno, evalúan el caso y se lo derivan por una plataforma para la que él pueda hacer una evaluación, ¿no? Y si el médico determina que el usuario tiene una comorbilidad o tiene un diagnóstico moderado, vas a ser usuario del Comunitario de la Perla.

**E:**Claro, y ahora ya estamos en el punto de todo lo que hemos conversado, y en el futuro también, después de poder establecer ahora ya la función de telecomunicación, normalmente, en la directiva, etc., ¿cuáles son, si bien, especialmente, los principales bloqueos o dificultades para que se puedan implementar en la realidad? Eso quiero saberlo por comentarios.

**e:**Bueno, en mis establecimientos el tema del interés por los equipos tecnológicos, ¿no?Porque, por ejemplo, tenemos una laptop nada más, y las otras computadoras, si bien es cierto, tienen acceso a internet, pero no hay cámara, ¿no? Entonces, ahí se ven un poco las limitaciones, e incluso como nosotros estamos cerca a la playa, rápido el cable del uso de internet se para dañando y tienen que venir a hacer reparaciones, entonces, esa señal de internet tampoco es muy buena. A distinto, de repente, si tuviéramos USB, sería mucho más fácil para poder dar una atención, porque también es incómodo que se esté colgando en plena llamada o atención con usuarios.

**E:**Y en el mismo contexto, ¿cuáles serían las principales características que se facilitarían en el éxito de tener las atenciones?

**e:**Uno, el éxito de las atenciones, ¿no? Porque muchas veces en el tema de salud mental hay muchos estigmas y pedirle permiso a tu jefe para atenderte en psicología o psiquiatría a veces no es muy viable. En cambio, si fuera virtual, por último, no me ha pasado que a veces en hora de almuerzo el usuario me dice ya voy a ir a un lugar medio privado y te reúnes y tienes tu atención. Dos, un menor costo, ¿no? Porque también el trasladar implica un costo para el usuario. Tres, el poder también brindar una atención a los usuarios que te había mencionado que tienen dificultades para trasladarse, ¿no? Y también, como la mayoría de comunitarios estamos en zonas, de repente, un poco difíciles de acceso. Por ejemplo, aquí en la tarde usuarios a veces no quieren venir por el tema del riesgo, ¿no? Entonces, a ellos, por ejemplo, les podríamos atender de manera virtual y dar una atención y no correr el riesgo de perder un turno porque a veces programan pero no vienen. Entonces, sí, será mucho más beneficioso.

**E: [NO ES INTELIGIBLE LO QUE DICE EL ENTREVISTADOR]...**Y ahora ya, en cuanto a cuidados y supervivencia, que caracteristicas..

**E:**Bueno, el hecho de, quizá que los formatos puedan ser a nivel nacional, porque si bien es cierto, adicciones, servicio de niños y adolescentes, o adulto, adulto mayor, lo tienen todos, entonces, ya no es tanto lo que yo considero, lo que yo creo, sino lo que se tendría que hacer, como el formato GIS, que ya tiene una serie de áreas que se tiene que llenar.

Otra sugerencia podría ser también el hecho de poder implementar la parte de las brechas tecnológicas que tenemos, sobre todo los comunitarios de provincia, me imagino que más de repente que vaya. Otra sugerencia también podría ser el poder hacer la difusión, porque a veces no todos los usuarios o adultos mayores tienen acceso de repente a estar atendidos en un mental comunitario de manera virtual. Entonces, sería bueno hacer eso y también capacitar a los profesionales, porque lamentablemente a veces el índice de rotación es bastante, entonces, en algún momento se puede capacitar a alguien, pero ya luego no se da la información y las codificaciones, porque a veces no nos queda muy claro cuando, por ejemplo, no tenemos correo, teleatención, y como el personal que recibió la charla se fue, queda luego ese vacío como lo que hizo.

**E:**¿Cómo se considera una plataforma digital? ¿Cuáles son las principales limitantes y las que se favorecen? ¿se considera alguna plataforma digital tan extra que podría ser una solución? Y en qué punto de vista los principales limitantes y los que se favorecen, ¿a qué tipo de capacidad de vida se necesitan? ¿Sobre la página web, ¿no?, ¿sobre aplicativos?

**e:**Bueno, aquí la mayoría del personal es joven, ¿no? Entonces, maneja las plataformas y considero que se va a hacer fácil el dominar una plataforma. Pero si habláramos a veces de personas mayores, no es tan común, digamos, el manejo, ¿no?, de los herramientas, porque ahí sí, por ejemplo, tienen que consultar, hacer preguntas. Yo creo que ayudaría bastante, ¿no?, si es que es una plataforma, porque esta plataforma te va a dar todos los datos y poder también retroalimentar, ¿no? Por ejemplo, me quedan dos pendientes, entonces, yo puedo volver a llamar y todo ello. Si fuese una plataforma, genial. Incluso hay aplicativos que te permiten, es el mismo aplicativo, contactarte con el usuario sin tener tu correo o tu número personal, ¿no? Entonces, sí, ayudaría muchísimo para poder guardar esa atención.

**E:**Y entre esos características funcionales, ¿te gustaría que desde el mismo aplicativo puedan salir llamadas telefónicas?

**e:**Me parecería genial, tipo los call centers, que creo que es un poco... Claro. ...de las plataformas que manejan. Sí, considero que sería bastante útil también para el usuario. Ahora, recordar que los usuarios de salud mental a veces no son muy conscientes, no tienen conciencia de enfermedades, pero en este caso sería trabajo de nosotros o también de enfermería el poder ver, porque a veces el usuario, entre comillas, dice que está bien, pero sabemos que no porque está pre-contemplativo o no hay conciencia. Entonces, ahí sería el trabajo con el familiar, ¿no? Para que sea una red de soporte y que le diga, que trata de estar atento a esa plataforma.

**E:**¿Esa plataforma debería permitir no solamente tener datos de contacto con el paciente, sino con su alumno de referencia?

**e:**Definitivamente, porque en salud mental, por lo menos en comunitarios, hay algunos casos crónicos de esquizofrenia, psicosis, dependencia a drogas. Entonces, a veces ellos te dicen, no, yo estoy bien y el problema es mi esposa, mi mamá. Entonces, sí necesitaríamos también tener acceso a esa información.

**E:**Ok, ¿y qué otra característica podría ser importante? Tal vez dentro de tu proceso de psicoterapia o de la atención, necesitas que tus instrumentos sean de evaluación o de intervención. ¿Crees que sea posible adaptarlos a una plataforma social? ¿O es un tema que definitivamente no es posible que puedan adaptarse a la atención? Si te permite compartir pantalla, uno ya puede personalizar de repente la sesión.

**e:**Bueno, los test psicológicos, por ejemplo, en el caso de los comunitarios, utilizamos las pruebas psicométricas. Entonces, ¿de que se puede pasar un Excel y tener un link? Eso sí. En cuanto a la intervención, se tendría que ir adecuando y adaptándonos a los medios, porque, por ejemplo, si te permite compartir pantalla, uno ya puede personalizar de repente la sesión. O de lo contrario, si es que no te permite ya tener una línea base, para que el usuario vaya viendo cuáles serían los procedimientos. Entonces, sí sería importante que pueda tener acceso, de repente por lo menos a links, o a compartir una pantalla de manera general.

**E:**O que las actuaran. Por ejemplo, que desde la misma plataforma puedas activar una función para empezar a leer el diccionario, diciendo lo que está diciendo la plataforma. Y si no, no están en la misma plataforma y no tienen acceso a la pantalla.

**e:**Claro, eso sería genial.

**E:**Igual con las intervenciones, que son, de repente, no están luchando por el mismo protocolo que los usuarios. O sea, tareas, funciones, que tienen que seguir en un proceso de tiempo, pero que no pueden salir en contra de la misma plataforma. Y, por ejemplo, uno puede exportarlo para su impresión, y cuantos medios de historia, por ejemplo.

**e:**Sí, y también, de repente, descargarlo y de repente mandarle luego al usuario, por esta misma plataforma, para que tenga acceso y pueda recordar, ¿no? Cuál es el programa, cuál es la actividad y todo eso. Sería genial.

**E:**Y, bueno, generalmente las intervenciones frecuenciales no existen ni en ningún momento registramos en la historia de la comprobación de que existe una grabación. En el caso de que los anuncios consideramos que esto sería necesario que exista una grabación o área que se le pueda dar a la gente, ¿qué hacen? ¿Una participación de la gente?

**e:**Ahí sería un poco polémica la situación, porque cuando son usuarios que asistan de manera voluntaria, es difícil que te lo puedan dar. Pero en casos judicializados, por ejemplo, sí sería importante el consentimiento informado de ese instrumento. Porque a veces nos ha pasado de que tenemos que enviar la copia de la historia clínica a poder judicializar y el usuario pide su copia. Y luego, pero me han tomado cierta prueba que ha salido, yo no he dicho eso, entonces, si está su puño y letra de que él lo ha llenado, es obvio que ha sido él, pero sí nos quedaría de repente el vacío cuando es virtual. Entonces considero que sea un consentimiento informado por un link, quizá como que usted me ha mandado, y ya el usuario también se hace responsable de la información que estaría brindando.

**E:**Claro, sería imposible negarla porque todavía no se ha dado informe de una grabación, de la fecha en la que viene el funcionario, y el resultado que se va a publicar. ¿Cómo debería ser editado?

**e:**Claro, no es para los casos judicializados, porque ahí los otros usuarios no son mucho, es tener inconveniente con ellos.

**E:**Bueno, muchas gracias licenciada —-----, esta era mi última pregunta, realmente ha sido muy apreciable conocer su experiencia, su experiencia en la comunidad y cómo es que se hace la judicialización. Yo creo que en un caso del que estamos hablando, es una de las cosas más críticas y crónicas en la ciudad, y que tiene un proceso que no es capaz de ser analizado de cómo conseguir la situación. Es un poco reconocer qué criterios está cumpliendo el paciente, como por ejemplo, la gestión de salud, como actividades, y la discriminación que algún trabajador esté ya en el momento en que está.

**e:**Gracias, gracias a ti también por la oportunidad, y cualquier cosa, estamos en contacto.

**D5: Trabajador_Profesional_Salud**

**E:**Ok, entonces, buenas tardes —----, mi nombre es Juan Ambrosio, voy a hacerte ya la entrevista relacionada al estudio, en el que ya te hemos comentado un poco el consentimiento, te hemos comentado de líneas generales de qué trata, entonces básicamente es eso. Entonces para comenzar quisiera preguntarte más o menos desde cuándo has estado brindando tú ese tipo de servicios de la atención?

**e:**Bueno, la experiencia que tengo es el tema de a inicios de la pandemia, cuando se hacían llamadas a los trabajadores de salud que estaban aprendiendo el tema del COVID, específicamente la gente que trabajaba en el INS en salud pública, en los laboratorios.

**E:**Entonces a partir más o menos de 2020?

**e:**Sí, ha sido abril, específicamente la fecha no la recuerdo.

**E:**Ok, entonces a partir de más o menos iniciada la pandemia, decretaron el tema de la nadie puede salir y ahí es donde de alguna manera se te dio la oportunidad, esta situación en donde empezaste a utilizar estos servicios de salud mental a distancia.

**e:**Sí.

**E:**Ok, desde allí hasta la actualidad imagino que has seguido brindando este servicio a distancia también, o ya no?

**e:**No, fue en esa época.

**E:**Ese primer año?

**e:**Sí.

**E:**Ok, más o menos cómo brindabas este servicio? Recuerdas?

**e:**Bueno, nosotros teníamos el área de personal, nos daban los números de teléfonos de los trabajadores que estaban en presencial, que estaban trabajando y distribuíamos a los psicólogos, a quienes tenían que llamar y brindar soporte, obviamente previa, no sé si diagnóstico, pero saber cómo estaban en ese momento, cómo se sentían y de acuerdo a eso también establecer una serie de consejerías.

**E:**Entonces tenían un primer contacto que era sólo por teléfono?

**e:**Sí, sólo por teléfono, las intervenciones han sido solamente teléfonos.

**E:**Ok, sólo teléfono, no ha habido digamos como uso de plataforma Meet, Zoom, eso que quizá más adelante se empezó a usar?

**e:**No me acuerdo.

**E:**Al menos en tu experiencia no hubo eso?

**e:**No, yo recuerdo que era telefónica.

**E:**Ajá, y cuando empezaste a iniciar o en el tiempo que estuviste utilizando el teléfono para vender atención, de alguna manera contaron con un protocolo o algo estandarizado que de alguna manera establecía así se debía hacer las cosas?

**e:**Sí, armamos una especie de esquema de qué consistía el contactar, presentarnos, enseñarles por qué los estábamos llamando, qué requerían y de acuerdo a ese requerimiento, dependiendo de cómo encontrábamos a las personas, era llamarlos cada ciertos días. Y eso básicamente. Y dentro de estas llamadas que hacíamos, además de preguntarles cómo se sentían, también preguntábamos cómo estaban trabajando, si tenían las condiciones adecuadas.

**E:**Ok, entiendo que hablas de que ustedes, a quiénes se refieren ustedes que establecieron el protocolo?

**e:**Ah no, el equipo de psicología.

**E:**El equipo propiamente? No hubo algo así como una guía directiva, digamos, de más arriba? Ustedes mismos organizaron?

**e:**Sí, fue por iniciativa también con la dirección ejecutiva, que en ese momento estaba yo, si mal no recuerdo. Y él nos sugirió, nos propuso hacer esas intervenciones y acordamos con los psicólogos de ese entonces, porque éramos menos, y empezamos a llamar, teníamos un horario, teníamos un número de números a quienes convocar. Y también obviamente coordinábamos con los jefes de esas áreas para que nos proporcionen información de sus trabajadores.

**E:**O sea, había cierta facilidad de arriba, de la dirección ejecutiva, más no había algo, digamos, protocolizado de parte de ellos, sino que ustedes tenían esa libertad, la necesidad de armar algo más estructurado para la parte operativa interna de ustedes.

**e:**Sí, como para tener un orden, para que todos hagamos lo mismo.

**E:**Ok, y dentro de esa estructura, más o menos entiendo que estos pasos que tú mencionas son como que identificando, otra vez estas facilidades, ir viendo qué requería cada personal y luego irlos atendiendo en términos de usar el teléfono y llamarlos. ¿Había algún otro paso más que considerabas que hubiese sido necesario, que se tenga en consideración?

**e:**En realidad, en los casos que hemos tenido hubiese sido ideal continuar con este proceso.

**E:**¿Seguimiento te refieres?

**e:**Sí, porque hay unos trabajadores que emocionalmente sí estaban, no muy mal, pero sí, de repente no por enfermedad de ellos, sino por familiares, pero no logramos continuar.

**E:**¿Qué ocurrió? ¿Por qué es que no se logró continuar?

**e:**Al inicio estábamos solamente los psicólogos del CENSOPAS y después, no recuerdo bien qué ocurrió, pero creo que la demanda fue muy grande porque no solamente trabajábamos con los trabajadores, sino también con familiares.

**E:**¿De los trabajadores?

**e:**De los trabajadores. Entonces, también se les daba soporte, pero obviamente nuestro objetivo principal eran los trabajadores. Luego, ya habíamos conversado con todas las personas que estaban trabajando en ese entonces. Muchos de ellos también nos decían, no, si estamos bien ya no es necesario, o mira, te voy a llamar a tal fecha, ¿está bien? Bueno, si quieres. Como que para algunos sí fue importante y para otros, de repente, quizás interrumpíamos su actividad. Pero ojo, llevábamos en horarios distintos, podría ser en la mañana, en la tarde, el primer contacto. Y después, en qué momento ellos tenían mayor disponibilidad y nos daban la hora y la fecha.

**E:**Entonces, entiendo que había falta de personal para cubrir.

**e:**Sí, eran muchos.

**E:**Ok, eran muchos para los psicólogos que había. Porque además, entiendo que había, por un lado, familiares de estos profesionales trabajadores que más bien ellos querían. Pero por otro lado, había otro tipo de trabajadores que más bien, un poco reticentes, renuentes a ser atendidos.

**e:**O sea, sí, no lo tomaba mal, pero creo que es un tema de confianza. Es decir, mi empleador, porque nos presentábamos como INS, como CENSOPAS. Entonces, de repente, no tenían tanta confianza como para abrirse completamente.

**E:**Claro, es como que esto viene de mi empleador y tener cierto recato porque viene de mi empleador.

**e:**Claro que también, no ha habido ningún filtro de información y tampoco... Porque también ha sido una oportunidad para el trabajador de sus quejas, por ejemplo. Si no tenía guantes, no tenía EPS. Y obviamente también nosotros hemos canalizado un poco los requerimientos que había. En el sentido de la percepción que tenían los trabajadores. No, obviamente, como grupo.

**E:**Claro, o sea, podríamos decir que de alguna manera lo que hacía que también atiendan la llamada era un poco su descargo. O sea, no era solamente atender lo que convencionalmente se ve, digamos, que tienes algún problema de salud mental. Sino también, de alguna manera, generaba este descargo, una especie de centro de reclamos.

**e:**Porque estaba relacionado, o sea, su malestar estaba relacionado con las condiciones con las cuales estaban trabajando.

**E:**Ok. ¿Has tenido algún caso difícil? De repente que tú consideres que ha sido difícil de manejar. Sea por circunstancias de la persona o circunstancias que puedan haber sido contextuales.

**e:**Debe ser, pero sinceramente no recuerdo bien.

**E:**No recuerdas bien. ¿Alguna vez tuviste algún problema de repente con el teléfono, la señal? ¿Algún paciente de repente que no se escuchaba bien, se te recortaba?

**e:**No, no. Lo que sí era frecuente que quedabas en una hora, los llamabas y no te contestaban. Y luego, después de un rato, volvías a llamar y a veces te contestaban o a veces ellos te devolvían la llamada. Pero decían que el celular lo habían dejado en su locker. ¿Sabes? Porque al laboratorio no entraban con equipos. Entonces era por esas cosas. De repente se demoró procesando y ya no pudo salir.

**E:**¿Y había, digamos, como que cierta facilidad de parte del empleador, en este caso del INS, para darles facilidades para atender el teléfono durante la jornada laboral?

**e:**Sí tenían facilidades. Porque también yo creo que para el INS, para los gestores de ese entonces, como que les veía, les hacía bien a su imagen de que se estaban preocupando por sus trabajadores. Entonces sí había facilidades. El tema era de que, por ejemplo, en zonas en el laboratorio, por ejemplo, donde tenían que procesar, por más de que tengan la libertad, no podían llevar el teléfono. Porque tenían, no sé, sus procesos tenían un tiempo y tenían que estar en un lugar, pues, no sé, como que con más protección. Y hasta que se pongan y eso se demoraba, entonces obviamente tenían que priorizar el procesar las muestras. Porque la demanda y la carga de trabajo era muy alta. Trabajaban por turnos.

**E:**Claro, también la forma de trabajo, la estructura de trabajo... Y entiendo que su, digamos, el horario donde atendías con el equipo de psicología, era en el horario laboral.

**e:**No necesariamente. No necesariamente. Era en el horario que ellos podían. A veces podíamos contactar con las personas cuando se estaban trasladando hacia... o los consiguieron un lugar donde descansar en la villa panamericana. No recuerdo. Entonces mientras los llevaba, pronto decían ya llámeme a las 6, o llámame a las 7, o llámame a las 8. A veces la gente decía, llámame a las 10 de la noche. No teníamos un horario... O sea, lo ideal, claro, era acomodarnos un horario de trabajo. Pero dependíamos del horario que ellos tenían.

**E:**Y eso, ¿podrías decir que fue lo que más te disgustó? ¿O lo que de repente considerabas que era adecuado? ¿Te gustó o no te gustó eso?

**e:**La verdad no recuerdo bien, pero no creo que haya sido de molestia o algo. Al contrario, yo creo que era también sentirnos bien de estar colaborando con esas personas.

**E:**¿Podrías decir entonces que esa facilidad de comunicarte a cualquier hora cuando lo necesite?

**e:**Claro, nosotros estábamos en nuestras casas. No había ningún problema. Ellos sí eran los que tenían que salir y todo lo demás. Entonces no había mucha incomodidad. Claro, obviamente el ideal hubiese sido el horario de trabajo. Pero eso no se podía. O sea, perdías el rastro cuando tenías que acomodar.

**E:**Pero por tu caso no habría problema. Estaba como bien, no habría problema. ¿Qué podrías decir que fue lo que más, entonces, en ese sentido, te agradó o te gustó de esta nueva forma de atender? A distancia.

**e:**Yo creo que fue bueno porque... Las personas podían encontrar cierto soporte en las condiciones en las cuales estaban. Que no necesariamente estaba asociado tener que ir hasta un consultorio o tener que ir hasta un hospital, etc. Para poder recibir el soporte. Yo creo que en ese tema sí fue muy muy importante.

**E:**Claro, podríamos decir que esta parte de tener un soporte en cualquier momento, esa facilidad de conseguirlo, como que fue lo que más te agradó.

**e:**Sí. Por ejemplo, una vez un señor me llamó en la noche. En la noche no estaba planificado, mandó un mensaje, no sé si me podía llamar. No sé qué había pasado, no recuerdo bien si había tenido un problema de algo. Pero estaba llorando. Entonces como que necesitaba conversar con alguien. Y eso, que él tenga la confianza de que en el momento que requiera, porque siempre le decíamos eso, si usted en un momento necesita hablar con nosotros, llámenos. Entonces yo creo que eso era importante para ellos. Como que me están dando el soporte. No me pagan bien, no tengo buenas condiciones, pero al menos me están dando el soporte. Hay alguien ahí, al menos.

**E:**Ok, eso fue lo que más te gustó y te agradó.

**e:**Ok, qué fue quizá lo que agradó a todo el equipo.

**E:**Ajá, ok. A todo el equipo. Y qué fue lo que personalmente te desagradó más de repente esta nueva forma de atender. ¿Me desagradó? Sí. De repente no te parecía, no estabas tan de acuerdo. La verdad. Esta forma de trabajo, digamos, de atención, remota.

**e:**Yo creo que no había mucho que me desagradaba. Sí la preocupación del tema de estar obviamente como que disponible, y también el hecho de, no sé, que de repente llamabas y no contestabas, no contestabas y era... A veces se cruzaban y nos quedaban el mismo horario para varias personas. Entonces era... Ahí era un poco complicado, creo yo. Pero después no recuerdo, mira, han pasado tantos días.

**E:**Claro, o sea, la estructura de cómo ejecutar es lo que quizá, al no haber, digamos, un cronograma claro o una estrategia que evite, digamos, este cruzamiento, quizá eso fue lo que más te...

**e:**Pero no era por nosotros, era por ellos. Ok. Porque nosotros sí habíamos separado horarios y también los números. O sea, yo no podía llamar a los que te asigné. Y teníamos cada quien creo que manejaba sus propios horarios. Entonces el tema era de que si tú tenías que convocar a tres personas ese día, y esas tres personas no podían en el horario que tú habías planificado para ellos y te daban el mismo horario. Entonces tampoco les podía decir, mira, te paso por otro día. Entonces para no perder esas, tenías como que ajustarte en la llamada con alguno de ellos para poder cubrir, o sea, terminar de llamar a todos.

**E:**Y podría ser un poco difícil ese ajuste.

**e:**Claro, porque a veces la persona te está esperando a que conversara contigo. Claro, entonces a pesar de haber... No, entonces tú estabas así como...

**E:**Claro, a pesar de haber esa disponibilidad, de todas formas había eso, esa, digamos, dificultad, a pesar de tener, digamos, el teléfono, algo que te pueda acercar y hacer más disponible el comunicarte, esta dificultad que había, al menos con el teléfono, que me entiendas, que se cruzaba, se podían cruzar, ¿no? Lo llamabas y la otra persona, de repente, te devolvía la llamada, y se cruzaban las llamadas y no se concretaba a veces la comunicación.

**e:**Sí, pero, por ejemplo, tú me dijiste que íbamos a conversar a las 9 de la mañana y habíamos quedado a esa hora y te llamo a las 9 y no contestas. Y yo, y de pronto tú me mandas un mensaje y me dices, no puedo a las 9, por favor a las 10 de la mañana. Y tú ya habías quedado con otra persona a las 10 de la mañana.

**E:**Ah, ok.

**e:**Entonces, este, y es el único horario que puede. Entonces, al de las 10 lo recibes, pero ya no de repente todo el tiempo que habías pensado, sino como que lo recortas un poco para no perder al otro, ¿no? Algo así.

**E:**Claro. ¿Y los trabajadores de algún momento llegaron a referir qué es lo que más les gustó? De esta forma de trabajo.

**e:**No recuerdo.

**E:**¿O lo que más de repente les gustó de esta nueva forma? ¿Crees que coinciden con lo de ustedes?

**e:**Yo creo que... Yo creo que, no sé, habrá un grupo de trabajadores que les habrá molestado, ¿no? O sea, como que, ya no, o sea, yo estoy bien, porque habían unos que eran muy importantes. Sí, todo estoy bien, sí, no se preocupe, gracias, ¿no? No se preocupe, le debo la llamada. No, no, yo le llamo, no, no nos llamen otros miembros. Pero también había otras personas que sí, que esperaban la llamada, ¿no? Y después, cuando ya dejamos de ver eso, porque ya la UCI se hizo cargo. Ajá. Ah, por eso dejamos de ver, porque ya la uso se hizo cargo. Entonces, a veces te mandaba mensajes, ¿no? ¿Qué tal? ¿Cómo estás? Oye, te cuento lo que te conté, mira, ya eso se ha mejorado, cosas así.

**E:**Claro, le daba una especie de actualización después de...

**e:**Pero ya no podíamos seguir, porque ya la uso. Estaban sumiéndolo, ok, ok, entiendo.

**E:**¿Recuerdas también cuando ha atendido pacientes de forma presencial? ¿Ha dado atenciones?

**e:**¿De ese que viene ese contexto?

**E:**No. No, no en ese contexto, pero sí cuando ha tenido atenciones presenciales.

**e:**¿Como clínica o ocupacional?

**E:**Como clínica, una atención, ¿no?

**e:**Ya.

**E:**Ok, ¿de alguna manera podría comparar entre esta forma de atender remota, por teléfono, con la forma de atender en persona, no? ¿Podría de repente comentar cuáles son sus impresiones? ¿Hay diferencias, no hay diferencias?

**e:**Hay que entender de que lo que hacíamos era apoyo psicológico. Claro. No era evaluación o terapia, ¿no? Entonces, no podemos comparar. Pero en cuanto a la atención en sí, yo creo que desde el punto de vista del teléfono, te cuesta un poco más establecer el rapport con la persona. Pero desde el punto de vista práctico, yo creo que es mejor porque puedes coberturar a más personas. Porque puedes estar hablando con personas que viven muy lejos, que económicamente no va a ser factible que vengan hasta donde tú estás. Entonces, yo creo que esa es la principal ventaja.

**E:**Claro, claro. Está cobertura, pero el tema del rapport puede ser un poco más limitado.

**e:**Sí, pero yo creo que con la práctica, yo creo que esas cosas se pueden solucionar.

**E:**¿Podría decir que a partir de esto, en adelante, cuando se trabaja en atenciones remotas, una sugerencia importante para usted sería el tema de capacitar en cómo generar rapport en este tipo de atenciones remotas?

**e:**Yo creo que sí. Como parte del proceso de intervención. Sí, de repente, porque para la persona, bueno, es que va a depender. Hay gente que te va a buscar porque quiere, necesita. Y como que está más abierto, va a contribuir más rápido. Y va a haber gente en la cual necesita, pero de repente tiene mucha desconfianza. A ver, ¿con quién está hablando? No te conozco, no te veo, no sé cómo eres, cómo me vas a tratar. Entonces, el tema de modular la voz también, porque yo creo que esos aspectos sí son importantes para que la gente te diga, oye, sí, me pareció amable o me pareció, no sé, muy importante o muy directo.

**E:**Ok, entonces dentro de este ámbito, sería bueno que quienes vayan a dar ese tipo de atención estén capacitados en el establecimiento del rapport, que es lo que tiene que ver mucho con el aspecto verbal, de cómo hablar, el tono, las palabras, las pausas.

**E:**¿Habría alguna otra cosa a tener en cuenta más adelante, en futuras atenciones?

**e:**No sé, no se me ocurra más que eso.

**E:**Podría mencionar alguna otra capacitación que haya querido que le brinden en el uso del teléfono o generar ese rapport

**e:**Yo creo que por ser psicólogos estamos preparados para todo, pero hay algunas cosas que necesitamos pulir eso si es importante. Cómo conectar con la gente, que la persona no sienta que se está haciendo por obligación o porque ya ve como te va, sino porque hay un interés genuino cómo te van a recibir las personas. Ahora que recuerdo, nosotros mandamos correos informativos para dejar un poco la desconfianza y para que nos contesten y para que por ahí si no podemos llenar alguno, una persona pueda comunicarse con nosotros. De alguna manera institucionalizando la actividad

**E:**¿Habría alguna recomendación más?

**e:**No

**D6: Trabajador_Profesional_Salud**

**E:** Buenos días, estamos 22 de junio de 2023, estamos con —------, psicóloga que ha brindado servicios de telesalud en el contexto de pandemia por COVID-19. Te pediría, —-----, por favor, podías presentarte, tu nombre, profesión y cargo actual.

**e:**Buenos días, mi nombre es —-------, soy psicóloga y trabajo para un centro de salud mental comunitario, se llama 12 de noviembre, que queda en San Juan de Miraflores.

**E:** Muchísimas gracias, estás justo en nuestro grupo objetivo. Bueno, la primera pregunta de las siete que tenemos, cuéntame brevemente, desde hace cuánto tiempo has brindado servicios de telesalud o psicología a través de medios digitales y cómo ha sido tu experiencia general brindando estos servicios.

**e:**Empecé en 2021, diciembre de 2021, bueno, la experiencia no fue muy, no nos ayudaba mucho, en realidad no nos ayudaba mucho cuando era teleconsulta o por, ¿cómo le llamaban esto? virtual, de manera virtual, porque en el distrito donde se encuentra el centro de salud, la gente no tiene mucha accesibilidad a las redes, al internet, o tener un internet que pueda abastecer una videollamada por whatsapp, menos por Zoom, muchos solamente se pueden comunicar por su celular y las veces que conectábamos, llegábamos a conectar, pues, había mucha interferencia, se colgaba, se quedaba congelado, y terminábamos mayormente haciendo por solamente llamada telefónica. Muchas veces empezábamos para poder ver al paciente, pero se terminaba haciendo solamente llamada telefónica.

**E:** Ok, y para, digamos, estas situaciones en las que tú mantenías que tomar decisiones de por qué medio utilizar la llamada, cómo iba a ser el medio de abordaje, ¿existía o contabas con alguna guía, protocolo, manual, sea de tu centro de salud mental comunitario, tu DIRIS o MINSA?

**e:**Como una guía, bueno, estaba teniendo que, no, no había una guía, nosotros abordábamos como una sesión, no, como una sesión de manera presencial, pero no teníamos un manual en realidad.

**E:** Ok,¿recibieron algún tipo de capacitación para, digamos, tener algunas consideraciones o qué métodos debían utilizar en teletensión?

**e:**No.

**E:** Y entonces, en esta situación, digamos, que no se contaba con un documento, guía, protocolo como tal, ¿qué condiciones o características, qué contenidos debería tener una guía de telesalud en base a tu experiencia? Más que todo dirigido al psicólogo, ¿no? O sea, al que va a hacer la...

**e:**Nosotros vamos a dirigir. ¿Qué contenido debe tener? Bueno, creo que establecer este, de repente, primero con unas normas o reglas, poder explicarle a la otra persona cómo se va a realizar, ¿no? En cuanto a horarios, pero nosotros, para que las reglas sean para nosotros. Bueno, yo no tuve mayor, este, molestia, no, yo tenía que entender que, por ejemplo, en ese lugar no íbamos a poder tener mayor confianza con una videollamada, menos por Zoom, y pues yo me acomodaba, ¿no? A lo que, a cómo se podía, pero... ¿Cómo podría brindar? No sé, la verdad que me hace raro.

**E:** Es decir, ¿tuviste alguna situación en la que, digamos, sobre cómo actuar tenías alguna duda y te hubiera gustado tener algún manual de consulta que te diga cómo solucionarlo?

**e:**Bueno, este, sí, mira, sí es bueno siempre, ¿no? Tener que contar con un manual, saber qué paso, seguir, pero en realidad, este, también creo que cada profesional puede, ¿no? Ahí lo, como se hizo, ¿no? Cada profesional tomó ya en base a... Sus experiencias en atención presencial. A cómo manejar, experiencias al manejo, o sea, sí, en realidad es, también depende, ¿no? La experiencia, de repente alguien más joven no hubiera sabido qué hacer, es decir, de repente, creo yo que de repente, ahorita que me pongo a pensar, es, por ejemplo, se realiza, este, va a ser, si una, por vía Zoom, ¿no? Entonces, puede ser que si no haya una regla, unas normas que digan, este, en caso no se pueda llegar a contactar vía Zoom porque pasar a otro intento, que segundo punto sería pues por WhatsApp, ¿no? Y bien, si en caso no se puede, si en caso tampoco usted llega a revisar, no se puede WhatsApp, entonces, o sea, siempre agotar hasta el último que se llegue a realizar al menos el contacto telefónico con el paciente, ¿no? Porque puede ser que haya psicólogos profesionales que digan, bueno, no tenía internet, y ya no, no, este, ya no se llegaron a contactar, porque si eso ha pasado, yo escuché a muchos pacientes que me decían, no señorita, ya nunca, al menos en la época de la pandemia, perdieron todo contacto, ¿no? Porque ellos, ¿qué ponían en la historia? Este, pues, este, no nos llegó a contactar, no contestaron, entonces, hubo ya, si perdieron muchos pacientes, perdieron el contacto de poder seguir, continuar con sus sesiones psicológicas, ¿no?

**E:** Pero fue justamente utilizando esta última modalidad, que es la de teléfono, llamar por teléfono.

**e:** Ajá, sí.

**E:** Me comentabas que habías utilizado Zoom, WhatsApp y llamadas, ¿has utilizado otra plataforma?

**e:**No, yo por ejemplo, solamente lo que tenía en el celular, porque nosotros no teníamos computador ahí.

**E:** Ok, en el caso de Zoom, ¿siempre solicitabas que hubiera, digamos, una pantalla prendida, la cámara prendida por parte del evaluador?

**e:**Ah, sí, sí, sí.

**E:** ¿Cuando era por WhatsApp, siempre eran videollamadas, o podía ser incluso por chat?

**e:**Primero, como yo te digo, por un lado, era videollamada, pero cuando se quedaba congelado, o daba vueltas, no podía tener, entonces ya no se realizaba la videollamada, y yo llamaba. Pero por chat, por WhatsApp, conversaciones también, más que nada, sería como para acordar nada más, ¿no? Tal día lo volvemos a realizar, porque a veces el otro lado de los pacientes estaba en otro lugar, o estaba mi señorita, tal día mejor acordamos, y para que esté en un lugar donde tenga mejor señal, ¿no? Nada más era por chat.

**E:** En el caso de estas tres plataformas que has utilizado, ¿cuál es la que más te ha gustado y por qué?

**e:**¿En el caso de las sesiones?

**E:** No, de las tres plataformas, Zoom, WhatsApp por telellamadas, videollamadas o teléfono.

**e:**¿Cuál es la que más te gustó?

**E:** Sí, pero en el caso de solamente abordarlo con los pacientes, en ese caso nada más, ¿cierto?

**e:**La que más me gustó para poder, creo que con la que, a mí me gustaría más la de Zoom, pero como no se lograba obtener una buena señal, la que más me funcionó a mí fue la de teléfono.

**E:** Pero paradójicamente también es la que más te disgustaba.

**e:**Claro, porque no la podía ver. Sí, nada la podía ver y no podía, porque muchas veces eran niños, por ejemplo, ¿no? Con los que papás contestaban y a veces no los podía ver a ellos, porque el contacto visual también es difícil.

**E:** Claro, qué complicado, ¿no? Con niños por teléfono.

**e:**Claro, era ya, a veces, sí, sí, en esa época fue muy complicado, en la época de pandemia.

**E:** Y ahora, en lo que respecta, en lo que me mencionas, ¿cómo te ha parecido la interacción de los pacientes utilizando plataformas de telesalud? ¿Crees que utilizar Zoom, videollamadas por WhatsApp, teléfono, ha influido en la adherencia del paciente directamente, en los distintos grupos de pacientes que hayas tenido?

**e:**¿Ha influido de manera positiva?

**E:** No, de alguna forma, ¿negativa o positiva en la adherencia, en que ellos continúen, en que acepten participar?

**e:**Ah, no, no mucho, ellos sí, en su mayoría prefieren que sea de manera presencial. Cuando se les ha avisado de que por algún motivo, también, por algún otro motivo que se iba a realizar la sesión de manera virtual, ellos no han rechazado. Prefieren ellos que sea de manera presencial.

**E:** ¿Actualmente continúan brindando servicios por telesalud?

**e:**No, yo, a menos, yo ya no.

**E:** No, pero en tu centro, digamos, ¿tienes como política todavía la oportunidad de sacar citas como telesalud?

**e:**No, a ver, yo creo que ya no, solo, no, ya no.

**E:** ¿Qué estrategias de comunicación y difusión pudiste observar o conociste que el sistema de salud realizaba para promover las teleconsultas? ¿Para promover las teleconsultas?

**e:**Me van a botar.

**E:** ¿Qué estrategia? No. Es anónimo, no te preocupes.

**e:** Ah, ya. Este, no, no vi ninguna.

**E:** Ah, ya, ok. No hay problema, esto es válido. ¿Cuáles son las principales dificultades? O sea, me has contado algunas, ¿no? Pero ya ahondando en esta pregunta en específico, si es que tuvieras alguna más. ¿Cuáles consideras que han sido las principales dificultades al momento de brindar telesalud?

**e:** Las dificultades, ah, ya. Que los pacientes no tienen una buena red, ¿no? ¿Cómo lo llaman? La red de internet. No cuentan con los medios materiales, ¿no? Una laptop o una PC. No tienen ellos, ¿no? Los medios para poder ellos recibir una, digamos, una atención de manera virtual. Son muy pocos, casi nadie. Y esa de que no tienen, no cuentan con los medios, con los aparatos necesarios. No tienen una computadora, una laptop. Y los celulares que tienen, con los cuales ellos podrían realmente recibir, mayormente no tienen pues este, el internet de una banda. Sus datos, ¿no? Como dicen los datos. Ellos colocan, creo que le pagan este, ¿cómo se llama?

**E:** ¿Prepago?

**e:** Sí, eso, prepago. Sí, entonces eso se les va rapidísimo. A ellos no les conviene por eso. Eso, y después cuando son mayores que no saben, no saben pues este, manejarlo, ¿no? Por ejemplo, si es una persona mayor y tiene que haber alguien a su lado para que le ayude a conectarse. Muchas veces no hay esas personas porque las personas pues están trabajando y a veces son personas mayores o niños. Y en caso de personas mayores y niños, tiene que haber alguien que les ayude, ¿no? Ese es otro inconveniente.

**E:** Y del otro lado, digamos, facilitadores o situaciones, factores que han beneficiado el uso de TeleSalud.

**e:** Por parte de nosotros, soy de TeleSalud. O sea, que le faciliten a ellos, a nosotros, que nos faciliten a nosotros.

**E:** En general, en general, ¿ustedes, el usuario, al proceso de atención?

**e:** No. Ok. Mira, por ejemplo, perdóname que te corte. Sí, sí. Yo pertenecí, yo cuando ingresé, pertenecía a un programa que se llamaba PAPSI. No sé si lo escuchaste. Entonces, era como nuevo, ¿no? Entonces, no teníamos ni siquiera un lugar establecido en el centro. Estábamos en un auditorio, luego ya nos pudieron acomodar y tuvimos un lugar. Y todo, como te digo, era por nuestros medios, ¿no? Yo usaba mi internet, por ejemplo, ¿no? Yo usaba mi internet, yo usaba mi teléfono, las llamadas también eran, todo era por mis medios. Porque no había una, o sea, había una sola laptop, pero es utilizada, pues, ¿no? Por muchas compañeras. No teníamos, este no. Y el internet en el segundo piso donde estaba, en que yo estaba ubicada, el wifi y esto no, tampoco tenía. Yo muchas veces me compartía de mi internet, de mi teléfono, comparto a la computadora.

**E:** Y digamos si, el fin de este estudio, justamente es, digamos, proponer una plataforma de telesalud que ayude a solucionar estos problemas, ¿no? Entonces, tal vez, de lo que he podido recoger con las entrevistas contigo, es que una característica principal debería ser de que tenga un consumo de internet o de datos muy bajo, ¿no? Para que pueda ser accesible a otras personas que tienen bandas muy bajas o que no tienen mucho, porque justamente hacen una recarga prepago y en plena consulta puede cortarse y se pierde la comunicación. Entonces, si esta plataforma, digamos, existiera, solucionar ese aspecto, ¿qué otros elementos adicionales debería tener esta plataforma para que faciliten tu trabajo? Puede ser desde un enfoque de necesidades que te gustaría que tenga la plataforma en el sentido de historia, de instrumentos u otros, ¿no?

**e:** Yo soy —------------ en tecnología, no, en historia, instrumentos, que me dices, no te entiendo bien.

**E:** Ah, ya, no, en historia me refiero a la historia clínica, psicológica.

**e:** Ah, ya, ya, hoy sería, hoy eso sería que nos facilitaría, eso sería genial, que cada historia estuviera ya en, digamos, en la computadora, eso sería una gran ayuda. Sí, claro, porque a veces demora mucho el tema de que, por ejemplo, hay que tener un paciente, yo no puedo empezar a tener un paciente si no me entregan la historia, que lo he hecho a veces por ya temas de tiempo, porque cada paciente tiene un horario, y si yo me paso ese horario y el otro empieza más tarde, más tarde y así, ¿no? Entonces, si no tengo la historia, a veces no recuerdo bien quién es, y en el tema administrativo, a veces hay problemitas, ¿no? Que se tenga una PC, bueno, no sé, una PC, un aparato que se escanean con el ambiente, pero sí, sería de mucho, mucho aporte, ¿no? Porque trabajar, seguir trabajando, a veces así, como se trabaja, ¿no? con todo, están las historias, no sé, de manera, ¿cómo te puedo decir?

**E:** Física.

**e:** Física, ya, no, trae muchas, muchas dificultades, y por ejemplo, a veces, como hay distintas áreas, esta historia está en otro lugar, en otra área, en otro consultorio, y ya no, dice, no la encuentro, entonces es todo un desorden, y si yo la tuviera ya, en cambio, la historia en la computadora, ya podría trabajar, ¿no? No estoy molestando a varias, a varios personal del centro.

**E:** Claro. Tú trabajas en un centro de salud mental comunitario, entiendo de que en la pandemia sí ha habido un acceso directo, es decir, el paciente podía ir directamente al centro de salud mental comunitario y sacar su cita, ¿no? O accedía a través, creo, de enfermería.

**e:** Los pacientes obtienen una cita cuando vienen con una hoja de referencia. Sí, del establecimiento más cercano de donde ellos pertenecen, les entregan una hoja de referencia, y con esa referencia llegan a mi centro, y de ahí ya pueden ser atendidos.

**E:** Claro, porque en tus centros se entiende de que la atención ya es especializada, no es de tamizaje, sino es justamente ya de intervención.

**e:** Sí, sí es especializado, sí, ¿no? Pero también pasan primero también por una enfermera.

**E:** Claro, claro. Entonces, digamos, pensando en hacer un adecuado tamizaje, ¿cuáles considerarías que deberían ser las características que tenga que tener una evaluación previa antes de que el paciente llegue a ti, como ya para una intervención especializada? Para estar seguros de que se trata de un tema de salud mental complejo y no de una situación más ambulatoria que pueda ser tratado en el centro de salud de primer nivel.

**e:**------—--, ahorita que me has hecho esa pregunta, no sé si me has hecho acordar de que sí hay tamizajes que se realizan vía telefónica. Ya, entonces creo que en esa área sí todavía es, como tú me preguntaste si todavía hay de manera virtual, pero lo hacen vía telefónica. Ya, eso sí, sí me acordé. Entonces no es que no totalmente se haya cerrado, pero sí, esos tamizajes, eso las enfermeras lo realizan vía telefónica.

**E:** Ok, y para ello ¿emplean algún cuestionario psicológico?

**e:** Sí, tienen un cuestionario, tienen un formato ellos.

**E:** Ah, ok.

**e:** De preguntas, ¿no? Como un cuestionario. Eso va en la historia clínica, ¿no? Y cuando ya llega a mis manos está todo ese cuestionario que ya han pasado.

**E:** ¿Ese cuestionario es un filtro? Es decir, digamos, si no respondiera a suficientes preguntas como para que el cuestionario te arroje un resultado de que sí necesita atención psicológica por... ¿No pasaría atención contigo? ¿Sería regresado a su centro?

**e:** No sé si ha habido esos casos que los hayan regresado, pero yo creo que sí, definitivamente sí es un filtro, pero no conozco algún caso... En este caso, cuando ya igual pasan con nosotros, ¿ya? Entonces, lo que yo he visto en mi experiencia es que igual pasan conmigo o con otro profesional y ahí nos damos cuenta de que no es necesario, de que no es necesario que sigan en el centro, que no requiere de una atención especializada, como tú lo has dicho. Y ahí se le hace la contrareferencia, creo, y vuelven. Eso sí lo...

**E:** Ok. Entiendo también, ya esa es la última pregunta. Sí, sí, es la última pregunta. Que ustedes no solamente atienden casos de salud mental propiamente dicho, es decir, una persona con una patología o un trastorno mental, sino que a veces llegan situaciones legales, ¿no? De embarazadas, judiciales, fiscalía. En esos casos, ¿consideras que también sería aplicable el uso de una plataforma de telesalud?

**E:** Sí, eso se llama el módulo VIR, ¿no? Es que ayuda mucho la plataforma, pero a veces yo creo que en esos casos que son más fuertes, ¿no? Donde el paciente requiere de mucha contención emocional, ¿no? De repente la primera sería a partir de la segunda sesión. Ok. La primera vez que tú recibas al paciente, se sienta, ¿no? Está acogido, ¿no? Se sienta acogido, que está en contacto más personal. Y luego ya a partir de eso podría hacerse ya, ¿no? Porque también hay desfavorecidas, ¿no? El hecho de que no tengan que venir, porque a veces también hay casos de que ellos como están poco, bueno, ¿no? Es un distrito que no tiene muchos medios económicos, ¿no? Y a veces hasta dejan de venir porque no tienen para pagar el pasaje o la moto. Eso también es fácil. Por ese lado sí va a ayudar, ¿no? Ok.

**E:** Una última re-pregunta.

**e:**No, —----, si tienes que ser, ya. Pero por favor que no se tan largas porque yo…

**E:** No, no, ya estamos por terminar, no te preocupes. Me comentabas que, por ejemplo, ustedes todavía hacían por llamada el tema del filtro a través de una llamada telefónica con un cuestionario que ya está estandarizado, ¿no? Pero luego pasa a ti y tú todavía tienes una especie de filtro, me comentabas, en el que podrías identificar si a la revés de verdad meritaba o no pasar esa atención. Entonces, ¿sería bueno de que esa función que tú haces en tu primera sesión, de volver, evaluar si de verdad merita esa atención especializada, podría ser absorbida por otro profesional de salud mental que esté, digamos, en la posta antes de que pase al Centro de Salud Mental Comunitario? ¿Y qué característica te debería tener esa entrevista para que tú con más seguridad ya empieces a evaluar directamente como un tratamiento?

**e:** O sea, ¿antes de… después de que haya pasado su acogida con la enfermera, pase otro filtro más antes que conmigo?

**E:** Claro, para que solamente te lleguen casos en los que, digamos, ya cuentes tal vez con una historia breve, una anamnesis, y no tener que explorar desde cero porque no estás 100% segura de que el paciente requiera atención.

**e:** Bueno, tiene que ser un… yo creo que también tiene que ser un psicólogo, ¿no? Tiene que ser un psicólogo, un profesional de salud mental que esté capacitado para poder observar bien si realmente tiene la necesidad. Sí, claro, porque yo personalmente preferiría, no sé si es por lo que yo pienso, ¿no? O sea, yo sí prefiero que pase primero, o sea, que ya de frente pase conmigo y ya yo derivar si es que se queda, ¿no? Decidir eso, ¿no?

**E:** Ah, ok.

**e:** Pero sí, me quedaría como un poco… siempre me gustaría yo conversar con el paciente y ver, ¿no? Porque pasa a veces que… esto tal vez es verdad. Personalmente yo quisiera… Que características tiene que tener que sea un buen profesional, que tenga ética, que no… sobre todo la ética, que no solamente piensen que nos vamos a llenar de pacientes porque a veces, ¿no? Hacen esto de que dicen, no, ya por no saturarnos, porque en realidad sí estamos muy saturados, dicen, los regresan, ¿no? Pero en realidad sí necesitan… He tenido pacientes que se han quejado conmigo y entonces lo hacen a veces, ¿no? Como para que ya… un rato liberarse un poco. Entonces sí, la ética del profesional, que sea un buen profesional, que tenga las preguntas… tal vez si es… yo no considero que se deben hacer como medio mecánico porque a veces ellos llenan un cuestionario y no los pacientes se sienten como…

**E:** Entrevistados.

**e:** Sí, y no que realmente los han recibido, ¿no? Los han sido escuchados. Eso mecánicamente y sí he tenido quejas de eso porque me dicen, solamente señor te hacía bien telefónica y me ha hecho un montón de preguntas y ni siquiera me escucharon.

**E:** Ok. O sea, el filtro tamizaje, antes de llegar a ti, no debería ser exclusivamente una evaluación psicométrica, sino debería haber también una valoración del clínico.

**e:** Ajá. Sí, pero de repente con eso van a odiar, no, que no sepan que he sido yo.

**E:** No, no, al contrario, esto ayuda bastante para entender mejor cuál es la situación y como tú dices, tienes una gran demanda y sería bueno de que hay un filtro apropiado para que no tengas esa cantidad de demanda, pero sabemos que siempre van a haber personas que necesitan atención.

**e:** Sí, lo que yo veo es que es de acuerdo al profesional, porque hay muy buenas enfermeras que son muy solidarias, humanas, lo hacen muy bien, pero en todas partes hay personas que son un poco más, no sé, pues, hay muchos factores, ¿no?

**E:** Claro.

**e:** No quiero hablar mal de nadie, pero eso también depende de cada persona, ¿no? A veces hay personas que no se han sentido escuchadas, ¿no? Entonces eso. Y ellos también ya saben, ya piden en el libro reclamaciones, también ya algunos pacientes, ajá. Sí, hay de todo, ¿no Miguelito? Hay de todo un poco, pero sí, siempre yo creo que al ser un centro de salud mental, los pacientes ya vienen como en una desesperación, ¿no? O sea, ya para que se sientan escuchados, para acogerlos, para que aliviar un poquito ya todo lo que traen, ¿no? Entonces, siempre en esa pregunta que tú me hiciste de que si una plataforma nos ayudaría, sí nos ayudaría a ambos lugares, tanto como para el paciente como para nosotros, pero al menos la primera que sí haya contacto, ¿no? Contacto personal.

**E:** Ok. Bueno, esa era mi última pregunta, —-----, muchas gracias por tu apoyo en esta entrevista. Voy a detener la grabación.

**D7: Trabajador_Profesional_Salud**

**E:**Ya. Buenos días, hoy día veintiséis de junio del dos mil veintitrés, nos encontramos con la licenciada —--, del Centro de Salud Mental Comunitario San Gabriel. Eh, bueno, nuestra primera pregunta es que nos cuente brevemente, desde hace cuánto tiempo ha brindado servicios de salud mental a través de TeleSalud, y cómo ha sido su experiencia general, brindando estos servicios.

**e:**Ya, eh, buenos días, sí, eh, bueno, eh, la actividad de realizar las atenciones en salud mental, básicamente, a través de lo que son las llamadas de teléfono, a raíz de la pandemia, ¿no? Antes de eso, aquí en el Centro de Salud Mental Comunitario de San Gabriel Alto, las atenciones se han hecho siempre de manera presencial. Entonces, a raíz de la pandemia, como una forma de mantener el contacto con nuestros usuarios, es que buscamos el poder, como digo, mantener el contacto, y empezar a hacer el uso de estos dispositivos como son el celular.

**E:**Y durante este contexto de pandemia, inició recién estos servicios desde la atención, o tuvieron una experiencia semejante antes.

**e:**No, propiamente no, es a raíz de la pandemia. OK. Antes era...

**E:**Contaron con alguna guía, protocolo, manual, para apoyarse en el cómo van a realizar esa teleconsulta?

**e:**No, lo que nos dieron posteriormente fue más que nada unos códigos para registrar este tipo de atenciones. En sus. En los GIS, exacto, ¿no? Lo que era telemedicina. Entonces, ahí habían códigos que hacían atención a la atención de manera sincrónica o asincrónica, ¿no? Donde decían, por ejemplo, la manera sincrónica era que yo podía llamar por teléfono y la persona me contestaba. Y asincrónica era que de repente como no contestaba la persona, yo dejaba un mensaje, o la persona devolvía un mensaje al número de teléfono por el cual se le había hecho la llamada.

**E:**OK. ¿Usted consideraría importante que hubiera existido una guía de cómo de cómo tendría que realizarse estas comunicaciones?

**e:**Claro, sí, yo creo que hubiera sido, como dice, una guía, ¿no? O una forma de poder ir canalizando este tipo de atenciones sobre todo en el contexto, ¿no? Por ejemplo, de manera personal, el hacer las atenciones a través de este medio, al inicio era como que bastante difícil, ¿no? Como que de por sí impersonal, incluso porque nosotros realizábamos solamente las llamadas, ni siquiera era una videollamada porque no tenemos los implementos para poder realizar la atención. Entonces, básicamente era con el uso de celulares que el personal se agenció para poder hacer uso y realizar este seguimiento telefónico de acompañamiento en el tiempo de pandemia.

**E:**Me comenta que fueron principalmente llamadas. ¿Qué plataformas utilizó? ¿Solo llamadas telefónicas o utilizaba llamadas a través de aplicativos como WhatsApp, Messenger?

**e:**Eh, por ejemplo, el gran porcentaje era a través de llamadas telefónicas, menos en lo que es el servicio de psicología, y uno que otro paciente podía incluso recibir o hacer la llamada, este, por WhatsApp. Pero en la mayoría, más del cincuenta por ciento, era vía telefónica. Después, con el paso del tiempo, pudimos implementar a través del Facebook, por el Messenger, actividades, entre comillas, si le podríamos decir grupales, solo con algunos pacientes que contaban con el servicio de internet, que manejaban las redes, principalmente lo que es Facebook. Pero la gran mayoría de los usuarios de acá del centro de salud mental de la zona de Villa María, donde nosotros nos encontramos, no contaba con eso, o sea, no tenía el internet, no manejaba redes, incluso, eh, con el celular, muchos solamente tenían las llamadas, ¿no? Los celulares básicos, y eran muy contados los que podía hacerse las llamadas a través del WhatsApp y que ya era una videollamada, ¿no?

**E:**OK. Y en su experiencia, ¿qué es lo que más le ha gustado de estas dos modalidades? Una a través de llamadas y otra videollamadas.

**e:**Definitivamente a través de la videollamada, ¿no? Porque de alguna manera me permite ver a la persona y que la persona también me pueda ver a mí, ¿no? En la relación que se establece con el paciente, nace de este tipo de contacto, vamos a decir así, en presencial, ¿no? Y al perderlo, y solamente a través de la voz, sin una imagen, para algunos pacientes era también un poco difícil, incluso, ellos esperaban a que en algún momento puedan salir de sus casas o que se aperture el que puedan venir a atenderse, ¿no? Si bien es cierto, nosotros hemos venido atendiendo porque habían pacientes que sí, a pesar de todo, era necesario que vengan, pero definitivamente la videollamada era como que de alguna manera poder mantener el contacto visual, el poder ver el estado de la persona, ¿no? Porque a veces por teléfono no era suficiente, ¿no? Si bien es cierto, hay que estar atento más a los silencios, al tono de la voz, pero igual el verlo complementa un poco parte de la información que para nosotros también es importante.

**E:**Y considera que la interacción con el paciente cambió durante estas evaluaciones, es decir, afectaba la elección de que sea por llamada o por videollamada en la adherencia del paciente?

**e:** Yo creo que no, al menos con los pacientes, relativamente a los pacientes de repente un poco más jóvenes era como que más accesible, ¿no? Los pacientes que sí eran adultos mayores les costaba mucho, incluso como que eran sus familiares los que hablaban por ellos y muy poco con ellos directamente, ¿no? Por el tema de usar estos medios, vamos a decir así, un poco más modernos, ¿no? Pero igual yo creo que con el paso del tiempo tanto los profesionales como nosotros, como los pacientes aprendimos que esta también era una forma diferente de poder mantener el contacto con los pacientes o con los familiares.

**E:**Y entre estas dos modalidades de atención, ¿usted o los pacientes consideran que hay algo que realmente les disgustó de estas modalidades de atención en llamada y videollamada?

**e:**No, yo no creo que les haya disgustado, sino que era algo nuevo y es como que estábamos acostumbrados a que vengan, ¿no? Pero no es propiamente porque les haya disgustado, sino que era algo nuevo y costó adaptarse, otros como que no lograron adaptarse, ¿no? Igual al final por teléfono se llegó a hacer el contacto, se estableció de alguna manera una cercanía porque muchos de nuestros usuarios que los conocimos por teléfono porque a nosotros nos derivaban casos con esta línea del 113 que se implementó, entonces bueno, hacíamos la contención, el acompañamiento por las diferentes situaciones que venían, que pedían ser atendidos y en el proceso es como que se fueron adaptando para finalmente cuando ya pudieron venir de manera presencial, era como que por la voz, como que por la voz nos reconocimos, ¿no? Ah, yo sé que es la licenciada, yo la escuchaba así o la imaginaba así, ¿no? Y uno también se hace la imagen de cómo es el paciente y que a veces sí coincidía con quién era cuando lo tenía en frente como a otras veces no. Ajá.

**E:**Y considera que existieron estrategias de comunicación y difusión por parte de sea del Ministerio de Salud, de su red este DIRIS o en su Centro de Salud Mental Comunitario para difundir el uso de las teleatenciones?

**e:**Sí, porque nosotros, el Centro de Salud Mental Comunitario tiene el Facebook, tiene la red social del Facebook, no estoy segura si tiene el Instagram, pero a través del Facebook incluso nosotros presentábamos ya actividades así grupales, sesiones, ejercicios, sesiones informativas, ¿no? Entonces, sí se buscó ya incluso el mismo MINSA implementaba, ¿no? Y ahí creo que, por ejemplo, en el hospital, en el Noguchi, me parece que ya se venía haciendo este tipo de atenciones, ¿no? Que incluso en algún momento nos capacitaron a nosotros para ver la posibilidad de que ese sistema se implementara, sin embargo por el tema de la logística no se dio, porque nosotros tenemos dificultad acá con el Internet, las máquinas no tienen cámara, ¿no? Incluso algunos, el tema del audio también es otra situación, por eso que nosotros mismos, como le decía, implementamos los celulares, ¿no? Incluso el mismo centro buscó la forma de que los profesionales por servicio tuvieran un celular para poder hacer estas atenciones.

**E:**Cuando me menciona que buscó que se cuente con celulares, ¿era celulares comprados y con saldo pagado por el centro? ¿O cada uno de los profesionales tenía que tener su celular y pagaba su propio saldo?

**e:**Algunos profesionales si optaron por usar su propio celular, a otros se buscó que el mismo centro nos facilitar el tema del pago, ya sea del celular o en algún momento hacer los pagos de los equipos, o incluso era como que prepago, ¿no? Se ponía un saldo, pero después de eso, como que el centro no pudo mantenerlo, entonces cada servicio buscaba de alguna forma poder solventar eso, ¿no? Porque no había un presupuesto específicamente para eso, al menos para nosotros, ¿no? Entonces como una forma de mantener el contacto con los pacientes, y bueno, con el paso del tiempo, como se fue aperturando más el hecho de que puedan asistir, entonces eso se fue reduciendo, ¿no? Y hoy en día se maneja también así las videollamadas o las capacitaciones por estos medios, pero ya está en función de lo que cuenta con el servicio y también el profesional,

¿no? Si no hay datos, si no hay el saldo, entonces es como que uno mismo lo pone, ¿no? Para poder cumplir con eso.

**E:**Entonces, ¿se continúan brindando algunas teleatenciones?

**e:**Sí, por ejemplo, ya ha quedado como parte, creo yo, de este sistema de atención, las teleatenciones. Yo personalmente tengo usuarios que son adultos mayor o que tienen algún problema para la movilización de manera frecuente, entonces yo los llamo por teléfono. Los llamo por teléfono y después hay un grupo que son las técnicas de un hogar protegido con el que damos capacitación, soporte a las técnicas, que se hace por vía el MIT, ¿no? Nos reunimos dos veces al mes y hacemos capacitaciones y conversamos acerca de cómo van acompañando a las pacientes que tienen a su cargo en ese hogar protegido.

**E:**Ah, qué bueno. Casi para finalizar un poco consolidar las percepciones que nos han mencionado, ¿cuáles consideraría entonces que son las mayores dificultades para que un servicio de telesalud pueda ser implementado?

**e:**Yo creo que una de las y la básica es el tema de la logística, ¿no? Tanto los equipos como el servicio de internet y si fuera necesario pues el tema del teléfono, ¿no? Porque sin eso definitivamente no se podría dar la atención como se debe, ¿no? Nosotros en la situación de pandemia hemos buscado solventar y poder mantener las atenciones y hemos visto de acuerdo a nuestros recursos poder hacerlo, ¿no? Pero yo creo que esa es la mayor dificultad. Para nosotros está el tema del internet y los equipos. Hay algunos consultorios que tienen un equipo de computadora pero no tienen cámara, no tienen audífono, micrófono y contamos con una laptop que me parece que fue donación que es la que nos ayuda por ejemplo con este tipo de capacitaciones que le menciono y que incluso cuando hay otros centros de salud que son parte del acompañamiento clínico-psicosocial que nosotros realizamos se puede hacer por ahí las capacitaciones vía ya sea el Zoom o el Meet, ¿no? Pero básicamente es el equipo, ¿no? O sea los equipos. Sin eso yo creo que sería mucho más difícil porque el profesional finalmente se adapta y tiene la capacidad para poder realizarlo.

**E:**Claro. Y desde el otro lado, ¿cuáles considera que han sido los mejores facilitadores o las situaciones que han beneficiado que se haya logrado implementar servicios de telesalud y que haya alcanzado esa adherencia de los pacientes?

**e:**Creo que está el tema de la calidad de la atención, ¿no? Hasta antes de pandemia nuestro centro de salud mental comunitario es un centro que está reconocido dentro de la comunidad. Hemos podido hacer alianza con diferentes agentes y eso nos ha permitido también seguir manteniéndonos y estableciéndonos dentro de la comunidad. Entonces yo creo que eso ha sido un factor que ha influido mucho para que el paciente a pesar de no venir de manera presencial pueda mantener el contacto, aceptar de alguna forma esta nueva forma de relacionarse o de vincularse y para las personas nuevas que recién se se se acercaban o hacían el contacto con nosotros yo creo que es el tema de la atención y también la necesidad de que como todo estaba cerrado, todo estaba como que más dentro de casa el que nosotros seamos una opción para poder comunicar, creo que facilitó el hecho de que a pesar de que no era presencial la atención se diera el tema de permitir ser atendido, ¿no? Yo creo que también el Ministerio de Salud cuando implementó esta plataforma del 113 a pesar de que había gente que decía que no contestan y no llaman yo creo que fue también una forma de poner más a la vista algo que ya venía presentándose sino que no era tan común, ¿no? No era tan usado porque este medio de la virtualidad tiene años sino que creo que para el tema de la salud y que las personas acepten que esta es una nueva forma de atenderse yo creo que ahí es donde hizo la diferencia, ¿no? El promoverlo más, ¿no? Sí, sí, sí, al haberlo promover, creo yo

**E:**Se hizo más visible

**e:**Sí, sí, sí, porque esto ya existía, ¿no? Claro, porque antes era como ¿Cómo vas a atender? Yo quiero que me atiendas de manera física, ¿no? Y bueno, una desventaja fue el que no teníamos las cámaras, pero yo creo que si hubiésemos tenido cámara hubiera sido muchísimo mejor

**E:**Claro, aunque me comentaba que su población tal vez no todos tenían

**e:**No todos tenían, claro. Pero al fin y al fin sí hubiera sido más enriquecedor. Sí, seguro que sí, seguro que sí. Entonces, por ahí que a pesar de eso, bueno, hemos buscado yo creo poder hacer la contención de toda esta situación que se presentó durante los dos primeros años, pues, ¿no?

**E:**Claro Y en su punto de vista ¿Qué otros elementos adicionales se deberían considerar por parte de, sea, puede ser del Centro o del Ministerio de Salud cuando se planifica una plataforma de teleatención? Es decir, no solamente a través de celulares y llamadas por WhatsApp o Zoom, sino de repente que exista una plataforma como tal en la que ustedes como usuario y contraseña puedan ingresar, tener la historia pruebas disponibles para aplicar ¿Qué elementos les gustaría que pueda componer esta plataforma de teleatención?

**e:**Tengo entendido que ya hay una plataforma, sí pero que tengan o sea, que sea quizá adaptada o sería mucho pedir no sé, ¿no? Para, si bien es cierto la medicina atiende, ya sí, los médicos tienen este formato ya incluso el salud maneja, sí no contacta, a veces contactan al paciente vía telefónica y ya tienen todo una plataforma para ellos, ¿no? De repente como usted dice, dentro de lo que es el aspecto de psicología, pues, tener propiamente una plataforma o un área en el que no solamente la videollamada sino también el acceso a las pruebas o cuestionarios y que sea fácil de poder registrar ¿no? Porque teniendo en cuenta que cuando el usuario va describiendo su historia o generando su malestar o su sintomatología, o sea, por ejemplo yo no soy taquígrafo ¿no? Y me sería muy difícil, ¿no? O sea, yo tendría que escribirlo y luego tener que pasarlo al formato, ¿no? Entonces no sé de qué forma se podría implementar eso o como que te den más tiempo para llenar porque creo que había plataformas donde tienes determinado tiempo para llenar esto, ¿no? Y claro, ser preciso, breve va a influir mucho, entonces por ahí que de repente sería genial que se consideren ciertos aspectos dentro de lo que es la atención psicológica ¿no?

**E:**De la mano con las necesidades del psicólogo ¿no? De repente algunos aún usan pruebas proyectivas o psicométricas o sirven

**e:**Claro, las pruebas psicométricas, ¿no? Porque las proyectivas sería un poco más difícil pedirle al paciente a no ser que le diga, ¿tienes un papel? ¿Tienes un lápiz? ¿no? Quizá de repente cuestionarios como dice usted, más psicométricos como que más puntuales si yo voy preguntando y marcando la respuesta ahí en ese momento y que de repente si no terminara por el tiempo de la atención tenga la posibilidad de que se guarde y volverlo a abrir y encontrarlo ahí ¿no? No que lo guardo y después yo no lo encuentro ¿no?

**E:**Y que al aplicarlo ahí en el sistema te dé el resultado ahí mismo también. Que digo que o sea que si bien se encuentra el instrumento en el sistema que no sirva solo para marcar como un checklist, sino que me dé ya el resultado la interpretación.

**e:**Ah claro sería genial también ¿no?

**E:**Para no tener que usted que dedicar tiempo a buscar sus manuales llenar ¿no?

**e:**Corregir. Claro, sí eso sería genial y eso implica pues o sea no solamente tener la plataforma sino tener, como le digo yo, para nosotros es importante la logística porque sin eso puedo yo tener la plataforma pero no tengo el acceso desde el celular llenar un cuestionario es muy difícil ¿no? para el momento de la atención o incluso el tener que después registrar todos los datos ¿no? Por ejemplo para nosotros cuando se buscaba implementar lo de la telemedicina vinieron a hacer estas capacitaciones para nosotros era difícil por el tema del internet o sea, era imposible todos los profesionales cuando estaban en la capacitación estaban con su celular o sea no podíamos acceder a las computadoras que hay en algunos consultorios ¿no? incluso el tema de la que son muy antiguas hay todo un proceso para poder pedir ya sea que le pongan un antivirus o que la revisen ¿no? entonces eso hace que sea más difícil

**E:**O sea digamos, usted puede percibir que si hubo voluntad de su centro o el jefe del centro o el mismo personal de salud de modernizar pero las barreras fueron más administrativas o sea no encontraron de repente el apoyo que les pueda guiar esos son los formatos, se llenan así, así se hacen los solicitudes de presupuesto, etc. Claro, o sea, y están los formatos, todo un tema en el que cuando se han pedido requerimientos nos piden a nosotros una ficha técnica de una computadora yo no soy experta en qué pedir, de cuántos gigas la memoria, etc. Entonces sí, y sobre todo para nosotros es el internet, sin el internet hoy en día prácticamente si tengo una computadora no me va a servir.

**E:**Es como una máquina de escribir.

**e:**Exacto, ustedes lo han dicho. Actualmente sí.

**E:**¿Sí cuentan con internet ahora?

**e:**Tenemos un internet creo que tiene solo 8 megas y se le da prioridad a la computadora que está en el área de admisión porque ellos tienen que revisar si el paciente tiene SIS para que se les pueda hacer la ficha que también hoy en día está siendo impresa para atender al paciente. Entonces la única y la de jefatura que es por el tema de documentos después las otras computadoras no tienen el internet, entonces es básicamente para tipear y los documentos yo los tengo que sacar con el USB para después ver cómo los imprimo de acuerdo a lo que necesito.

**E:**Con eso también podría ser el reto.

**e:**Claro. Incluso solamente contamos con un teléfono fijo. Después todas las llamadas como le digo se han hecho a través de los celulares personales o que el centro buscó la forma de agenciarnos teléfonos porque tampoco MINSA o DIRIS no tienen un presupuesto para eso. No estaba considerado.

**E:**No eran sus propios números. Es decir, no recibían llamadas fuera del horario de pacientes.

**e:**Claro, por ejemplo, hay compañeros que usaron su teléfono y sí recibían llamadas fuera de sus turnos. Eso era incómodo. Entonces yo prefería agenciarnos un celular diferente al personal porque con la misma situación de ansiedad, de tensión, de hecho, qué iba a hacer que el paciente te busque sin considerar un poco a veces sus horarios personales. Definitivamente no. Algunos servicios, como le digo, tenían su celular. Por ejemplo, terapia de lenguaje usó básicamente videollamada porque sí era necesario. Entonces la mayoría de sus pacientes trataron de ver cómo poder hacer las sesiones virtuales con la cámara. Pero aquellos que no, entonces simplemente era orientación, contención, ver, dar pautas. Porque en terapia de lenguaje sí es importante verlo porque hay que hacer ejercicios de fonética, vocalización, etc. Entonces era bastante difícil. Claro, sí, es una situación muy semejante me paso.

**E:**Bueno, licenciada —--, muchas gracias. Esa era nuestra última pregunta. Creo que he podido comprender mucho cómo ha funcionado la teleatención en el Centro de Salud Mental Comunitario de San Gabriel. Y nuevamente agradecerle por su tiempo y su aporte en todo este periodo.

**e:**Ay, bueno, ojalá que le haya servido y que de alguna forma pues tampoco no es que nosotros queramos hacer quedar mal, ¿no? Ni a nuestra institución, ni a mí, ni a Nadir, pero es un poco lo que nosotros hemos venido haciendo. Y bueno, yo creo que hemos buscado lo mejor dentro de la situación y los recursos que teníamos y siempre el personal bastante dispuesto a poder resolverlo, ¿no? Y como dicen, hacer frente a esta situación que llegó tan inesperada y bueno, pues se mantuvo por buen tiempo, ¿no?

**E:**Correcto. Igualmente le reitero que este video es anónimo, es decir, no va a ser proyectado ni total ni parcialmente por ningún medio. Solamente va a servir para una auditoría que quisiera revisar que existió o una entrevista entre nosotros. Y no, no, sus datos personales tampoco van a ser compartidos como parte de algunas de las afirmaciones que se hayan dicho en esta reunión.

**e:**Bueno, igual, muy amable pues. Muchas gracias Licenciado. Gracias por la espera porque hoy día hemos tenido, estamos de atención, ¿no? Y buscando hay que tener el espacio, el pacientito que no viene, entonces ahí hemos hecho.

**E:**Muy agradecido porque se haya podido dar ese tiempo también.

**e:**Ya, de nada, pues entonces ya cualquier cosa igual, porque ahí yo vi en el cuestionario que decía fase 1, no sé, entonces si en la fase 2 también igual nos pedirán algún tipo de información, entonces ya.

**E:**Ah, no, este, en la fase 1 es con los personales de salud del Centro de Salud Mental Comunitario. La fase 2 es el desarrollo de una plataforma y la fase 3 es la prueba, pero se va a contratar psicólogos para que apliquen, ¿no? Entonces esos que utilicen van a ser los que van a, este, a ser entrevistados en las fases que vienen. Ah, ahorita es un poco con las personas que ya lo utilizaron, que ya han sido parte.

**e:**Ya. Ah, ya. Bueno, yo decía, de repente si hay una fase

**E:**No, pero si le voy a mandar los links de capacitación para que puedan hacer difusión entre los profesionales de salud que ustedes conozcan.

**e:**Ah, ya. Ya, pues, licenciado, más bien muy amable y ha sido un gusto de poder completar y, bueno, tener, como dice usted, información de lo que nos ha tocado vivir.

**E:**Sí. Muchas gracias.

**D8: Trabajadora_Profesional_Salud**

**E:**Ok, doctora —----, como ya habíamos comentado anteriormente, hoy día hemos venido a hacer la entrevista con usted, por ello me gustaría que por favor pueda brindarme su nombre completo y su profesión.

**e:**Mi nombre es —----- y soy médico psiquiatra.

**E:**Ok, está bien. Entonces doctora, quería iniciar esta entrevista un poco preguntándole, ¿usted desde hace cuánto tiempo es que viene brindando este servicio de telesalud mental?

**e:**El servicio de telesalud inició en el 2020 a raíz de la pandemia, que se interrumpieron las atenciones presenciales como eran habituales y empezamos a adaptarnos a la situación de coyuntura y empezamos a brindar atenciones vía telefónica y a organizarnos en general.

**E:**¿Usted desde el 2020 ya estaba aquí en Carabayllo?

**e:**Sí, ya estaba acá desde el 2019.

**E:**Ah, 2019, ok, entonces ya se había familiarizado un poco con la situación, ¿verdad? Y bueno, ¿cómo ha sido el, digamos, el brindar el servicio? ¿Por teléfono, por Zoom, Meet? ¿Cómo ha sido? ¿Me podría describir un poco eso?

**e:**Ok, la atención para los usuarios principalmente ha sido vía telefónica, en muy pocos casos ha habido una videollamada por la limitación de los usuarios, no todos contaban con el servicio de internet y el manejo también de los diferentes programas, ¿no? Del Meet o de Zoom o del WhatsApp, no todos cuentan con WhatsApp. Las reuniones como equipo sí lo hacíamos vía Zoom.

**E:**Ok, pero en la parte, digamos, de atender específicamente al usuario, ¿no?, al trabajador, ¿este? ¿Lo hacían a través del teléfono principalmente?

**e:**Así es, a través del teléfono y llamábamos o hacíamos las teleconsultas con nuestros usuarios, también si es que se, digamos, se enfermaba uno de los trabajadores, hacíamos un seguimiento también vía telefónica.

**E:**Claro, ¿y me puede comentar un poco cómo era este proceso? O sea, entiendo de que ustedes de alguna manera tuvieron que organizarse, ¿no?, para hacer algo nuevo, no se había hecho antes, recién se está implementando, ¿cómo fue al inicio? ¿Hubo alguna, de repente, guía de estándares sobre esto debe de hacer, esto debe ocurrir, así debe ocurrir? ¿Hubo?

**e:**Primero empezamos a capacitarnos con los cursos que se brindan en el SAP, que es del Ministerio de Salud, y ahí empezamos a entender un poco los términos nuevos, ¿no?

Algunos eran telemonitoreo, otros eran teleconsultas, teleorientación, teleorientación, telemonitoreo y eran términos nuevos para nosotros, ¿no? Teníamos dificultad para registrarlo también, ya que tenían códigos nuevos. Y también la manera como lo registrábamos, porque nosotros solemos registrarlo vía escrito, en un formato escrito a mano, y ahí en el tiempo de la pandemia empezamos a registrar todas nuestras atenciones vía virtual. Entonces, en ese sentido, tuvimos al principio, digamos, fue algo nuevo y empezamos a capacitarnos gracias al curso, y ya una vez que empezamos a entender bien cómo era el sistema, empezamos a trabajar.

**E:**Ok, entonces yo entiendo que usted se capacitaron por el uso de herramientas digitales, pero de repente no hubo como un soporte sobre un flujo de, digamos, esta es la atención presencial, como veo que hay un flujo ahí en el ingreso, pero la atención virtual, ¿Hubo algo así como una guía que le indicara qué pasos se iban a hacer, cómo? ¿O ustedes tuvieron que definirlas?

**e:**Bueno, de acuerdo a las capacitaciones, íbamos aprendiendo de que primero solía hacerse la teleorientación a cargo del personal de enfermería principalmente, y ellos de acuerdo a la necesidad que solicitaban sus usuarios, programábamos un telemonitoreo para los usuarios conocidos. Y sí, pues al principio fue nuevo, empezamos a conocer el sistema, que no lo habíamos usado antes, y ya una vez que empezamos a conocerlo mejor ya registrábamos y trabajábamos mejor. De hecho hemos recibido esa guía de cómo se debe hacer gracias a esa capacitación, y así hemos ido aprendiendo todo eso.

**E:**Ok, ok, ah, qué bueno, me alegro bastante. Entonces, por ejemplo, usted menciona también que allí en ese trámite, en ese tipo de trabajo utilizó para anotar los datos y herramientas electrónicas. ¿Cómo qué tipo de instrumentos utilizó?

**e:**Ah, ok, teníamos un formato, aquí usamos el FUA, es una hoja en físico que llenamos, pero de manera virtual era el FUA, que era de teleatención. Y nos mandaban ese formato y nosotros teníamos que llenar los datos del usuario, también hacer una descripción de lo que habíamos conversado, de la intervención que habíamos hecho, y luego lo enviamos al correo de signado.

**E:**Ok, entonces, por ejemplo, llegaba un paciente, ¿cómo usted por ejemplo conocía, sabía de un caso? ¿Tenían que venir aquí presencialmente, se comunicaban por teléfono, les escribían al WhatsApp? ¿Cómo fue al inicio?

**e:**Ya, primero se cerró todo de manera así rápida, ya teníamos usuarios citados, entonces lo que hacíamos era, veníamos nosotros igual de manera presencial, dos veces a la semana por lo menos, y a nuestros usuarios citados, cogíamos los datos, su número de teléfono, su nombre, etc., y los empezamos a llamar, como hacer un telemonitoring. Luego, cuando nos fuimos organizando mejor, primero era con la teleorientación, llamaban al centro, los usuarios llamaban desde sus casas al centro de salud mental, y solicitaban, necesito, solicito una atención con psiquiatría, con psicología, ya no tengo medicamentos, o he tenido este problema, he tenido esta crisis, entonces ya el personal le asignaba una cita con ya sea psicología o psiquiatría para darles atención. Y se colocaba una semaforización, por así decirlo, porque había usuarios que venían, necesitaban una atención más urgente, se les ponía rojo, digamos, otros que no eran tan urgentes, se les ponía verde. De acuerdo al paso del tiempo, también, si había pasado 5 días, 6 días, y no se les había llamado, también pasaba rojo.

**E:**¿Y eso era en el sistema, en un Excel? Ah, en el sistema, tenían la opción de poder hacer eso.

**e:**Ah, sí, en el sistema tenían esa opción.

**E:**¿Y eso fue igual con los trabajadores?  En un caso, por ejemplo, un trabajador necesitaba atención porque un familiar había tenido COVID, él había tenido COVID, y quería recibir atención, digamos, no pasar por una teleorientación, pero ¿cómo era ahí? ¿Era semejante?

**e:**Cuando eran personal aquí del centro, porque varios se contagiaron, entonces nos dividíamos, por así decirlo, digamos, se ha enfermado la enfermera tal y lo va a asumir o le va a atender o va a hacer el seguimiento, el equipo de adicción, por ejemplo, o el equipo de niños. Entonces ya sabíamos el psiquiatra y el psicólogo de ese servicio, íbamos a hacer las llamadas respectivas. Como que fue un poco más sencillo de organizarnos ya que nos conocían. Y a veces la DIRIS nos enviaba trabajadores de otros centros, nos enviaba los datos. El trabajador de tal centro se ha contagiado y está con descanso desde tal día para que le hagan el seguimiento. Y también le asignábamos así un equipo, y aquí trabajamos en equipo de niños, de adultos, de adicciones y de participación comunitaria. Entonces un equipo lo distribuíamos o le asignábamos para que en el seguimiento se trabajara.

**E:**Entonces en ese sentido, digamos, también la atención era por teléfono. O por alguna plataforma, Meets o...

**e:**No, generalmente era por teléfono.

**E:**Era, digamos, muy similar. El contacto llegaba, digamos, referido o entre ustedes, digamos, se enteraban, llamaban, ¿no? De acuerdo a los datos que les daban, de acuerdo a esa información, le daban una telemonitoreo o pasaba por teleconsulta, ¿no? Entiendo que en función de eso también anotaban la problemática en el sistema, igual ponían en verde o en rojo si era más urgente. Era muy similar en todo caso. ¿Cuál podría ser, digamos, que es la diferencia más notoria que usted pudo de repente encontrar?

**e:**En atención para trabajadores y para usuarios. Bueno, en trabajadores, de hecho, ya había una mayor predisposición, ¿no? Porque coordinábamos de manera más directa. Somos del centro y los que ya nos conocían sabíamos que nos presentábamos, ¿no?Somos de aquí, volvíamos a hacer los seguimientos y vamos a llamar a determinada hora. Eso era más fácil, coordinar al momento de la intervención o de la atención. En cambio, con los usuarios, a veces les llamábamos y no respondían. No había forma de comunicarnos o nos estaban esperando y resulta que nosotros estábamos con otro usuario y no le llamamos exactamente ese mismo tiempo. Y cuando le llamábamos ya estaba haciendo otra actividad y no nos respondía. Entonces había un poquito más de dificultad para coordinar el momento exacto con el usuario que con los trabajadores, ya que con ellos había una comunicación más directa, ya sabían que nos íbamos a llamar, ya sabían cómo es la atención, cómo es el apoyo.

**E:**¿Por qué tu motivo cree que de repente fue más sencillo coordinar con los trabajadores que con los, digamos, usuarios?

**e:**También podría mencionar esto de las ventajas y desventajas que puede tener la virtualidad por entre la presencialidad. En la virtualidad a veces no podemos ver el lenguaje corporal, el lenguaje no verbal. Y en cambio cuando ya los conocíamos, ya sabíamos, también ya nos conocían, tal vez había más confianza, nos decían con más precisión cómo nos sentían. En cambio cuando eran usuarios que no nos conocían mucho, a veces nos decían, sí, sí estoy bien, no pasa nada, pero no podíamos tener esa información de la observación directa que podíamos obtener.

**E:**Claro. ¿Cree también de repente que el espacio puede haber influido? ¿Tener de repente una llamada puede ser diferente que tener una atención en un consultor? Por ejemplo, como ahorita estamos, estamos a puerta junta y no hay quien ingrese a este espacio, a diferencia de por teléfono donde quizá de repente usted llama y le contestan y están de repente yendo al mercado o dejando al niño en el colegio.

**e:**Sí, sí, de hecho, definitivamente también.

**E:**¿Ha tenido algún caso así?

**e:**Sí, muchas veces habíamos pactado a determinada hora y le llamábamos, pero escuchábamos una bulla en el fondo, como que hubiera ido a algún lugar, al mercado probablemente.

**E:**¿Eso pasó también con los trabajadores?

**e:**Con los trabajadores eran menos, porque ya sabían que íbamos a llamar a ese determinado momento y tal vez como que había un poquito más de esa comprensión o responsabilidad, por así decirlo, de que ellos nos esperaban así más de una manera ya establecida. En cambio, en los usuarios sí ha habido bastante, en los usuarios ha habido bastante interrupción muchas veces, la bulla, o que estaban muy apurados por hacer alguna otra actividad.

**E:**Entonces, ¿podríamos decir que en los trabajadores se resalta más esta conciencia de que es una teleconsulta, la espero?

**e:**Así es.

**E:**¿Y el medio de usar el teléfono cree que también, en caso de los trabajadores, influyó en que de repente no contaran ciertas cosas o era de repente más sencillo?

**e:**Yo lo sentí un poco más sencillo, porque ya nos conocían, ya saben cómo se trabaja, nosotros procuramos identificar los síntomas, los signos para brindar una atención, como que hay un poquito más de confianza. Con algunos usuarios que nos conocen, que ya los vemos por tiempo, también hay esa confianza, pero con los más nuevos, los más recientes, sí hay un poquito de desconfianza todavía.

**E:**Claro, entonces usted le atribuye una característica, digamos, en cuanto a establecer este rapport, esta confianza, más difícil a usuarios o trabajadores que son nuevos, de repente, que no saben cómo es el trabajo.

**e:**Claro, conforme ya nos conocen, ya hay más confianza.

**E:**Entiendo que usted ha tenido trabajadores que han sido de la DIRIS, trabajadores de la DIRIS. ¿Cree que eso de repente ha influido por ser también trabajadores de salud y que conocen cómo es el trabajo?

**e:**Sí, sí. De hecho, los trabajadores, ya sea que trabajan en un centro primario o en un centro comunitario como este, tienen ya el conocimiento de cómo es el trabajo, cuál es nuestro objetivo, síntomas, malestares, para poder brindar una consejería, un apoyo emocional, etc. Así que, efectivamente, eso ayuda también porque hay esa conciencia. En cambio, hay unos usuarios nuevos, hay un poquito de desconfianza al principio, sobre todo.

**E:**Ajá, ok. ¿Llegó a tener algún otro, digamos, trabajador que no sea de la DIRIS, pero de repente sea de la localidad aquí del Cono?

**e:**¿Trabajador?

**E:**Ajá. Por ejemplo, policía, algún otro tipo de, digamos, de trabajador que pidió atención, básicamente, por este tema, digamos, de he perdido un familiar, necesito pasar atención, pero estoy en mi trabajo. ¿Le tocó en algún momento atender algún caso así?

**e:**Sí, hemos atendido policía, por ejemplo, pero ya como usuario, ¿no? Es decir, solicitaban una atención y ya se les aperturaba su historia clínica y se les citaba para un seguimiento.

**E:**¿Y cómo era con ellos? ¿Había también esa, digamos, esa dificultad para acercarse, para que cuenten cómo estaban sintiendo? ¿Así como refiere antes?

**e:**Sí, claro. En un principio había un poquito de esa dificultad, ¿no? No había esa comunicación directa, esa observación directa que uno puede tener para observar la parte no verbal, como mencionas, ¿no? Y ya con el paso de las consultas, de las atenciones, ya iba mejorando esa confianza. Pero al principio sí había un poquito de la dificultad, de que no hay un trato personal, ¿no? Hay un trato a través de un teléfono, a través de una pantallita, no es un trato directo. Pero sí podía ir mejorando con el paso del tiempo.

**E:**Ok, ok, entiendo. ¿Si, por ejemplo, estos servicios se siguen utilizando actualmente?

**e:**Actualmente ya tal vez muy poco, pero yo al menos personalmente no. Son de manera virtual, perdón, de manera presencial, y salvo si no viene el usuario a su cita, sí le llamamos en algunos casos, ¿no? Para hacerle recordar básicamente si tenía una cita hoy día y no ha venido, ¿qué otro día puede venir? Para eso, ¿no? Solamente para coordinar que venga a recibir la atención que ha permitido.

**E:**¿Cuál sería el motivo de que ya no esté utilizando, digamos, este método, digamos, de teleatención, teleconsulta, telemonitoreo?

**e:**Ok, de alguna manera, vamos preguntando también a los usuarios y de hecho prefieren que sea de manera presencial. De manera presencial. Y también es lo más, digamos, lo más usado, lo más, digamos, con lo que más nos hemos acostumbrado, por así decirlo, ¿no? Y hacerlo de manera virtual implicaría volver a utilizar todas esas herramientas, formatos electrónicos, ¿no? Que ya ahora no lo usamos porque lo hacemos de manera manual, ¿no? Y ya nos hemos quedado con esa forma de trabajo.

**E:**Sí, claro. Entonces entiendo que para usted también es más sencillo al momento práctico, operativo, utilizar formatos físicos que los formatos virtuales.

**e:**Claro, sí, sí, de alguna manera sí. Porque usar los formatos virtuales yo sentía que tomaba un poquito más de tiempo. Porque cuando uno conversa con el usuario no estábamos ahí escribiendo, digitando. Lo hacía, yo al menos lo hacía en un cuadernito donde notaba, ¿no? La fecha, tal usuario y lo que íbamos conversando. Y luego tenía que sentarme un tiempo adicional para hacer todo el llenado de datos, ¿no? Y eso, cuando es así presencial, brindamos la atención, ¿no? Anotamos lo necesario, llenamos y termina la atención, ¿no? Y ya pasamos a un siguiente usuario. Pero cuando es virtual termina la atención pero dejo pues puesto el llenado y registro para después, ¿no? Por eso se siente como que hay un trabajo adicional para los usuarios.

**E:**Y lo aplican, ¿no? Ok. Ok, entonces, digamos, algo que podría facilitar si en caso se volviese a implementar este tipo de servicios o ser necesario sería, digamos, esta flexibilidad de la tecnología para que usted pueda, a la par que atiende, ir llenando la información, ¿verdad? Como si fuese un cuaderno donde está anotando las cositas y a la par va colocando en su informe que cosas. Termina la sesión y ahí va a ser firma y termina de llenar su... como si fuese un documento físico, ¿no?

**e:** Ah, sí. Ok. Sí, sí, definitivamente sería mucho mejor. Eso ayudaría que se pueda utilizar. Porque yo pienso que se puede complementar en todo caso porque hay situaciones de usuarios que no pueden venir hasta acá ya sea porque tienen alguna dificultad para desplazarse, ¿no? En esos casos sería muy bueno esto de utilizar las herramientas digitales, ¿no? Pero tal vez con esas facilidades y también un poco con la práctica ya uno se va haciendo más fácil, más sencillo.

**E:**Claro. Entonces también sería necesario de repente unas sesiones de entrenamiento para utilizar estas herramientas digitales sin caso se volviese otra vez, ¿no? O para estos casos particulares. ¿Algo más que de repente podría sugerir?

**e:**El entrenamiento, la facilidad, la capacitación y el tener tal vez los equipos adecuados, ¿no? Porque de hecho utilizamos básicamente nuestra computadora, nuestro laptop o nuestro celular, ¿no? Claro. Podríamos tener esa facilidad porque la computadora que tenemos acá no tiene ni cámara, ni audio para comunicar, ¿no? Tal vez un poquito de las herramientas se puede hacer del ministerio.

**E:**Claro, como que el hacerlo presencial no requiere digamos tanto material adicional. Una copia de la hoja y un lápiz es más que suficiente si lo atiendes presencialmente. De manera virtual tienes que tener un teléfono, de repente el tuyo... ¿Te ha pasado alguna vez que a altas horas te han pedido el teléfono personal?

**e:**Si porque era una herramienta del propio centro, no había esa sobrecarga

**E:**Por mi parte eso seria todo. ¿Algún comentario?

**e:**Es necesario para estar a la par de la tecnología, implementar el servicio de telesalud. Para estar preparados cuando estemos confinados

**E:**Muchísimas gracias

**D9: Usuario_Docente**

**E:**Buenas tardes, nos encontramos con la voluntariamente aceptado participar del estudio de implementación de un servicio de telesalud mental para las profesiones docente, policías y otras profesionales de la salud. Buenas tardes profesora, voy a proceder a hacerle unas cuantas preguntas sobre su experiencia usando los medios digitales en cuanto a su proceso de atención. En cuanto a su experiencia sobre el uso de estas intervenciones, ¿podría describir un poco cómo ha sido su experiencia en todo el proceso de atención por teleconsulta que ha tenido? Me refiero al hecho de buscar atención, a sacar citas o el hecho de haber sido atendido. ¿Cómo fue todo este proceso para usted?

**e:**Buenas tardes señorita —----. En cuanto a la atención, al hecho de conseguir la cita, sí es un poquito problemático porque muchas veces te responde no la persona sino la contestadora y a veces te tenía ahí con la música cinco minutos, diez minutos y una vez se fue por el cansancio lo dejábamos. Pero también el hecho de que cuando uno consigue la cita la atención, como se dice por teléfono, no es como una atención que sea presencial donde al menos tienes para relatar los problemas, los síntomas que tienes y puedes también tener una atención directa. Una atención por teléfono es una atención fría, se podría comentar.

**E:**Y en este proceso en que usted buscaba sacar una cita para atenderse principalmente, ¿cuál era el motivo por el cual quería conseguir una consulta? ¿Era por un control? ¿Era por temas de evaluación? ¿O por qué motivo decidió usted buscar este tipo de citas?

**e:**Bueno, por dos cosas. Yo soy una persona de alto riesgo, por eso que soy diabética y hace tiempo. Y por los que estaba, por el hecho de la situación que estaban pasando en esos días, por los síntomas del COVID. Y eso es lo que nos preocupaba, sentía mucho dolor en el cuerpo, a veces fiebre por rato. Si uno pues estamos con esa atención, tienes o no tienes la enfermedad. Y ahora para atenderte, pues en los hospitales han estado demasiado, se llama muy saturados, donde ya no teníamos oportunidad. Al menos las personas de mi edad, como muchas veces me dijeron ahí, personas de edad ya no se les están recibiendo los hospitales, solamente a los jóvenes que tienen mayor posibilidad de vivir. También ante esa situación, nos encontramos como se dice, preocupados, sin saber qué hacer. Lo único que nos queda ver es sacar citas por teléfono.

**E:**Entiendo que usted ha buscado citas en su seguro de salud, correcto? Sí. Durante este proceso en el cual usted intentó sacar citas para su atención, cómo fue ese proceso? ¿Le pidieron algún requisito? ¿Le pidieron algún código de identificación? ¿O alguna derivación previo, orden de pase previo para sacar esa consulta? ¿Qué requisitos le solicitaron?

**e:**El DNI. El DNI como único requisito, el número, pero también, por ejemplo, yo por la diabetes, tengo que ir a un centro que tenga esa especialidad, por la zona donde vivo, la clínica no lo tiene. Yo tengo que pedir una transferencia al hospital Sabogal. Y para que yo me pueda dar esa transferencia también, pues requiere tiempo. Entonces, ante ese inconveniente, lo único que hacían acá, cerca de la clínica, bueno, ellos ya sabían cuáles eran los medicamentos que me tenían que dar. Entonces, cada mes que iba, ya simplemente me daban nada más, pero realmente pues no me estaban diciendo cómo estaba, cómo está la salud, si se mantiene mi nivel de azúcar, qué otros síntomas tengo. No era como, digamos, hablar personalmente con el médico, ¿no? Como les digo, por teléfono, a veces no te entienden o no te comprenden, ¿no? Entonces no puedes hacer más preguntas de lo que te está, ni contarle todo lo que está pasando. Es algo limitado.

**E:**Desde su perspectiva fue bastante limitada esa atención que recibió. Ahora, ¿más o menos cuántas atenciones vía telefónica, ya sea porque entiendo que la vía principal de monitoreo ha sido por teléfono? O sea, no hubo un contacto quizás a través de una reunión Zoom, un Meet, alguna interconsulta o atención que recibió a través de esta vía.

**e:**Todo fue por teléfono. Sí, todo por teléfono. Y dentro de esas atenciones de monitoreo, seguimiento, ¿cuántas fueron en promedio durante todo este periodo de pandemia? Un aproximado de 5 o 6.

**E:**Y durante esas 5 o 6 de las atenciones que usted recibió, ¿era con el mismo personal?¿Era, por ejemplo, la misma enfermera quien la llamaba? ¿O la llamaba un médico? ¿Quién era quien la contactaba usted?

**e:**Era una señorita. Eso sí, era una señorita. Mujer.

**E:**Ya, ¿y esta señorita le llamaba para agendar primero y luego ya le decía cuándo le iba a llamar el profesional de la salud? ¿O la señorita directamente a usted le decía que era la profesional y la atendía directamente?

**e:**No, sino este llamaba para decirme que me iban a derivar, que me iba a preparar una cita. Entonces ya una vez que concordaban o acordaban la fecha con usted, pues ya usted se atendía con el profesional de la salud.

**E:**Sí, vía telefónica. ¿Y qué tal le pareció este tipo de atención que recibió ya con el profesional de la salud? ¿Qué le agradó de este proceso de atención? Y también, ¿qué quizás no le agradó mucho?

**e:**Bueno, lo que no me agradó es que, como le digo, el médico no está presencial para ver al menos tu estado anímico. Simplemente te da la receta, te escucha y según eso ya te dice esto, esto, eso, una lista de medicamentos que tienes que tomar. Y luego hay que ir a recoger. Y como le digo, es una atención fría.

**E:**Y cuando dice usted atención fría, lo dice quizás porque no había un contacto físico, no estaban en persona.

**e:**Claro, porque por ejemplo yo necesitaba que se me mida la presión, necesitaba que me tome la temperatura. No, ¿no? Entonces esas cosas no teníamos acceso a esos servicios en esos días.

**E:**Ok, entonces más que todo por la relación interpersonal. Y quizás un poco más allá de la atención, digamos interpersonal, la persona, el profesional que la atendió, ¿se mostró empático o empática con usted vía teléfono? O sea, cuando le empezó a preguntar sobre sus síntomas, sobre su estado. ¿Usted pudo percibir quizás un poco de empatía de ese personal? ¿Cómo describiría esa atención?

**e:**Sí, un poco de empatía, pero no como debería ser, ¿no?

**E:**Como realmente debería ser una, digamos, la atención del médico al paciente. Ok. ¿Qué le hubiese gustado que hiciera esta persona quizás para hacerla sentir a usted más cómoda o más confortable durante su atención? ¿Qué le hubiese gustado que hiciera esta persona quizás?

**e:**Bueno, que sea un poquito más empático y dado los síntomas que yo le estaba redactando, le estaba contando, que al menos mandara hacerme los análisis debidos, que me dieran los análisis previos antes de darme la lista de los medicamentos. Quizás un poquito más de empatía y profundizar un poquito más quizás en la patología, completar quizás con otros exámenes, en su caso.

**E:**Ok, bien. Y en todo este proceso, en cuanto ya hablemos de un poco la conectividad, quizás las disposiciones de horario, también la disponibilidad de usted, digamos, ¿cuál sería o cuáles han sido los problemas principales que ha identificado usted durante este proceso para poder ser atendida?

**e:**Sí, en cuanto a la conectividad también, pero un inconveniente ha sido, por ejemplo, la llamada que sea entre ocho, ocho y media, nueve. No es un momento sin que a veces uno está temprano con los deberes de la casa, estás con la cocina prendida, ¿no? Es el inconveniente porque ellos te llaman, suena el teléfono, uno corre, estás ocupada haciendo algo urgente en la cocina. Y suena el teléfono, corres y te das con la sorpresa de que es el médico. Entonces, si vos sí hay que escucharlo, atender y no tienes una cita a tal hora o ya esta es tu cita, te pregunta los síntomas que tienes, cómo vas. Ese es el inconveniente, ¿no? El horario que a veces te llaman, pues un momento en que estás ocupada.

**E:**Quisiera un horario en que usted, por ejemplo, estaba dictando su clase con los alumnos o estaba haciendo otras labores del hogar y por ahí había el conflicto, entonces no podía atender como quisiera la llamada.

**e:**Claro, más aún, ¿no? Si está uno en el trabajo, es imposible atenderlo en el trabajo. Entonces, la única forma de dejarlo en la cita era para los sábados o por la tarde, de lunes a viernes por las tardes.

**E:**Y en algún momento, alguna de esas personas que se contactó con usted para decirle o bueno, para agendarle una cita, ¿le preguntó, señora Alfonsina, en qué horario preferiría ser contactada o preferiría que nos comunicásemos con usted o ellos de frente aleatoriamente elegían un horario para usted?

**e:**No, nunca, bueno, en mi caso no tuve esa oportunidad de que me preguntaran en qué horario me podría recibir la llamada, sino creo que ellos son los que agendaron la cita.

**E:**¿Y cuál hubiese sido el horario? Bueno, ya estamos en esta actualidad de 2023, ya las clases son presenciales. ¿En qué horario a usted, por ejemplo, le hubiese gustado ser contactada?

**e:**Por las tardes, a partir de las cuatro, porque a partir de las cuatro y más o menos, cuatro y media, cinco, yo ya estoy en mi casa. Entonces, a partir de ahí, yo lo puedo atender, ¿no? Ya me encuentro disponible, pero porque yo trabajo en un colegio que es de jornada escolar completa, estoy desde las 7 y 45 hasta las 3 y media de la tarde. Entonces, mientras yo me alisto, salgo, por lo menos de ahí, las cuatro, cuatro y media, por ahí estoy en mi casa recién. Fuera del horario de trabajo.

**E:**Quisiera extender un poquito más el horario, fuera del horario laboral. Y un poco quisiera saber su perspectiva, ¿qué beneficios quizá a usted le podría encontrar o ha podido percibir de esas atenciones que ha tenido vía telefónica, de esas teleconsultas vía telefónica? Si bien ya vimos, digamos, la parte de barreras, la parte menos bonita, digamos, una parte buena, la parte positiva, digamos, ¿qué beneficios quizás usted le encontró durante todo este proceso? ¿Cuáles serían, si es que hay alguno? Bueno, no todo es negativo, ¿verdad?

**e:**Es muy cierto. Siempre tiene que haber un beneficio. Uno de los beneficios ha sido que ya no tienes esa necesidad de ir presencialmente a un hospital o trasladarte en el carro y que corres el riesgo de contagiarte nuevamente, ¿no? Ese es el único beneficio, que te atiendan, que te hagan una llamada en la casa, o al menos, pues, en este caso, que te den un horario adecuado. Esa sería la única ventaja, ¿no? La desventaja es que el horario que te llaman no es lo adecuado, ¿no? Y que no tienes, digamos, los análisis respectivos de lo que te pasa para que recién te puedan dar tus medicamentos, sino simplemente te escuchan, te escuchan y ¡pam, pam, pam! Te dan una lista de medicamentos y ya se acabó. Eso es lo que habría que mejorar.

**E:**Ok, si usted tuviese la oportunidad de quizás asistir a un programa, o ser parte de un programa de teleconsulta, y hablando un poquito más del plano de salud mental, quizás, ¿qué le gustaría que tuviese ese programa? ¿Qué podría ser ese programa para usted? Considerando que va a abordar la parte de salud mental, quizás un poquito el manejo de depresión, ansiedad y otras sintomatologías psicológicas. Y pensando también de qué va a ser de manera virtual, ¿qué le gustaría que tuviese ese servicio de teleconsulta para que usted se sienta, digamos, más cómoda o para que no pierda el seguimiento? ¿Qué le gustaría que tuviese este programa?

**e:**Mucho. Me gustaría, como el programa es de apoyo en salud emocional, los maestros necesitamos mucho que trabajemos con seres humanos. Y un maestro, una maestra, necesitamos tener muchas hechas estrategias, recursos para trabajar con los diferentes tipos de estudiantes, porque nuestros estudiantes vienen de hogares diferentes, algunos estudiantes vienen con un carácter a veces que no le puedes, digamos, decirle, alzarle la voz o llamarle la atención con una voz alterada, porque los niños se pueden sentir mal, o a veces los chicos, chicos que son pocos comunicativos, y así muchas cosas. Y realmente en esa parte yo creo que los maestros necesitamos bastante, como se dice, apoyo emocional para que nosotros también podamos darle también un buen soporte emocional a nuestros estudiantes. Porque, como le digo, ellos tienen diferentes actitudes, carácter, diferentes formas de comportarse, diferentes formas de reaccionar, y realmente nosotros tenemos que lidiar con todos ellos. Y por eso es que a veces un colegio, entramos, un profesor de secundaria, entramos, ingresamos por horas a un aula, estamos 45, 90 minutos, tenemos que salir, entrar al otro aula, también otros 90 minutos, entramos y salimos, entramos y salimos. Y a veces en ese tiempo, a veces no podemos, a veces se nos va algún estudiante que de repente vino ese día, de repente vino triste, sin querer trabajar, o lo ves que estaba preocupado, y a veces esos tiempos que se nos va rápido, a veces no nos damos cuenta. Entonces a veces se nos escapan las manos, entonces por eso que un maestro si no está bien fortalecido emocionalmente, yo creo también cómo podríamos también ser su fortaleza para ayudar a ese estudiante que siga adelante. Entonces esa parte sí, yo creo que sí los maestros necesitamos un soporte emocional bastante, bastante.

**E:**Entonces a usted le gustaría ser parte de ese programa, sentirse también un poco asesorada, guiada, quizás durante todo este proceso, para poder fortalecer su salud emocional, mental, y también que usted pueda con esto apoyar a otros su salud. Me parece que sí, creo que es necesario. Es necesario. Y para todos los docentes, me gustaría que ese programa, más que teleconsulta, llegase a cada colegio.

**e:**Creo que unos 45 minutos, una media horita, de ponencia, charlas que usted pueda dar a los maestros, porque yo sé que ellos van a dar su tiempo, pero durante la jornada de trabajo, que ustedes dieran estas charlas, yo creo que sería de muchísima ayuda.

**E:**Muy bien, muchas gracias profesora —---, ha sido un gusto conversar con usted.

**e:**Gracias a usted señorita —----.

**D10: Usuario_Docente**

**E:**Ok, ¿qué tal —---? Buenos días, me gustaría por favor que te presentes, para iniciar la entrevista.

**e:**¿Qué tal? Buenos días, mi nombre es —-----, ¿número de DNI necesario?

**E:**Sí.

**e: —---**--, último dígito es —.

**E:**Ok, entiendo que tú eres docente y bueno, hoy día vamos a tener una entrevista para poder hablar un poco de tu experiencia, habiendo recibido alguna atención en teleatención virtual, remota. Entonces, me gustaría saber, ¿Cuándo te has atendido? ¿Cuándo es la última vez que te has atendido?

**e:**El año pasado, fue la atención remota, no miento, el año 20-21 cuando tuve COVID. Ok, ok. Ahí fue la atención que tuve en forma remota, no sé si es bueno mencionar la clínica, porque es una clínica privada, porque tengo un EPS de particular, fue mediante una cámara en la cual me pidieron evaluaciones, que explique lo que sucedía, mis signos, síntomas, qué pasó, una breve narración, y de acuerdo a eso, la doctora que me atendió me indicó de que iba a venir al laboratorio, se acercó a mi domicilio, me sacaron las pruebas, salió positivo, y la medicación fue también por medio del IBERI, que es el EPS que lo cubre.

**E:**Ok, entiendo entonces que tu atención fue a través de una plataforma, digamos, que te permitía visualizar a la otra persona.

**e:**Sí, así es.

**E:**¿Fue el Zoom, Meet?

**e:**Ajá, fue Zoom.

**E:**Zoom. Ok, ¿Cuántas sesiones tuviste atención así remota?

**e:**Una, dos, tres, cuatro sesiones. Las tres primeras fueron por un tema de COVID, porque fue primero una evaluación, laboratorio, lectura de análisis, y luego ya a los 14 días, creo que 15 días, nuevamente con la misma doctora, a ver cómo voy, y luego me dieron el ARCO. Y la última fue por un tema de uso de las mismas, entonces también fui por medio de la virtualidad, la cita médica, que luego me dijo que serían evaluaciones que no seamos presenciales. Se hicieron cuatro hasta el momento. Ya luego, este año, sí, este año, el año pasado, pero ha sido presencial, ya no ha sido virtual.

**E:**Ok, entiendo entonces que las tres primeras fueron un poco como para analizar tu situación, y la última ya fue más como para darte una orden para que tú acudas ya de manera presencial a un, digamos, atenderte, ¿Verdad?

**e:**Así es.

**E:**¿Qué tan fácil fue acceder al servicio? Bueno, realmente, personalmente me sigo muy fácil.

**e:**Muy fácil, ¿No? Bueno, también la aplicación del EPS es bien amigable y es fácil de deducir, ¿No? Ese es lo satisfactorio.

**E:**Ok, entiendo entonces que en tu primera, digamos, tu primer contacto no fue complicado, fue algo sencillo. ¿Qué tuvo que pasar, digamos, como para poder acceder a este primer, atención a este primer contacto? ¿Qué tuvo que pasar? ¿Qué sintió? ¿Me sintió más? Digamos, llamaste por teléfono, ellos te contactaron.

**e:**Te explico, por lo tanto, compañero, te explico. Lo que fue inicialmente, primero, cuando me dieron este, yo tengo un EPS particular, o una entidad. Entonces, y luego de esta entidad nos dieron una rama de, bueno, un file de PDF de las clínicas en las cuales me puedo atender. Entonces, yo en una de esas clínicas con mi esposa, descargamos la aplicación y automáticamente descargando la aplicación te salía, ¿No? ¿Qué cita médica deseas y en qué horario y qué mes? Como el calendario de Google, similar. Tú colocabas el tema, la cita médica, pero en este caso había, vienen cuatro opciones, medicina general, geriatría y pediatría. Entonces, en este caso, pues adulto, medicina general. Colocaba la fecha y la hora, por medio de WhatsApp me respondían, me respondían este proceso, aceptaban, me llegaba ya por correo el tema del Zoom, de la ruta de acceso y me indicaban claramente que tengo que estar 15 minutos antes, 15 minutos antes para estar en cola. Yo ingresé y me acomodaba todo en mi computadora y como actualmente así, escribía y con 15 minutos ya estaba escribiendo, ya estoy acá, por ejemplo. Y en unos casos, a veces la doctora se demoraba, a veces entraba puntual. Pero si, como te digo, era una aplicación amigable. Ingresar y era solamente leer y digitar, no había otra cosa.

**E:**O sea, digamos, era sencillo identificar los botones para agendar, era clara, digamos, la aplicación, no había tanta cosa, no era tan enredada.

**e:**Realmente me sorprendió mucho porque era recontra, claro, es decir, había citas médicas, con quién especialista, con quién, elegías al doctor o la doctora, le ponías un clic, como un check, y luego automáticamente aparecía el calendario. Y tú colocabas la fecha. En otros casos, la segunda o tercera vez ya no colocaba el calendario, sino ya aparecía con la doctora, aparecía sus horarios de atención y qué día está libre. Y tú así como determinaba el asiento del avión, así tal cual.

**E:**¿Y tenías que registrarte o podías ingresar sin loguearte?

**e:**No, sí tenía que registrarme, porque para ingresar a esto yo tenía primero que ingresar primero por aplicación en mi EPS. Y cuando ingresaba a esta otra aplicación, automáticamente me jalaba la información.

**E:**¿Eso te parece que fue algo beneficioso? Que digamos, el hecho de haber tenido primero que inscribirte en tu EPS y luego que la información se jalara. ¿O te hubiese sido más sencillo que desde la misma EPS automáticamente, no sé, te identifique, o consideras que tal y como fue, estuvo bien?

**e:**Creo que de repente es lo ideal, pero creo que hubiera sido que desde la misma EPS me jale automáticamente la clínica. Pero hay vistas de sistema engorroso. Bueno, como decía, como ha sido un tema de atención, no había una premura de mi parte, no ha sido una emergencia, entonces no tomaba con calma, pero si me pongo en el otro lado de que ha sido algo muy rápido, tema de urgencia, emergencia, pues de mala tiempo.

**E:**Ok, entonces entiendo que en el contexto de no urgencia de tu parte, digamos, el diseño de la aplicación, la facilidad que tenía, era de alguna manera open, pero si, tú hubieses estado en una situación de mayor urgencia, quizá la forma en cómo esto, estas facilidades, no hubiesen sido totalmente suficientes.

**e:**Claro, o de repente hacer un botón, caso de emergencia, urgencia, tú automáticamente ingresas a emergencia, urgencia y te sale la parrilla de acciones que necesitas.

**E:**Ok, entiendo, está bien. Ok, entiendo que tuviste cuatro sesiones.

**e:**Sí.

**E:**¿Puedes hablarme un poco de las sesiones? Entiendo que la sesión 1 fue para conocerte, las dos, tres primeras sesiones sí, pero ¿podrías darme un poco más de detalle de repente de cómo fueron estas sesiones?

**e:**Claro, la primera sesión fue con una doctora en la cual se presentó, soy doctora tal, y luego me dijo, cuéntame por favor, cuéntame qué tienes, y yo le empecé a detallar. En aquel entonces, se acordaremos, en el tema de COVID, decía mucho que tienes que apuntar el tema de la fiebre, si tienes fiebre, toda ese información le envié el pantallazo y ella lo veía. La doctora en este caso accedió mucho a escuchar, bastante a escuchar, es lo que me llamó mucho la atención, cuando es un tema más presencial, el doctor escucha o la doctora te escucha y igualmente dice ok, te recetan, en este caso se escuchó, le explicaba todo lo que tenía, y luego de eso, cuando terminé de parar, me dijo uy, entonces vamos a sacarte análisis, de frente, sacarte análisis, a tu celular, a tu whatsapp, te va a llegar la receta, tu whatsapp te va a llegar la fecha que venías al laboratorio, entonces, esa fue la primera.

**E:**Una pregunta, ahí, ¿por qué entiendo que esto de que te parece extraño, tiene una connotación positiva para ti, de que la doctora haya escuchado, verdad?

**e:**Sí, lógico, sí, sí. ¿Por qué? Porque cuando he ido, por ejemplo, a hospitales o clínicas también presencialmente, me ha pasado que cuando estoy frente al médico, médico, doctora, le explico y en un momento dice ok, paramos ahí, ya sé lo que tienes. A veces siento, yo no soy médico, a veces siento que, siento yo que falta que me pregunte, si soy alérgico a algo, que exámenes me han sacado, referencias más al ámbito laboral posiblemente, pero no, acá en este caso sí me parece muy positivo de que empecé a explicar todo lo que tenía y apuntaba, apuntaba, apuntaba. Lo que sí yo me he dado cuenta, que lo puedo aclarar, es que la tema, por ejemplo, estaba en un tiempo de 30 minutos de atención, como máximo, por ejemplo, era de 8 a 8 y media, el otro paciente ingresaba, no sé, de 8 y 35 a 9 y 10, así 30 minutos, pero obviamente el tema propio de la evaluación, el más extenso era de 5 minutos, 6 minutos, no creo que me haya demorado más. Igual, el tema de la receta es igualito. En la segunda sesión, que ya fue mucho más rápido, ¿por qué? Porque en la primera me sacaron las pruebas de sangre, que salió positivo, me enviaron las medicinas automáticamente preventivas, y en la segunda cita virtual, la doctora me dijo, ¿sabes qué? Tienes el COVID, te ha salido positivo, ¿cómo vamos con la tos? ¿Hay carraspera? ¿Alguna sensación que has perdido? No, no, ninguna sensación. Vamos a continuar con los mismos jarabes, con los mismos medicamentos, que igual, ¿no? Por delivery te van a llegar las medicinas. El mismo día y la tarde me llegó la receta médica PDF, y no me llamaron de la Libre, y el mismo día y la noche, o el día siguiente, creo que me llegó la medicina.

**E:**Para ti entiendo que fue muy cómodo y quizá algo positivo el hecho de que toda la información, digamos, que tenías tú que hacer, tanto recetas como indicaciones, te llegara al WhatsApp. ¿Sí? Te resulta entonces más cómodo que de repente te lo diga verbalmente, y ya, como usualmente es una consulta presencial, ¿no? El médico te dice, mira, vas a tomar eso mañana, tarde y noche, te pone una receta, ¿no? De veces su letra es bien difícil de entender, le da la receta y tú ya vas, ¿no? Y a veces un poco desorientado, no sabes qué finalmente te dijo. Entonces, para ti entiendo que es positivo que no solamente haya, digamos, te lo haya mencionado en la sesión, sino que luego hayas recibido en un formato digital, más entendible, elegible, las indicaciones.

**e:**Así es. Sí. Eso para mí pasó, fue lo mejor, lo fabuloso, porque... ¿Por qué? Porque a veces, como tú comentas, el médico escribe una letra, ¿no? Un poco no tan entendible, que tienes que adivinar, se toma cada 12 horas, cada 8 horas, otra vez al día, ¿no? En este caso, estaba impresas las indicaciones, el presuntivo y el diagnóstico final, y los medicamentos. Todo estaba en PDF, entonces tú ya fácilmente lo leías, ¿no? Ya no tienes que estar ahí preguntando a la farmacia misma, ¿no? En este caso, no pisé en farmacia, porque todo me lo llegó. Recordemos que mi caso ha sido una citas médicas por COVID. Entonces, en el año 2021 aún estaba el tema del temor, entonces todo fue delivery, ¿no? Eso fue la segunda. Ya en la tercera cita con la doctora, me dijo, bueno, ya pasaste los quince días, no sé, catorce, quince, dieciséis, catorce, quince, dieciséis, días, algo así ¿Cómo? ¿Has hecho fiebre? Le dije, bueno, yo me he tomado tortura, ya no, caraspera tampoco, tos tampoco tengo, saben bien que tengo el tema de problemas en la nariz, ¿no? Lujos, nada más, le digo, termina el retraso de la nariz y con eso retraso de algo. Entonces me dijo, y con eso me dijo, digo, ¿sabes qué? Necesito un descanso, me pongo un certificado, un descanso. Entonces me envió por correo también a mí, ¿no? Andando a la clínica, ¿no? Y me dijo, bueno, pues el punto comienzo sencillo, que decían, ¿no? Que había tenido covid, de tal a tal fecha, que he hecho tal procedimiento, y actualmente por la semiología de la enfermedad ya estoy apto, ya estoy bien, ¿no? Y eso también me gustó, porque fue muy rápido, o sea, eso es lo que a mí mucho me llamó y me gusta.

**E:**Ok, entonces, no es que se demoró, de repente tenías que hacer otra cola para que te hagan tu descanso médico, sino que te llegó, entiendo, a la hora.

**e:**Sí, sí, el mismo, sí, fue la... Yo las citas médicas, que bien recuerdo, siempre las sacaba temprano, siete u ocho. ¿Por qué yo? Porque yo digo, bueno, prefiero hacer una cita a primera hora, porque tengo yo, creo yo, en mi entender, que tengo el médico fresco, después del descanso, ¿no? No tengo fresco con días más claros, ¿no? Se rinde que se haga una cita a las cuatro de la tarde y ya está el doctor cansado, de repente no me va a prestar atención. Entonces yo siempre la sacaba en la mañana y a la tarda ya me llegaba el documento. Y entonces fue la carta con la doctora y, como digo, sí, fue muy positivo, muy, muy positivo el tema de que no tenía que ingresar correo, no tenía que reenviar un correo porque nunca me llegó algo o porque me faltó algo, no. Incluso la botica que me enviaron, me llamaron, porque le preguntaron si estoy bien con los medicamentos. Ojo, EPS no es un EPS premium, ¿ah? Ni tampoco es un EPS que tú lo pagas mensualmente y te cubre algunos gastos, ¿no? Porque entiendo que si fuera un EPS premium, no obviamente, pues no hay otro tipo de trato, pero en mi caso no. Es un EPS que nos cubre lo que la emergencia nos cubre, ¿no?

**E:**Ok, entiendo que esto, entonces la tecnología en este caso para ti resultó una facilidad principalmente porque, bueno, por un lado la tecnología y por otro lado también las características del médico que te atendió, ¿no? Por un lado, digamos, la tecnología al ser rápida, al permitirte estar más inmerso en la atención, o sea, recibir información clara, ¿no? La brevedad, la facilidad con la que puedes agendar tu cita, te evitas sintiendo colas, ¿no? Como cuando vas a un centro de salud para sacar una cita temprano, tienes que ir una hora y media antes, o sea, cola, estar ahí, ¿no? Entiendo que ahora no, simplemente fue por una aplicación y obviamente por las características del médico, digamos, entiendo que su forma de atender te permitió sentir escuchado, los detalles que tú le podías brindar los tomó en consideración. Pero ¿Qué habría que mejorar? ¿Qué consideras tú que habría de repente que puede mejorar? Esto quizá no me gustó tanto.

**e:**Con estas tres citas creo que cumplió mi expectativa. Con respecto a lo otro, por ejemplo, con la última cita que tuve que fue con un neumólogo, en otra clínica, no, miente, en la misma clínica, pero fue virtual, resulta que en esta última la experiencia no fue tan agradable porque ahí el médico, sí, le expliqué lo que estaba sintiendo, tenía realmente tos, no fue el año pasado, tenía tos, y me dijo, ¿Sabes qué? Hay que sacarte placas radiográficas. Ante lo posterior de los pulmones, vas a tomarse de tos jarabe y unos pastillas para chupar, ¿no? Entonces yo le dije, ¿Sabes qué, doctor? Yo el año pasado he tenido COVID, ¿no? Entonces me dijo, sí, está en la historia clínica, no te preocupes, pero no es eso. Así de cerrado. Y bueno, las placas radiográficas, con las placas tienes que personarte a la clínica a ver si el EPS te cubre. Y ahí fue un problema, ¿por qué? Porque yo asumía, mi esposo y yo asumíamos de que por medio de la aplicación del EPS, tú podrías ver qué clínica, sí, de qué porcentaje te cubre. Entonces en ese caso, no teníamos esa referencia, ahí sí tuvimos que llamar con el broker, con la ejecutiva que nos brindó este servicio, y a ella la llamamos. No respondió a tiempo, nos demoró dos días, tres días después, porque estaba preocupada, no sé. Y le dije, ¿Sabes qué? Digo, vas a sacar placas radiográficas, no sé si esto me va a cubrir el EPS o qué porcentaje, ¿no? Y ella fue la que gestionó, ¿Sabes qué? Si te cubre, te cubre un setenta y cinco por ciento, vas a comprar, vas a pagar la diferencia, solamente veinticinco, y es presencial. Ese fue un tema, ¿no? De que en este, evoluciones específicas, ¿no? En mi caso, en mi experiencia, con las placas radiográficas, no lo pude ver en la aplicación. Y cuando llamaba a la clínica, la clínica me decía, ¿no? Usted sabe, ¿no? Si hay una clínica, ni te responde, ni por un mail, whatsapp, ni mensaje, esto no sirve, es presencial. Pues yo soy un poquito, no me gustan esas cosas, no me gustan, no me gustan, me dan a esperar. Entonces, dije, ah, habló con el broker, y el broker me dijo que eso sí es bueno, entonces me tiene que solucionar. Entonces, ya no, no sé si estaba bien o estaba mal, pero la llamé, como te dije, y ella fue la que me solucionó. Tal clínica vas, me dice con tu número, con tu DNI, dice que tiene la EPS con tal aseguradora, y ya recuerda que vas a pagar solamente veinticinco por ciento. Si te dicen algo diferente, me llama, pero el broker, la aplicación no me brindó.

**E:**Entonces, ahí quizá allí lo que podía haber faltado era haber de repente definido bien qué cosas se podía hacer, ¿no? O sea, como que estos roles de, mira, en estos casos, con la aplicación no, pero al menos tener esa información a la mano, saber a quién sí. Porque en este caso, usted, digamos, tomó la iniciativa, ¿no? Porque no encontró por este canal telefónico, intentó de otro medio y no lo encontró, buscó otra alternativa, y así fue como solucionó. Pero esa solución, digamos, como que no fue mapeada, no le fue explícita a usted para saber cómo solucionar este caso, digamos, particular. Y por otro lado, entiendo de que, como parte de la experiencia, pero fuera del uso de tecnología, influye muchísimo la actitud de quien la está atendiendo.

**e:**Sí, bastante. Muchísimo es la actitud, ¿no? Porque la señorita de broker fácilmente, un pueblo dicho, sabes qué, no me compete a mí, ¿no? Soluciona, ya estás fuera de su alcance. Pero ella, gentilmente, me dijo, ya saben, yo te ayudo, ¿no? Y yo fue la que me indicó, al final ella fue la que me ayudó, ¿no? Y ya para luego por una resonancia magnética que le pegamos a mi esposa, en su rodilla, que fue este año, también la llamamos a ella. Y nos solucionó, ¿sabes qué? Tal si te cubre un mayor porcentaje, ¿no? Casi el 85% lo cubría. Ya, vamos ahí. Pero por ella, por la aplicación no se podía.

**E:**Ok, entiendo eso. Entonces le pregunto, si le dijeran que, digamos, hicieran la consulta de si volvería a recibir alguna atención de manera virtual, remota, ¿a usted le parecería bien? O quizá no.

**e:**Dependiendo de la evaluación, ¿no? Personalmente hablando. Yo creo que sí, ¿ah?

**E:**¿Por qué sí?

**e:**Sí, dale que sí. Sí, sí, sí. ¿Por qué? Porque uno, no sé si es una cosa correcta, pero no pierdo tiempo en trasladarme hasta la clínica. Ese tiempo lo puedo ahorrar y seguir trabajando o desayunar o descansar un poco. Otro, es que voy a estar en la clínica y voy a esperar. En la clínica tú llegas 20 minutos antes, 30 minutos antes para que nos atiendan, posiblemente no a tu hora, sino pasado unos 15, 20 minutos, que nos ha pasado. El día sábado me puedo usar a la compañía de la clínica, igual por la EPS, y la cita médica a las 4 y media y al final atendieron a las 5 y 10. ¿Por qué? Porque lo estoy subprocediendo anterior. Yo simplemente sí me gustaría que se atendiera a mí por la parte virtual, ¿no? A la manera que sea sentido, ¿no? Siempre cuando sea algo importante, más no algo urgente, ¿verdad?

**E:**Claro, claro. ¿Y por qué de repente, si quizás su respuesta hubiese sido no, quizás por qué no?

**e:**Ah ya, no, porque entiendo yo que el médico a veces tiene que hacer ciertos procedimientos que necesita obligatoriamente el paciente. Por ejemplo, no sé, problemas respiratorios tienen que ser las amígdalas, tener temperatura, el tema de los oídos. Si es un tema de presión o problemas de corazón, tienen que escuchar, ¿no? O de repente ahí mismo va a solicitar que hagan la prueba de presión alta o tema ocular también, ¿no? Pero creo que sí hay evaluaciones que se puede sobrellevar en forma virtual.

**E:**Claro, entiendo entonces que usted ingresaría a una evaluación, en general, mientras no es una urgencia, en general, mientras no requiere el problema de salud, para usted estaría bien una, digamos, atención virtual. Siempre y cuando obviamente las soluciones se puedan mapear, pueda ser explícito qué cosas sí se pueden hacer y qué cosas requeriría de repente un soporte de alguien, de una persona, ¿no?

**e:**Sí, o incluso, ¿no? El doctor me puede decir, ¿sabes qué, señor? A usted haga el... según lo que hemos detectado, posiblemente no tiene cierta enfermedad o cierta... Si tiene enfermedad, para descansar, sería bueno que te acepto sacar unas hojas para que te acerques a sacar sangre, radiografía, no sé, evaluación ocular, ¿no? Entonces, me voy, hago la prueba, ¿no? Porque es por la aplicación, bueno, me lo saco a la prueba y al no más, a los dos días, entiendo yo, tengo la cita también con el mismo médico y tiene esa cosa, me va a decir, ¿cierto? Y me envían medicamentos de libre. Es que el tema también es el tiempo, ¿no? Si falta todo eso es el tiempo, ¿no? De acuerdo, uno vive, incluso con mi esposa, de acuerdo, nosotros vivimos, buscamos que la clínica en la cual nos atendemos se cerra a donde trabajamos y donde vivimos. Ese se puede decir que es lo más importante, el tiempo que ahorramos.

**E:**Claro, si en caso de repente la tecnología, al contrario, no le ayudaría a ahorrar tiempo, sino más bien es difícil, le toma tiempo aprender cómo usar la tecnología.

**e:**Ah, no, me voy, ¿no?

**E:**Mejor, claro, ok, entiendo que para usted lo más valioso es la parte de optimización de tiempo que le brinda el uso de tecnología. Ok, perfecto. Bueno, eso sería todo por mi parte, —---. Gracias más bien por, justo por tu tiempo, por haber respondido estas preguntas, hay mucha información valiosa de tu parte. No sé si quisieras terminar comentando algo más, como para ir cerrando.

**e:**No, lo que sí podría indicar, ¿no? Es que si vas a hacer una aplicación, considerar mucho en la aplicación que no todos estamos inmersos en este mundo de la tecnología. Hay adultos, pacientes, adultos pediátricos que es complejo, ¿no? Y ahí es donde, posiblemente, también es un punto fuerte, ¿no? En mi caso, sé cómo, tengo cuarenta años, trabajo con las computadoras, las reuniones, pues también. Pero de repente, para un adulto mayor que vive solo, de repente es complejo. O de repente, para un familiar que va a tener a su hijo virtualmente y tiene el niño o la niña, o este adulto, discapacidad. También tiene que ser un poco amigable. Claro, claro, claro, entiendo. Ok, muchas gracias. Vamos a tomar todo esto en consideración. Y bueno, nuevamente reitero el agradecimiento de parte de todo el equipo por compartir un poco de tu experiencia. Y bueno, por mi parte eso sería todo.

**E:**Ya, muchas gracias y aquí estaremos terminando la reunión.

**e:**Listo, muchas gracias —---, un abrazo.

**D11: Usuario_Docente**

**E:**Ok, qué tal, buenas tardes. Estamos hoy entonces con —----, voy a agradecer que te puedas presentar, por favor.

**e:**Ya, buenas tardes. Mi nombre es —------.

**E:** ¿Tu número de DNI?

**e:**—----.

**E:**Ok, perfecto. Bueno, —-----, queríamos justo aprovechar esta oportunidad para poder consultarte algunas cosas respecto a tu experiencia con servicios de telesalud, con el servicio de atención remota. Cuéntanos, ¿cómo ha sido tu experiencia?

**e:**Bueno, yo, bueno, más ha sido en pandemia, por motivos de salud, sobre todo. Entonces, sí, sí, he tenido, no me puedo quejar, he tenido bastante suerte porque me han podido ver a través de las líneas y no, de verdad que yo no me puedo quejar. Esa ha sido mi experiencia que he tenido, ¿no? Tanto personal y familiar.

**E:**Ok, entiendo entonces que has tenido la atención por teléfono, ¿verdad?

**e:**Sí, sí, he tenido atención por teléfono.

**E:**Ok, ¿solo ha sido por teléfono o a través de algún otro medio?

**e:**No, no, no, ha sido presencial y también telefónicamente.

**E:**Pero ¿has tenido alguna atención, digamos, por Zoom?

**e:**No, no, no, no, no, no, no, solo por teléfono.

**E:**Ok, perfecto. ¿Cuántas veces más o menos te has atendido por teléfono?

**e:**Por teléfono, a ver, sin mentirle, habrá sido más de 30 veces. Más de 30 veces. Sí, ajá.

**E:**¿Todo correspondía a un mismo motivo de consulta o fueron diversas oportunidades?

**e:**No, diversas oportunidades, pero todas por salud.

**E:**Ok, ¿podrías comentarme, digamos, de alguna de alguna oportunidad, de alguno de estos, digamos, veces que acudiste a atenderte que de repente son más relevantes para ti?

**e:**Justamente cuando salí positivo con el virus, me mandaron reposo absoluto. Es el tiempo que estaban de 40 días que no podía salir. Entonces, las atenciones frecuentes han sido, ellos me han llamado para preguntarme cómo iba, cómo iba mi sistema, cómo estaba respondiendo con los medicamentos, si me hacía falta algún medicamento, los síntomas, y me enviaban los medicamentos. De verdad que yo agradecida, agradecida, de verdad. He tenido muy, muy buena suerte, se podría decir, ¿no? Porque yo he consultado a otros compañeros que no han tenido esa suerte, ¿no? Este porque no han recibido la atención que yo he tenido, ¿no? Pero yo sí, sí he tenido esa atención.

**E:**Ok, entonces, en este caso, por ejemplo, que me comentas, ¿no? Que recibiste la atención, ¿cómo fue? ¿Fue fácil acceder a este servicio?

**e:**Este, la verdad que ellos, como se llama, ellos han utilizado sus teléfonos particulares. Los doctores, por ejemplo, yo llamé, yo fui al sub y me atendieron, y ellos, los doctores mismos, para verme en seguimiento, ellos me han llamado a través de sus teléfonos, este, particulares, se podría decir, porque no eran fijos, no eran, y te decían, este, y tenían una hora, ¿no? Por ejemplo, te decían, hoy día te atendí, ya me dice, ya veo esto, este, te van a enviar esta medicina, y nos vemos de acá a dos días te llamo. Y yo esperaba, porque ellos eran dos en punto, y dos en punto te llamaban, o te decían, 9 de la mañana, 9 de la mañana te llamaban. De verdad que sí, yo tenía bastante control en ese aspecto.

**E:**Ok, entiendo que fue usado más para el seguimiento, pero tú, digamos, para poder acceder al servicio, entiendo que tú llamaste a una línea en particular.

**e:**Sí, sí, yo llamé, ajá.

**E:**¿Fue sencillo encontrar ese número?

**e:**No, no, no, o sea, no es que fue sencillo, sino que sonaba muy ocupado, ocupado, ocupado.

**E:**Ok, entonces, de alguna manera, digamos, la información de a dónde tenías que llamar, ¿no fue complicada? Lo complicado fue, digamos, que te conteste la línea.

**e:**Sí, ajá, que me contesten, pues, por la demanda, ¿no? Ajá.

**E:**Ok, tú llamaste entonces, ¿cuánto tiempo más o menos demoró en que alguien te conteste la llamada?

**e:**A ver, sinceramente, ajá, a ver, pasa que yo empecé temprano en la mañana y como no tenía, lo que voy a insistir, he dejado dos horas. A ver, ahora sí, pues tres horas, tres, cuatro horas, pero no, no, no continuo, ¿no?

**E:**Claro, o sea, ¿te tomó más de un día, más de dos días?

**e:**No, no, no, menos, menos, cuatro horas. Ajá. Así de la insistencia.

**E:**Ya, entonces, claro, entiendo que usted de alguna manera, de manera intermitente ha estado llamando.

**e:**Sí, ajá, ajá.

**E:**Ok, ok, está bien. Entonces, una vez que atendieron la llamada, digamos, ¿la atención sí fue más fluida o aún hubo ciertas dificultades?

**e:**No, no, no, no, me preguntaron los síntomas que tenía y más bien me derivaron, me derivaron al Essalud más cercana donde yo pertenecía para que me atendiera por emergencia.

**E:**Ok, entonces, usted llamó a la línea de Essalud.

**e:**La respuesta fue rapidita.

**E:**Usted llamó a la línea de Essalud.

**e:**Sí, a la línea de salud. Y me llamó y me fui y justamente pues había un, no sé si se ha salido, emergencia para el COVID. O sea, era una emergencia exclusivamente para lo que tiene síntomas de COVID.

**E:**Claro, ok. Y luego ya cuando usted regresó, retornó a su casa, ¿el seguimiento sí fue netamente por teléfono?

**e:**Sí, todo fue por teléfono. Todo, todo fue por teléfono.

**E:**¿Alguna de esas atenciones fue, digamos, en el área de salud mental? O sea, ¿algún profesional psicólogo o psiquiatra le llamó para hacerle seguimiento?

**e:**No, en ninguna oportunidad.

**E:**¿En ninguna oportunidad, esas atenciones remotas?

**e:**No, no, no, en ninguna oportunidad.

**E:**Ok, está bien. ¿Cómo calificaría, digamos, estas veces que usted, entiendo que para usted ha sido muy valiosa, importante, pero...

**e:**Sí.

**E:**Digamos, si usted le diera la opción de seguir atendiéndose de manera remota o atenderse de manera presencial, ¿usted qué preferiría?

**e:**Presencial.

**E:**¿Por qué?

**e:**Porque, porque es, este, te permiten examinarte, porque allá solamente, pues, este, cuando es control, cuando es por vía telefónica, este, solamente utiliza sus síntomas, no te pueden examinar, no te pueden ver las gargantas, no te pueden ver, este, tu respiración, o sea. Yo creo que en el tema de salud tiene que ser presencial, ¿no?

**E:**Ok, entonces, ¿depende mucho también del problema de salud, o en general todos los problemas de salud deben ser presenciales?

**e:**No, yo creo que, dependiendo, ¿no? Dependiendo.

**E:**Ok, ok. ¿Alguna vez usted ha ido, digamos, atenderse por algún problema de salud mental, o ha ido al psicólogo en alguna oportunidad?

**e:**Sí, sí.

**E:**Ok, si yo le hago la consulta, ¿usted llevaría las atenciones psicológicas, ¿usted qué preferiría, que sean virtuales o que sean presenciales?

**e:**Bueno, yo creo que, de las dos formas, porque ellas serían consultas. De una vez, yo creo que de una vez que nos den el diagnóstico, yo creo que, de las dos formas, ¿no? De las dos formas porque tanto vía Zoom, ¿no? Uno puede, ¿cómo le podría decir? Uno puede mirar, ¿no? O sea, puede conversar, este, de repente a través de una cámara, pero se puede ver, ¿no? Y presencial es lo mismo, porque yo creo que tanto los psicólogos o los psiquiatras nos ayudan a través de las conversaciones, nos tratan de ayudar a dar una mejor solución o guiarnos, ¿no? Eso es lo que hay.

**E:**Ok, entiendo entonces que debería ser como un mis, tanto lo uno como lo otro. Sí, sí. Pero debería ser, al inicio debería ser presencial o al inicio debería ser virtual o cómo debería ser, digamos. ¿Cuál sería su preferencia?

**e:**Yo creo que al inicio debería ser presencial.

**E:**¿Por qué?

**e:**Porque se supone que todavía no te dan un diagnóstico de lo que tú tienes que hacer, entonces te da un poco más de confianza y después ya de ahí el seguimiento o hago las siguientes entrevistas y ya puede ser virtual.

**E:**Ok, igual como la atención en salud, ¿no? Un diagnóstico presencial y un seguimiento.

**e:**Sí.

**E:**Ok, y en este caso usted mencionó que sería mejor por Zoom. ¿Cuál sería la diferencia para usted entre Zoom o por teléfono? ¿Por qué sí en salud mental con Zoom y por qué en salud en general por teléfono?

**e:**¿Por qué? Porque cuando uno, por psicología es diferente, ¿no? Porque en cirugía tú tienes que entrar en confianza con la persona y yo creo que, a través de mirarse cara a cara, yo creo que es mejor la interacción, ¿no? Para poder conversar, ¿no? Porque bien los problemas psicológicos o psiquiátricos son más netamente de confianza, ¿no?

**E:**Ok, entonces usted confiaría más en alguien que usted puede ver al momento de atenderlo.

**e:**Sí.

**E:**¿Les sería más difícil de repente poder confiar y contar sus cosas con alguien solamente por teléfono?

**e:**Sí.

**E:**Ok, entiendo. Está bien. ¿Qué otra cosa más usted cree que debería tener para que se sienta cómoda, para que se regrese o prefiera una atención virtual?

**e:**No, yo creo que, a ver, ¿qué podría ser? No, yo creo que como se llama algo, la seguridad que te da, ¿no? Siempre es este, siempre cuando uno conoce a alguien, bueno, eso me pasa a mí, uno siempre, o sea, al inicio uno tiene esa conexión, o sea, esa buena vibra. Entonces yo creo que por eso primero tiene que ser este presencial para que luego que entre con confianza puedan irse netamente al virtual, ¿no?

**E:**Ah, ok, entiendo, entiendo. Entonces para usted es muy importante la confianza con la persona que lo vaya a atender.

**e:**Sí.

**E:**¿De qué otras formas, digamos, la persona puede, digamos, tener su confianza, ¿no? Para poder atender.

**e:**Que sea seguro. La persona que te debe atender, que te transmite seguridad.

**E:**Debe transmitir seguridad.

**e:**Sí.

**E:**Digamos, con la forma de hablar, con sus gestos, ¿de qué manera?

**e:**Sí, claro. Con la forma de hablar y sus gestos. De las dos formas.

**E:**¿Qué más debería tener?

**e:**Yo creo que para mí es suficiente.

**E:**O sea, si usted, por ejemplo, se fuese a atender con algún profesional de salud, si usted ve que, bueno, en este caso un psicólogo, ve que es una persona segura y ve que con sus gestos puede demostrarse, denotar seguridad, para usted es suficiente. O se regresaría a atender.

**e:**Yo regresaría. Ok.

**E:**Entiendo que o bien presencial o bien por Zoom. Ok, está bien. Retomando un poco entonces su experiencia que ha tenido, ¿usted volvería a pasar por atención, digamos, virtual?

**e:**Sí. Sí, por la experiencia que he tenido, sí.

**E:**Pero en este caso tendría que ser, digamos, o sea, la línea al menos debería estar más ágil, ¿verdad?

**e:**Sí. Eso sí debería mejorar. Tendría que estar más ágil, sí. Eso sí. Y bueno, hasta ahorita, hasta ahorita, por ejemplo, para lo que es este, para sacar cita a especialistas, ¿no? Porque ahorita encuentras a medicina general, ¿no? Pero para encontrar especialista se demora un montón. Entonces la gente, la gente se amanece. Entonces, o insiste, insiste, insiste para lograr una cita. Eso es, bueno, hasta ahorita es el problema, ¿no? Creo que va a seguir.

**E:**Claro, entonces un mecanismo que ayude a optimizar el agendamiento de citas es lo que usted de repente considera que debería mejorar aún estos servicios de atención virtual, ¿no?

**e:**Sí. Debería mejorar.

**E:**Ok, está bien, entiendo. Bueno, estas serían las consultas por mi parte. No sé si de repente quisiera comentar algo más relacionado a qué podría mejorar, más sobre su experiencia o algunas otras sugerencias que le gustaría brindar.

**e:**No, no, creo que es todo.

**E:**Ok, voy a dejar entonces de grabar.

**e:**Gracias, —----. Ya, ok, gracias.

**D12: Usuario_Docente**

**E:**Muchas gracias profesor —----. Bueno, el día de hoy como ya nos ha podido dar un poco de su experiencia general que ha tenido con las evaluaciones en telesalud durante la pandemia, nos comentaba inicialmente hubo una respecto a una percepción de que podía haber tenido COVID y quería hacer un descarte y luego una segunda para el tema de efectos secundarios de la vacunación. Me gustaría saber cómo en manera general ha sido su experiencia accediendo a estos mecanismos de teleatención.

**e:**Ya, sí, como le decía, en primera instancia por los síntomas generales, después de haberme tratado en casa yo tomando algunas pastillitas y todo para el resfrío y todo y cuando ya se pasaron los días, dos, tres días, llamé a salud para la línea de, no, para ese entonces, la 113 me parece que era, entonces cuando llamé me respondió un médico y prácticamente me dijo los síntomas que tenía y todo, lo tuve que describir y me recetó unas pastillas para tres días, que tome algunas pastillas, que tome agua fría y todo, en ese momento muy bien, me sirvió y todo, pero después también me hicieron un seguimiento, me llamó el día siguiente una doctora casi en la noche me llamó, hasta el tercero, hasta el cuarto, pero resulta que después cuando ya volví a llamar, lo que yo hubiese querido es que se tenga ahí como una historia para que la persona que cuando uno llama nuevamente ya tenga la historia y para que sepan también qué es lo que nos ha dado, en el caso mío personalmente yo tenía una libretita y decía me han dado estas pastillas para los tres días, ya tomé, no, y que todavía me siento mal, tengo tanto de esto, no, le hacía el seguimiento porque cuando llamaba ya el personal que me atendía prácticamente no tenía creo, a mi parecer, parece que no tenía el seguimiento, le tenía que describir yo qué es lo que estaba consultando, tenía que repetir, sí esa, pero en realidad en ese momento no se me dio ninguna orden para el descarte del COVID y nada, yo prácticamente estaba sola en casa, estaba como desesperada, pero al final tuve que salir por propios medios a otro lugar para hacer menos descartes, eso es lo que yo.

**E:**En la primera instancia, cuando busca esta solicitud de atención por telesalud, le pidieron algún requisito, alguna afiliación a algún sistema de salud, sí, de salud o algún otro requisito para poder acceder a la consulta?

**e:**No, no, no, no me dijeron, me atendieron de manera directa, no, pese a que soy asegurada, pero no, no, no me mencionaron.

**E:**Me comentó que hubo un seguimiento, en total cuántas teleconsultas recibió?

**e:**Ah no, más de tres a cuatro porque me hicieron un seguimiento, otro seguimiento más creo, pero cuando ya continuaban los síntomas yo misma ya llamaba y sí me han hecho, me han respondido, me han respondido.

**E:**O sea, no es que hubo un seguimiento, sino que usted misma solicitaba la atención?

**e:**Claro, cuando ya, cuando no me pasaba, así es.

**E:**Considera de que esta cantidad de citas que al final tuvo fueron suficientes?

**e:**No, porque tuve que salir a otro lugar, no, porque como que se me complicaba, me sentía mal y al final también me dijeron que era estrés, que era este, no, que era estrés y tal, porque hasta llegué a que quería desmayarme, no.

**E:**Por el temor de todo el contexto de pandemia?

**e:**Claro, así es, así es.

**E:**En sí, de manera general, qué es lo que no le gustó de estas atenciones?

**e:**Lo que no me gustó es cuando preguntaba al doctor, no, que me dolía, tengo este síntoma, no sabía el seguimiento, nuevamente tenía que decirle y me trataban como si recién empezara.

**E:**Lo que nos comentaba, que no pareciera que hubiera una historia?

**e:**Ajá, ajá, eso es, eso es.

**E:**Hay algo más que le hubiera gustado que se fuera mejor o que no le gustó en la entrevista?

**e:**Ay, lo que me hubiese gustado es que, ya no importa, de repente que no nos llamen mañana o más tarde, no, pero sí el seguimiento, no, cuando uno se siente mal, ay ya, se le dio esto y que no, no se ha sentido bien con esto, no.

**E:**Ah, el seguimiento del tratamiento, ya.

**e:**Claro, el seguimiento, no, y estás bien, no, eso es lo que les quisiera, no.

**E:**Sí, me dice que sus tratamientos, pero me atuvieron 113 únicamente, no?

**e:**Sí, sí, sí.

**E:**A través de qué medio lo hizo? Telefónico? También tiene una línea de WhatsApp?

**e:**Sí, creo que lo hice por el... Telefono? Telefono, telefono.

**E:**Ah, ya, llamada.

**e:**Sí, llamada, llamada.

**E:**Bien, ya pasamos a la siguiente etapa. ¿Cuáles considera que son las barreras o los principales problemas que tienen las personas para recibir teleconsultas? Tal vez problemas con la conexión o para solicitar citas.

**e:**No, de repente, justo ahora que me dice, de repente la persona que recibe la teleconsulta, en realidad, de repente no entiende de salud o algo así, no? Y por eso, como que un poquito no lo tomará así como tan importante o algo, no? De repente la persona que recibe la llamada debe ser conocedora de la salud o algo, no? Como para que te derive, tengo este caso y todo para que te pueda derivar. Aunque a mí en esa oportunidad me derivaron, creo, a una enfermera, no sé.

**E:**Pero inicialmente le dio la impresión de que hubiera faltado más versatilidad en el triaje, como se le dice en la licencia.

**e:**Ajá, sí, sí.

**E:**Ok, y del otro lado, ¿qué considera que son los mayores beneficios o facilidades de las teleconsultas?

**e:**Hoy, si fuera una teleconsulta y te atendieran así prácticamente con un diagnóstico así, hoy nos facilitaría el tiempo, no? El tiempo y también nos ahorraría lo que es los, los, económicamente también, porque uno se traslada de un lugar a otro. Por ejemplo, yo estoy trabajando acá, pero estoy asegurada en otra, en otra. En otra red. Por ejemplo, en San Martín de Porres. Si yo tengo que ir a atenderme, tengo que trasladarme y casi necesito un día. Y por esto, por no perder el trabajo, por la responsabilidad que tenemos, uno deja de pasar. En mi caso, por ejemplo, no me atiendo porque me genera todo un día. Ajá, claro. Y para sacar cita, lo mismo, no? Para sacar cita, por decir, yo vivo al final de San Martín de Porres, casi por San Diego, pero mi centro de salud está al frente de la UNI. Entonces, y claro, y la cola, justamente cuando uno quiere sacar cita, me dijeron que vaya, va a haber los últimos días del mes, para decir qué día lo van a programar. Por decir, el primero nada más, programa, o el 31, ahí ponen un cronograma. Pero, ahí quienes ganan los cupos, los que prácticamente están a las 5 de la mañana. Yo, del extremo de San Martín de Porres, hasta donde me trato, prácticamente no llego a esa hora. Primero por la movilidad, y también es oscuro. La seguridad también. Estoy llegando a lo mucho a las 6 y media o 7, que ya prácticamente, ya la cola es inmensa y ya no alcanzo. Y me ha pasado varias veces. Y ahorita, por ejemplo, yo necesito atención en diferentes, este, ¿cómo se llama? Ahorita necesito salud. Por ejemplo, en las terapias. A producto de la vacuna también que me hicieron y todo, se quedó en una. Me programaron una más, y hasta ahora no puedo lograr porque no puedo acceder a las horarias. Pero con esta de las telecitas o teleconsultas, sí nos estarían programando. Y ya, presencial, ya igualito, ya vamos, ya tenemos el cupo. Correcto. Eso sí.

**E:**Bueno, nos ha comentado varias cosas muy interesantes de lo que debería tener una teleconsulta, ¿no? Como una historia clínica, la capacidad de poder sacar mis citas si es que necesito temas presenciales, ¿no? Como evaluaciones, tratamientos, terapias físicas o vacunaciones. ¿Considera que hay algo más que debería considerarse en un servicio de teleconsulta para que este sea más cómodo para usted? ¿Más agradable, o que usted sienta que es eficiente el tratamiento?

**e:**A ver, aparte del diagnóstico. Uy, sí, el seguimiento sería bueno, ¿no? Ah, la programación de seguimiento. El seguimiento. Y en los casos, sobre todo, ¿no? Esas personas que de repente de alguna manera tenían... Hasta ahora yo no sé si en realidad estuve con COVID o no, porque me hice una prueba antígena, no salió. Después me hice una rápida que no salió, de repente no fueron los días y no me salió nada, pero prácticamente yo me sentía bastante mal, ¿no? Para esos casos, por ejemplo, el apoyo psicológico. ¿No? El apoyo psicológico.

**E:**Se identifica en el profesional de salud si también hay un compañero psicológico que aprender.

**e:**Sí, sí, sí, eso es. A pesar de que no fuera la consulta inicial. Así es. Pero yo me quedé afectada. Yo prácticamente esa vez, una vez, me quedé afectada, porque casi me desmayaba, estaba sola, me daba miedo hasta tomar los taxis y todo. Pero tuve que salir porque ya no me quedaba yo.

**E:**Claro, lo entiendo. Bueno, profesora —-----, esta era nuestra última pregunta. Muy agradecido por su colaboración. A ustedes. En comprender estas experiencias de los usuarios en Telesalud.

**e:**Muchas gracias.

**D13: Usuario_Docente**

**e:**Totalmente abierto, usted puede acogerse.

**E:**Bien, profesora —---. Como le mencionaba, nuestro estudio busca conocer en esta etapa la experiencia en telesalud. Entonces, en manera general, ¿cuál ha sido su experiencia accediendo a servicios de telesalud?

**e:**Ya, bueno, en primer lugar, no he tenido ninguna comunicación por telesalud en cuanto a la salud mental. Y yo necesité, necesité bastante. Sí he tenido, porque yo soy hipertensa, entonces, llamé a Medicina General y ahí sí me respondieron. Me mandaron la facilidad de darme los medicamentos por la farmacia más cercana a mi casa, que le llaman la farmacia vecina. Entonces, yo me iba a recoger ahí. Cuando no podía salir, mandaba a una persona que también le podía dar con mi DNI. Y le daba. He estado los dos años así, porque cada mes me tienen que dar los medicamentos de la hipertensión. Después de otra especialidad, no he tenido.

**E:**Ok. Y ¿cómo ha sido su proceso para acceder a este servicio de telesalud? ¿Le pidieron algún requisito tal vez?

**e:**No, solicité, solicité con mi DNI. Me tardaron en darme casi como cinco o seis meses, pero me dieron. Me dieron la cita, hablé con el médico. El médico me preguntó cómo estaba, si necesitaba algo, si me encontraba. Me dio una interconsulta, sí, a salud mental, pero nunca la conseguí. En los dos años que he estado pidiendo, solicitando, no conseguí la interconsulta para mental.

**E:**¿A qué se debía que no se lograba conseguir?

**e:**No había cita, decían. No hay citas, no hay citas. No hay médicos, no hay especialistas. ¿Qué me decían? Que tenía que ser presencial. Que tenía que ser presencial. No lo atendía.

**E:**¿Dónde era que se atendía?

**e:**Yo me atiendo en Pablo Bermudez, en Essalud.

**E:**¿En Essalud?

**e:**Sí, en E-Salud.

**E:**¿Y ahí no tenían el servicio de su propia, en telesalud?

**e:**No tenía.

**E:**Ah, ok. No tenía. Entonces, ¿el único requisito tal vez para atenderle era que su afiliación a E-Salud?

**e:**Sí, mi afiliación y sí. Eso nomás.

**E:**Ok. ¿Cuántas teleconsultas has recibido?

**e:**En los dos años, tres casi, bastante. Ah, ok. Cada mes casi, o cada dos meses tenía como me...Indiqué que yo era hipertensa, entonces me daban ciertas preferencias. No tanto, pero sí, al menos me daban. Porque yo decía que necesitaba medicamentos y me daban la cita para que precisamente me den los medicamentos. Porque también me enfermé de la gastritis, me enfermé de otras cosas también que bueno, me daban, ¿no? Pero más era para la presión.

**E:**Ok. Y a través de qué medios recibía la teleconsulta?

**e:**Por teléfono.

**E:**¿Todos fueron teléfonos?

**e:**Todos por teléfono.

**E:**¿Qué le pareció que las afecciones hayan sido por este medio?

**e:**Sí, estaba bien. Sí, estaba bien por teléfono. A veces mi hija me ayudaba a hacer por internet. Me daban la cita, todo, pero no se cumplían.

**E:**Ah, no entraba el profesional.

**e:**No, no. Cuando yo esperaba que me dieran, que me comunicaran con alguien por internet o algo así. No, no, no. No se podía. No respondía el médico, no se enteraba, no lo sé. Pero era más directo por teléfono.

**E:**¿Ha tenido experiencias de atenciones de salud fuera de salud en este contexto de pandemia?

**e:**Sí, en MINSA me pusieron la inyección.

**E:**Ah, para la vacunación.

**e:**Sí, y también me llamaron ellos para preguntarme cómo estaba. Y era bueno porque yo no esperaba que alguien me preguntara cómo estaba. Y sí, me llamaban.

**E:**¿Y ahí sí tuvo una atención con un profesional de salud mental?

**e:**No, no llegué. No, fui al privado porque necesitaba...

**E:**¿Fue de manera presencial, privado?

**e:**Sí, sí, fue presencial.

**E:**Ahora ya un poco, centrándonos en las características del servicio de teletención que recibió, ¿cuáles consideran que han sido los principales problemas o barreras para que usted haya tenido una buena atención de teleatención, de salud?

**e:**Las barreras, no hay citas. No cuentan con citas. Y otra barrera, las interconsultas, no las hacen. O sea, yo consigo solamente para medicina general, pero para especialidad no las da.

**E:**Ah, ok.

**e:**O sea, a pesar de que el médico lo designe... A pesar de que el médico da la interconsulta.

**E:**Por falta de citas es que no logra.

**e:**Por falta de citas es que no se puede. Yo con él me dio para cardiología porque yo necesitaba una cita para cardiología, no me la dieron. Necesito una cita para ginecología, tampoco me la dieron. Ahorita necesito una cita para gastro, tampoco me la han dado.

**E:**A pesar de que ahora ya hay presencial.

**e:**A pesar de que hay presencial. Y menos en pandemia, nunca menos. Solamente lograba para medicina general.

**E:**Y desde el otro lado, ¿cuáles consideran que fueron los beneficios o los mayores facilitadores de haber utilizado servicios de telesalud?

**e:**Bueno, a distancia el teléfono fue un facilitador. Y bueno, las señoritas que siempre están atentas cuando llamamos, nos atienden. O sea, priorizan a las personas.

**E:**¿Les encargan de agendar cita?

**e:**Sí, sí. Priorizan, me parece, a las personas que son más mayores. Porque cuando yo era más joven no nos daban cita para nada.

**E:**Bueno, ya casi finalizando. Ya desde su percepción, su experiencia, ¿qué características debería tener un servicio de telesalud para que sea óptimo? Considerando todos los aspectos, desde sacar su cita hasta qué resultado final espera.

**e:**¿Qué esperaría? Más eficiencia, ¿no? O sea, coordinar mejor las citas, me parece. Y me parece que también más personal. Porque ellos dicen no hay personal, no hay médicos, no hay enfermeras, no hay... Eso es lo que nos disponen. Y hay tanta cantidad de gente que no se abastece. Entonces me parece que ese es el mayor problema. Aparte también hay medicamentos. No nos damos medicamentos. O sea, nos dicen para tal fecha, tampoco nos damos. Es un problema también. Un problema que hemos tenido. Y tenemos que recurrir al privado. Y no siendo pues, ¿no? Porque cuesta. El privado sí cuesta.

**E:**Tal vez, respecto a los resultados que esperan, tal vez hay algunos servicios que desearía que no fueran por telesalud. Que fueran de forma presencial.

**e:**Claro. Porque nos tienen que... De todas maneras, pienso que el servicio presencial es bueno siempre. Es mejor. Porque ahí los médicos antes, ¿sabes? Auscultaban. Por lo menos cuando yo iba al médico, por lo menos me medía la presión. Y veo que ahora ya no lo hacen tampoco. No. Ellos desde la pandemia... Nosotros estamos sentados aquí y ellos están tres metros allá. O sea, no sé. Ese es un problema también. A pesar de que es presencial. En el presencial ha cambiado. Y, ¿qué se llama? Y no se acercan a auscultarnos. O sea, cuando yo he ido varias veces con... Yo me enfermo de la... También a veces de la garganta. He estado con una tos terrible. Entonces, el médico antes me auscultaba el pulmón. Lo escuchaba acá. No lo hacen ahora. Ellos recetan por receta un poco. Han cambiado esas franjas. Cambiado.

**E:**Y en cuanto a servicios de telesalud, me dice que eso lo ha pasado por experiencia de... Por llamadas telefónicas.

**e:**Sí, por llamadas.

**E:**¿Pero estaría dispuesto? ¿Se sentiría más cómoda con un cara a cara? O sea, con una videollamada.

**e:**Ah, sí. Claro, con el médico. Sí. O psicólogo. Excelente.

**E:**¿O de otra profesión?

**e:**Excelente. Sería bueno.

**E:**¿Cree que existe alguna limitación para recibir algún tipo de videollamadas?

**e:**No creo. Acá en Lima no creo. Porque todos tenemos acceso a los celulares. A la computación. En cambio, me parece que eso va a ser un buen rollo en provincia. Porque hemos tenido ese problema nosotros. Cuando dictaban clases también. No tenían los mismos medios o comandos para tener. Ni siquiera un celular. Para poder hacer sus clases. En cambio en Lima, acá sí hemos tenido a varios niños que han hecho las clases a distancia.

**E:**Muy bien. Muy bien. Ok. Bien, —---, esa era nuestra última pregunta. Muy agradecido por su disponibilidad para comentarnos las experiencias. Nos ayuda bastante a entender las necesidades que hay de asegurar un sistema claro y fácil para sacar citas. Y que el seguimiento sea importante. Sí. Que no sea...

**e:**El seguimiento. Porque hay personas que tenemos enfermedades crónicas. O sea, al menos los mayores. Ustedes los jóvenes todavía no, pero los mayores sí. Ya tenemos hipertensión. Otras personas tienen azúcar en la sangre. Tienen un montón. Acá tenemos un montón de profesoras que tienen ese problema. No solo eso. Están del corazón. Y los medicamentos son mensuales. Todos los meses tienen que tomar eso. Años de años. Yo tomo años de años. Y medicamentos para la presión. Y tiene que haber un seguimiento. El médico ya debe saber, ¿no? En la historia. A ustedes ya saben. No descontinuar eso. Claro. El seguimiento. Y aparte de eso, antes, ahora ya no lo hacen. Me mandaban unas evaluaciones cada dos años del corazón. Porque la hipertensión malogra el corazón, malogra el riñón,  malogra la vista. Y otras cosas más. Entonces nos mandaban. Habían unos controles. Había, sí. Había una, este... ¿Cómo le digo? Especial. Una cita especial. No era cualquier cita. En la que todo el día le hacían ese control. Le sacaban sangre, le sacaban orina. Todo. La vista, el corazón. Me hacían ecografías del corazón. Todo. Nos hacían en un solo día. Y si no alcanzaba el día, al día siguiente. No necesitaba yo pedir cita. Un mes, dos meses, otra vez. Todo. Entonces ese control. Mandaban a mi médico tratante. Entonces me decían, no, la señora está bien de él. Y felizmente, gracias a Dios, he estado hasta hace poco, hasta antes de la pandemia. Mi corazón estaba bien, mi vista estaba bien, mi riñón estaba bien. Ahora después de la pandemia, yo ya no sé si estoy bien o no. A mí me dio, en la pandemia, me dio una ansiedad terrible, terrible. No podía, hasta cuando he venido acá al colegio he estado muy mal. Entonces con pastillas, estaba medicada. Ahora ya estoy bien. Poco a poco, poco a poco.

**E:**Bueno, nosotros con esta experiencia que nos dan nos permite crear esta plataforma. Que bueno. Esperamos tenerlo listo para septiembre. No va a llegar de repente al punto de hacer la terapia como final. Pero sí que pueda salir el trabajador ya con su informe de evaluación. Listo para la terapia que dice ahí. Claro. Para que no pierdan citas.

**e:**Y que sea oportuno. Exacto. Que sea oportuno. Que sea el momento en que les quede.

**E:**Y va a ser un horario amplio, ¿no? De seis de la mañana a once de la noche. Entonces en el horario que puedan, se ajena. Cuando ustedes ya puedan, van a poder responder también. Nosotros no vamos a decirles qué citas tenemos o qué horario tenemos para ustedes. Sino ustedes van a ver la lista y eligen el que les convenga.

**e:**Ya, qué lindo. ¿Quién están haciendo eso? ¿El MINSA o el Salud?

**E:** INS, somos el Instituto Nacional de Salud. Ya. ¿Y vamos a poder acceder a lo que estamos asegurados? Sí. No, no importa el seguro. Solamente ser de los sectores. Educación, salud o policía.

**e:** Ah, qué lindo.

**E:**Y justamente la siguiente semana también tenemos una actividad de difusión...

**D14: Usuario_Policia**

**E:**Hola, buenas tardes, señor —----. ¿Qué tal? Muchas gracias por aceptar esta entrevista. Vamos a proceder a realizar la entrevista, solamente para comentar que usted ya llenó su consentimiento informado previamente y también ha dado su consentimiento para la grabación de esta llamada. Ahora, las preguntas que le voy a realizar son preguntas que buscan recopilar un poco de su experiencia que ha tenido en cuanto al uso de los servicios de salud. La primera pregunta que me gustaría realizarle es justamente cómo ha sido su experiencia en el proceso de atención por todas las consultas que ha tenido, es decir, en cuanto a la búsqueda de la atención, al tratar y al ser atendido.

**e:**Bueno, en nuestro caso, por ser efectivo policial nos atendieron a la sanidad, ¿no? Nos desplazaron el COVID, nos dieron los tratamientos, bueno, en casa los pasamos a hacer tratamientos, ¿no?

**E:**Correcto. ¿Usted por ser efectivo policial entonces tenía un acceso, digamos, más rápido al tema de la atención?

**e:**La sanidad, ¿no? Nuestro seguro de la sanidad.

**E:**Exacto. ¿A usted le solicitaron algún requisito previo antes de brindarle las teleconsultas o atenciones, aparte de que usted ya ha sido, bueno, tiene su seguro en la sanidad, algún documento adicional le pidieron para ser atendido a través de las teleconsultas?

**e:**No, solamente su carnet, el carnet y el correo que tiene de nuevo.

**E:**De acuerdo. Ahora, ¿usted recordará cuántas teleconsultas ha recibido, por qué medios fueron estas atenciones, ya sea por llamadas, por otro...

**e:**Ha sido por llamadas, espero consultas por llamadas.

**E:**¿Cuántas más o menos habrá tenido usted, cuántas de este tipo de llamadas habrá recibido?

**e:**Unas tres, tres llamadas.

**E:**Estas llamadas se hicieron cuando usted, digamos, contrajo COVID y luego el personal de salud se contactó con usted para monitorear su estado, ¿es así?

**e:**No, en el tiempo del COVID uno se veía personalmente, veía los síntomas e incluso ingresaba en ese tiempo con un tubito por la nariz, por la garganta, creo, pero era presencial.

**E:**Eso fue para las tomas de pruebas, ¿correcto? Sí. Ok, y aparte, ¿a usted el personal lo llamó por teléfono para preguntarle cómo se sentía, cómo estaba yendo?

**e:**Ya eso nos llamaba cuando supervisaba mediante celular.

**E:**Ok, todo fue a través de celular.

**e:**Sí, ya nos veían cómo está, cómo se veía.

**E:**Correcto, y cuando lo llamaron, ¿qué le pareció el tipo de atención que le brindaba el personal? ¿Qué fue lo que más le gustó quizás en el tema de la atención, de la empatía? ¿Qué fue lo que más le agradó de ese tipo de monitoreo por llamadas?

**e:**Una señorita, usted por lo que le hizo la consulta, o sea, por lo que tomaba medicina y bueno, se me dio su receta y todo, y me respondió una medicina, que bueno, se hizo mejoría en ese tiempo.

**E:**¿En ese personal que digamos que usted lo llamaba, que lo monitoreaba, ¿solía ser el mismo, es decir, la misma señorita lo llamaba o eran diferentes personales?

**e:**Bueno, los tres llamadas seguidas fueron de las mismas personas, después ya llamaban diferentes, ¿no?

**E:**Ok, y en todo ese proceso, ellos le preguntaban por su estado de salud, monitoreaban sus signos, sus síntomas, digamos que la atención podría haber sido amable, empática, ¿usted la llamaría así?

**e:**Puede ser amable.

**E:**Ok, muy bien, y ahora ya hablando un poquito de quizás las barreras o facilidades que pudo haber se percibieron durante este proceso de atención, ¿cuáles diría que pudieron haber sido unas barreras principales? Es decir, por ejemplo, algún problema con el uso de la llamada, en la conectividad, quizás lo llamaba en un momento en el que no estaba disponible, ¿cuál fue, digamos, uno de los inconvenientes que pudo haber presentado?

**e:**Bueno, ya en el momento no había problema, porque como uno estaba en su domicilio, tenía, por mal, o sea, te llamaban a cualquier hora, ¿no?

**E:**Usted estaba disponible todo el día, digamos, para eso.

**e:**Claro, porque como usted estaba en su dormitorio, no podía salir, obvio que tenías ese tiempo para poder contestar, ¿no?

**E:**Ok, y en cuanto, bueno, ahora ya que es todo esa atención presencial, ¿usted diría que hubieron más beneficios o facilidades, quizás para las atenciones por teleconsulta en comparación a las atenciones presenciales? ¿Fue igual o usted vio alguna diferencia?

**e:**No, no fue igual, o sea, no. Solamente era para saber el estado, pues no, no.

**E:**Ok, digamos que había la misma posibilidad de obtener una atención virtual, digamos, una llamada, tanto como presencial.

**e:**Sí, eso puede ser, sí.

**E:**Ok, ahora una consulta ya un poco más proyectándonos quizás a un servicio de teleconsulta. Si usted tuviese la oportunidad de participar o hacer un servicio de teleconsulta o de teleatención, ¿Qué le gustaría más que tuviese este servicio para usted? ¿Qué le ayudaría o le haría a usted un buen usuario de este servicio?

**e:**Que haya para más, o sea, que, lo repito, sea para diferentes tipos de consulta, obviamente gastro, cosas ahí que después, porque en ese tiempo solamente había, tenía otras cosas, otras no había, pero solamente una especialidad o dos. Que haya diferentes especialidades, por supuesto.

**E:**Diferentes especialidades, entre ellas quizás, por ejemplo, el servicio de la atención en salud mental, quizás como un tipo de consejería para problemas quizás de ansiedad o depresión o estrés que puedan tener, también estaría interesado.

**e:**Claro, más o menos, si hubo, pues, algo de psicología, no, psicología, claro, pues.

**E:**Ok, y de participar en un servicio así, ¿exigiría algún horario de preferencia para usted en el cual podría participar o le gustaría hacer quizás sus sesiones de atención de psicología, quizás por la mañana, por la tarde o por la noche?

**e:**Por la tarde, digamos que su disponibilidad es más por la tarde. Sí. Ok, muy bien.

**E:**¿Algún otro comentario que le gustaría dar en cuanto a su experiencia, algo positivo o un comentario negativo en cuanto a lo que ha podido percibir?

**e:**No, no, no, no tengo nada. No, no, no, no.

**E:**Ok, muy bien. Muchas gracias señor —-----, hemos terminado con las preguntas, le agradezco la suma y su participación. Igual cualquier consulta, estamos disponibles por la página web.

**e:**Ya, muy bien, gracias.

**E:**Muchas gracias a usted, hasta luego.

**e:**Ya, hasta luego.

**D15: Usuario_Policia**

**E:**Buenas tardes, muchas gracias por haber aceptado la entrevista para el estudio que estamos llevando a cabo en el Instituto Nacional de Salud de CENSOPAS. Por favor, ¿nos podría decir su nombre completo y su profesión?

**e:**Mi nombre completo es —-----, mi DNI —----, mi profesión Policía Nacional del Perú, actualmente trabajo en lo que es en ese parte, en la Comisaría La Victoria Lima.

**E:**Muchísimas gracias. Para comenzar con la entrevista, le voy a hacer algunas preguntas relacionadas a su experiencia recibiendo alguna vez una teleatención. Dícese una teleatención como por ejemplo cuando uno recibe una atención de salud a través de un teléfono o de repente a través de una videollamada, otra vez de cualquier soporte tecnológico que no tenga una presencialidad. En ese aspecto, cuéntenos cuál ha sido su experiencia en estos tipos de servicios si alguna vez usted lo ha tenido. ¿Qué le pareció? ¿Cómo se sintió?

**e:**El 2020 justamente cuando comenzó la pandemia tuve experiencia porque me monitoreaban a raíz de que recibí el COVID en marzo del 2020 en principios que no se sabía absolutamente la atención para todos los pacientes. A raíz de eso la Policía Nacional en el hospital me detectaron que el COVID era positivo. Posteriormente me comenzaron a monitorear porque me enviaron a mi domicilio y me monitoreaban tanto telefónicamente, vía virtual también el Centro de Salud y también la Policía Nacional del Perú.

**E:**Y coméntenos, para poder realizar este tipo de atención que usted recibió en el contexto que nos cuenta, ¿le solicitaron algún tipo de requisito previamente antes de brindarles las teleconsultas?

**e:**No, solamente que esté pendiente tanto en la mañana, tarde y noche que siempre me monitoreaban porque aparte de eso en realidad yo tuve dos veces que obtuve ese virus. Fue en mayo que me duró hasta junio, 7 de junio que prácticamente me dieron el alta de monitoreo.

**E:**Y más o menos sacando un promedio, ¿cuántas teleconsultas cree usted que ha recibido en total?

**e:**En total exactamente no puedo aseverar porque me monitoreaban casi todos los días.

**E:**Correcto.

**e:**Todos los días me monitoreaban. Uno a veces me monitoreaba salud, al día siguiente me monitoreaba el personal encargado. En ese tiempo había una doctora psicóloga que me monitoreaba ella así constantemente e incluso en pleno tratamiento tuve una intoxicación. Más aún todavía la capitana psicóloga encargada del monitoreo constantemente me monitoreaba mañana, tarde y noche para ver mi estado de salud.

**E:**¿Y entonces nuevamente durante cuántos meses fue este proceso que le estuvieron haciendo seguimiento diario?

**e:**Abril, mayo, junio, tres meses.

**E:**Y qué es lo que más le gustó de haber recibido esta teleatención?

**e:**Lo que más era justamente que se aseveraban, o sea, el esfuerzo que hacían para poder saber cómo uno se encontraba.

**E:**Y por el contrario, qué es lo que no le gustó tanto?

**e:**Que, como decir, lógicamente que en ese tiempo por haber sufrido tanta gente, no era el único paciente. No, este en el caso mío, como decir, este, cuando obtuve yo la intoxicación, este solicitó la ambulancia que nunca llegó. Y eso fue en mayo, como decir, la primera semana de mayo sucedió eso con la intoxicación. Y en ese tiempo, en ese tiempo todavía, como digo, no había tanta oportunidad de ese tipo de servicio para que llegara la ambulancia, quizás por eso.

**E:**Correcto. Y sobre qué medio recibió esta teleconsulta? Por teléfono, computadora o una tablet?

**e:**No, vía teléfono, vía teléfono.

**E:**vía teléfono, directamente al número telefónico?

**e:**A mi número telefónico.

**E:**Y alguna vez usted ha recibido algún tipo de atención por Zoom o por Meet de repente? Esas plataformas de videollamada?

**e:**De videollamada no. De Zoom sí he recibido, pero ya casi al final de mi monitoreo.

**E:**Correcto. Y he encontrado alguna ventaja o desventaja entre hacer una teleatención por videollamada que por teléfono?

**e:**Lógicamente que es totalmente diferente porque uno ahí justamente ve a la persona, también nos ve, nos aprecia y se ve en realidad el estado emocional de salud que uno se encuentra.

**E:**Y dígame, en el proceso de estas teleatenciones, cuál cree usted que ha sido alguna barrera o problema que de repente usted haya tenido para al momento de recibir la atención? O si bien me cuenta que ha tenido monitoreo constante, ¿siente que hay algo que de repente era un poco difícil de sobrellevar al momento de ser, digamos, de recibir o de ser atendido por teleatención?

**e:**Lógicamente que hay veces, como vuelvo a repetir, en ese tiempo muchas veces nosotros mismos como pacientes nos encontramos emocionalmente mal, que no queríamos recibir la llamada. Pero nos sentíamos obligados a recepcionarla, estemos como estemos nuestra salud.

**E:**Correcto. ¿Y el uso de otros tipos de atención como las presenciales?

**e:**Lógicamente que ha servido mucho, porque como vuelvo a repetir, eso en sí, ahora que uno ya salió de esa etapa de salud, de ese tipo de enfermedad, ese medio valió de mucho, tanto telefónicamente como vía Zoom, valió de mucho porque como digo, veían el aspecto emocional de cada persona, del paciente, cómo uno se encontraba.

**E:**Correcto. Y si usted pudiera mejorar algo de este servicio, supongamos, en base a la experiencia que usted ha podido vivir, de repente para que el servicio de teleatención, por ejemplo de atención médica o atención psicológica, esté constantemente disponible a trabajadores como usted en la policía, ¿Qué tipo de cosas cree usted que deberían sumarse en este tipo de servicio de manera remota?

**e:**Que de repente actualmente usted ha visto que no tiene o que necesidades ha visto que se tienen que mejorar. Que den charla constante vía Zoom, no solamente para nosotros, como decir, en todo caso para todos los servidores públicos e incluso privados.

**E:**Y respecto de repente a la parte del servicio en sí, o sea al quedar, agendar, o de repente al uso mismo de la tecnología, ¿cree usted que hay algo que mejorar en ese aspecto?

**e:**Como digo, lógicamente, sobre todo buscar un medio, una hora prudencial para poder, tengan el tiempo suficiente las personas que pueden recibir este tipo de mensajes.

**E:**En su caso, usted tuvo que estar siempre pendiente de la llamada porque no sabía en cierta medida a qué hora le iban a llamar.

**e:**Claro, lógicamente, por eso hay veces, como digo, en ese tiempo uno se sentía, muchas veces la emoción no era de todos los días igual. Hay veces uno se sentía adolorido del cuerpo, el malestar que sentía, más aún estando completamente encerrado, entonces aislado. Entonces era un poco dificultoso y estar perenemente pendiente, uno lo veía de otra manera.

**E:**Ok, y si usted tuviera que de repente elegir entre una videollamada o una llamada telefónica como servicio de teleatención, ¿Cuál de usted preferiría para una atención así virtual o remota?

**e:**Como digo, para mí sería virtualmente vía Zoom.

**E:**Ah, vía Zoom, más que llamada telefónica.

**e:**Más que llamada telefónica. Porque aprecia uno a la persona y también nos aprecia.

**E:**Bueno, pues vamos a enviar información para que en el caso usted desee utilizar el servicio de manera gratuita, también lo pueda realizar. Y bueno, nuevamente gracias por su tiempo, señor —----. Hasta aquí, bueno, voy a poner stop en la llamada, perdón, en la llamada, no, en la grabación.

**D16: Usuario_Policia**

**E:** Le voy a comentar brevemente acerca de qué va el estudio antes de empezar las preguntas. Este estudio se llama Diseño y Evaluación de Usabilidad, Aceptabilidad y Satisfacción de un Servicio de Telesalud y de tamizaje, Manejo Inicial, Derivación Oportuna en Salud Mental para Trabajadores de Grupos Ocupacionales Vulnerables con Antecedentes de COVID-19. El objetivo de este estudio es diseñar y evaluar un servicio de telesalud. En este caso, nosotros estamos entrevistando a personas como usted para conocer un poco de su experiencia y de esa manera permitirnos a nosotros diseñar un nuevo servicio en las cuales vamos a poner en marcha en estos meses. Las preguntas, de hecho, y la información que usted nos va a brindar es netamente confidencial, así que solamente los investigadores van a poder acceder a esta grabación que vamos a realizar con usted. Y como le comenté, el único fin es poder desarrollar una propuesta de servicio que también luego vamos a compartir con usted en el caso de ese también poder asistir a las sesiones de psicoeducación, a los talleres virtuales que nosotros realizamos también para el personal policial, también dirigidos a personal de salud y docentes. Entonces, para confirmar su participación, ¿estaría de acuerdo en brindar su consentimiento en iniciar la entrevista?

**e:**Claro, sí, estoy a su disposición en la entrevista.

**E:**Correcto, muchas gracias. Para poder colocar sus nombres, ¿me podría dictar su nombre completo, por favor?

**e:**—-------.

**E:**Ok. Rufino Lobatón Llanos.

**e:**Ajá, correcto.

**E:**¿Me puede dictar, por favor, su número de su DNI?

**e:**—-----.

**E:**—--------- correcto. ¿Cuántos años tiene usted, señor —---?

**e:**70 años tengo. 70 años. Correcto.

**E:**¿Y tendrá alguna dirección de correo electrónico?

**e:**No, no tengo. Yo casi poco los conozco de eso. Ok, no se preocupe. Entonces, voy a guardar sus datos.

**E:**A ver, entonces, muy bien. Comenzamos entonces con la primera pregunta. Ya. Ok. Cuéntenos un poco cuál ha sido su experiencia en algún proceso de atención de teleconsulta que alguna vez usted ha tenido. En términos de teleconsulta, aquella sesión en que usted de repente llama por teléfono, de repente una videollamada con una red profesional de la salud, ¿Qué haya tenido? Cuéntenos un poco cómo fue su experiencia.

**e:**Bueno, con relación al COVID, ¿no? Eso fue en el mes de noviembre. Así que yo siempre salía a comprar, ¿no? Y en una de esas comencé a sentirme un poco mal, tosía bastante. Y yo soy atleta, amigo. Yo corro desde mi infancia hasta ahora corro yo. Y bueno, yo decía pues como soy atleta, tengo una alimentación especial, que yo casi soy vegetariano, amigo. Y así que, bueno, no le di importancia y poco a poco me iba poniendo mal. Tengo una hija que le comuniqué y me fui al hospital, pero no me interné ese día. Al día siguiente me seguía sintiendo mal y ya me internaron. Así que cuando me internaron vieron los médicos que yo me encontraba muy delicado de salud. Y la situación esta del haga, de los gases arteriales, yo estaba bien bajo, me quisieron entubar. Y así que estuve hasta el 14 de diciembre estuve yo internado, que ya me mejoré un poco y ya salí del hospital.

**E:**Ok, y cuéntanos en general, entiendo que usted tuvo en contacto con una teleatención cuando estuvo en este proceso de enfermedad. ¿Cómo siente que ha sido su experiencia? ¿Buena, mala, regular? En el proceso solamente de la teleatención que tuvo, ¿no? Cuando le hicieron el seguimiento por teléfono o usted solicitó una atención por teléfono.

**e:**Ya, mire, con relación a.… bueno, yo no estuve como... no, no, yo, a mí, mi hija me llevó al hospital, pero la atención ahí en el hospital, créanme que ha sido pues regular, porque mire, hay trabajadores, enfermeros, enfermeras, ¿no? Que son buenos, como también hay enfermeras que no, pues por ejemplo a mí me bañaban a las 12 de la noche, a las 1 de la mañana, a veces con agua fría, a veces con agua tibia, oiga, eso es lo que más me mortificó, yo me quería morir ahí. Sí, y.…Y yo me quería...

**E:**Claro, claro, y cuénteme un poquito, o sea, entiendo que de repente la atención en el hospital fue bastante, de repente, complicada, ¿no? En el tema de la atención presencial.

**e:**Mire, señor, con relación a la atención, mire, por ejemplo, venía la enfermera, solamente me ponía el antibiótico y de ahí no la veía hasta que cuando terminaba su turno, oiga. Con relación a los alimentos, oiga, que era la muerte, era porque me sacaban bastante sangre todos los días para hacer análisis, pero no me daban de comer. Yo le pedía a la enfermera, le decía que me dé comida, pero créame que la comida era bien escasa, bien poquita, yo me moría de hambre, oiga. Me moría de hambre, yo le decía que me den un pancito, que me den cualquier cosa, porque yo bajé como 20 kilos, 30 kilos, bajé, pero me puse huesito y pellejo que casi no muero, oiga.

**E:**¿Disculpe la pregunta, pero usted en qué hospital estuvo internado?

**e:**Yo estuve internado, primeramente, estuve en el Heysen, pero en el Heysen estuve poco tiempo, estuve más o menos, no, 3, 4 días y después me pasaron al Almanzor. Me faltaba el oxígeno, pero yo, allá en el Heysen me estaban poniendo 15 litros de oxígeno y allá en el Almanzor me pusieron 60 litros.

**E:**¿Entonces usted estuvo en una clínica privada?

**e:**No, no, no, en el hospital estuve yo, yo no estuve en clínica. Ah, ok. En el Almanzor.

**E:**Ok, este hospital, disculpe la ignorancia, ¿le pertenece de repente al sector policial o del ejército?

**e:**No, no, no, a Essalud señor, a Essalud.

**E:**Ah, a Essalud, correcto, correcto, correcto. Y en el proceso de atención también hicieron algún seguimiento por teléfono, por videollamada, así en remoto, ¿o sea desde su casa?

**e:**Cuando yo salí, sí señor, sí, sí, me llamaron, sí, pocas veces me llamaron, pero cuando me llamaban pues me decían, ¿cómo estás? ¡Paah!, me cortaba ya, ya no me llamaban ya.

**E:**Ok, y dígame, el proceso de, ¿en qué momento le llamaban? ¿Usted ya sabía a qué hora le iban a llamar? ¿O ya había tenido como que un agendamiento, digamos, para poder realizar esa atención?

**e:**No señor, no, no, no, no, no me decían, mira, ¿sabe qué? Lo llamo, lo estamos conversando ahora y aquí lo voy a llamar tal fecha. Y no me llamaban, pues no llamaban así a las quinientas, llamaban, oiga, y yo créame que ha demorado ya pues como casi dos años, oiga, y ya han quedado un poco con secuelas, pero gracias a lo que yo practico, a lo que yo corro todos los días, ¿no? Y ya me está un poco, ya se está yendo a las secuelas, oiga, por ejemplo, la garganta, los oídos, el sabor, un poco me afectan las vistas, oiga.

**E:**Sí, comprendo, fue complicado.

**e:**Sí, fue complicado. Sí, el excedente, ¿no?

**E:**Una consulta adicional, entiendo que de repente no fue tan ordenado el tema del seguimiento que tuvo usted por teléfono, pero ¿cree que esas llamadas que le hicieron le ayudaron en algo, en su tratamiento, en su recuperación?

**e:**Créame señor, de que me llamaban por compromiso nomás, nada más, me llamaban por compromiso y como está, me cortaban ya, me llamaron, según ellos ya me llamaron, pero no, pues no, no, no, no, no ha sido una llamada pues que se han avisado, ¿no? Como usted, por ejemplo, usted me dice, a tal hora lo llamo y me ha llamado, usted está atento conmigo, ¿no? Bacán, ¿no? Yo estuve en un bus, señor, yo estuve en un bus, pero usted dame que hasta el curita me iba a contestar todos los días, oiga.

**E:**Sí, lo comprendo. Y dígame, si usted pudiera, por ejemplo, si usted estuviera en la decisión de poder decirles al personal de salud cómo realizar el seguimiento de llamadas, ¿cómo a usted le hubiera gustado que fueran estas llamadas? ¿O qué cosas le hubiera gustado que le hubiera facilitado por medio de esta comunicación así en remoto para que pudiera mejorar su tratamiento?

**e:**Mire, a mí me gustaría pues que siempre comunique, ¿no? Para uno estar atento a la hora que lo van a llamar, ¿no? Y créame que vengan a verlo a uno cómo se encuentra, ¿no? Y porque esas cosas pues siempre lo orientan a… más que todo uno queda psicológicamente un poco mal, amigo, porque créame que yo he vuelto a nacer, oiga.

**E:**Así es, sí. ¿Cree usted que, si los mensajes fueran brindados de, digamos, de una manera como que más empática, entiendo que no lo fueron con usted, le ayudaría muchísimo a quizás a tener una mejor recuperación o de repente recibir un consejo que realmente le ayude?

**e:**Por supuesto que sí, claro. Ustedes son de salud, ustedes saben cómo uno se debe recuperar lo más pronto posible, ¿no? Pero si a uno lo llaman por compromiso, por decir, el señor, cuéntenos a todos, ¿Cómo está? Y ahí nunca lo cortan a uno. A mí me han llamado como dos o tres veces, nada más.

**E:**Y cuénteme, señor José, actualmente, bueno, existe además del teléfono, de repente usted lo sabe, otros medios, ¿no? Para poder realizar también una teleatención remota desde su casa, ¿no? A veces por la computadora, a veces por el mismo teléfono, está la videollamada, ¿no? Se pone la cámara y se conversa u otras veces la misma llamada telefónica como la que tenemos ahora. ¿Cuál de todas las formas, digamos, tecnológicas cree usted que le ayudaría mejor en hacer un seguimiento? Le pongo un ejemplo. Algunas personas les gustan las videollamadas, o sea, ver a la otra persona detrás de la pantalla, a veces en el teléfono o en la computadora, porque creen que se puede comunicar mejor, ver las expresiones, ¿no? Hay otras personas que de repente les es más sencillo, más fácil, simplemente la llamada telefónica.

**e:**La llamada telefónica para mí sería mejor, ¿no? Pero si sería pues una videollamada también, sería bien, sería bien. Pero que pongan, que pongan, que pongan esos señores de salud, que pongan mucho empeño, mucha preocupación en nosotros. Bueno, créanme mi deuda, mi amigo. Yo no, yo no, yo ahorita estoy contestándole la llamada, amigo, este celular es de mi esposa. A veces ella sale, sale y ya por cualquier cosa yo me quedo sin comunicación. Si usted me llama, créanme que yo no le voy a responder. Pero si usted me dice a tal hora, a tal hora lo voy a llamar, yo le digo a mi esposa que no salga porque usted me va a llamar.

**E:**Correcto, claro. Para usted obviamente el quedar y tener una agenda ya preprogramada es muy importante, ¿no? Para que pueda tener ordenado.

**e:**Por supuesto, sí, sí. Así es. Yo le estoy respondiendo a usted, ¿no? Y le he dicho a mi esposa que no salga porque el papá de la señora Borja es mi promoción. Y él me ha pedido que yo acepte la llamada, por eso estoy conversando con usted.

**E:**Sí, nuevamente, señor José, le agradezco muchísimo poder escucharlo y que nos cuente su experiencia porque nos va a ayudar a nosotros a tener una mejor forma de ver las necesidades de personas también como usted, ¿no? Para poder brindar un servicio de teleatención y hasta el momento lo que he podido escuchar es que efectivamente si fallamos en el agendamiento, como usted dice, es algo que no va a garantizar un servicio como el que usted desee. Me gustaría también terminar con unas pocas preguntas para no quitarle mucho más tiempo. En relación justamente a las teleconsultas, ¿no? Mire, le pongo el contexto, un ejemplo. Muchas veces hacer colas o ir a un hospital genera mucho tiempo, ¿no? Sacar una cita, atenderse con un médico de manera presencial es todo un trámite, ¿no? A veces la llamada telefónica, digamos, o la teleatención que le llamamos nosotros, ¿no? Que es estar en la videollamada o la llamada telefónica, quedar en un agendamiento, digamos, o tener una cita que uno puede reservar por la computadora. En algunos casos podría ser rápido. Dígame usted, entre las dos opciones, una atención presencial y una atención remota, en su caso, ¿no?, en su caso en particular, ¿cuál de los dos cree usted que le resultaría mucho más fácil para poder acceder a un servicio de salud?

**e:**Bueno, la presencial, ¿no?, porque uno conversa con un médico, ¿no? Pero en la situación de la presencial, pues, uno tiene que levantarse a las 4 de la mañana. Yo no vivo en Chiclayo, yo vivo a 7 kilómetros de Chiclayo. Hay que levantarse a las 4 de la mañana para ir a hacer su cola, ¿no?

**E:**Sí. En ese caso, una atención presencial no le resultaría ningún problema, ¿versus una atención virtual, quizás que sí?

**e:**Claro, sí, sí.

**E:**Y en el caso tuviera la opción de una atención virtual en vez de una presencial, ¿la tomaría o no?, ¿o siempre elegiría la presencial?

**e:**Bueno, sería mejor la presencial, porque uno conversa con el médico, pues.

**E:**Correcto, correcto. Bueno, señor —----, yo creo que hasta aquí hemos culminado con varias preguntas. Le agradezco muchísimo que me haya podido brindar su opinión. Así que yo voy a finalizar la grabación en estos momentos.

**D17: Usuario_Policia**

**E:**Buenas noches, señor —---. ¿Qué tal? Vamos a realizar la entrevista, pero antes, por favor, nos pediría que dijera su nombre completo.

**e:**Mi nombre completo es José Luis Rosales Baca. Muy buenas noches, señor —- -.

**E:**Gracias por aceptar participar de esta entrevista. Esta entrevista está en el marco del proyecto de diseño y evaluación de usabilidad, aceptabilidad y satisfacción de un servicio de telesalud de tamizaje, manejo inicial y derivación oportuna en salud mental para trabajadores de grupos ocupacionales vulnerables con antecedentes de COVID-19 en Lima. Para comentar, el señor —----- ya ha llenado previamente el consentimiento informado y también ha aceptado la grabación de esta llamada solo con fines de investigación. Buenas noches, señor —---. A continuación, voy a proceder a hacerle unas cuantas preguntas.

**e:**Sí, claro, señorita, continúa.

**E:**Ok, señor —-----, ¿usted en qué contexto hizo uso del servicio de telesalud? ¿Usted tuvo COVID anteriormente? ¿Fue monitoreado o fue por alguna otra razón?

**e:**Sí, señorita, yo tuve COVID. Yo he tenido el año pasado, ya casi finalizando la pandemia, tuve dos semanas con descanso domiciliario. Me monitorearon por medio de videollamada y por medio de una llamada telefónica del centro de salud de la sanidad PNP. Estuvieron monitoreando para ver cómo iba mi evaluación en ese proceso. Correcto.

**E:**Estas atenciones que usted recibió, ¿a través de qué medios? ¿Fueron llamadas por WhatsApp, reuniones por Zoom o por Meet? ¿Cómo fue ese proceso?

**e:**No, solo mediante llamadas del centro de salud de la sanidad PNP. Todo era llamadas. Nos indicaban qué es lo que debíamos tomar, qué síntomas teníamos, qué mejoras habíamos presentado durante el día o cuál transcurriría los días. Nos indicaban también con respecto a la temperatura, si habíamos tenido fiebre, cuánto habíamos hecho de fiebre y cositas así.

**E:**Estas llamadas o teleconsultas que usted recibía, ¿con qué frecuencia se daban? ¿Usted recuerda cuántas de estas teleconsultas o teleatenciones se había recibido?

**e:**Bueno, en la semana dos veces por lo menos. De las dos semanas que estuve con descanso domiciliario, por cada semana me hubieran llamado dos veces. En total, habrán sido cuatro llamadas. Cuatro llamadas.

**E:**Usted me comentó que se atiende en el centro de salud de la sanidad policial. ¿Había algún requisito o le pedían algún documento previo para que usted pudiera recibir estas atenciones?

**e:**Sí. ¿Qué pasa? Que nosotros, para que nos emitan el descanso médico domiciliario, teníamos que ser atendidos físicamente por medio de la sanidad. Tendríamos que haber concurrido para la atención de vida. Posteriormente nos emiten el descanso domiciliario y ellos llevan un control. Con ese control, te llamaban, te identificaban por medio de la llamada y posteriormente te comenzaban a atender o a dar las consultas que tú requerías o las cositas que de repente tú las consultabas.

**E:**O sea, exigían, digamos, que primero sea algo presencial y después ya todas las atenciones podían ser por llamada.

**e:**Sí, en el caso únicamente de COVID.

**E:**Solamente por COVID. ¿Alguna vez le pidieron algún documento, presentar algo como DNI, algún otro documento, una póliza, seguro, algo adicional o solamente era ir presencial?

**e:**No, solamente me pidieron nada más que brinden mis datos, mi número de carnet policial, mi número de DNI y hay una foto que en una ocasión recuerdo haber enviado a pedido de la sanidad.

**E:**Ok. Digamos, para recibir ustedes las teleatenciones, ¿eran inmediatas? ¿Usted tenía que sacar cita, concretar primero con el personal de salud o ellos lo llamaban a usted directamente? ¿Cómo era ese proceso?

**e:**Recuerdo que los primeros siete días que me dieron de descanso médico, yo presenté los síntomas, fui a atenderme a la sanidad, fui atendido, me hicieron mi descarte. Posteriormente me dieron los resultados a los 20 minutos de haber sido atendido y luego de haberme dado el resultado positivo, me entregaron mi descanso médico para mi descanso domiciliario por siete días. Entonces, luego de haber concluido los siete días, tuve que nuevamente ir a la sanidad para que me renueven ese descanso médico por siete días más.

**E:**Ok. Entonces, era así más o menos el flujo. En algún momento cuando ellos lo llamaron, ¿había un horario específico en el cual lo contactaban o podía ser a cualquier horario del día?

**e:**No, era durante el día, pero era inopinado, las llamadas no eran en un horario fijo.

**E:**Era digamos más según la disponibilidad de ellos que lo llamaban.

**e:**Al parecer sí.

**E:**Y bueno, durante ese periodo usted estaba en casa, probablemente con su familia, así que estaba digamos más disponible para ser atendido.

**e:**Sí, bueno, yo estaba en casa, pero ahí al lado de mi familia estaba en mi cuarto y podía haber atendido en ese momento las llamadas, ¿no? En su mayoría. En las dos ocasiones que me llamaban en las semanas, sí le contestaba normalmente sin ningún problema.

**E:**Y posterior a ese proceso, digamos ya desde la atención por COVID, quizás usted tuvo algún monitoreo posterior, o sea ya no estando de descanso médico, sino digamos volviendo a sus labores, ellos siguieron contactándose con usted quizás para ver cómo seguía evolucionando o ya no persistió este monitoreo.

**e:**Si no me acuerdo, señorita, fue una vez que me volvieron a llamar cuando yo ya estaba laburando, había regresado nuevamente a trabajar y me llamaron de la sanidad para ver cómo seguía. Fue una única vez, nada más.

**E:**Solo una única vez. Entonces más o menos cuatro o cinco sesiones o teleatenciones que habrá recibido en total.

**e:**Exacto.

**E:**Ok, muy bien. Y de todo ese proceso de teleatenciones, llamadas que usted ha recibido, digamos, ¿cuál habrá sido la parte que más le gustó de haber recibido esa atención por teleconsulta?

**e:**Bueno, de la experiencia de las llamadas, recuerdo que me indicaban cómo iba con respecto al tema de síntomas, se preocupaban bastante en ese aspecto y me preguntaban qué es lo que yo estaba tomando, si estaba siguiendo a pie de la letra lo prescrito por el doctor. Que en ese caso no debía tomar o automedicarme, sino tenía que seguir a pie de la letra lo que me habían recomendado, que era solamente el paracetamol en ese entonces, nada más.

**E:** Ok, entonces le gustó, digamos, el tipo de atención, quizás la preocupación, podríamos decir quizás la calidez humana con la cual lo atendieron.

**e:**Sí, sí, sí, podría decir que sí, porque me atendieron en todo momento de buena manera, con buen trato y sobre todo se notaba la preocupación por mi persona.

**E:**Se mostraba el interés en su mejoría. Sí, correcto. Y digamos, viéndolo ya por el otro lado, ¿Cuál sería la parte quizás que no le agradó o no se sintió muy cómodo quizás al ser atendido mediante este servicio? ¿Cuál fue la parte menos agradable, digamos?

**e:**Menos agradable de repente la renovación del descanso médico, pues no, porque nosotros estando todavía convalecientes por el tema del COVID, creo yo que hubiese sido mejor renovarlo de otra manera, sin necesidad de que nosotros vayamos físicamente, presencialmente a la sanidad, puesto que todavía podíamos infectar a otras personas.

**E:**Ok, y en cuanto quizás a los horarios de atención, a la disponibilidad de personal, quizás a usted en algún momento le hubiese gustado usted también poder contactar libremente con alguno de los profesionales de la salud en caso de tener alguna duda, quizás le hubiese gustado eso.

**e:**Sí, claro, no me hubiese gustado que me digan ustedes en el momento que de repente se siente mal o siente que requiere la atención de un personal especializado, usted puede llamar este número y directamente le va a atender tal persona o tal doctor, pero no sucede eso.

**E:**Quizás como que faltó un poquito brindarles, digamos, más canales de atención o estar más disponibles para los pacientes, quizás.

**e:**Claro, porque nosotros únicamente, para ser sincero, no seguíamos al pie de la letra lo que era nada más el paracetamol, ¿no?, ¿por qué?, porque por eso estaba nuestra familia en casa, nuestra esposa en casa, para que nos suministraba, por ejemplo, una bebida casera, de repente, bebidas calientes, una sopita bien caliente, tomar, hace poco, bastante kion, o de repente por ahí un vaso de limón. Con bastante ajo en algunas ocasiones, ¿no? Claro, que se puede recibir. Entonces era el método de cuidado en casa, aunque nos ayudaba de cierta manera a tener una mejoría.

**E:**Quizás tuviera mayor orientación, ¿no?, mayor suporte.

**e:**Claro, en mi caso no fue tan fuerte, pero en el caso de otros colegas, tuvieron que estar internados y la pasaron un poquito más complicado, ¿no? Pero yo creo que, si hubiesen tenido una mejor atención o hubiera, ellos, tenido conocimiento de que de repente tenía disponibilidad una persona que los atendiera en caso de una emergencia o que había una disponibilidad de una ambulancia a su disposición cuando ellos lo requiriesen. Así no sea verdad, pero eso en el tema psicológico influye mucho.

**E:**Exacto, claro. Y justamente por el tema psicológico, usted, bueno, también puedo suponer que quizás se sintió afectado emocionalmente, quizás un poco de estrés o ansiedad por lo que podía pasar, por lo que podría ocurrir. En ese sentido, quizás hubo algún profesional de salud mental que lo pudo llamar o lo pudo atender durante este proceso, porque, bueno, si bien no todo era físico, un malestar físico, también quizás había algo de preocupación y salud emocional y mental. ¿Tuvo esa oportunidad de poder contactar con algún profesional de salud mental o alguien que le haya brindado soporte emocional?

**e:**La verdad no, si bien en aspecto laboral, en el aspecto de mi trabajo, de personal de salud especializado, como es lo psicólogos, no, pero sí de un familiar que es psicóloga, me llamó y me orientó medio como que ese apoyo, suporte emocional que ellos necesitaban en ese momento, porque yo lo primero que tenía en la cabeza era que me podía pasar algo, o tenía miedo de infectar a mi familia, a mi hogar, entonces tenía ese temor, ese temor que no quería que nadie se me acerque porque tenía miedo de infectarlos.

**E:**El temor también de quizás poder ocasionar daño a nuestros familiares, a nuestros seres queridos.

**e:**Sí, exacto, ese es lo más triste, emocionalmente te deprime esa situación, el saber que podés contagiar a tus hijos, a tu familia, que está en tu entorno, que vive contigo, eso psicológicamente a uno le afecta bastante.

**E:**Y justamente, hablando de eso, a la parte de salud mental y emocional también, si usted tuviese la oportunidad de participar en un programa de teleatención ya de salud mental, un poco más dirigido a este aspecto, ¿qué le gustaría que tuviese este programa, o qué le gustaría, en cuanto a facilidades que le brindase a este programa, ¿cómo le gustaría que fuese dado?

**e:**¿Con el tema de COVID, específicamente?

**E:**Sí, el tema de salud mental, emocional, porque bueno, si bien ahora ya el COVID no está tan fuerte, quizás un poco más el tema de la ansiedad o el estrés que vivimos día a día, ¿no?

**e:**Claro, me parece, eso es fundamental, creo yo, fundamental, no solamente por el hecho de que seamos felices, sino fundamental para la persona, porque la recarga laboral es una, la recarga personal, me refiero a los problemas que cada quien pueda tener, eso hace que llegue un momento en que la persona se siente estresada, ansiosa, y a veces te bloqueas, no puedes hacer tus cosas como deberían ser, por eso no creo que yo, y pasan tantas enfermedades, por ejemplo, hay personas que no, pueden tener un derrame, ¿no? Con una parálisis facial, producto de repente la recarga de problemas que puedan tener, la cabeza.

**E:**Claro, entonces sí sería importante, bueno, le gustaría, muy importante poder tener ese soporte, ¿no?

**e:**Porque me imagino yo, no puedo tener un mal día, y si yo no tengo la capacidad para poder solucionarlo en lo personal, sabiendo que yo no puedo traer ese problema de trabajo a mi hogar, si yo no sé solucionar eso, no sé separar eso, para eso de repente hay una persona que te puede ayudar, ¿no? Diga, mira, ¿sabes qué? Estamos acá para apoyarte, para ayudarte a poder ayudarte a responder sobre un problema que esté pasando, puedes contar con nosotros, ¿no?

**E:**Claro, y ahí también hay que usted pueda contactar, también pueda buscar apoyo.

**e:**Claro, ¿no? Mira, no sabemos si tienes niños en casa, si tienes una familia o no, pero tus niños, mira, tienes que saber y tener presente que tus niños, tu esposo, tu familia en casa no tienen nada que ver, son muy ajenos a los problemas que puedan tener en casa, ¿no? De repente tus hijos esperan un abrazo tuyo, tu esposo también, hay que ir con una buena actitud, los niños siempre esperan eso, tú también has sido niño, pasas perfectamente, entonces yo creo que eso es muy, muy importante. No, porque hay muchas personas, conozco, que llegan con todos los problemas del trabajo y con alguna familia, con hijos, cuando no tienen nada que ver los niños también.

**E:**Claro que sí. Ahora, en cuanto al uso de esos servicios ya de telesalud mental, digamos, que se pudiesen proponer, ¿a usted le gustaría que fuese por medio de llamadas? Preferiría que fuese quizás una reunión virtual por Zoom o por Meet, ¿cuál sería, digamos, su medio más factible, hablando de virtualidad?

**e:**Yo me siento más en confianza por este medio quizás, ¿no? Hablando por teléfono, por medio de una llamada, pero tampoco soy ajeno a poder realizar una videollamada o una consulta por medio de Zoom, o sea, no soy ajeno a eso, pero siempre, para mí creo que eso tiene que ser preparado, porque a veces decir, o que te llamen de un momento a otro, puedes estar de repente fuera de tu domicilio, puedes estar en el carro, puedes estar en otro lado, pues yo creo que para hacer una videollamada hay que estar preparado o coordinarlo, ¿no? Pero una llamada tú le puedes responder a cualquier persona, ¿no? Donde estés.

**E:**Si tuviese, digamos, la posibilidad de elegir horarios de atención, quizás en los que usted pudiese ya coordinar sus citas, ¿a usted le gustaría que fuese en la mañana, en la tarde, por la noche, digamos, ¿cuál sería su mayor disponibilidad?

**e:**Por mi horario de trabajo, en estos horarios, en este horario que usted me llamó, para mí sería limitado, porque yo tengo una labor administrativa dentro de la unidad, entonces yo trabajo todos los días, en horarios de 8 horas.

**E:**Ok, entonces la noche, así tarde, noche, 6, a partir de las 6 de la tarde sería ideal para usted.

**e:**A partir de las 7, 8 de la noche sería ideal para mí, porque yo ya estoy en casa, estoy tranquilo, de repente ya soné, tengo disponibilidad aquí.

**E:**Y claro, y también ya puede estar quizás más cómodo para poder llamar, ¿no?, un lugar tranquilo, en lugar, en la calle.

**e:**Claro, ya estoy en casa.

**E:**Para que tenga buena conectividad también, quizás.

**e:**Claro, claro, señorita. Eso es importante, creo.

**E:**Muy bien, quizás durante el tiempo que, a usted, bueno, le estuvieron haciendo el telemonitoreo, la teleconsulta por parte del hospital, ¿tuvo algún problema con el tema de las llamadas? ¿Se le entrecortaba? ¿Por ratos entraba la señal? ¿Hubo alguno de estos problemas?

**e:**Yo podía contestar la llamada normalmente, cuando ellos me han llamado, pero de repente me llamaron en una ocasión cuando quizás creo que estaba almorzando, finalmente me equivoco, entonces tuve que dejar almorzar para poder atender mejor la llamada. Pero después problemas de conexión en la llamada, no.

**E:**Muy bien, entonces veo que a usted más quizás el tema de avisar o coordinar previamente sería bueno también para que no interrumpa sus otras actividades.

**e:**Claro, yo digo, siempre con previa coordinación uno se realiza mejor.

**E:**Y volviendo al tema del programa de teleconsulta, ¿qué podría tener ese programa para que usted se sienta más cómodo, no, durante la teleconsulta, durante el uso del servicio?

**e:**Yo creo que la dinámica es importante. El hecho de interactuar con la persona que poco a poco se va a abrir a ti.

**E:**Que se empático, que te escuche

**e:**Que no haga tanto gesto, eso que he dicho, empatía, porque en general uno quiere que sean empáticos

**E:**¿Algún comentario adicional?

**e:**Espero que se integrado en el sistema de Essalud para personas policiales de las unidades, pero no es como debería ser. Porque en muchas ocasiones he sacado citas para nuestros pequeños para que puedan tener una mejor orientación, para que puedan pasar los problemas que hayan pasado en el colegio, por su creencia de la misma profesora. A modo personal, en mi opinión, que yo he ido, he encontrado a personas que todavía no han terminado la carrera que si bien es cierto tienen toda la disposición para poder atenderlo, pero tiene que ser visto por un profesional ¿no? Pero no los atienden ellos, sino que ponen al personal que está haciendo sus prácticas.

**E:**Le gustaría que sea un personal que sea profesional, con la formación del caso

**e:**Mas que todo por el tema de la experiencia, porque la experiencia te hace conocedor de lo que está pasando el paciente o de qué manera ayudarlos a resolver el problema

**E:**Gracias señor —----

**e:**Muy amable por haberme llamado y conversar este tema

**D18: Usuario_Policia**

**E:**Ok, entonces comenzamos. Buenos días, estoy aquí con la oficial —---, me gustaría que por favor se pueda presentar para poder iniciar la entrevista.

**e:**Mi nombre es —--------, tengo 34 años.

**E:**Su número de DNI por favor.

**e:**—--------

**E:**Ya, primero agradecerle por su tiempo, no le va a tomar mucho tiempo. Quería consultarle, entiendo que usted ha recibido una, ha tenido digamos una atención, ha usado un servicio digamos de atención remota. ¿Puede comentarme cómo ha sido esta experiencia que ha tenido?

**e:**Bueno, en la época del COVID no teníamos mucha, casi no había ninguna atención médica y lo que, lo más próximo fue una cita virtual, ¿no? Llamé por teléfono, me agendaron y me pusieron una cita y de ahí ya recibió todo el acceso para poder entrevistarme con un doctor.

**E:**¿Cuántas sesiones fueron de atención?

**e:**Bueno, habrán sido tres.

**E:**Me dice que usted llamó por teléfono. ¿Era fácil conseguir el número al que tenía que llamar?

**e:**No.

**E:**¿Fue complicado?

**e:**Fue complicado porque no tenía mucho, más que todo también tienes que tener un poco de conocimiento y que te recomienden un buen doctor, pues también, ¿no? para poder llegar.

**E:**¿Usted llamó a algún establecimiento o llamó a algún doctor particular, una clínica?

**e:**A un doctor particular porque me lo recomendaron.

**E:**Ah, ok. Y entonces entiendo que había allí digamos como un servicio de atención al cliente o usted directamente llamó con el médico para agendar una cita.

**e:**Bueno, en este caso llamé directamente a su secretaria y de ahí recién me pusieron un cronograma, ¿no?

**E:**Ok. Entonces entiendo que hubo dificultades para conseguir el número porque digamos no había, digamos usted esperaba cierta recomendación, ¿no? Entiendo que es algo que considera que es muy particular de usted o es algo digamos que ocurre en la mayoría del personal policial.

**e:**Sí, creo que en la mayoría porque a veces, por ejemplo, vamos a un lugar y no nos atienden bien o las medicinas que nos dan no nos cubre pues todo, ¿no? Y por eso es mejor de repente alguien que te recomiende para poder ir a ese doctor, ¿no?

**E:**Ok, entiendo. Podría decir que es algo más particular, digamos, de ustedes, ¿no?

**e:**Claro, exacto. Sí, que menos.

**E:**Ok, entiendo. Perfecto. En ese sentido, entonces usted me dice que le agendaron, inicialmente le agendaron tres sesiones o solamente le agendaron una.

**e:**No, al principio una y conforme voy evolucionando, ¿no? Cómo voy reaccionando a los medicamentos ya me citaron los demás. Ok.

**E:**¿Cuándo le agendaron? ¿Cómo le agendaron? ¿Le dijeron para tal fecha le vamos a llamar? ¿Iba a ser una reunión por videollamada? ¿Cómo iba a ser?

**e:**Bueno, me dieron unos siete días así y de ahí ya tenía cita. Ese mismo día me dijo, depende, vamos a recetar tanto y en siete días nos volvemos a conectar para ver cómo ha estado yendo.

**E:**¿Fue por Zoom entonces?

**e:**Exacto.

**E:**Ok. ¿Le dieron algún enlace o ese mismo día se lo enviaron?

**e:**Ese mismo día me enviaron a mi celular el enlace.

**E:**Ok. ¿Le pareció, digamos, la mejor forma, la forma más óptima que le envíen minutos antes el enlace o usted prefería que se le hubiesen enviado mucho antes como para programarse?

**e:**Creo que es mejor antes para que uno también se programe y vea pues un lugar, ¿no? Todo eso antes porque uno está preocupada, ¿me mandará o no me mandará? y eso, ¿no?

**E:**Ok. Mencionó del lugar. ¿La primera atención usted considera que fue en un lugar cómodo como le enviaron minutos antes, digamos, pudo encontrar un lugar como para que pueda tener la reunión de manera cómoda, privada, obviamente es una atención de salud.

**e:**Bueno, el primer día sí porque sí me dijeron tal día, tal, pero el siguiente me iban a confirmar la hora y todo eso y ahí como que no.

**E:**Hubo esa dificultad.

**e:**Exacto. Entiendo que porque es un tema de salud muchas veces también la conciencia, la privacidad es importante.

**E:**Ok, entiendo. Entiendo que para usted es importante, digamos, colocar una programación anticipada para que usted tenga el espacio de poder ver en dónde, en cuándo. Perfecto. Coménteme, ¿cómo fue la primera sesión con este médico?

**e:**Bueno, el doctor me mencionó cuáles son mis síntomas, él fue, este, supo llegar a mí porque me escuchó porque hay otros doctores que agarran y ya que tiene, ya, chao, algo así. El doctor me fue pacientoso, fue, este, didáctico también al decir las cosas y sí, sí me gustó su atención.

**E:**Ok, entonces la forma en la que le habló, la forma en que le preguntó información.

**e:**Claro, sí, supo llegar al paciente, pues, ¿no?

**E:**A usted en este caso, ¿no? ¿Cómo diría usted que fue, digamos, la atención? ¿Demostró interés en su?

**e:**Claro, claro.

**E:**¿Ok, demostró interés?

**e:**Demostró interés.

**E:**Ok, también le supo escuchar con las cosas que a usted le refería y para usted eso es muy importante.

**e:**Exacto.

**E:**Ok, en la segunda sesión entiendo de que ahí hubo problemas porque no le, estaba por confirmar la hora.

**e:**Claro, había, este, que el mismo día me mandaron a dos horas antes, creo, el enlace para poder, este, entrevistarme con el doctor.

**E:**¿El mismo doctor?

**e:**El mismo doctor, exacto.

**E:**¿Y cómo fue esta segunda sesión?

**e:**Mucho más rápida porque los medicamentos que me había recetado sí estaban haciendo efecto en mi cuerpo, ¿no?

**E:**Claro, era más para seguimiento, ¿entiendo?

**e:**Exacto, cómo seguía y todo eso y ya.

**E:**Ok. Ya no fue necesario entonces tanta, tanta información, tanta escucha, tanta didactica.

**e:**No, ya no tanto.

**E:**Era algo más breve y usted lo considera, digamos, también propicio.

**e:**Exacto.

**E:**Ok, la tercera, ¿cómo fue?

**e:**Ya la tercera ya, este, fue para darme algunas indicaciones para que no vuelva a recaer cuidados, ¿no? Y alimentación más que todo, ¿no? O medicinas que puedan subir mi, en ese tiempo, como vitaminas.

**E:**Ok, ok, perfecto. Entonces, de manera general, las tres sesiones, ¿usted cómo lo calificaría, digamos, del 0 al 10?

**e:**Bueno, en la atención muy bien como el doctor, ¿no? Pero la inquietud sería eso del, del Meet, ¿no? Pues, por ejemplo, este, que te mandan el enlace de repente ahí a las dos horas, tres horas. Ahí, este, uno está preocupado, pues, ¿no? ¿Me mandará? O todo eso. Pero en la atención, muy buena atención.

**E:**Y usted contaba con los dispositivos para poder tener la atención, o les fue difícil?

**e:**Bueno, yo, yo sí, sí contaba con los aplicativos.

**E:**¿Y usted está familiarizada con el uso, digamos, de este tipo de, de plataformas, de aplicaciones?

**e:**Sí.

**E:**Entonces, ¿no le fue difícil, digamos?

**e:**No me fue difícil porque yo también trabajo con este tipo de aplicaciones.

**E:**Ok, perfecto. Entonces, eso de alguna manera facilitó la atención.

**e:**Exacto, sí, porque hay gente que no conoce y, y es difícil, pues, ¿no? ¿Cómo abro esto, esto? Y tiene que estar al costado de una persona para que le pueda orientar.

**E:**Y respecto a las indicaciones médicas, ¿cómo se las envió? ¿Se las envió luego por WhatsApp?

**e:**Todo por WhatsApp. Me la envió de receta, todo, al toque. Termina y me manda el diagnóstico, me manda de repente qué análisis tengo que hacer.

**E:**Y esa información fue como por foto o en PDF?

**e:**En PDF.

**E:**¿Eso le fue más sencillo para usted? Sí, claro. Porque es más fácil entender la letra en PDF.

**e:**Exacto, que he escrito, pues, también.

**E:**Perfecto, son detalles. Entonces, ¿entiende usted que si volviera a tener algún problema de salud, podría, digamos, para ustedes, podrían volver a optar por la opción de atención remota o preferirían atención presencial?

**e:**Bueno, en ese tiempo yo hice así, ¿no? Pero ahora prefería, de repente, que me vea un doctor, pues, no ir. Porque una, ya, ahora que me vea, que me sienta, todo eso ya es diferente.

**E:**Entonces, usted dice que para ustedes es más importante, o digamos, no optaría tanto.

**e:**Depende la magnitud de cómo yo me sienta, pues, ¿no? De repente es algo leve, puedo agarrar y llamar, o de repente también la hora y todo eso. Porque muchas veces en el hospital de nosotros no nos quieren atender. O de repente por algo muy, para ellos muy, este, no es emergencia, ¿no? Por eso, no agarra. Vamos muchas veces a emergencia y no nos quieren atender. Entonces, ¿qué optamos? ¿En ir o de repente llamar y decir nuestro diagnóstico para que nos puedan atender?

**E:**Y respecto a, si por ejemplo, suponiendo que fuese un tema de salud mental, ¿usted optaría por tener una atención remota o preferiría que sea una atención presencial?

**e:**Si fuera algo, bueno, yo...

**E:**Por ejemplo, un tema de ansiedad, un tema de estrés...

**e:**Bueno, eso sería, creo que remota, pues, ¿no? No tendría un conveniente con que sea presencial. Exacto, podría ser en forma remota.

**E:**Ok, está bien. Bueno, no sé si tendrá de repente algún comentario adicional que le gustaría brindar.

**e:**Bueno... No, no, yo...

**E:**Ok, muchas gracias por su tiempo.

**e:**Sí, gracias.

**D19: Usuario_Profesional_Salud**

**E:**Buenos días, estimadas —----. Gracias por participar en el estudio llamado Diseño y Evaluación de Usabilidad, Aceptabilidad y Satisfacción de un Servicio de Telesalud, de Tamizaje, Manejo Inicial y Derivación Oportuna en Salud Mental para Trabajadores de Grupos Ocupacionales Vulnerables con Antecedentes de COVID-19 en Lima 2022. Te agradezco que hayas podido aceptar la entrevista y podernos haber brindado tu consentimiento de que informado a través del enlace que se te envió por Google Form. Y a continuación vamos a comenzar con la entrevista. Mi nombre es Jimmy Cainamarks. Te voy a realizar algunas preguntas relacionadas al proceso de investigación que llevamos a cabo. Y bueno, la idea es que nos cuentes con mucha sinceridad y nos cuentes de repente tu experiencia respecto a aquellas preguntas que te voy a realizar. En general, ¿cómo has sido, sientes que ha sido tu experiencia en todo el proceso de atención por teleconsulta que has tenido alguna vez? Por ejemplo, si es que has tenido que buscar atención, sacar citas, ser atendido. ¿Cuál ha sido de repente la valoración o la experiencia que has tenido?

**e:**Regular, diría, porque cuando yo me enfermé de COVID no me contactaron durante varios días a pesar de que me habían dicho que sí lo iban a hacer. Y yo solo estaba preguntándome si mi descanso médico se había hecho efectivo, si mis resultados habían llegado a donde correspondían, no sabía. Y bueno, eso, después de yo haber consultado con la jefa de la unidad de seguridad y servicio en el trabajo, recién recibí una llamada del médico y fue una única vez. Y me dijo el médico que se había equivocado registrando mi número de teléfono y que por eso no me había contactado en los días anteriores. Y bueno, fue la única vez que me llamó en los 14 días que estuve con descanso médico.

**E:**Entonces, en general, para que te hagan ese tipo de servicio, ¿te solicitaron algún requisito previo antes de brindarte la teleconsulta o atención online?

**e:**No, porque estábamos en pandemia y bueno, todas las pacientes que no tenían síntomas graves eran referidos a sus domicilios para guardar su cuarentena. Entonces, el monitor e iba a ser básicamente por videollamada. Y bueno, eso, me llamó, el que me llamó dos veces fue el psicólogo y él sí me atendió. Me gustó cómo me atendió porque me preguntó cómo me sentía, quiso saber más datos acerca de cómo yo vivía, si me estaba alimentando bien o si tenía algún síntoma. Pero de parte del médico no hubo una retroalimentación.

**E:**O sea, se entiende que entonces el único requisito como para que puedas haber recibido una atención solamente fue el diagnóstico, el diagnóstico de COVID-19.

**e:**Sí.

**E:**Y este dio dos tipos de consultas, ¿no? Una con un médico y una con un psicólogo. Y en el caso del psicólogo, pues su experiencia fue mucho mejor que la del médico. Sí. Y en todo ese tiempo, desde que tuviste tu primer diagnóstico de COVID, ¿cuántas teleconsultas has recibido?

**e:**Bueno, dos veces tuve COVID y en la segunda vez que me dio COVID, no recuerdo que no me llamó ningún médico. No, sí creo que sí me llamó. Me llamó una doctora y pues solo por una vez. No, no me volvió a llamar.

**E:**Y de las dos veces que te has atendido, entonces de las dos experiencias que has tenido, ¿Cuál crees que ha sido como que mejor tu experiencia?

**e:**Diría que la segunda vez.

**E:**Ajá. ¿Por cuáles razones de repente?

**e:**Primero porque me llamaron. El mismo día de mi diagnóstico me llamaron y me consultaron si tenía algún otro síntoma. Y me dieron las medidas que debía tomar para durante los días que iba a estar de descanso médico.

**E:**Y, o sea, en que te hayan llamado inmediatamente fue una de las cosas que más te gustó. ¿Podría decirse? ¿Hay alguna otra cosa que más te haya gustado de esa segunda atención respecto a la primera?

**e:**Que la doctora fue más amable, quizás. Me dio a entender que se preocupaba por que yo estuviera bien.

**E:**Ajá. Y haciendo un resumen, ¿Cuáles fueron las cosas que de repente no te gustaron en general de estas dos atenciones que has tenido?

**e:**Bueno, me habían comentado que el médico o doctora iba a llamarme para saber mi estado cada día. Me indicaron eso, ¿no? Pero en ambas oportunidades solo me llamaron una vez. Y pues no sentí un real monitoreo, digamos.

**E:**O sea, te prometieron algo que al final no cumplieron.

**e:**Ajá.

**E:**Y por el otro aspecto, lo que sí te gustó fue que inmediatamente te hayan abordado y tú hayas sentido una preocupación por tu estado de salud.

**e:**Sí.

**E:**Y cuéntame, ¿a través de qué medios recibiste esta teleconsulta, esta teleatención? Por ejemplo, teléfono, computadora, tablet, celular, una aplicación.

**e:**A través de teléfono en ambas oportunidades.

**E:**Ajá. ¿Alguna vez has recibido algún tipo de teleconsulta por Zoom, Mead o algún software especial?

**e:**No.

**E:**Ajá. Y ¿cómo consideras que ha sido tu experiencia utilizando estos medios, ¿no? Como, por ejemplo, me comentas que has recibido teleatención por teléfono. ¿Cómo ha sido tu experiencia por ese canal?

**e:**La verdad que parece poco personal. Hubiera preferido una atención más... una atención directa. O sea, que el médico hubiera ido a mi domicilio a constatar lo que yo le decía, los síntomas, signos que tenía. O a través de una videollamada, porque a través de una videollamada uno puede percibir ciertas cosas y uno quizás puede comunicarse mejor.

**E:**Ajá. Por ejemplo, en el contexto de pandemia no había como que presencialidad. Era algo bien difícil de pensar. Entonces, ponemos en la mesa, por ejemplo, dos medios. Uno que es teléfono y uno que es las videollamadas, como mencionas. ¿Cuáles crees que hubieran sido los beneficios y las ventajas de ambos canales? O las cuales te hubiera gustado recibir la teleatención como paciente.

**e:**Los beneficios de una videollamada, pues yo creo que es más... hay más cercanía, hay más... el médico puede observarte mejor. O tú puedes hablar más con el médico y consultarle ciertas cosas, dudas quizás. Y en la videollamada... perdón, en la llamada, pues hubiera querido que el médico me preguntara más. Y no sé, quizás su trato. Y pues, no sé, por la llamada me parece que es algo más... no sé, es más accesible porque quizás algunos otros pacientes no tienen internet y no podrían acceder a una videollamada.

**E:**Pero entre los dos, para ti, por ejemplo, ¿Cuál sería más accesible? Si estuviéramos que elegir algún medio virtual.

**e:**La videollamada.

**E:**Y cuéntame, ¿Cuáles sientes que han sido las barreras o los problemas que has tenido para recibir tu atención por teleconsulta? O sea, de repente me has comentado ahí algo respecto a que, por ejemplo, no cumplían con la agenda. Pero en el proceso, de repente, no sé, por ejemplo, se te fue la señal o de repente algún otro problema técnico. ¿Sientes que has tenido algún tipo de esas barreras? ¿U operativas?

**e:**Quizás porque hubo un momento en el que el psicólogo quiso contactarse conmigo, pero a mí me asustan los ruidos fuertes, las vibraciones, entonces si no voy a recibir una llamada, mi celular está en silencio. Y entonces perdí su comunicación, pero él me contactó a través de WhatsApp. Y entonces cuando le dije que estaba disponible, el psicólogo me llamó.

**E:**Correcto. ¿En ese momento ustedes habían pactado un horario específico para atenderse?

**e:**No, esa es otra cosa que también no me terminó de gustar, porque entiendo que es por la pandemia, pero no había un horario en el que te contactaran. Te podían contactar en la mañana, en la tarde, no...pienso que eso no estaba como que, arreglado, o sea, a mí no me habían comunicado cuándo me iban a llamar. Entonces tenemos algunos temas que a veces no se cumplía el agendamiento tal cual, o a veces no te podían llamar en cualquier momento, y por otro lado que a veces por tu tema de trabajo tenías el teléfono en silencio y por lo cual no estabas atenta a poder recibir, o estar atenta a recibir la llamada.

**E:**¿Consideras que hay alguna otra barrera adicional a estas?

**e:**No.

**E:**Ok. ¿Y qué beneficios o facilidades crees que se deben tener en cuenta durante una teleconsulta que puedan ser mucho mejor en comparación a las atenciones presenciales?

**e:**Pues que, si es concertado, tú puedes evitarte el estar trasladándote a un centro de atención, ahorrando tiempo en el transporte y poder manejar tus tiempos al tener una tele. No invertir este tiempo en poder trasladarte y poder desde tu casa o desde tu trabajo quizás acudir a una consulta y en caso se necesite alguna prueba complementaria que el médico ya te facilité la orden a través de un correo, o no sé, o que estabas establecido. Tal vez un sistema en el cual te llega una orden al celular y con la cual tú puedas acercarte a un centro de atención y puedas hacerte las pruebas que te recomienda el médico.

**E:**¿Qué canal crees tú que sería el más adecuado para justamente recibir ese tipo de información?

**e:**El correo.

**E:**¿Qué otras cosas de repente sentirías que haga falta en temas operativos y de la atención que crees tú que haría que te sientas más cómoda durante la teleconsulta o de repente durante el agendamiento de la teleconsulta?

**e:**Principalmente que se tenga una historia clínica electrónica en la cual estén todos mis datos y que cuando otro médico me atienda pueda tener acceso a esa información en línea y que la información no se disperse en hojas, en computadoras, no lo sé. Me gustaría que haya una historia clínica electrónica y si fuera nacional mejor.

**E:**En comparación, entiendo que me comentaste al inicio de que en términos ideales te gustaría que en un tipo de atención de teleconsulta sea presencial, pero luego también me comentaste sobre algunas ventajas respecto a tener una teleconsulta porque lo puedes realizar en la medida de tu tiempo. Entre las dos, específicamente para teleatención, salud mental, de repente con psicólogo profesional, ¿Cuál de las dos crees tú que sería un buen medio para que puedas recibir ese tipo de atención?

**e:**Bueno, nunca he acudido a una atención psicológica, aparte de la que me brindaron. Yo consideraría que un buen medio podría ser la teleconsulta, pero es porque no he experimentado una consulta presencial. Yo considero eso.

**E:**Bueno, muchísimas gracias —------ por haber participado en la entrevista y gracias por la información que nos ha dado.

**e:**Ok, gracias.

**D20: Usuario_Profesional_Salud**

**E:**Ok, buenos días licenciada, entonces vamos a iniciar la entrevista, para lo cual nos gustaría que por favor pueda mencionar su nombre completo y su profesión.

**e:**Yo soy —--------- y soy tecnólogo médico en terapia de lenguaje.

**E:**Ok, está bien licenciada, bueno, como ya sabe, nuestra intención es poder conocer un poco de su experiencia recibiendo este servicio de teleconsulta. Entonces quisiera iniciar para saber si... ¿Cuándo recibió usted este servicio?

**e:**Yo recibí el servicio de teleconsulta en julio del año 2020.

**E:**Ok, julio del año 2020. ¿Cómo fue su experiencia?

**e:**Bueno, todo esto viene porque mi familia en el mes de junio cae infectada por COVID, tanto lo que es mi hermano, mi papá y mi mamá. Entonces, mi hermano y mi papá y mi mamá estuvieron hospitalizados, mi mamá y mi papá en el hospital de la Fuerza Aérea y mi hermano en el hospital... ¿Ese es el seguro? ¿Cómo se llama? Se me olvidó el nombre. Ah, en un hospital del seguro. Ok. Y la cosa es que yo también estuve enferma, pero no sé por qué motivo no adquirí los mismos síntomas que ellos. Ellos llevaron a neumonías en porcentajes altos. Mi hermano llegó en 50%, mi papá en 20% y mi mamá en 70%. Entonces, todo esto pasó, se complicó mi mamá de manera bastante grave, estuvo hospitalizada en UCI en la FAP por 8 días y mi hermano estuvo hospitalizado 5 días y mi papá también estuvo hospitalizado 12 días, no recuerdo mucho. Entonces, llega a morir mi madre y yo me encontraba básicamente sola haciendo todas las actividades, ¿no? ¿A qué me refiero con actividades? Pero bueno, a hacer el entierro sola, a ir a ver a mi papá sola, al retiro del cuerpo sola, a mi hermano también, atenderlo solo porque cuando le dieron la etapa yo tuve que atenderlo en casa.

**E:**Y en base a eso, usted con esa experiencia requería atención.

**e:**Sí, en realidad me sentí bastante... justo lo mencionaba yo a mi hermano de lo que iba a pasar y me sentía desesperada, sola, pero sentía que necesitaba hablar con una persona profesional. Tuve soporte desde antes de que pasara todo esto por un especialista en salud mental del MINSA que me empezó a decir que si yo no sintiera culpa por estar bien en comparación con mi familia y me estuvo llamando regularmente.

**E:**La atención fue, entiendo, por teléfono con ese profesional.

**e:**Sí, todo fue por teléfono. Llamé a la línea 113 y ahí me atendió.

**E:** Ok, ok, está bien. Entonces su primera experiencia con esa teleatención, teleconsulta fue usted en ese contexto necesitando apoyo llamando a la línea 113. Luego que se comunicó con la línea 113, asumo que le derivaron a este profesional de salud, el cual ya tenía sus datos y él se estuvo comunicando con usted, usted se comunicó con él. ¿Cómo es que fue eso?

**e:**Él me llamó una vez más y después ya no me llamaron. Me llamaron del seguro y también me llamaron del DIRIS, de la DIRIS Lima Norte. Entonces, no sé, supongo que había mucha demanda, qué sé yo. Me llamaron del seguro, del seguro sí me estuvieron llamando continuamente y de la DIRIS Lima Norte también, lo que es la PAR, el área de salud ocupacional. Y también me hacían, no sé si era su trabajo, pero la verdad es que me acompañaron mucho.

**E:**Ok, entonces fueron digamos tres experiencias que usted ha tenido. Uno con la línea 113, otra fue con la de su seguro y la última fue con la DIRIS, la unidad de salud ocupacional de la DIRIS. Ok, hablemos un poco sobre la primera experiencia con la línea 113. Entiendo que ahí usted se comunicó, entiendo que estaba en esa situación y bueno, ¿Qué necesitó para ser atendida? ¿Fue suficiente llamar? ¿Le pidieron algún dato más?

**e:**No, no, bueno, me pidieron mi DNI, mi nombre y al toque me derivaron. No fue nada complicado. Y si mal no recuerdo, quien me atendió fue un psiquiatra que se presentó como médico. Entonces, le conté mi experiencia, no me sentí apurada, no me sentí que necesitaba hacer otra cosa. O sea, se siente cuando te atienden con tiempo. Se dio todo el tiempo de escucharme, se dio todo el tiempo de hablar conmigo. Fue una conversación de 40 minutos, fue algo largo. Y me explicó, ¿no? Porque en ese momento cuando mi mamá estaba enferma era un poco agresiva conmigo. Entonces yo me sentía muy frustrada por eso, por su agresividad. Entonces él me explicó, me dio ciertos nombres, ¿no? De que en realidad lo que se siente es mal, está frustrada, que tú tienes que entenderlo. O sea, me hizo entender, ¿no?

**E:**Claro. Entonces entiendo de que, a raíz de esta situación, ¿no? Usted en esta primera experiencia hizo como, pudo generar este vínculo con el profesional. No le fue difícil, digamos, ¿no? Sino que pudo generar este vínculo, no hubo problema. En la siguiente comunicación que él le llamó a usted, ¿Cómo fue?

**e:**Eso sí fue un poco más rápido. Eso ya fue cuando mi mamá estaba internada. Le comenté, me volvió a repetir que no tenga culpa por sentirme, por estar sana. Sino que, al contrario, me identifique como la persona fuerte dentro de mi hogar. Que tenga que cumplir con mis actividades y que yo voy a poder porque en realidad las coincidencias para él no existían. O sea, no me lo jaló tanto por lo místico, pero como que me dio una idea, ¿no? Entonces me sentí acompañada, a pesar de que fue un poco más rápido, como le digo.

**E:**¿Cuánto más o menos duró?

**e:**10 minutos. A pesar de que fue un poco rápido, me ayudó.

**E:**¿Sí? Sintió ahí, como ya había tenido una previa experiencia con él, de repente ya había esa confianza, ¿no? No había, digamos, tanto...  ¿Qué considera que de repente faltó en esa segunda sesión?

**e:**En realidad, no siento que haya faltado mucho. Porque como dijo usted, ya sentí un vínculo posterior. Me agradó haberme sentido acompañada. O sea, siento como que a pesar de que sé que hubo muchas llamadas, al menos se acordó de mí. Claro. Y eso me agradó.

**E:**¿En qué momentos fue dado, digamos, la... ¿Usted qué estaba haciendo mientras recibió estas atenciones?

**e:**Cuando fue la primera atención, yo estaba en casa. Estaba haciendo teletrabajo. Ok. Entonces, atendía... Hacía terapia de lenguaje a nivel virtual. En la segunda, sinceramente, con todos los movimientos y cuando mi mamá ya estaba enferma, todos, yo ya dejé de hacer eso. Suspendí, así que me dedicaba solamente a atenderlos a ellos. A cocinar, a bajar a mi perrita, a encargarme de todo lo del hogar.

**E:**Ok, ok. Está bien. En el segundo caso, cuando le llamaron... Le entiendo que le llamaron de su seguro, ¿verdad?

**e:**Sí, me llamaron del seguro.

**E:**Ok. ¿Qué pasó allí? ¿Se comunicaron con usted por teléfono? ¿Le enviaron un WhatsApp? ¿Cómo fue?

**e:**Se comunicaron conmigo por teléfono. La verdad es que no sé por qué. Supongo que habría... Como yo fui a verme, a quererme atender, pero al final resultó que estaba saturando 100. O sea, todo estaba en mi cabeza, supongo yo, por algo ansioso. Ahí no sé cómo se generó el vínculo y la verdad es que hasta ahora no entiendo cómo me llegaron a llamar.

**E:**Pero usted directamente no fue que... ¿Cuál se enteró?

**e:**No, yo no lo he buscado. No, no, no. Nada. Ah, ya sé por qué. Lo que pasa es que yo llamé para que hicieran pruebas a mi hermano, a mí, porque los dos somos asegurados. Entonces, imagino que por eso generaron un contacto y me estuvieron llamando. Ellos ya me llamaron posterior a lo del doctor, pero si le soy sincera, esas personas se notaban que no eran psiquiatras o algo especialista, pero sentí mucha más empatía con ellos.

**E:**¿Por qué?

**e:**Porque ellos me contaban... justo le comentaba al médico jefe que, con ellos, por ejemplo, yo le contaba que había muerto mi mamá, que me sentía muy mal, y el señor me decía que él también se sentía mal, que había fallecido mucha gente en su trabajo, que por todos lados pasaba eso y que era inexplicable. O sea, me sentí identificada con lo que él me comentaba. Entonces, por eso sentí mucha más empatía con esa persona, porque en el seguro te llaman diferentes personas, no te llaman la misma. Ya. Entonces, pero, o sea, si usted me pregunta de las otras comunicaciones, no me acuerdo tan bien como el discurso de esa primera persona a la cual yo me sentía identificada y me comentaba que se sentía dolida porque él también había perdido muchos compañeros de trabajo, que también trabajan en el área del trabajo y ya no estaban con él presente. Entonces, me identifiqué.

**E:**¿Cuántas consultas tuvo con el seguro?

**e:**En el seguro habré tenido fácilmente 6 o 7 consultas.

**E:**¿6 o 7? Pero eran diferentes personas.

**e:**Sí, eran diferentes personas.

**E:**Usted, por lo que entiendo, para usted fue más significativa esta primera llamada. Sí. ¿Fue mucho más empático? ¿Cuánto más o menos duró?

**e:**Habrá durado 10 minutos. 10 minutos.

**E:**¿Fue por teléfono? ¿Fue, digamos, muy directa y generó bastante empatía con usted? Con lo que conversó.

**e:**Sí.

**E:**Ok. ¿Con las demás llamadas cómo fue?

**e:**Fue algo más corto, 2 o 3 minutos, en los que me preguntaban cómo me sentía yo a nivel físico, cómo se encontraba mi familia a nivel físico. Y fue algo más de seguimiento, de observación, no tanto de conexión para ver cómo yo me sentía moralmente. Ok, entiendo.

**E:**Entonces, ¿estos profesionales que le llamaron se identificaron con algún cargo o algo?

**e:**Me decían que somos responsables del área de teleconsulta del seguro de salud social.

**E:**Sí, ya muy general. En la tercera experiencia, que ya le llamaron de acá a la oficina de seguridad del trabajo, de la DIRIS.

**e:**De la DIRIS yo sentía bastante empatía, bueno, también por lo que me pasó. Me llamaron varias veces. La primera fue muy genérica, tipo para decirme, ¿sabes qué? ¿Vas a tener descanso? ¿Por qué estás enferma? ¿Por qué haces algo positivo? Blah, blah, blah. Para coordinar cómo tomarme el examen, qué sé yo. La segunda fue para preguntarme cómo estaba y justo mi mamá había fallecido y yo estaba yéndome al cementerio. Y yo le digo al doctor, mi mamá ha fallecido. No, perdón, recién había fallecido y yo me estaba movilizando para sacar el cuerpo. Me decía, mi mamá ha fallecido doctor. Me dijo, olvídate que te he llamado. Ay, olvídate que te he llamado. No te preocupes, te llamo en dos semanas. O sea, como que, porque supuestamente por la ley yo tenía ya que volver otra vez a trabajar. Entonces él me dijo, descuídame. Yo no te he llamado, olvídate, concéntrate en tus cosas, en tu familia y ya hablamos en dos semanas. Y en dos semanas me volví a llamar, el mismo doctor. Para preguntarme cómo estaba, cómo estaba físicamente. Y de ahí, la tercera me llamó la señorita Marilyn, que es una señorita de, hasta ahora tengo su nombre. Y a veces hablo con ella. La señorita Marilyn fue recontra empática conmigo, me ayudó mucho. Ella fue la que me dio el alta en el escenario ocupacional. Me dio el alta y fue muy buena conmigo. Hasta me acuerdo su nombre, te imaginas. Sentí mucho, mucho, mucho, bueno, como ese cuidado de trabajador que uno necesita. No que ya sabes que por ley cinco días falleció tu familia, ya te tienes que reintegrar al trabajo. No, al contrario, me comprendieron mucho. Que me entienden.

**E:**¿Cuántas sesiones más o menos tuvo?

**e:**Me habrán llamado seis, siete veces también.

**E:**Allí usted fue también, no tuvo problemas para hacer, fue el mismo...

**e:**Sí, fue la de DIRIS la que me llamó. Igual ellos tenían un correo o un número de teléfono por si te querías comunicar con el área ocupacional.

**E:**Claro. ¿Cuánto más o menos duraban las llamadas con la Iris?

**e:**Eso sí, era muy corto, cinco, seis minutos.

**E:**¿Ha sido breve entonces?

**e:**Sí, era breve.

**E:**Entonces, por lo que usted menciona, el tema del canal del teléfono no fue, digamos, una dificultad.

**e:**No, para mí no.

**E:**Para usted más bien fue, digamos, algo que le permitió tener soporte en esos momentos donde no tenía soporte.

**e:**Exacto. Lo que pasa es que como le comenté, el día de ayer estaba comentando que vamos a hacer esto, yo a una persona, mi pareja, que era mi pareja en esa época, mis primas, mis tías, yo podía contarle, pero a mí no me gusta generar lástima, entonces no me sentía cómoda comentándolo con otras personas. Al contrario, cuando hablé con otras personas que no tenían que ver con mi entorno, me sentí escuchada. Igual, bueno, no sé si sirva mucho para la investigación, cuando, por ejemplo, yo estaba esperando a mi hermano fuera del hospital Rebagliati, hicimos como una especie de círculo, cinco o seis mujeres que no nos conocíamos, que nuestra familia estaba internada, y la habíamos pasado tan mal que de broma nos empezamos a medir con el oxímetro, y todas teníamos COVID, pero todas estábamos allí, y todas empezamos a contar nuestra historia. Fue tan, tan liberador y enriquecedor el que cada una de nosotras escuche y hable de eso, que eso también me hizo sentir bien.

**E:**Ok, entiendo. Entiendo que usted, entonces, volvería a pasar por teleconsulta.

**e:**Sí, sí volvería a pasar por teleconsulta. Yo confío mucho en ese tipo de atenciones, en esa área, en el área de escucha psicológica, porque posterior a ello yo también he recibido, pero de manera particular, terapia psicológica por vía virtual. Entonces, yo sí siento que conecté con mi psicólogo.

**E:**Ok, ok, está bien. ¿Tendría algún motivo por el que quizá no preferiría llevarlo de manera presencial antes que virtual o por teléfono?

**e:**En realidad no, porque mi ritmo de vida es muy agotador.

**E:**Ok, entonces, para usted, por el tema de acceso, por el tema de la facilidad, ¿preferiría, entonces, en su caso, pasar por el tema de atención virtual?

**e:**Sí, lo preferiría por una cuestión de accesibilidad, de rapidez y porque me parece más práctico.

**E:**¿Preferiría que sea por teléfono, por video?

**e:**Videollamada me gusta más.

**E:**¿Por qué?

**e:**Porque puedo mirar los gestos de la persona, y siento que me está escuchando más directamente. Yo soy muy terapeuta de lenguaje y me gusta mucho mirar el rostro de las personas cuando estoy hablando. Entonces, para mí, eso me da una información de que sí me está entendiendo, me está escuchando.

**E:**Claro, el lenguaje no verbal.

**e:**Exacto.

**E:**¿Qué podría usted mencionar que le gustaría, aparte de visualizar el lenguaje? Asumo que entonces el medio tiene que ser un medio virtual, le sirve, o sea, remoto le sirve porque tiene muchas cosas que hacer y en cualquier momento es fácil para hacer conectarse. Tiene que tener, aparte, una especie de contacto visual, un feedback visual tiene que tener, o sea, tiene que haber una cámara. ¿Qué otra cosa más debería de tener? Para usted.

**e:**Un buen audio. Ok. Un buen audio porque a veces cuando se pierde la comunicación.

**E:**¿Le ha pasado eso? Sí, sí me ha pasado. ¿Y cómo ha sido? ¿Ha interrumpido ese rapport que estaba generando?

**e:**Sí, exacto, pero a mí corta el rapport totalmente que no le puede entender lo que me dice. O se va una buena conexión también, también me ha pasado que a veces el profesional no tenía una buena conexión de internet y decía, te está viendo, te estoy viendo. Y para mí esos son minutos perdidos, entonces me parecía incómodo. ¿Y qué más? Nada más, básicamente eso. Ah, y que el ambiente donde se encuentre el profesional, o sea, el fondo sea cálido. Por ejemplo, yo recuerdo mucho que la psicóloga particular que veía tenía un fondo muy bonito, de colores pasteles, que a mí me parecía más agradable esa vista y me daba más confianza que ella.

**E:**Ok, ok, está bien. Entonces, la conectividad, el audio, un feedback visual, que implica también obviamente en dónde se ve el fondo, le influye bastante para poder usted tener esta, digamos, facilidad en la atención. Ok, por último, quisiera, digamos, consultarle de si acaso, por ejemplo, hay algo más que usted podría decir que de repente como que no le ha gustado tanto en esas atenciones. ¿Qué podría, digamos, quitar de lo que en su experiencia ha tenido?

**e:**Eh, había algunas llamadas que son las que no recuerdo tanto, en las que solamente es la pregunta, ¿cómo te sientes? Bien, o sea, tipo, ¿cómo te sientes? Recolectar si estás bien, mal o peor, pero no decirte, y ¿cómo te sientes al respecto? O sea, las preguntas muy directas me parece que no generan en una persona a que se abra más. En cambio, puedes preguntar un simple ¿cómo te sientes bien? y luego ¿cómo te hace sentir ello? y ¿qué piensas al respecto? Son preguntas que están más dirigidas a que te apertures, ¿no? ¿Cómo te sientes el día de hoy?

**E:**Claro, ¿cuáles fueron las preguntas que le ayudaron a usted a aperturarse más o sentir que le interesaba?

**e:**Y ¿qué piensas de ello?

**E:**O sea, por ejemplo, imagínense, yo le estoy llamando, o usted me llama a mí, por ejemplo, ¿qué me diría, digamos, para iniciar la conversación?

**e:**Hola, y también en todo de voz, o sea, hay cosas que yo hago mucho.

**E:**Ajá, por ejemplo, y es perfecto, a mí me gusta esa información. Imagínese, usted me llama, yo le voy a contestar, hagamos la simulación, llámame.

**e:**Hola, ¿cómo está?

**E:**Aló, sí, ¿qué tal?

**e:**¿Qué tal? Le hablamos del centro de salud, queremos hablar el día de usted, ¿cómo no puede hablar conmigo?

**E:**Sí, ahorita estoy saliendo de mi trabajo, pero sí, le puede escuchar.

**e:**Muy bien, le estoy llamando porque me encantaría saber cómo ha ido usted en su tratamiento, qué ha sentido, cómo se sienten estos días.

**E:**Bueno, no me ha ido de cierta forma porque he estado trabajando. O sea, ¿a usted le interesaría, digamos, ese tono de voz?

**e:**Claro, un tono de voz que vaya. O sea, por ejemplo, le digo que es mi carrera, pero yo me siento más escuchada por un tono de voz más prosódico. Como yo recuerdo, la señorita, hola, ¿cómo te sientes el día de hoy? O sea, es muy frío.

**E:**Ayuda mucho esa calidez de hablar.

**e:**La prosodia siempre va a ser que la persona este más dispuesta a aperturarse. Si a nivel presencial es muy importante a nivel esto, es muy... Por eso a la señorita Marilyn la recuerdo por su tono de voz muy dulce. Ese tono de voz "ya, no te preocupes... tú tranquila... nosotros te vamos a apoyar". Esa forma de hablar tan cariñosa, tan bonita. Quizás por mis circunstancias, lo valoraba. Y mira ahora recuerdo hasta su nombre. 3 años después

**E:**Por mi parte, no tengo ninguna duda

**e:**Gracias

**E:**¿Quisiera mencionar algo más?

**e:**Que en realidad en ese momento todos pasamos por algo difícil, pero me di cuenta que soy muy querida por mis amigos, mis compañeros de trabajo, me hicieron un club de fans que tengo. El sentirte querido te hace ser fuerte en los momentos difíciles. Porque para mí de verdad, sino hubiera sentido el apoyo de gente que yo en algún momento ayude sin pensarlo, no habría salido adelante como lo hice.

**E:**Gracias

**D21: Usuario_Profesional_Salud**

**E:**Muy buenas tardes, —-. Mi nombre es —-----. Gracias por participar en el estudio que lleva por título Diseño y Evaluación de Usabilidad, Aceptabilidad y Satisfacción de un Servicio de Telesalud de Tamizaje, Manejo Inicial y Derivación Oportuna en Salud Mental para Trabajadores de Grupos Ocupacionales Vulnerables o Antecedentes de COVID-19 en Lima de este año. Bueno, habiendo hecho lectura ya del consentimiento informado y habiéndonos confirmado tu participación, entonces vamos a comenzar con entrevista que va a tener una duración de 15 a 20 minutos. Entonces te voy a realizar una serie de preguntas y nos puedes comentar con toda amplitud sobre acerca de las opiniones que de repente te van a surgir a través de estas preguntas.

**e:**Ok

**E:**¿Cómo ha sido tu experiencia en todo el proceso de atención por teleconsulta que has tenido? O sea, por ejemplo, si es que has tenido que buscar la atención o sacar cita para ser atendido, por ejemplo, a través de teleatención.

**e:**En este caso, por teleconsulta en el contexto de COVID, me llamaron prácticamente. Una vez que recolectaron mi muestra, me hicieron la prueba, resulté positivo. Después de ello, me contactaron vía telefónica para saber cómo iba mi procedimiento, cómo iba mi enfermedad, mis síntomas, cómo me sentía y a veces también envían un recordatorio sobre cada cuántas horas debía de tomar los medicamentos que me habían presentado.

**E:**Correcto, ¿y cómo consideras tú que ha sido en forma general tu experiencia? ¿Cómo te has sentido? ¿Te has sentido cómodo o no tanto? ¿Ha sido positiva, negativa? ¿Cómo lo ves?

**e:**Voy a decir intermedia. En lo que yo esperaría, esperaría que estén constantemente conmigo todos los días. En cada cierta hora que ellos me decían, vamos a llamar a las 2 de la tarde, de repente que sea constante esa hora o incluso que sea de manera diaria, lo cual no se daba muchas veces. Venía una atención un día a la una de la tarde y luego pasaban tres días, cuatro días, me volvían a llamar y de repente muy muy tarde, ya 6 de la tarde, 8 de la noche. Entonces pasaba eso, que no se cumplía digamos que estrictamente los horarios para poder llamar y comunicarse conmigo.

**E:**Ok, entonces de manera general consideras que habría cosas que no llegaron a completar tus expectativas iniciales. Ajá, sí. Y para ello, de repente te solicitaron algún requisito previo antes de realizarte las teleconsultas o las atenciones.

**e:**En cuanto a requisitos, a mí no me pidieron nada. Lo que tengo entendido es que ellos ya tenían mi resultado de la prueba de COVID y en base a eso, sus requisitos para ellos deben ser eso, que el resultado sea positivo y con ello ya contacto conmigo, ya que ellos también tienen mis datos de número de teléfono, dirección, correo.

**E:**Claro, o sea el proceso administrativo fue la confirmación de tu diagnóstico para poder iniciar el proceso. Y recordarás en total cuántas de las consultas has recibido respecto a ese tipo de servicio.

**e:**Exactamente habrán sido unas 3 o 4, te refieres a las oportunidades de teleconsulta, ¿no? Claro, habrán sido entre 3 y 4, de las 3 a 4, 2 por médicos y 2 por psicólogos.

**E:**Ok, y cuéntame un poco sobre esa experiencia que has tenido con estos distintos tipos de profesionales. ¿Qué es lo que más te gustó, por ejemplo, en general por el servicio de teleconsulta y en particular sobre hacerlo con cada uno de estos perfiles que te ha tocado recibir la atención?

**e:**En el caso de los médicos que me atendieron, fue más puntual realmente, preguntaban por síntomas, si estaba tomando o no estaba tomando y si me sentía, digamos que un poco mal, un dolor de cabeza, que siga tomando, que siga tomando. Por la parte del otro profesional de psicología, sí era un poco más amplia el tiempo de la consulta, porque me hacían más preguntas de cómo me sentía, cómo me iba, cómo iba mi familia, cómo estaba también. Entonces sentía un poco más de preocupación por parte del profesional psicólogo que digamos que por el profesional médico, pero bueno, quizás son sus funciones, esa manera de cada uno.

**E:**¿Y crees que hay algo más de repente que te gustó por uno o por otro lado? O en general es lo que me acabas de comentar. En general, sí. Y ahora vamos por lo otro contrario, ¿qué es lo que no te gustó de ambos perfiles o de manera general?

**e:**De manera general lo que te comentaba al inicio, básicamente por la atención médica, que eran muy puntuales y no se daban de manera constante.

**E:**Y en particular de repente por cada tipo de profesional.

**e:**En manera particular, pues la fase del psicólogo me parece una buena atención, no tengo nada que agregar, de verdad. Solamente por la parte de la atención médica.

**E:**Ah, ok, ok. Y cuéntame, esta teleconsulta, ¿por qué medio lo pudiste recibir? ¿De repente a través de un teléfono, a través de tu computadora, de tu celular?

**e:**Sí, fue a través de celular, me llamaron por celular.

**E:**Ok, fue por llamada telefónica.

**e:**Llamada, así es.

**E:**¿Alguna vez has tenido una experiencia dentro de esas veces que cuentas que has tenido teleconsultas por otro medio que no sea llamada telefónica? Videoconferencias, Zoom, ¿no?

**e:**No, en teleconsultas, no, ninguna. Todas han sido por llamadas.

**E:**Y en general, ¿qué te pareció el medio con el cual recibiste la teleconsulta? En ese caso lo que hiciste fue por teléfono, ¿no? Y en general, ¿cuál ha sido tu perspectiva? ¿Qué te pareció hacerlo por teléfono?

**e:**Me pareció buena, sin embargo, creo que considerar solamente la vía telefónica como llamada normal podría tener ciertas limitaciones. Porque podría yo quizás ubicarme en una zona donde no haya buena señal de teléfono, pero sí buena señal de internet, ya que como si voy a estar en aislamiento por COVID, generalmente estoy en mi casa, no es normal que estaría en mi casa, y ahí sí cuento yo, particularmente con Wi-Fi. Entonces una llamada por WhatsApp sería, digamos que mucho más estable la señal que una llamada por llamada normal. Entonces, en ese aspecto quizás podría mejorarse, ¿no? Si es que podrían hacer la llamada quizás por WhatsApp, o intentar quizás las dos llamadas, ya WhatsApp y llamada normal.

**E:**Ok, ¿y cuáles crees que han sido de repente las ventajas y desventajas, como conclusión, ¿no? De, por ejemplo, haber recibido la atención a través de teléfono, ¿no? O con esos perfiles profesionales de salud que te ha tocado.

**e:**Mmm... Más que ventaja, pero un poquito de desventaja. Sobre todo, para la atención médica, porque digamos que el médico quisiera preguntarte un poco más sobre tus síntomas, pero también quisiera ver cómo tú te ves, cómo te encuentras, y esa evaluación no te la puede hacer por una llamada de teléfono, a menos que sea una videollamada. Posiblemente ahí te podría ver, ¿no? Pero eso creo que podría haber faltado, ¿no? Quizás sí hubiese sido bueno contar con videollamadas en las teleconsultas.

**E:**Mmm... Ok. Y cuéntame un poco sobre cuáles han sido de repente las barreras o los problemas que has tenido para recibir tu atención por teleconsulta, ¿no? Por ejemplo, comentaste un poco del teléfono, a veces el tema de señal, ¿no? Que, por ejemplo, en tu caso en particular, no hay muy buena señal de teléfono cerca de tu casa, te convendría el tema de Wi-Fi, ¿no?

**e:**Sí.

**E:**¿Qué otros problemas operativos o técnicos tú has encontrado, por ejemplo, al momento de poder recibir esta teleatención?

**e:**Mmm... Otro problema, más allá que el tema de la señal, no he podido encontrar otro realmente. Eso es lo que he podido identificar.

**E:**Ok, ¿o de repente para poder agentar la atención? O, por ejemplo, si es que has tenido que citarte en tal horario, o de repente...

**e:**Claro, eso más o menos es lo que te comentaba casi al inicio, ¿no? De que el tema que el médico te dice que te voy a llamar tal fecha a esta hora, pero no pasa a esa hora. Ah, ok. Incluso quizás tampoco pasa en esa fecha. Entonces eso es como que tú pensabas que te iba a llamar, pero luego no te llaman. Es una limitación que ha tenido, un problema que he podido identificar.

**E:**El incumplimiento de la agenda que se había acordado.

**e:**Sí.

**E:**Ok, ¿y tú crees que existen beneficios o facilidades para recibir las atenciones por teleconsulta en comparación a las atenciones presenciales?

**e:**Beneficios es que no necesitas moverte en caso tuvieras algún problema de moverte en caso de inmovilización. Y en este caso sí fue necesario. Eso sería una de las ventajas. Otra ventaja, si fuese una teleconsulta por videollamada, digamos que podría visualizar un poco mejor al paciente. Pero, por otro lado, una consulta presencial, digamos que también transmite una mejor comunicación con el médico o con el profesional de salud. Si es que el profesional tiene que hacer algunos tipos de auscultación, exámenes médicos, clínicos, entonces ahí en ese caso solo se pueden dar de manera presencial. Muchos no se pueden dar de manera virtual. Eso es una limitación.

**E:**Y en particular en la atención, en la teleatención por ejemplo psicológica que has tenido, ¿has sentido de repente alguna facilidad o beneficio en comparación con la presencial?

**e:**Sí. La verdad es que compararlo con una atención psicológica presencial no sabría cómo hacerlo porque no he recibido anteriormente consultas presenciales por psicología. Pero esta teleatención por psicología me pareció buena. Me pareció así interesante como se desenvolvió.

**E:**Ok. Pero, o sea, ¿no habría como que un punto algo para decir que esto es mejor que otro?

**e:**En mi caso no podría porque no he tenido una oportunidad de una atención psicológica presencial.

**E:**Ok. Y como recomendación de repente que podrías dar a un programa de teleconsulta.

¿Qué crees tú que haría que te sientas más cómodo durante la teleconsulta? ¿O qué debería haber o pasar en un programa así de ese tipo?

**e:**El cumplimiento de horarios, el seguimiento al paciente y si es que existe la posibilidad de que haya una teleconsulta por videollamada en la cual se pueda ver al paciente, sería ideal. Sí, eso básicamente.

**E:**Y cuéntame un poquito sobre el seguimiento, por ejemplo. ¿Qué te gustaría que hay en ese seguimiento?

**e:**Bueno, si en este caso hablar de manera general o hablando por COVID nada más.

**E:**Empecemos por lo general y luego vamos por COVID.

**e:**De manera general, que bueno, si es que es una teleconsulta por algún problema de daño de la salud que tenga, una enfermedad crónica, por así decirlo, sí me gustaría que estén programadas las atenciones diarias o interdiarias o una fecha determinada y que estén preguntando constantemente cómo están tus síntomas, cómo te está yendo. Y el otro tema es que uno particularmente si se siente un mal de salud en ese momento, digamos que no existe la posibilidad de comunicarte con el médico tratante, sino que tienes que esperar a que el médico te llame. Digamos que eso, no sé, te limita un poco en que tu atención sea buena. Porque puedes tú tener un síntoma un día que no te va a llamar el médico y no hay manera de cómo reportarle a ese médico. Porque tú tienes que esperar a que el médico te llame según la programación.

**E:**Justo mencionas dos partes importantes del proceso de seguimiento. Uno es que por ejemplo cuando se apertura la cita, que es como maneja la logística de que tal fecha te vas a atender y tal horario. Y otro que mencionas es cuando tú tienes una retroalimentación, de repente tienes algo que preguntar o consultar, no está el profesional de manera inmediata. ¿Crees tú que hace falta algún tipo de soporte en ese proceso?

**e:**Sí, sí hace falta un soporte de todas maneras.

**E:**Sí. Ok, y ya. Yo creo que eso sería todo, —---. Muchísimas gracias por tu participación y la información que nos has proporcionado nos va a servir muchísimo para nuestro estudio.

**e:**Gracias, excelente, y muy éxitos en su estudio.

**D22: Usuario_Profesional_Salud**

**E:**Ok, entonces para continuar, bueno para iniciar la entrevista voy a necesitar que por favor me digas tu nombre completo, tu profesión y bueno comenzaríamos.

**e:**Mi nombre completo es —-----, soy enfermera

**E:**Ok, ok —----, entiendo que tú has pasado por el servicio de salud mental virtual, ¿verdad?

**e:**Sí, por la Uson, que brinda la Uson

**E:**¿Puedes comentarme un poco como ha sido ese servicio? ¿Hace cuanto lo has recibido?

**e:**Ha sido más o menos el 2021

**E:**¿Más o menos de que fecha?

**e:**¿Inicio? En mayo, a partir de mayo o junio. Y yo solicité el servicio para que me ayuden a superar el proceso de luto de mi papá

**E:**Ok, entonces entiendo que tú te comunicaste por correo, por teléfono, ¿cómo fue?

**e:**Ellos tienen este, se solicita una cita, valga la redundancia, a un número. Entonces ellos te responden y te...

**E:**¿Pero tú lo escribiste por WhatsApp?

**e:**Por WhatsApp. Y por WhatsApp nos agendan una cita y la cual el psicólogo o psicóloga, en este caso fue psicólogo, nos crean una reunión Meet o Zoom y ahí se hace la atención

**E:**Ok, entonces entiendo que tú habías pasado por una situación difícil, requerías la atención ¿Cómo te enteraste de este número?

**e:**Lo publican por los correos institucionales.

**E:**Ah ok, por publicidad digamos institucional de los correos del INS, ahí encontraste el número y escribiste solicitando digamos la atención ¿Qué tan sencillo fue solicitar la atención?

**e:**Fue sencillo, es escribir un número y...

**E:**¿Y qué tan sencillo fue agendar la atención?

**e:**También fue sencillo

**E:**¿Cuándo se realizó la sesión? Digamos, si tú lo agendaste un día lunes, ¿Cuánto más o menos demoró?

**e:**En el transcurso de la semana

**E:**En la misma semana, o sea si fue un lunes, el día miércoles aproximadamente ya estabas recibiendo la atención

**e:**Sí

**E:**Ok, fue rápido ¿Cómo fue?

**e:**¿Cómo, ¿cómo fue?

**E:**La atención. Estabas aquí en CENSOPAS, estabas en tu casa

**e:**No, era en el horario de trabajo del psicólogo, estaba acá en la oficina

**E:**¿En horario laboral?

**e:**O sea, él nos agenda la cita en su horario laboral

**E:**Ok, ¿y tú estabas?

**e:**Yo estaba acá

**E:**¿Aquí en CENSOPAS? Laborando

**e:**Sí, Laborando. Porque era un horario de oficina y las sesiones son una vez por semana.

**E:**Ok, ok, una vez por semana, entonces no lo viste solo una vez

**e:**No, fueron varias sesiones.

**E:**Más o menos, ¿cuántas?

**e:**Tres, cuatro sesiones- Cuatro sesiones

**E:**Ok, todas las sesiones fueron por Meet

**e:**Por Meet

**E:**Ok, ¿Cuánto más o menos duraba?

**e:**Una hora

**E:**¿Todas?

**e:**Todas son sesiones, eran sesiones de una hora. De una hora Estando aquí en el trabajo

**E:**Ajá Ok ¿Tu experiencia? ¿Te gustó, no te gustó las sesiones? ¿Cómo fue?

**e:**Sí, este, a pesar de ser una teleconsulta, es muy importante, sobre todo que el profesional tenga la cámara encendida. Así

**E:**¿Tú tenías el equipo para poder ver?

**e:**Claro, mi laptop personal

**E:**Ah, tú tuviste que traer tu laptop

**e:**Sí, mi equipo personal para poder conectar. Porque acá no tienen cámara. La metodología que usó es, bueno, de acuerdo, es personalizada por cada situación, entiendo, sí. Sí me ayudó

**E:**Pero, ¿cómo te sentiste tú al inicio, digamos? ¿Ya habías llevado antes una atención así virtual? ¿Es la primera vez?

**e:**Ha sido la primera vez Porque normalmente habré ido a, siempre ha sido antes de la pandemia, todo ha sido presencial

**E:**¿Y qué esperabas, digamos, de la atención? ¿Cómo iba a ser? ¿Tenías alguna expectativa antes de llevarla?

**e:**Mi expectativa era que sí me ayude, y sí me ayudó porque, bueno, la metodología que usó el psicólogo es muy buena. Al inicio te dio apertura para que un poco vayas mostrando toda la información que necesitas, ¿no?

**E:**Ok, y digamos, ¿podrías describirme un poco más esa metodología a la que me comentas?

**e:**A ver, este, primero él te pregunta, ¿Cuál es el motivo de la consulta? Entonces le explicas, en mi caso fue que tengo como un nudo en la garganta. Me siento, él me pregunta qué es lo que yo sentía, entonces yo le decía, me siento más físico y me siento más emocional. Y yo le decía, de acuerdo a mi formación también un poco le explicaba que lo relacionaba a lo que es mi papá. Y otros problemas familiares que tengo con mi hermano y lo cargada que me sentía. Por lo que yo soy la cabeza de la familia en este momento, por ser hermana mayor y todo eso. O sea, fue una etapa de... la primera sesión fue una etapa de que yo le manifieste por qué acudía él Y de parte de él que explore todo lo... el contexto, analice el contexto

**E:** Entonces lo que te agradó entiendo fue de que te permitiera explicar de manera detallada el problema, el motivo...

**e:**Y con una voz calmada, creaba un ambiente agradable, su voz transmite tranquilidad. Y así continuamos la fluidez de la sesión, pero hay un factor interviniente porque es en mi horario de oficina. Y tenías que buscar un lugar donde no hacían bulla y a veces se escuchaba la bulla. Yo también trataba de buscar, y lo mismo, pasaba el trabajo de buscar un lugar donde no había mucha bulla para que se pueda llevar la sesión de la mejor manera

**E:**Claro, dirías que algo que se complicaba la sesión era que pese a que la facilidad de poder utilizar los recursos virtuales, no necesitaban estar en el mismo lugar, sin embargo, al ser en horario laboral. Las propias actividades laborales a veces como que impedían un poco este espacio destinado solamente a platicar ¿Crees que eso influyó un poco en que expresaras como te sentías?

**e:** No mucho, creo que lo manejo bien

**E:**¿Lo supo manejar?

**e:**Si

**E:**¿Qué hizo para manejar eso?

**e:**Su voz era, sin necesidad de gritar, creo que su voz era más firme, levanta un poco la voz sin gritar, sin perder esa calma. Y continuamos

**E:**¿Pese a que se escuchaba bulla por otro lado? Ok, ok. Eso fue la primera sesión, ¿las siguientes sesiones?

**e:**Ah ya, cuando terminaba, la primera sesión ya fue exploratoria, no me dejó ninguna actividad, a partir de la segunda sesión ya me dejaba como tareas.

**E:**Ok, ya, entonces la primera fue exploratoria, digamos, entiendo que pudo haber sido un poco cargada emocionalmente la primera sesión.

**e:**Sí, fue terrible, no, la primera sesión no hice catarsis. La catarsis fue...

**E:**Pero te sentiste cómoda.

**e:**Después de la catarsis, sí, me sentí cómoda porque lloré, me desfogué.

**E:**¿Recuerdas tus sesiones? Tú dices que hayas sido psicólogo antes de manera presencial. ¿Recuerdas tus sesiones presenciales?

**e:**No mucho, porque eran cuando era más joven, era muy reciente.

**E:**Pero ¿podrías comparar un poco las sesiones que tuviste presenciales con las sesiones que has tenido en esta experiencia, digamos, de manera remota?

**e:**Esta experiencia que tuve tuvo una secuencialidad, hubo un inicio, hubo un pico de emoción en la catarsis, luego me dejó tareas, pero no he llegado... Bueno, yo estoy avanzando un poco, ¿por qué no? La diferencia, seguro más adelante te haré una pregunta más específica, la diferencia con las anteriores experiencias, que puedo decir que fueron experiencias sueltas.

**E:**Ok, de una sesión.

**e:**De una sesión. En cambio, ahora sí tuvo un proceso, hubo pasos, tal vez no hemos llegado al final que me dé de alta porque tenía mucha demanda, así como yo, estaba haciendo esta COVID. Hubo mucha demanda de trabajadores, los que requerían sus servicios, entonces seguro había personas que estaban peor que yo.

**E:**Y posterior a ello, ¿llegaste a atenderte con psicólogo de manera presencial?

**e:**No, ya no.

**E:**¿Tampoco virtual?

**e:**No, no, no.

**E:**Ok, ya. Pero para ti, de la experiencia que has tenido, digamos, en estos eventos aislados, de alguna manera las primeras sesiones, los primeros encuentros, ¿consideras tú que la presencialidad, el face to face, te permitía, de alguna manera, entablar una mejor relación, poder sentirte más libre, expresarte?

**e:**Sí, definitivamente, nunca se va a comparar nada al contacto persona a persona, ¿no? Eso siempre va a tener un valor agregado, siempre.

**E:**¿Por qué? ¿Cuál es el motivo?

**e:**Porque, digamos, que puedes sentir esa calidez humana, que a través de una máquina no lo vas a lograr, por más que hagas...

**E:**¿Qué tendría que pasar para que sientas la calidez, digamos, cuando es en persona?

**e:**Por ejemplo, cuando el psicólogo o la psicóloga logra hacer la catarsis con una persona, una persona se quiebra, hay un contacto. No es que te abraza, te estruja tampoco, sino que te puede tocar la mano, te puede tocar el hombro, y eso como que te reconforta.

**E:**Y eso es algo que no se puede dar. ¿En algún momento sentiste que, al momento de llevar esas sesiones remotas, el psicólogo te dio también un poco esa calidez, ese apoyo, ese soporte?

**e:**Sí, en la medida de la posibilidad.

**E:**¿Qué hizo? ¿Qué hizo para que sintieras eso?

**e:**Cuando yo me quebré, no dejaba de mirarme, de tener esa voz calmada, me decían, llora, llora, está bien que llores.

**E:**¿Te daba una especie de feedback? O sea, siempre había una especie de... mencionaba, o sea, no se quedaba en silencio. ¿Crees que eso te ayudó a sentir esa calidez como diría alguien cuando tocarte la mano, tocarte el hombro?

**e:**Sí. Bueno, es eso, sí. Eso es muy importante, ¿no?

**E:**No sería un equivalente, pero ayudaría.

**e:**Exacto. Que haya una voz continuar y calmada, ¿no?

**E:**Claro, que, de alguna manera, pese a no estar presente, con el aspecto, te entiendo, ¿no? Con el aspecto tanto del contacto visual como del feedback con la voz, de alguna manera que sientas que está ahí presente. Ok. Entiendo entonces que tú volverías a llevar teleconsultas.

**e:**Sí, no tendría ningún problema. No tendrías ningún problema. Pero si me dieran a escoger ahora que estamos pos-pandemia en poder tener esas mismas consultas presenciales, yo preferiría, sí, si pudiera, preferiría que sea presencial. Que sea presencial. Exacto. Cosa que ahí buscaremos un lugar más privado.

**E:**Ok, uno por la privacidad, para evitar bulla, ¿no? Ajá. Lo otro, ¿por qué sería?

**e:**Por el contacto.

**E:**Esos dos puntos principales serían, digamos, por los cuales tú, antes que una sesión virtual, preferirías una presencial. Sí. No sería tanto el problema de desplazarte, de ir con el carro, no sería tanto el problema para ti.

**e:**Bueno, en este caso, y bueno, sé que por esos temas tenemos permiso, o sea, con mi papeleta me voy donde está el psicólogo en Chorrillos o ahí en la central.

**E:**Ok. ¿Tuviste algún momento un problema con la conectividad?

**e:**Sí, sabemos que la conectividad acá es pésima, entonces hay problemas en ciertos momentos en que hay baches, en que la comunicación se, lo que ya sabemos, ¿no? Se pone lenta, a veces se corta, se entrecorta la comunicación.

**E:**Y eso también imagino que afectaba un poco, digamos, el vínculo en ese momento. Entonces entiendo que tú usaste tu laptop, te conectaste a la red de aquí, de la institución, igual generaba esos problemas, ¿no? ¿Qué podrías, entonces, de tu experiencia que ahora me has comentado, qué podrías realmente sugerir en próximos, digamos, programas que se establezcan, ¿no? Para hacer este tipo de atenciones. ¿Cuál sería tu sugerencia?

**e:**¿Cómo teleconsulta?

**E:**Claro, ¿cómo teleconsulta? La propuesta es teleconsulta.

**e:**Sí. Este, bueno, el recurso humano es importante. Como te decía, a mí no me han dado de alta. Yo solita me he tenido que dar de alta.

**E:**Ok, la disponibilidad, entonces, de personas que puedan estar allí.

**e:**Sí, pues, porque hay que entender que acá a nivel de inicio somos más de mil trabajadores, entonces es un poco complicado poder atender a todos, ¿no? Entonces, la idea es eso, que inicien y terminen con un trabajador y que le den de alta, ¿no? Ahora también hay casos que ya no están en sus manos y los tienen que derivar, como ha habido con el sacar de compañeros. En el caso mío, me he podido, me siento mejor como estaba, ¿no? Cargada por los problemas que tengo, pero estoy funcionando, ¿no? Soy funcional, creo que se dice. Sí, y los que, sí me ayudan. Si en el caso yo estuviera más complicada, ya hubieran derivado de la correspondencia.

**E:**Ok. ¿Por qué de alguna manera sí volverías a llevarlo? Si te dijeran, te dan la opción, ¿por qué sí lo volverías a llevar?

**e:**Porque me sirvió, tengo una experiencia positiva.

**E:**Ok, es una buena cuestión. Pero aun así no es lo suficiente, si te dicen presencial, para ti es presencial mejor.

**e:**Será que yo soy de la vieja escuela, ¿no?

**E:**¿Qué otra consideración, según tu experiencia, debería tener un programa de teleatención?

**e:**Ah, es importante que, como nuestro derecho a ser atendidos, que cuando sepan que una persona, es que también tiene que ver con la confidencialidad. Porque todavía hay un pequeño estigma en salud mental, ¿no es cierto? No necesariamente acá, lo digo en general. Nadie tiene por qué enterarse de que está recibiendo la atención de salud mental. Aunque sería lo más normal, ¿no? Pero digo, en el caso nuestro, acá en CENSOPAS no hay problema porque tenemos otro enfoque. Pero tal vez con esos tipos de trabajadores pudieran tomar a mal que otro compañero esté recibiendo la atención. ¿Ha sido en tu caso? Siempre es que hay una manera de... A lo que voy es lo siguiente, que cuando estamos en esa atención, por ejemplo, acá yo no tengo privacidad porque me tocan la puerta por una y otra cosa. Entonces, si no estoy acá me buscan donde sea. Yo a donde me iba, me iba a audiometría. Entonces, nadie tiene por qué interrumpirte en ese espacio. No sé si está recibiendo atención.

**E:**O sea, entendería que sería un poco también el tema, digamos, de facilidades de la propia estructura laboral. Para que, si te vas a atender, tengas un espacio en donde puedas atenderte.

**e:**Y que nadie te interrumpa.

**E:**Claro, interrumpir. Pero lo otro que mencionaste también era el tema de que se enteren. Para ti entonces es importante que no se enteren que llegues.

**e:**Sí, porque no todos estamos preparados para... no todos tenemos la misma capacidad para entender que la salud mental es importante y que no necesariamente tú acudes a un servicio porque estás loco. Eso no todos lo manejamos a este mismo nivel. Por eso lo dije.

**E:**¿Tú consideras que eso pudo haber pasado?

**e:**¿En mi caso? No. Pero en caso de otras personas, sí. Pues, es probable.

**E:**¿En qué otros contextos pudieron haber pasado?

**e:**¿Cómo que en otros contextos?

**E:**Tú me dices que consideras que, en otras personas, otros contextos, pudo haber pasado. ¿En cuál es, por ejemplo?

**e:**Este... no, es que no te puedo dar ese ejemplo porque acá, bueno, al menos entre nosotros, pero en muchos no hay. No sé, otras...

**E:**No, la pregunta es porque quisiera saber un poco más puntualmente si tú has observado, no necesariamente en la institución aquí, sino en otros lugares de experiencia laboral, de repente, en centros de salud, colegios... En donde tú hayas podido escuchar, ver de casos en donde por saber qué antes están llevando...

**e:**Sí, por eso te digo, por saber que alguien está llevando...

**E:**¿En qué casos? Este... ¿En qué instituciones? O más o menos qué rubro...

**e:**Cuando eres estudiante, por ejemplo... ¿En la universidad? En la universidad. Ok.

**E:**Entonces, este... Pero en el ámbito laboral. Laboral, laboral...

**e:**No, no, laboral no tanto. Pero sí he escuchado en otras personas que, ah, sí, ese está loco, ese está recibiendo atención psicológica porque algo tiene...

**E:**La universidad. Pero en el caso, digamos, de atención laboral, de salud mental, no, no perdieron mucho eso.

**e:**No. Ok. Lo digo, ¿no?

**E:**Pero es una sugerencia válida.

**e:**Porque sí se crea el estigma, porque lo he visto en otros lados, he escuchado.

**E:**Ah, sí, sí, es una sugerencia totalmente válida. Se lo he consultado para tener más claro la información. Bueno, eso sería todo por mi parte. No sé si querías comentar algo más respecto.

**e:**No, no, no está interesante.

**E:**Ya, ok. Muchas gracias, entonces.

**D23: Usuario_Profesional_Salud**

**E:**Buenas tardes,, bióloga del Centro Nacional de Salud Ocupacional y Protección de la Mente para la Salud. Gracias por aceptar y dar tu consentimiento para participar en esta entrevista del estudio denominado Diseño y evaluación de usabilidad, aceptabilidad y satisfacción de un servicio de telesalud de tamizaje, manejo inicial y derivación oportuna en salud mental para trabajadores de grupos ocupacionales vulnerables con antecedentes COVID-19 en Lima 2022, proyecto ganador de prociencia y aprobado por el Comité de Ética del Instituto Nacional de Salud. Bien, como te comenté minutos antes, las preguntas van a estar orientadas sobre tu experiencia en el uso de plataformas de telesalud de cualquier tipo que hayas sido durante el contexto de pandemia. Entonces, como primera experiencia tenemos, ¿cómo ha sido tu experiencia en todo el proceso de atención por teleconsulta que has tenido? Por ejemplo, en la forma en la que has buscado tu acceder a una teleconsulta, ¿cómo sacaste cita? ¿Cómo llegaste a acceder a un servicio de telesalud?

**e:**Bueno, por el tema del COVID, te registraban los de epidemiología y ellos mismos hacían el seguimiento a cada paciente. Imagino que por eso me llamaron, después de tres meses. Imagino que de esa manera, porque dejé mis datos, la ficha epidemiológica. La otra fue por Essalud, que me estaba atendiendo en el otorrino presencialmente, pero como vio que no era tan necesario que yo acuda en persona, me dijo que la siguiente vez iba a ser con llamada, nomás, telefónico. Y así me dio mi cita para que me hagan una teleatención.

**E:**¿Te solicitaron algún requisito previo antes de brindarte las teleconsultas o atenciones online?

**e:**No.

**E:**¿Tuviste que tener algún tipo de documentación, algún tipo de identificación al momento de acceder a tu consulta?

**e:**No.

**E:** Si quieres hacerle una valoración, por ejemplo, en tu experiencia, de las veces que tuviste la teleatención, ¿cómo ha sido en general?

**e:** ¿Valoración? Bueno, para la del COVID me pareció muy tarde el hecho de que me hayan llamado después de tres meses. Y en la de salud, bueno, me limité a aceptar la llamada, que sí fueron puntuales para la hora que me dijeron. Todo cumplieron con darme la cita virtual por llamada.

**E:**En ese punto, por ejemplo, nos estabas comentando que es un aspecto positivo de estas teleatenciones que han sido puntuales. ¿Consideras que hay otros aspectos positivos en tu experiencia de haber recibido teleatenciones destacables para ti?

**e:**Bueno, el hecho de no tener que ir hasta el hospital a hacer cola, esperar un ratazo, hasta que te atiendan, a ver si te atienden. Me pareció, la teleconsulta, para que sea puntual en una hora, me pareció genial. No estabas convencida de que a qué hora te llamarán, no. Te dieron la cita y te llamaron. Claro. Que no sucede en una presencial.

**E:**Y en caso contrario, por ejemplo, ¿qué aspectos de la teleatención no te gustaron o te gustaría que fueran diferentes?

**e:**Como que, dependiendo de la enfermedad, a veces necesitas que te ausculten. Y eso no puede ser por una llamada, ni por video. A veces el paciente puede hablar y no se escucha bien, o se corta la señal. Puede pasar eso. Esa sería la parte negativa.

**E:**De lo que hemos venido hablando, ¿hay algún aporte más respecto a una percepción negativa o positiva de tus atenciones en tu experiencia? En general, ¿qué cosas te gustaron puntualmente y qué cosas no te gustaron?

**e:**Me gustó que fueran puntuales, a la hora del día que me dijeron. Que no tuviera que esperar, como te digo, como cuando haces la cita presencial. Que no me gustó... La tardanza, que no me gustó que fuera demasiado tarde. Bueno, en el COVID ya era comprensible con tanto paciente, ¿no? Pero aún así, si hubiera estado mal, no habría para qué contarlo. Así que bueno, se dieron el tiempo de llamar. Eso se considera.

**E:**Retomando entonces la experiencia, en ambas situaciones me dijiste que fue por teléfono. ¿Recibiste las llamadas a través de algún aplicativo como WhatsApp, Messenger, o fueron llamadas directamente por teléfono?

**e:**Llamadas por teléfono. Acabo de recordar que una desventaja sería que me llamen de un celular, pero como yo no atiendo a los números desconocidos, pude haber descartado o no haber contestado. No me pasó eso, pero... Porque creo que esos días, las fechas que me llamaron yo he estado como pendiente. Pero sí es probable que haga eso si me llaman sin una previa coordinación, una previa cita.

**E:**En el caso del COVID no lo esperabas. Pero contestaste...

**e:**Sí, creo que aquella vez todavía contestaban. Porque ya últimamente ya no contestan. Porque hay tantos que te llaman y te llaman y son de teléfono, de empresa, bancos, qué sé yo. O a veces devuelven la llamada y no existe ese número, entonces por la desconfianza no. Pero si hay una previa coordinación sería genial.

**E:** Y me comentaste que pasó mucho tiempo para que tengas tu primera atención con el COVID. ¿Después de esa primera atención hubo seguimiento?

**e:**No. Porque ya estaba sana para ese tiempo.

**E:**¿Hubo una condición de alta o algo por el estilo cuando ustedes...

**e:**Justamente era para que me dieran el alta. Ah, ya. Para que ellos en su registro aparezca como de alta.

**E:**Y ese era el cierre del proceso de atención.

**e:**Sí.

**E:**Y en el caso de Otorrino, me comentaste que estuviste en cita presencial y el doctor te comentó que en adelante iban a ser por teleatención.

**e:**Sí.

**E:**¿Te dio algún documento, algún papel que te diera la fecha en la que se te iba a llamar?

**e:**La verdad no recuerdo. Me comentó, me dijo saca tu cita y... Imagino que puso... En Essalud no te dan médico la cita, sino vas al módulo de citas y ahí ves. Entonces me imagino que internamente ellos han puesto que se le va a dar telellamada, no sé.

**E:**¿Teleatención?

**e:**Teleatención, sí. Y la cosa es que me dieron, no recuerdo bien, pero sí.

**E:**Pero tú te acercaste a sacar la cita. Ah, ya. Entonces contaste con un documento que decía qué día te tocaba. Un ticket, tal vez.

**e:**No, lo que Essalud hacen, al menos en el Rebagliati, por ejemplo, los 18 de cada mes o de ese mes, se sacan citas para Otorrino. Entonces tú tienes que llamar, bueno, si es que haces tu llamada, sacas cita ese día, no te hacen otro día. O si vas presencialmente te dicen espérate, está el 18. Pero yo la saqué creo que ese mismo día, no recuerdo bien, pero...Uno busca la cita, la próxima cita, no es que el médico te diga.

**E:**Ah, ok, solo te avisó que en adelante tú eras candidata para hacerte la atendida.

**e:**Pero ahora, por ejemplo, que he vuelto, bueno, me falta otra vez reintentar. No sé si me van a atender presencial o por teléfono. Porque ya como me agotaron que ya no me van a.… como que no tiene solución. No sé cómo que... no me evitan, no es tan grave lo que tengo. No sé si la próxima vez que yo saque, porque estoy que intento hasta ahorita tener cita, me van a dar por llamada o presencial, no lo sé.

**E:**Y cuando es por llamada, o sea, no pactas una fecha y un horario específico, también ellos te llaman en cualquier momento.

**e:**No, ellos te dicen. Cuando tú sacas cita te dicen tal día y tal hora te van a llamar. Estate atento.

**E:**Y si se cumple en ese caso.

**e:**De la vez que me pasó, si se cumplió como te digo, ¿no?

**E:**Y has tenido en paralelo alguna otra experiencia, por ejemplo, por videollamada u otro tipo de, de repente, de atención, de la atención privada.

**e:**Privada, sí, las terapias psicológicas, que las estoy haciendo prácticamente virtuales.

**E:**Cuéntanos un poco de esa experiencia, ¿no? ¿Cómo ha sido tu experiencia en general? ¿El valor que le das a esa atención? ¿Y cómo ha sido el proceso?

**e:**Mira, por ejemplo, cuando yo estaba muy deprimida, el hacer una teleconsulta, o sea, una consulta virtual, no me ayudaba porque yo me sentía muy sola. Entonces, la primera vez se me iba presencial. Las siguientes las hice virtuales, por un tema de costos también, porque las presenciales te cobran más y las virtuales menos. Cuando me fui a mi casa en provincia por un tratamiento, me convenía tener la terapia virtual, obviamente, ¿no? Este, por la distancia y todo, porque ya estaba acompañada con mi familia, en fin, tenía un soporte. Pero estando sola no, este, no me ayudaba, no. Y además lo que se recomienda con las terapias psicológicas es que mejor sean presenciales, como ahora después del COVID se ha dado la modalidad de que sean virtuales, pues serán virtuales.

**E:**Y cuéntanos un poco sobre tu experiencia para acceder a terapias o atención psicológica por teleatención. ¿Fue difícil conseguir una cita? ¿Cuál fue el medio de, digamos, pactar la fecha, horario y plataforma?

**e:**Generalmente usan WhatsApp, WhatsApp o Meet. Este, después de que tú te comunicas y haces el pago previo, te mandan el link y ya, te conectas.

**E:**¿Hace WhatsApp para llamadas o para videollamadas? Videollamadas, todo es video, siempre es video.

**e:**Y en caso de que el Meet no funcionaba, te daban la opción de WhatsApp. Bueno, no me pasó hasta ahorita.

**E:**Ajá, ok. Y como resumen, ¿qué es lo que más te gustó de este tipo de atención, por ejemplo, por videollamada?

**e:**De todo ese servicio. De que es práctico, o sea, sirve yo creo que para cuando no estás tan grave o no tienes algo que requiera que el médico te chequee. Este, para esos casos está bien creo porque te ahorras el tiempo en ir hasta el hospital o no sé dónde te atiendan, esperar, gastar pasaje, que es el tiempo. Por ese lado, es bueno las citas virtuales. Pero como te digo, siempre y cuando no sea algo que requiera la presencialidad.

**E:**Y, por último, ya englobando todas estas experiencias, habernos recordado todo, que has usado distintas plataformas, has tenido modalidades de seguimiento y modalidades de atención propiamente dichas, ¿qué crees que debe tener un servicio de telesalud para que este sea óptimo y te sientas cómoda con ese servicio?

**e:**¿Qué debe tener? Bueno, que te den a conocer de qué número te van a llamar para que esperes. Que te den las citas, que sean puntuales en el momento que te llamen. Simplemente lo son.

**E:**Es en el caso de las llamadas, ¿no? Y en el caso, por ejemplo, de las videollamadas.

**e:**También, también. Suelen ser muy difíciles, pero es lo que suele ser. Y también deben ser puntuales. O sea, ellos también están con sus tiempos. Porque por lo que veo, hay muchas personas que acuden y después de que termine mi cita, la siguiente hora ya está inmediatamente otra persona esperando. Entonces ellos también tienen que cumplir sus horas. A veces cuando no hay nadie más después de ti, se puede explayar un poquito más la cita. Pero si no, son bien puntuales.

**E:**Interesante. Por ejemplo, en el tiempo que nos dices, ¿Cuánto tiempo crees que es lo prudente como para este tipo de atención psicológica virtual?

**e:**Hasta una hora. Hasta una hora. Pero tengo uno, por ejemplo, que es 40 minutos. Exacto. Hay otros que, si me ha demorado la hora, 45 minutos. Pero ellos, como te digo, si es que tienen varios pacientes, tratan de diseñarse su horario y programar su actividad para ese horario.

**E:**Pero ¿una hora qué es? ¿Qué sí se podría conversar de muchas cosas?

**e:**Hasta una hora sí.

**E:**Ya estás hablando de un enfoque de atención psicológica.

**e:**Sí.

**E:**En salud, por ejemplo, cuando comentaste que tuviste atenciones en Otorrino, ¿Cuánto era la duración promedio y te sentías cómoda con esa duración?

**e:**Bueno, me preguntó varias cosas. ¿Cuánto las duraba? Quizás, por lo mucho, 10 minutos. No demoró mucho, creo. No recuerdo. Es que, en la terapia psicológica, en psiquiatría, esos sistemas necesitas mucho tiempo para conversar.

**E:**¿Para la atención Otorrino, crees que fue el tiempo suficiente?

**e:**Creo que sí. Como te digo, no es una cosa grave que yo tenga. Me preguntan las cosas básicas. Ya saben los resultados de mis análisis, porque antes ya me los han hecho presencialmente, ya me los han chequeado. Más que todo es para que te dé tu medicación, si sigues así o no sigues, cómo te sientes. Pero no es para que se explique.

**E:**Y en el ámbito de la atención remota, por ejemplo, enfocándonos en la atención psicológica, si tuvieras que elegir entre un medio que para ti es más cómodo, ¿sería el teléfono o sería la videollamada?

**e:**No, videollamada. El teléfono no funcionaría. El psicólogo tiene que mirarte, tiene que ver cómo estás. Con teléfono, no. El médico quizás por teléfono. Y, aun así, no. Pero mejor es con videollamada.

**E:**Hemos hablado, nos comentaste, de que tiene que ser claro el proceso de sacar una cita, horarios claros, tener un link, donde sé dónde va a ocurrir mi cita. Hemos hablado también un poco de que tiene que ser de preferencia videollamada, horarios claros, una duración tal vez de 40 minutos a una hora, para que de repente no sea agotador.

**e:**Dependiendo del tipo de atención. O sea, la psicológica y siquiera la cita te demanda tiempo. Pero las otras médicas, puede ser menos, quizás.

**E:**Y tal vez tocando la última fase de una atención, que sería cuando ya estás cerca al alta o al alta, ¿Cuáles crees que deberían ser las características que debe tener un servicio de teleatención para finalizar adecuadamente la atención con la paciente?

**e:**Me imagino que le iba su próxima cita a esa fecha, o sea, dependiendo de cómo es. Para saber cómo está, porque hay que hacer test... Bueno, tú dices que ya sería la última, ¿no? Pero es que no sé, pues, porque generalmente uno va por una próxima cita porque necesitan todavía hacer seguimiento.

**E:**Digamos que vas por un aspecto puntual. ¿Qué te gustaría obtener como cierre de tu procesada de atención? Tal vez un certificado, una constancia...

**e:**Ah, bueno. Te mandan la receta, si es que es particular, lo recibo por honorario. Esos dos, básicamente, para que cierre.

**E:**Con eso te sentirías como finalizando tú, digamos, el periodo de atención que has tenido.

**e:**Sí, siempre que sea particular, o sea, si fuera de la salud... Bueno, te mandan la receta. Siempre te la mandan virtualmente.

**E:**O de repente el proceso de derivación, de repente requieres una derivación... Pero él dice que ya es la última. Claro, digamos que es la última, es lo que estábamos hablando ahora. En otro ejemplo, digamos, que el profesional de salud considera que te va a derivar, ¿Qué características debería tener esa derivación para que tú consideres que es óptima?

**e:**No sé... La amabilidad, o no sé...Agradecer por la confianza en mí...

**E:**Igualmente, tal vez, que sean claras las fechas, las citas, en qué horario...

**e:**Pero si ya no va a haber otra atención, yo no le pediría eso.

**E:**No, derivación, una derivación.

**e:**Ah, si te refieren, como dicen, la referencia. No te podría decir eso, o sea, el médico no te podría decir eso, porque... En el Essalud me han referenciado, otro médico, pero eso fue presencial. Pero sí me dio... Ella misma te dio la cita. No. Tengo que sacar citas siempre.

**E:**Ah, ya. ¿Consideras que eso es adecuado, o te gustaría que eso estuviera incluido dentro de tu atención?

**e:**O sea, es que depende lo particular con lo del estado de salud. Claro. Si es particular te lo van a decir, te cito en un mes, en 15 días, qué sé yo... Si es el estado, a tu suerte, el de salud, ¿no? Tienes que sacar tus citas, caballero.

**E:**Ah, ya. Porque tienes que esperar que haya tiempo, recursos, atención.

**e:**Pero esto te estoy hablando por la presencial que tú dices, a lo que me refirieron. Pero, ¿referencia? ¿Con videollamada que me referencien en Essalud?

**E:**No, sin referencia.

**e:**No, no creo que... Tendría que ser presencial. O sea, tiene que ser algo más grave para que te puedan referir. Como te digo, creo que las... Porque el médico me envió la llamada, porque no vio que necesitaba... O no era tan grave y no era tan necesario que yo acuda presencialmente. Porque ya me chequearon todo antes, con análisis y todo. Entonces ya no necesito. Solo es un control. Entonces, por eso te digo, las llamadas serían, si es que no es muy grave. Si es que es algo que requiere presencialidad. Sí, tienes que estar ahí, por la gravedad. Eso define, si es videollamada, o sea, teleconsulta o es presencial. La gravedad.

**E:**Y en el caso, por ejemplo, de lo psicológico, digamos que inicias el proceso con una atención de tamizaje. Para identificar si realmente necesitas o no pasar un tratamiento psicológico formal. Una terapia psicológica. ¿Te parecería adecuado que ese proceso de tamizaje ocurra de forma remota, en teleatención?

**e:**Sí. Solo es tamizaje. Ahora, si tú acudes, ya depende de tu bolsillo. ¿Quieres presencial o quieres virtual?

**E:**Claro, ¿Cómo va a ser la terapia?

**e:**O cómo quieres que te atienda. Entonces, yo quiero que sea presencial. Y vas y te hacen tu evaluación presencialmente. Pero si es como tú dices, un tamizaje que no lo he visto, un tamizaje creo que puede ser virtual.

**E:**¿Tienes otra pregunta? Bueno, con eso, esas eran nuestras últimas preguntas. Pero si tienes aquí un momento de repente de si quieres agregar algo adicional. Siempre pensando en tu experiencia, en uso de estos tipos de atenciones nuevas durante la pandemia. ¿Cómo se adaptan ahora? ¿Cómo deberían ser para ti?

**e:**Creo que pueden implementarse cuando no es tan crítica la situación. Que sí ayuda de alguna manera. O sea, ya no tienes que irte hasta el hospital. Si es que no es algo muy grave, por supuesto. Después sí, pues, necesitas.

**E:**¿Tienes algún tipo de consejo? Listo. Muchas gracias —--- por la entrevista.
