# Supplementary material for: Barriers and limitations to the development of a telemental health service for workers in Peru- A user-centered approach
Source: PLoS One. 2025 Apr 9;20(4):e0321401. doi: 10.1371/journal.pone.0321401 (PMC11981184; doi:10.1371/journal.pone.0321401)
Supplement: Attachment — Submitted filename: RESPONSE LETTER.pdf [file pone.0321401.s005.pdf]

RESPONSE LETTER

Subject: Response to the observation on Dr. Jonh Maximiliano Astete Cornejo´s inclusion in the research team

Dear editor

I would like to thank for the feedback provided in your previous email. In reference to the inclusion of dr. Jonh Maximiliano Astete Cornejo in our research team as an investigator, I would like to clarify the following point:

As indicated in the submission, Dr John Astete Maximiliano Cornejo also referred to as "Jonh Astete Cornejo", serve as the principal investigator and is been listed as the first author in the PDF submission. For your convenience, I have attached a screenshot that corroborates this information.

Therefore, we kindly request a reconsideration of the suggested change, taking into account the clarification provided. Your attention to this matter is greatly appreciated.

Thanks for your information and support

Kind regards

Cruz-Ausejo Liliana

[illegible]
and facilitators related to mental telehealth services through a semi-structured interview guide. The interviews were recorded and transcribed verbatim, furthermore, a thematic analysis was done to identify key themes.

Results

The research identified barriers and limitations to developing a tele-mental health screening service based on the experiences of workers, some of them were related to user dissatisfaction emphasizing the need for personalized solutions beyond technical aspects. Scheduling issues call for flexibility and improved communication. Healthcare professionals' varied experiences highlight the necessity for targeted training, while successful telehealth integration demands addressing resource limitations through a comprehensive approach.

Conclusion

The study advocates for a holistic, user-centred paradigm in telehealth implementation, addressing both technological aspects and human and systemic elements. Multifaceted challenges inherent in telehealth, particularly in Peru, emphasize the need for strategic interventions by stakeholders. The study calls for a policy shift towards enhancing telehealth equity through system-level changes and eliminating structural barriers for marginalised populations

**Order of Authors:**	Jonh Astete Cornejo, Dr.
	Liliana Cruz- Ausejo, MSc
	Jimmy Cainamarks Alejandro, BSc

Powered by Editorial Manager® and ProduXion Manager® from Aries Systems Corporation

	Miguel Burgos Flores, Msc
	Juan Ambrosio Melgarejo, BPsyc
	Jaime Rosales Rimache, Dr.
	Sebastian Daniel Garcia Cruz, BSc
