# Supplementary material for: Barriers and limitations to the development of a telemental health service for workers in Peru- A user-centered approach
Source: PLoS One. 2025 Apr 9;20(4):e0321401. doi: 10.1371/journal.pone.0321401 (PMC11981184; doi:10.1371/journal.pone.0321401)
Supplement: Attachment — Submitted filename: PONE-D-23-44037.pdf [file pone.0321401.s006.pdf]

PLOS ONE

Barriers and limitations to the development of a tele-mental health service for workers - A user-centered approach

--Manuscript Draft--

**Manuscript Number:**	PONE-D-23-44037
**Article Type:**	Research Article
**Full Title:**	Barriers and limitations to the development of a tele-mental health service for workers - A user-centered approach
**Short Title:**	Barriers and limitations for tele-mental health service
**Corresponding Author:**	Liliana Cruz- Ausejo, MSc<br>INS: Instituto Nacional de Salud<br>Lima, Lima PERU
**Keywords:**	telemedicine; mHealth; health services; health personnel (MeSH terms)
**Abstract:**	Introduction<br><br>Over the past decade, the surge in digital healthcare services has brought about a transformative shift from traditional healthcare, necessitating multidisciplinary engagement to adapt to the digital realm. The global rise of telehealth, particularly amid the COVID-19 pandemic, has been widely embraced, but its implementation in Peru faces unique challenges, including infrastructure issues and economic constraints. So, this research aims to identify the barriers and limitations for the future development of a tele-mental health Service for screening, evaluation and timely referral, based on the experiences of workers as teachers, policemen and also from healthcare workers who provide the service and the decision-makers at the top<br><br>Material and Method<br><br>A qualitative study was undertaken. We adopted a phenomenological approach, utilizing semi-structured interviews with vulnerable occupational groups and decision-makers. We conducted 23 interviews with health professionals (users and attendance), teachers, police officers and decision-makers; exploring experiences, needs, barriers, and facilitators related to mental telehealth services through a semi-structured interview guide. The interviews were recorded and transcribed verbatim, furthermore, a thematic analysis was done to identify key themes.<br><br>Results<br><br>The research identified barriers and limitations to developing a tele-mental health screening service based on the experiences of workers, some of them were related to user dissatisfaction emphasizing the need for personalized solutions beyond technical aspects. Scheduling issues call for flexibility and improved communication. Healthcare professionals' varied experiences highlight the necessity for targeted training, while successful telehealth integration demands addressing resource limitations through a comprehensive approach.<br><br>Conclusion<br><br>The study advocates for a holistic, user-centred paradigm in telehealth implementation, addressing both technological aspects and human and systemic elements. Multifaceted challenges inherent in telehealth, particularly in Peru, emphasize the need for strategic interventions by stakeholders. The study calls for a policy shift towards enhancing telehealth equity through system-level changes and eliminating structural barriers for marginalised populations
**Order of Authors:**	Jonh Astete Cornejo, Dr.
	Liliana Cruz- Ausejo, MSc
	Jimmy Cainamarks Alejandro, BSc

Powered by Editorial Manager® and ProduXion Manager® from Aries Systems Corporation

	Miguel Burgos Flores, Msc
	Juan Ambrosio Melgarejo, BPsyc
	Jaime Rosales Rimache, Dr.
	Sebastian Daniel Garcia Cruz, BSc
**Additional Information:**	
**Question**	**Response**
**Financial Disclosure**<br><br>Enter a financial disclosure statement that describes the sources of funding for the work included in this submission. Review the submission guidelines for detailed requirements. View published research articles from *PLOS ONE* for specific examples.<br><br>This statement is required for submission and **will appear in the published article** if the submission is accepted. Please make sure it is accurate.<br><br>**Funded studies**<br>Enter a statement with the following details:<br>• Initials of the authors who received each award<br>• Grant numbers awarded to each author<br>• The full name of each funder<br>• URL of each funder website<br>• Did the sponsors or funders play any role in the study design, data collection and analysis, decision to publish, or preparation of the manuscript?<br><br>Did you receive funding for this work?	Yes
Please add funding details.<br>as follow-up to "**Financial Disclosure**<br><br>Enter a financial disclosure statement that describes the sources of funding for the work included in this submission. Review the submission guidelines for detailed requirements. View published research articles from *PLOS ONE* for specific examples.<br><br>This statement is required for submission	Funding: This work was funded by the Agencia Española de Cooperación Internacional para el Desarrollo (AECID, in Spanish) by Programa Nacional de Investigación Científica y Estudios Avanzados- CONCYTEC- Perú, under contract: N° 076-2021-PROCIENCIA.

and **will appear in the published article** if the submission is accepted. Please make sure it is accurate.<br><br>**Funded studies**<br>Enter a statement with the following details:<br>• Initials of the authors who received each award<br>• Grant numbers awarded to each author<br>• The full name of each funder<br>• URL of each funder website<br>• Did the sponsors or funders play any role in the study design, data collection and analysis, decision to publish, or preparation of the manuscript?<br><br>Did you receive funding for this work?"	
Please select the country of your main research funder (please select carefully as in some cases this is used in fee calculation).<br>as follow-up to "**Financial Disclosure**<br><br>Enter a financial disclosure statement that describes the sources of funding for the work included in this submission. Review the submission guidelines for detailed requirements. View published research articles from *PLOS ONE* for specific examples.<br><br>This statement is required for submission and **will appear in the published article** if the submission is accepted. Please make sure it is accurate.<br><br>**Funded studies**<br>Enter a statement with the following details:<br>• Initials of the authors who received each award<br>• Grant numbers awarded to each author<br>• The full name of each funder<br>• URL of each funder website<br>• Did the sponsors or funders play any role in the study design, data collection and analysis, decision to publish, or preparation of the manuscript?	PERU - PE

Did you receive funding for this work?"	
**Competing Interests**<br><br>Use the instructions below to enter a competing interest statement for this submission. On behalf of all authors, disclose any competing interests that could be perceived to bias this work—acknowledging all financial support and any other relevant financial or non-financial competing interests.<br><br>This statement is required for submission and **will appear in the published article** if the submission is accepted. Please make sure it is accurate and that any funding sources listed in your Funding Information later in the submission form are also declared in your Financial Disclosure statement.<br><br>View published research articles from *PLOS ONE* for specific examples.<br><br>**NO authors have competing interests**<br><br>Enter: *The authors have declared that no competing interests exist.*<br><br>**Authors with competing interests**<br><br>Enter competing interest details beginning with this statement:<br><br>*I have read the journal's policy and the authors of this manuscript have the following competing interests: [insert competing interests here]*<br><br>* typeset	The authors have declared that no competing interests exist.
**Ethics Statement**<br><br>Enter an ethics statement for this submission. This statement is required if the study involved:	The Research and Ethics Institutional Review Board at the National Institute of Health in Peru (Instituto Nacional de Salud- Peru) approved the study protocol under Resolution RD N°206-2023-OGITT/INS, with the assigned code OC-048-2022.

Powered by Editorial Manager® and ProduXion Manager® from Aries Systems Corporation

- Human participants
- Human specimens or tissue
- Vertebrate animals or cephalopods
- Vertebrate embryos or tissues
- Field research

Write "N/A" if the submission does not require an ethics statement.

General guidance is provided below. Consult the submission guidelines for detailed instructions. **Make sure that all information entered here is included in the Methods section of the manuscript.**

**Format for specific study types**<br><br>**Human Subject Research (involving human participants and/or tissue)**<br>• Give the name of the institutional review board or ethics committee that approved the study<br>• Include the approval number and/or a statement indicating approval of this research<br>• Indicate the form of consent obtained (written/oral) or the reason that consent was not obtained (e.g. the data were analyzed anonymously)<br><br>**Animal Research (involving vertebrate animals, embryos or tissues)**<br>• Provide the name of the Institutional Animal Care and Use Committee (IACUC) or other relevant ethics board that reviewed the study protocol, and indicate whether they approved this research or granted a formal waiver of ethical approval<br>• Include an approval number if one was obtained<br>• If the study involved *non-human primates*, add *additional details* about animal welfare and steps taken to ameliorate suffering<br>• If anesthesia, euthanasia, or any kind of animal sacrifice is part of the study, include briefly which substances and/or methods were applied<br><br>**Field Research**<br><br>Include the following details if this study involves the collection of plant, animal, or other materials from a natural setting:<br>• Field permit number<br>• Name of the institution or relevant body that granted permission	
**Data Availability**<br><br>Authors are required to make all data underlying the findings described fully available, without restriction, and from the time of publication. PLOS allows rare exceptions to address legal and ethical concerns. See the PLOS Data Policy and FAQ for detailed information.	No - some restrictions will apply

A Data Availability Statement describing where the data can be found is required at submission. Your answers to this question constitute the Data Availability Statement and **will be published in the article**, if accepted.<br><br>**Important:** Stating 'data available on request from the author' is not sufficient. If your data are only available upon request, select 'No' for the first question and explain your exceptional situation in the text box.<br><br>Do the authors confirm that all data underlying the findings described in their manuscript are fully available without restriction?	
**Describe where the data may be found in full sentences. If you are copying our sample text, replace any instances of XXX with the appropriate details.**<br><br>• If the data are **held or will be held in a public repository**, include URLs, accession numbers or DOIs. If this information will only be available after acceptance, indicate this by ticking the box below. For example: *All XXX files are available from the XXX database (accession number(s) XXX, XXX.).*<br>• If the data are all contained **within the manuscript and/or Supporting Information files**, enter the following: *All relevant data are within the manuscript and its Supporting Information files.*<br>• If neither of these applies but you are able to provide **details of access elsewhere**, with or without limitations, please do so. For example:<br><br>*Data cannot be shared publicly because of [XXX]. Data are available from the XXX Institutional Data Access / Ethics Committee (contact via XXX) for researchers who meet the criteria for access to confidential data.*<br><br>*The data underlying the results presented in the study are available from (include the name of the third party*	Data cannot be shared publicly because of personal information content. Data are available from the National Institute of Health (Peru) Institutional Data Access / Ethics Committee, contact for researchers who meet the criteria for access to confidential data.

Powered by Editorial Manager® and ProduXion Manager® from Aries Systems Corporation

and contact information or URL).<br>• This text is appropriate if the data are owned by a third party and authors do not have permission to share the data.<br><br>* typeset	
Additional data availability information:	

Manuscript
Click here to access/download;Manuscript;00. Manuscrito_Vf_ICA-2812-cualitativo.docx

Barriers and limitations to the development of a tele-mental health service for workers - A user-centered approach

Barriers and limitations for tele-mental health service

Authors

Astete Cornejo, John[1]; Cruz-Ausejo, Liliana[1*]; Cainamarks Alejandro, Jimmy[1;] Burgos Flores, Miguel[1] Ambrosio Melgarejo, Juan[1]; Rosales Rimache, Jaime[1]; García Cruz, Sebastián[2]

Affiliation

1 Centro Nacional de Salud Ocupacional y Protección del Ambiente para la Salud, Instituto Nacional de Salud, Lima, Perú.

2 Pontificia Universidad Católica del Peru, Facultad de Ciencias Sociales

Corresponsal author

Cruz-Ausejo Liliana, rcruzausejo@gmail.com ORCID: 0000-0001-7506-4939

Authors ORCID:

Astete Cornejo, John; jastetecornejo@gmail.com ORCID: 0000-0001-6225-6720

Cainamarks Alejandro, Jimmy; alejandrocainamarks@gmail.com ORCID: 0000-0001-7017-5277

Burgos Flores, Miguel; mabf77@gmail.com 0000-0002-6156-2176

Ambrosio Melgarejo, Juan; jiam0095@gmail.com 0000-0002-8791-802X

Rosales Rimache, Jaime; jaimerosale@gmail.com 0000-0002-1665-2332

García Cruz, Sebastián sebastian.garcia.c@upch.pe   0000-0003-4148-9810

ABSTRACT

Introduction: Over the past decade, the surge in digital healthcare services has brought about a transformative shift from traditional healthcare, necessitating multidisciplinary engagement to adapt to the digital realm. The global rise of telehealth, particularly amid the COVID-19 pandemic, has been widely embraced, but its implementation in Peru faces unique challenges, including infrastructure issues and economic constraints. So this research aims to identify the barriers and limitations for the future development of a tele-mental health Service for screening, evaluation and timely referral, based on the experiences of workers as teachers, policemen and also from healthcare workers who provide the service and the decision-makers at the top **Material and Method:** A qualitative study was undertaken. We adopted a phenomenological approach, utilizing semi-structured interviews with vulnerable occupational groups and decision-makers. We conducted 23 interviews with health professionals (users and attendance), teachers, police officers and decision-makers; exploring experiences, needs, barriers, and facilitators related to mental telehealth services through a semi-structured interview guide. The interviews were recorded and transcribed verbatim, furthermore, a thematic analysis was done to identify key themes. **Results:** The research identified barriers and limitations to developing a tele-mental health screening service based on the experiences of workers, some of them were related to user dissatisfaction emphasizing the need for personalized solutions beyond technical aspects. Scheduling issues call for flexibility and improved communication. Healthcare professionals' varied experiences highlight the necessity for targeted training, while successful telehealth integration demands addressing resource limitations through a comprehensive approach. **Conclusion:** The study advocates for a holistic, user-centred paradigm in telehealth implementation, addressing both technological aspects and human and systemic elements. Multifaceted challenges inherent in telehealth, particularly in Peru, emphasize the need for strategic interventions by stakeholders. The study calls for a policy shift towards enhancing telehealth equity through system-level changes and eliminating structural barriers for marginalised populations

Keywords: Telemedicine, mHealth, health services, health personnel (MeSH terms)

INTRODUCTION

In the last decade, the surge in digital healthcare services online has prompted a vital transformation for traditional healthcare businesses. This shift requires multidisciplinary team engagement and stakeholder involvement to adapt to the digital world, ensuring optimal patient care. The thriving telehealth method, marked by recent activity and substantial investment, is disrupting global healthcare services (1).

Telehealth has emerged globally as a recurring strategy employed by governments and their health systems to ensure uninterrupted care provision amidst the COVID-19 pandemic (2). In the evolving healthcare landscape, practitioners in low and middle-income countries (LMICs) have widely accepted these services (3). In the specific context of Peru, as part of the Latin American region, the COVID-19 pandemic stimulated the rapid adoption of telehealth. However, its implementation in Peru encounters unique challenges, including infrastructure issues, lack of electricity in some areas, limited access to an internet connection, and economic constraints hindering widespread access to electronic devices necessary for telehealth services (4).

In Peru, the advent of telehealth predates the COVID-19 lockdown, with various initiatives initiated by non-governmental and educational entities, involving multiple projects in rural communities (4). Presently, the Ministry of Health (MINSA) holds exclusive responsibility for the implementation of telehealth services in the country, encompassing both synchronous and asynchronous modalities (5). Synchronous interactions involve real-time engagement between healthcare providers and patients, while the asynchronous approach entails deferred communication, with transmitted information subsequently reviewed by healthcare professionals (5). It is noteworthy, however, that the Ministry of Health faces certain limitations as the sole regulatory entity for telehealth in Peru.

Concerning the mental health status of the Peruvian population, empirical evidence indicates that a substantial proportion, ranging from 28% to 41.3%, experienced mental health challenges during the pandemic lockdown (6,7). Consequently, the imperative to attend to these concerns remotely has spurred the Peruvian health system to confront novel challenges, exemplified by the integration of e-screening methodologies designed to

discern and potentially address mental health issues. Notably, the global experience underscores that, despite the widespread activation of various e-screening initiatives in routine clinical practice throughout the COVID-19 era, comprehensive studies evaluating their appropriateness, efficiency, and efficacy remain notably scarce (8). In the Peruvian context, telehealth assumes a pivotal role in bridging this existing gap.

Peruvian regulatory framework defines telehealth as the provision of health services delivered through communication and information technologies, thereby organizing and advocating for the widespread use of this service (9). Notably, during the pandemic, telehealth services, in general, increased their supply and demand by patients, so much so that the Ministry of Health reported a 6000% increase in the daily use of telehealth services provided by Infosalud in the first 6 months of the pandemic (10), and further noted that approximately 70% of telehealthcare corresponded to mental health issues (11).

However, these telehealth services focus on the treatment of patients, leaving a gap concerning early identification through screening and initial management of patients so that the disease does not develop. Our research aims to identify the barriers and limitations for the future development of a tele-mental health service for screening, evaluation and timely referral in mental health based on the experiences of workers who are susceptible to these issues and also from the health workers who provide the service and the decision-makers at the top. These vulnerable occupational groups (VOGs) have been particularly affected, as is the case with healthcare workers, police personnel, and educators who have been exposed to a substantial increase in workload, direct exposure to COVID-19, shortages of personal protective equipment, abuse, and discrimination, among other circumstances (12–20).

MATERIAL AND METHODS

Study design and methods

This qualitative study employed a phenomenological approach, immersing itself in data to interpret generated meanings that reflect participants' subjective realities (21). The verbatim transcription of interviews played a pivotal role in ensuring effective interpretation (21). Utilizing Interpretative Phenomenological Analysis (IPA) methods, the research conducted semi-structured interviews to explore the experiences of service users, health workers, and decision-makers. Crafted with specific questions, these interviews aimed to facilitate a dialogue conducive to uncovering new aspects aligned with the research objectives (22). Spanning durations of 10 to 35 minutes, the interviews were conducted to comprehend perceptions, needs, barriers, and facilitators related to the implementation of mental telehealth services. The analysis was carried out using Atlas.ti 8, encompassed coding based on textual quotes, information structuring, and the consolidation of the telehealth platform evaluation across the diverse study participants, occurring after the familiarization with transcribed interviews.

Data collection methods

Policemen, Health professionals and Teachers

We conducted a total of 15 interviews with various user groups, including health professionals, teachers, and police officers. The inclusion criteria comprised individuals who scored high on depressive or anxious symptoms, determined by the PHQ-2 and GAD-2 tools. This assessment was integrated into the occupational activities conducted by CENSOPAS-INS (*Centro Nacional de Salud Ocupacional y Protección del Ambiente para la Salud- Instituto Nacional de Salud*, in Spanish).

Police officers were invited by DIRSAPOL (*Direction de la sanidad policia*l, in Spanish; the police health directorate) and were selected using intentional non-probabilistic sampling. Meanwhile, teachers were invited through UGEL 3 (Unidad de Gestion Educativa local, in Spanish; a local educational entity in Perú).

In addition, we conducted five interviews with healthcare professionals engaged in teleconsultations, primarily psychologists or physicians providing mental health care within Community Mental Health Centers in Lima or other MINSA health facilities in Lima. Specifically, these professionals were sourced from the Community Mental Health Centers of the Ministry of Health (MINSA), including San Gabriel Alto, La Perla, and Carabayllo.

Decision-makers

We have conducted three interviews with decision-makers, defined as public officials whose responsibilities are associated with administrative, managerial, or decision-making aspects of remote mental health care. These individuals may represent institutions such as the Directorate of Mental Health (DSAME, in Spanish) within MINSA, the General Directorate of Telehealth, Emergencies, and Referrals (DIGTEL, in Spanish) of MINSA, and EsSalud's National Telehealth Center (CENATE, in Spanish).

Ethical statement and Informed consent

Informed consent was obtained before conducting each of the 23 interviews. Participants received an overview of the study, along with the informed consent document. Upon orally expressing their agreement to participate, audiovisual or voice recording, as applicable, was initiated. Additionally, the explanation and informational sheet explicitly communicated participants' autonomy to withdraw from the study at any point if they wished to do so. The study protocol was approved by the Research and Ethic Institutional Committee from the INS (Instituto Nacional de Salud- Peru) under Resolution: RD N°206- 2023-OGITT/INS, code OC-048-2022. The information collection started from June 23 th to September 9th, 2023.

Data Analysis

In the analysis of our findings, we have organized them into themes and subthemes derived from the data collected through semi-structured interviews. Adopting a phenomenological approach, the codes have been tailored to align with the nuances apparent in our dataset. This dynamic process has enhanced our coding framework, directing our focus towards the four key themes associated with the mental health telecare and

screening service. The primary themes and subthemes that emerged from this process are detailed in **Table 1.**

Table 1.

Topics	Subtopics
1. Telehealth does not satisfy users	1.1. Very cold and distant services<br>1.2. Lack of medical record
2. Problems with the schedules and timetable of attention.	2.1. Non-fixed schedules<br>2.2. Unfulfilled schedules
3. Training of health care professionals in tele healthcare	3.1. The rapport between patients and healthcare professionals<br>3.2. Lack of connectivity
4. Need for integration into the health system	4.1. Lack of infrastructure<br>4.2. Coordination with other ministries

RESULTS

Telehealth does not satisfy users

The dissatisfaction expressed by users with telehealth services highlights a significant aspect of the limitations associated with remote medical care. One user noted their discomfort with the absence of the physical presence of a doctor during teleconsultations, emphasizing the impersonal nature of the interaction. The sentiment expressed resonates with the challenges of providing holistic care through telehealth, where the inability to observe non-verbal cues and physical conditions might impact the quality of mental health assessments.

"I found it unsatisfactory that, as I mentioned, the doctor isn't physically present to assess one's mental state. They simply provide a prescription, listen to you, and based on that, recommend a list of medications for you to pick up. It feels like a distant form of care."

-High-school teacher-

Furthermore, the desire for a comprehensive medical record points to a critical need for better integration of digital platforms in healthcare. The absence of a centralized and accessible medical history for users creates a gap in continuity of care. This limitation becomes apparent in scenarios where ongoing follow-up is required, and the lack of a consolidated medical record hinders the effectiveness of subsequent interactions.

"Subsequently, they provided ongoing follow-up. A doctor called me the next day, even in the evening, and continued to do so until the third and fourth calls. However, when I reached out again, what I hoped for was a comprehensive medical record in place. This way, when a person calls again, they already have access to their history, and the healthcare providers are aware of the prescribed treatments."

-Elementary school Teacher-

These user experiences underscore the importance of addressing not only the technical aspects of telehealth but also the broader aspects of user satisfaction and continuity of care. Integrating solutions that provide a more personalized and comprehensive healthcare experience could enhance the overall effectiveness and user satisfaction with telehealth services.

Problems with the schedules and timetable of attention

The issues raised by users regarding the scheduling and timing of telehealth appointments shed light on the challenges associated with aligning virtual consultations with users' daily lives. One user highlighted the inconvenience of receiving calls during specific hours, such as eight to nine in the evening. This timing was noted as less than ideal, especially when users were engrossed in household activities, such as cooking. The

unexpected nature of the calls created disruptions and surprises, leading to a less-than-optimal user experience.

> "But one hassle has been, for instance, getting a call around eight, eight-thirty, nine. It's not the best time, you know? Sometimes, you're already knee-deep in household chores, maybe cooking in the kitchen. It's a bit of a hassle because they ring you up, the phone goes off, you rush, caught up doing something urgent in the kitchen. And there it is, surprise, it's the doctor on the line."
>
> -High-school teacher-

The second quote further emphasizes the inconsistency in adhering to scheduled appointments. Users expressed frustration when promised calls did not occur at the designated time or date, indicating a potential flaw in the reliability of telehealth scheduling systems. This lack of adherence to agreed-upon schedules not only disrupts users' routines but also raises concerns about the overall efficiency and effectiveness of telehealth services.

> "Of course, that's more or less what I was mentioning almost at the beginning, right? The issue where the doctor tells you they'll call you on a certain date at a certain time, but it doesn't happen at that time. Maybe it doesn't even happen on that date. So, it's like you expected the call, but then they don't call. It's a limitation I've noticed, a problem I've been able to identify."
>
> -Healthcare worker-

These user experiences underscore the importance of flexible scheduling options in telehealth to accommodate users' diverse daily routines. Additionally, there is a need for improved communication and adherence to scheduled appointments to enhance user satisfaction and trust in telehealth services. Addressing these challenges is crucial for optimizing the usability and acceptance of telehealth platforms.

Training of healthcare professionals in tele healthcare

The discourse surrounding the training of healthcare professionals in telehealth care brings attention to the varied experiences and viewpoints of practitioners adjusting to remote service provision. The first quote underscores the distinction between providing psychological support through telehealth and more formal evaluation or therapy. Despite the acknowledged challenge of building rapport over the phone, the practitioner emphasizes the practical advantages of telehealth, particularly in reaching a broader audience, including those in remote locations with limited economic means.

> "It's important to note that what we were providing was psychological support, not formal evaluation or therapy. Naturally, establishing rapport with individuals over the phone can be a bit more challenging. However, from a practical standpoint, I believe it's a more effective approach because it allows us to extend our services to a broader audience. We can engage with people who live at a considerable distance, making it economically unfeasible for them to come in person. So, I see that as the primary advantage."
>
> -Healthcare worker-

Conversely, the second quote provides a firsthand account of the challenges encountered during teleconsultations. The mention of connectivity issues, interference, dropouts, and freezing problems reveals the technological barriers faced in the process. This personal experience sheds light on the complexities healthcare professionals confront when implementing telehealth, particularly in areas with limited infrastructure.

> "I started in December 2021, and the experience wasn't very helpful, especially during teleconsultations or virtual sessions, as they call it. In the district where the health center is located, people lack easy access to networks and the internet. Most only have a cellphone connection, and when we managed to connect, there were often significant interference, dropouts, and freezing issues. We usually ended up resorting to a simple phone call. Many times, we started with the

intention of using video to see the patient, but it typically turned into a phone call due to these connectivity challenges."

-Healthcare worker-

Collectively, these quotes highlight the necessity for comprehensive training programs for healthcare professionals engaging in telehealth. Such training should not solely concentrate on the technical aspects of using telecommunication tools but also address the distinctive dynamics of remote patient interactions. Additionally, they underscore the significance of recognizing the limitations and hurdles associated with the implementation of telehealth services, with a focus on mitigating technological barriers to ensure effective and equitable healthcare delivery.

Need for integration into the health system

In discussing the need for integration of telehealth into the health system, two key quotes provide insights into the challenges and requirements for a successful implementation.

> "Not tied directly to regulations but rather to the resources needed for such interventions. It's crucial to have the know-how, and at that time, the ministry was gaining expertise. The telehealth department was still emerging, a limitation not just in mental health but overall. Implementation resources within services, like computerization, an electronic health record, and a reliable network, were lacking in the country. Users needed data, including mobile phones, for access. Especially for MINSA, serving 20 million people insured under the SIS, primarily due to limited economic resources. According to INEI reports, around 50-60% of people in some regions lacked a cellphone or computer in 2020-2021. You could have everything implemented, but if users lack network coverage and don't have the necessary equipment or data for connection, it becomes an important but insufficient tool for institutions in the Ministry of Health catering to a population with these economic characteristics."

-Decision-maker-

Initiating with the first quote, it underscores the critical aspect of resources necessary for telehealth interventions. It highlights the evolving nature of the telehealth department within the ministry, emphasizing the need for expertise. This is not limited to mental health but extends to the broader healthcare landscape. The lack of implementation resources, including computerization, electronic health records, and a reliable network, is identified as a significant limitation. The quote emphasizes the importance of users having access to data and devices, particularly in economically constrained regions. The challenges faced by the Ministry of Health, serving a large population with limited resources, are highlighted through statistics indicating a lack of mobile phones or computers among a significant percentage of the population.

> "In 2020, updates to the National Mental Health Plan were made in the context of COVID, for instance. We reviewed these to align with specific regulations for our strategy implementation. Operational definitions were derived from MINSA documents. Additionally, there were other regulations, such as those related to database use. Managing databases with confidential information from users necessitates procedures, like registration overseen by the Ministry of Justice. Participation, determining focal points, and ensuring database protection are essential aspects beyond merely developing the service..."
>
> -Decision-maker-

Conversely, the following quote delves into the regulatory and procedural aspects necessary for the integration of telehealth. It references updates in the National Mental Health Plan in response to the COVID context, signifying the dynamic nature of regulations. The operational definitions derived from MINSA documents illustrate the reliance on established guidelines. The mention of regulations related to database use highlights the importance of managing sensitive information. The necessity for procedures overseen by the Ministry of Justice, including registration and database protection, further emphasizes the regulatory framework and ethical considerations involved in telehealth implementation.

Collectively, these quotes underscore the multifaceted challenges in integrating telehealth into the health system. They emphasize the need for a comprehensive approach that addresses not only technical and resource-related challenges but also regulatory and ethical considerations to ensure a successful and ethical implementation within the broader healthcare ecosystem.

DISCUSSION

Holistic and User-Centered Paradigm for Telehealth Implementation:

The study's outcomes underscore the imperative shift toward a holistic and user-centered approach to telehealth implementation. This necessitates a comprehensive reevaluation extending beyond the conventional focus on technological aspects to encompass the intricate interplay of human and systemic elements in the remote delivery of healthcare. The integration of such an approach has the potential to address multifaceted challenges, paving the way for the realization of telehealth's transformative force in ensuring accessible, equitable, and high-quality healthcare services (23).

Multifaceted Challenges and Transformative Potential

The identified multifaceted challenges inherent in telehealth implementation serve as pivotal points for stakeholders to intervene. By strategically addressing these challenges, stakeholders can unlock the full potential of telehealth as a transformative force. Notably, experiences from multifaceted telehealth interventions indicate that this holistic approach is not only effective but can also serve as a viable solution for crafting intricate telehealth interventions, particularly in resource-constrained settings where time and resources are limited.

Policy Emphasis and Future Directions

The current landscape of telehealth policy has predominantly focused on expanding access to specialized services and allowable originating sites (24). Moving forward, a crucial paradigm shift is advocated, urging policymakers to enhance telehealth equity through the implementation of system-level changes. These changes are envisioned to eliminate structural barriers that historically impede access for marginalized and low-income populations. By addressing these barriers, policymakers can contribute significantly to fostering a more inclusive and equitable telehealth ecosystem.

CONCLUSION

In conclusion, the study's findings contribute substantively to the ongoing discourse on telehealth implementation. The call for a holistic, user-centered approach aligns with the evolving needs of healthcare delivery in a remote context. The insights provided underscore the importance of not only advancing technological capabilities but also understanding and addressing the intricate socio-cultural and systemic dynamics to fully unlock the potential of telehealth in improving healthcare accessibility and quality.

Funding: This work was funded by the *Agencia Española de Cooperación* Internacional para el Desarrollo (AECID, in Spanish) by *Programa Nacional de Investigación Científica y Estudios Avanzados- CONCYTEC-Perú,* under contract: N° 076-2021-PROCIENCIA.

Conflict of interest: Authors declare do not have competing of interest.

Author´s contribution: All the authors contributed equally to this work

REFERENCES

1. Poonsuph R. The design blueprint for a large-scale telehealth platform. Int J Telemed Appl. 2022;2022.
2. Doraiswamy S, Abraham A, Mamtani R, Cheema S. Use of Telehealth During the COVID-19 Pandemic: Scoping Review. J Med Internet Res [Internet]. 2020;22(12):e24087. Available from: https://www.jmir.org/2020/12/e24087
3. Mahmoud K, Jaramillo C, Barteit S. Telemedicine in low-and middle-income countries during the COVID-19 pandemic: a scoping review. Front Public Heal. 2022;10:914423.
4. Camacho-Leon G, Faytong-Haro M, Carrera K, Molero M, Melean F, Reyes Y, et al. A Narrative Review of Telemedicine in Latin America during the COVID-19 Pandemic. In: Healthcare. MDPI; 2022. p. 1361.
5. Ministerio de Salud. Resolución Ministerial N.° 117-2020-MINSA [Internet]. [cited 2020 Mar 26]. Available from: https://www.gob.pe/institucion/minsa/normas-legales/462698-116-2020-minsa
6. Pérez-Cano HJ, Moreno-Murguía MB, Morales-López O, Crow-Buchanan O, English JA, Lozano-Alcázar J, et al. Anxiety, depression, and stress in response to the coronavirus disease-19 pandemic. Cir Cir. 2020;88(5):562–8.
7. Ministerio de Salud del Perú. Documento Técnico: Plan de Salud Mental (En el contexto Covid-19-Perú, 2020-2021) (Internet) [Internet]. Lima - Perú: MINSA SA. 2020. p. p.29. Available from: https://cdn.www.gob.pe/uploads/document/file/811138/Plan_de_Salud_mental_.PDF
8. Grussu P, Quatraro RM, Alderdice F, Jorizzo GJ. Routine E-Screening for Perinatal Mental Health During the COVID-19 Emergency: Beyond the Instrument Cut-Off Points. Matern Child Health J. 2022;26(12):2357–61.
9. Ministerio de Salud del Perú. Decreto Supremo N° 005-2021-SA que aprueba el Reglamento de la

Ley Nº 30421, Ley Marco de Telesalud, y del Decreto Legislativo Nº 1490, Decreto Legislativo que fortalece los alcances de la Telesalud. D Of El Peru [Internet]. 2021;23–32. Available from: https://busquedas.elperuano.pe/download/url/decreto-supremo-que-aprueba-el-reglamento-de-la-ley-n-30421-decreto-supremo-n-005-2021-sa-1922320-2

10. Ministerio de salud del Perú. Más de 84 mil orientaciones en salud mental atendió el Minsa a través de la Línea 113 Salud en 2020 [Internet]. 2020 [cited 2023 Nov 20]. Available from: https://www.gob.pe/institucion/minsa/noticias/300531-mas-de-84-mil-orientaciones-en-s

11. Kang L, Ma S, Chen M, Yang J, Wang Y, Li R, et al. Impact on mental health and perceptions of psychological care among medical and nursing staff in Wuhan during the 2019 novel coronavirus disease outbreak: A cross-sectional study. Brain Behav Immun. 2020;87:11–7.

12. Zhang W rui, Wang K, Yin L, Zhao W feng, Xue Q, Peng M, et al. Mental health and psychosocial problems of medical health workers during the COVID-19 epidemic in China. Psychother Psychosom. 2020;89(4):242–50.

13. Silva N, Elizabeth-Cabral BR, Leão LL, Pena GDG, Pinho L, Magalhães TA. Working conditions, lifestyle and mental health of Brazilian public-school teachers during the COVID-19 pandemic. Psychiatriki. 2021;32(4):282–9.

14. Grover S, Sahoo S, Dua D, Mehra A, Nehra R. Psychological impact of COVID-19 duties during lockdown on police personnel and their perception about the behavior of the people: an exploratory study from India. Int J Ment Health Addict. 2020;1–12.

15. Degregori CI. Desigualdades persistentes y construcciónde un país pluricultural. Present hecha en el Taller Intercult Organ por CEPES [Internet]. 2000;1–9. Available from: http://www.pucp.edu.pe/ridei/pdfs/Carlos Iván Degregori.pdf

17. Caycho-Rodríguez T, Carbajal-León C, Vilca LW, Heredia-Mongrut J, Gallegos M. COVID-19 y salud mental en policías peruanos: resultados preliminares. Acta Médica Peru. 2020;37(3):396–8.

18. Villarreal-Zegarra D, Copez-Lonzoy A, Vilela-Estrada AL, Huarcaya-Victoria J. Depression, post-traumatic stress, anxiety, and fear of COVID-19 in the general population and health-care workers: prevalence, relationship, and explicative model in Peru. BMC Psychiatry. 2021;21:1–14.

19. Zamora Mendoza C, Velázquez Castro T, Del Solar Cárdenas Y, Gutiérrez Muñoz G, Sánchez Ponce C, Gonzales La Rosa Sánchez J, et al. Salud mental en docentes durante la pandemia: reflexiones en torno al género. Informe de Evidencia sobre Educación y Salud Mental, N° 3. 2021;

20. LaMontagne AD, Martin A, Page KM, Reavley NJ, Noblet AJ, Milner AJ, et al. Workplace mental health: developing an integrated intervention approach. BMC Psychiatry. 2014;14(1):1–11.

21. Duque H, Granados ETAD. Análisis fenomenológico interpretativo: Una guía metodológica para su uso en la investigación cualitativa en psicología. Pensando Psicol. 2019;15(25):1–24.

22. Smith J, Osborn M. Interpretative Phenomenological Analysis.[Documento en Línea] In J. Smith.(Ed.), Qualitative Psychology: A Practical Guide to Research Methods (pp. 53-80). 2008.

23. Davis SM, Jones A, Jaynes ME, Woodrum KN, Canaday M, Allen L, et al. Designing a multifaceted telehealth intervention for a rural population using a model for developing complex interventions in nursing. BMC Nurs. 2020;19(1):1–9.

24. Anaya YBM, Mota AB, Hernandez GD, Osorio A, Hayes-Bautista DE. Post-pandemic telehealth policy for primary care: an equity perspective. J Am Board Fam Med. 2022;35(3):588–92.

RESPONSE LETTER

Subject: Response to the observation on Dr. Jonh Maximiliano Astete Cornejo´s inclusion in the research team

Dear editor

I would like to thank for the feedback provided in your previous email. In reference to the inclusion of dr. Jonh Maximiliano Astete Cornejo in our research team as an investigator, I would like to clarify the following point:

As indicated in the submission, Dr John Astete Maximiliano Cornejo also referred to as "Jonh Astete Cornejo", serve as the principal investigator and is been listed as the first author in the PDF submission. For your convenience, I have attached a screenshot that corroborates this information.

Therefore, we kindly request a reconsideration of the suggested change, taking into account the clarification provided. Your attention to this matter is greatly appreciated.

Thanks for your information and support

Kind regards

Cruz-Ausejo Liliana

[illegible] and facilitators related to mental telehealth services through a semi-structured interview guide. The interviews were recorded and transcribed verbatim, furthermore, a thematic analysis was done to identify key themes.

Results

The research identified barriers and limitations to developing a tele-mental health screening service based on the experiences of workers, some of them were related to user dissatisfaction emphasizing the need for personalized solutions beyond technical aspects. Scheduling issues call for flexibility and improved communication. Healthcare professionals' varied experiences highlight the necessity for targeted training, while successful telehealth integration demands addressing resource limitations through a comprehensive approach.

Conclusion

The study advocates for a holistic, user-centred paradigm in telehealth implementation, addressing both technological aspects and human and systemic elements. Multifaceted challenges inherent in telehealth, particularly in Peru, emphasize the need for strategic interventions by stakeholders. The study calls for a policy shift towards enhancing telehealth equity through system-level changes and eliminating structural barriers for marginalised populations

**Order of Authors:**	Jonh Astete Cornejo, Dr.
	Liliana Cruz- Ausejo, MSc
	Jimmy Cainamarks Alejandro, BSc

Powered by Editorial Manager® and ProduXion Manager® from Aries Systems Corporation

	Miguel Burgos Flores, Msc
	Juan Ambrosio Melgarejo, BPsyc
	Jaime Rosales Rimache, Dr.
	Sebastian Daniel Garcia Cruz, BSc
