# Supplementary material for: Barriers and limitations to the development of a telemental health service for workers in Peru- A user-centered approach
Source: PLoS One. 2025 Apr 9;20(4):e0321401. doi: 10.1371/journal.pone.0321401 (PMC11981184; doi:10.1371/journal.pone.0321401)
Supplement: Attachment — Submitted filename: Response to Reviewers v2 (1).pdf [file pone.0321401.s008.pdf]

Dear Editor,

Thank you for the opportunity to review the manuscript entitled “Barriers and limitations to the development of a tele-mental health service for workers- A user-centered approach”.

In the following paragraphs, we proceed to comment on the changes made according to each observation made by the reviewers and Academic Editor.

Academic Editor Comments:

1. Please ensure that your manuscript meets PLOS ONE's style requirements, including those for file naming. The PLOS

ONE style templates can be found at

https://journals.plos.org/plosone/s/file?id=wjVg/PLOSOne_formatting_sample_main_body.pdf and

https://journals.plos.org/plosone/s/file?id=ba62/PLOSOne_formatting_sample_title_authors_affiliations.pdf

Answer: All the changes suggested by the reviewers have been made.

2. Thank you for stating the following financial disclosure: "Funding: This work was funded by the Agencia Española de

Cooperación Internacional para el Desarrollo (AECID, in Spanish) by Programa Nacional de Investigación Científica y Estudios Avanzados- CONCYTEC- Perú, under contract: N° 076-2021-PROCIENCIA."

Please state what role the funders took in the study. If the funders had no role, please state: ""The funders had no role in study design, data collection and analysis, decision to publish, or preparation of the manuscript."" If this statement is not correct you must amend it as needed.

Please include this amended Role of Funder statement in your cover letter; we will change the online submission form on your behalf.

Answer: The statement of non-involvement by the funders in the study was included.

3. We note that you have indicated that there are restrictions to data sharing for this study. For studies involving human research participant data or other sensitive data, we encourage authors to share de-identified or anonymized data. However, when data cannot be publicly shared for ethical reasons, we allow authors to make their data sets available upon request. For information

on unacceptable data access restrictions, please see http://journals.plos.org/plosone/s/data-availability#loc-unacceptable-data-access-restrictions.

Before we proceed with your manuscript, please address the following prompts:

a) If there are ethical or legal restrictions on sharing a de-identified data set, please explain them in detail (e.g., data contain potentially identifying or sensitive patient information, data are owned by a third-party organization, etc.) and who has imposed them (e.g., a Research Ethics Committee or Institutional Review Board, etc.). Please also provide contact information for a data access committee, ethics committee, or other institutional body to which data requests may be sent.

b) If there are no restrictions, please upload the minimal anonymized data set necessary to replicate your study findings to a stable, public repository and provide us with the relevant URLs, DOIs, or accession numbers. Please see http://www.bmj.com/content/340/bmj.c181.long for guidelines on how to de-identify and prepare clinical data for publication. For a list of recommended repositories, please see https://journals.plos.org/plosone/s/recommended-repositories. You also have the option of uploading the data as Supporting Information files, but we would recommend depositing data directly to a data repository if possible.

Please update your Data Availability statement in the submission form accordingly.

Answer: There are no restrictions on sharing the anonymized data obtained in the study. Accordingly, the full transcripts of the interviews conducted with the participants, along with the Atlas.TI code file, are attached as a Supporting information file.

Reviewer #1:

The manuscript reflects a justifiable need for this study to be conducted and dissemination of data thereof. The methodology used is appropriate to elicit information from the participants. However, the discussion is scanty and does not clear overview of the findings against existing studies in other countries. As such, this manuscript requires minor revisions to give it more meaning at a standard that could be published by the journal.

Answer: The discussion topics in the manuscript were expanded according to subtopics. The discussion was expanded to consider different international contexts.

1. **Abstract:**

Well written. However, the authors need to provide a further breakdown of the number of participants, i.e.15, 5 and three, so that the characteristics of the participants are clear. The last sentence of the introduction in the abstract could read better if simplified and shortened. It loses meaning in its current state.

Answer: The observation has been acknowledged and modified in the abstract.

2. **Introduction**

Clear and to the point. Please check line 78 if the percentage is well represented.

Answer: The referred percentage was the one reported by the government in the official gazette.

3. **Data collection**

Although the authors mention that they used semi-structured interviews, the following is not clear:

- What was the opening question? What were some of the probing questions used?
- At what point did the interviews stop or at what point was the data sufficient or what guided the authors to stop data collection at 23 with different types of participants (15,5 and 3)?

Answer: The base questions of the semi-structured interview, as approved in the research protocol, are attached as a Supporting information file. These questions introduced the topic, and as can be observed in the interview transcripts, they allowed the interviewer to ask follow-up questions and explore certain criteria in greater depth. The interviews ceased upon reaching the sample approved by the ethics and research committee, as the

three groups contribute to the identification of development requirements for the same software.

4. **Discussion**

While the implications and future directions are outlined clearly, the discussion is scanty. There is a need to broaden it as, in its current form, it is narrow-focused.

Answer: The discussion was extended.

Reviewer #2:

This study address an important topic: Which barriers and facilitators exist for telemental health services for vulnerable workers in countries were telecommunication may be limited or lacking. They conducted 23 semi-structured interviews with receivers (police officers and teachers) and planners/providers (healthcare personnel and administrators).

General comments: I think language should receive more attention, preferrably using an authorized translator. I think the scientific basis is lacking, and there is no attempt to describe the theoretical basis on which the identification of barriers and facilitators were identified and sorted.

Answer: Thank you very much for your feedback. We strive to take all of your observations into account when making the necessary corrections. Grammar corrections were also made to improve comprehension.

Full Title: Barriers and limitations to the development of a tele-mental health service for workers - A user-centered approach I think the findings in this study apply to Peru, which is also stated in the abstract, and the title should refer to Peru. Suggested change: Barriers and limitations to the development of a telemental health service for workers in Peru - A user-centered approach

Answer: The observation has been resolved as "Barriers and limitations to the development of a telemental health service for workers in Peru - A user-centered approach."

Abstract

32 users and attendance The meaning of «attendance» here is unclear

33 mental telehealth Why not continue calling it telemental health

37 some of them were related to user dissatisfaction emphasizing the need for personalized solutions beyond technical aspects This could be specified more, what kind of dissatisfaction?

38 Scheduling issues call for flexibility and improved communication Meaning?

Answer: Text related to "attendance" was replacing by "providers of mental telehealth". The user's dissatisfaction centered on the lack of customizable solutions, like fast communication. The grammar was revised to clarify this.

Introduction

General comment: I think the authors should define their use of the telemental health service term, and they should describe the plethora of teleservices that may be available

51 is disrupting global healthcare services Is disrupt the preferred verb? Consider using terms like challenging or altering.

Answer: The definition and services related to telemental health were included, as well as the correction in the term "disrupting."

Disrupt definition: to prevent something, especially a system, process, or event, from continuing as usual or as expected

65 It is noteworthy, however, that the Ministry of Health faces certain limitations as the sole regulatory entity for telehealth in Peru Reference?

69 Consequently, the imperative to attend to these concerns remotely Meaning?

71 Notably, the global experience underscores that, despite the widespread activation of various e-screening initiatives in routine clinical practice throughout the COVID 19 era, comprehensive studies evaluating their appropriateness, efficiency, and efficacy remain notably scarce Avoid using notably twice in one sentence.

Answer: The recommendations were adopted, including the references and improving the writing according to the suggestions.

MATERIAL AND METHODS

91 Spanning durations of 10 to 35 minutes, the interviews were conducted to comprehend perceptions, needs, barriers, and 99 facilitators related to the implementation of mental telehealth services On what theoretical ground were barriers and facilitators identified? Who conducted the interviews – what professional background did they have? Did you recruit interviewees from urban or rural areas, or both?

Answer: The barriers and facilitators were identified using a holistic and user-centered approach. The interviews were conducted by the study's researchers, all of whom were trained in qualitative research methodologies. Additionally, all interviewees were recruited from urban areas. These aspects were incorporated into the writing of Material and Methods.

117 Decision makers How where these recruited?

Answer: The indicated precision was included in the writing.

125 Upon orally expressing their agreement to participate, audiovisual or voice recording, as applicable, was initiated. A Did you not receive written consent?

Answer:The prior application of consent is described in the section "Ethical Statement and Informed Consent."

133 . Adopting a phenomenological approach, the codes have been tailored to align with the nuances apparent in our dataset Meaning?

Answer: The barriers and facilitators were identified using a holistic and user-centered approach.

134 This dynamic process has enhanced our coding framework, directing our focus towards the four key themes associated with the mental health telecare and screening service. The primary themes and subthemes that emerged from this process are detailed in Table 1 Wouldn't this be more appropriately put in results section, including table?

Answer: Table 1 was moved to the Results section.

Results

General comment: From 23 interviews 8 quotes are presented, for instance the reference to «healthcare worker» does not clarify whether it is quotes from the same healthcare worker or various (that is: it is impossible for the reader to assess it).

The same problem arises for «Decision-maker». One could extend the characterization of each responder, by indicating if rural or urban, if not in conflict with anonymity.

Answer: Modifications were made to the presentation of the interviewees by assigning them alfanumerical identifiers (D#) to distinguish between them.

General comment: The healthcare workers represent a completely different position as compared to the other interviewees, since these represent the service and not the receiver of services. Thus, I find it problematic that their experiences are included in the same table. See suggestion above.

137 Table 1 1.2. Lack of medical record Where this issue addressed? If not, consider omitting it from the table

137 Table 1 Consider revising the table, indicating that item 1 and 2 represent «Receiver of the services» and 3 and 4 represent «Provider/planner of the services»

Answer: The changes were made according to the suggestions.

Discussion

General comments: In discussion there is a chance to discuss strengths and limitations of the study. They should also compare their own findings with existing literature, and they should make an attempt to guide clinicians and decision makers, base don theri findings. In addition, the discussion addresses to a very limited degree the challenges that were identified in the results section. Rather than describing approaches to improve telehealthcare in Peru in very general terms, they should use this section to develop more specific thoughts on the significance of their findings

270 The study's outcomes underscore the imperative shift toward a holistic and user-centered approach to 271 telehealth implementation How?

Answer: More details were added based on feedback.

The authors consider that we have provided a response to all the observations made to the manuscript; Therefore, we send this document for your review.

Kind regards
