# Supplementary material for: Barriers and limitations to the development of a telemental health service for workers in Peru- A user-centered approach
Source: PLoS One. 2025 Apr 9;20(4):e0321401. doi: 10.1371/journal.pone.0321401 (PMC11981184; doi:10.1371/journal.pone.0321401)
Supplement: Attachment — Submitted filename: Recommendations from reviewer PLOS ONE.doc [file pone.0321401.s007.doc]

Dear Editor,

Thank you for the opportunity to review the manuscript entitled “Barriers and limitations to the development of a tele-mental health service for workers- A user-centered approach”.

Kindly see the comments below:

The manuscript reflects a justifiable need for this study to be conducted and dissemination of data thereof. The methodology used is appropriate to elicit information from the participants. However, the discussion is scanty and does not clear overview of the findings against existing studies in other countries. As such, this manuscript requires minor revisions to give it more meaning at a standard that could be published by the journal.

1. **Abstract:**

Well written. However, the authors need to provide a further breakdown of the number of participants, i.e.15, 5 and three, so that the characteristics of the participants are clear. The last sentence of the introduction in the abstract could read better if simplified and shortened. It loses meaning in its current state.

2. **Introduction**

Clear and to the point. Please check line 78 if the percentage is well represented.

3. **Data collection**

Although the authors mention that they used semi-structured interviews, the following is not clear:

- What was the opening question? What were some of the probing questions used?
- At what point did the interviews stop or at what point was the data sufficient or what guided the authors to stop data collection at 23 with different types of participants (15,5 and 3)?

4. **Discussion**

While the implications and future directions are outlined clearly, the discussion is scanty. There is a need to broaden it as, in its current form, it is narrow-focused.

Regards,

Reviewer

05/02/2024
