# Supplementary material for: Barriers and limitations to the development of a telemental health service for workers in Peru- A user-centered approach
Source: PLoS One. 2025 Apr 9;20(4):e0321401. doi: 10.1371/journal.pone.0321401 (PMC11981184; doi:10.1371/journal.pone.0321401)
Supplement: Attachment — Submitted filename: Response to reviewers.docx [file pone.0321401.s009.docx]

Dear Editor,

Thank you for the opportunity to review the manuscript entitled “Barriers and limitations to the development of a tele-mental health service for workers- A user-centered approach”. The following section addresses the resolution of the received comments:

1. **Abstract:**

Well written. However, the authors need to provide a further breakdown of the number of participants, i.e.15, 5 and three, so that the characteristics of the participants are clear. The last sentence of the introduction in the abstract could read better if simplified and shortened. It loses meaning in its current state.

**Response:** The observations had already been resolved in the last version sent

2. **Introduction**

Clear and to the point. Please check line 78 if the percentage is well represented.

**Response:** Although this is the percentage figure provided by an official source, the wording has been modified according to the observation.

3. **Data collection**

Although the authors mention that they used semi-structured interviews, the following is not clear:

- What was the opening question? What were some of the probing questions used?
- At what point did the interviews stop or at what point was the data sufficient or what guided the authors to stop data collection at 23 with different types of participants (15,5 and 3)?

**Response:** The interview began with introductory questions designed to explore general perceptions of telehealth in mental health. Subsequently, probing questions were used to delve deeper into participants' experiences and their perspectives on the care process. Additionally, the Supporting Information section of the manuscript includes S1 File: Semi-Structured Interview Format, which contains the interview guide used in this study.

The interviews were conducted until data saturation was reached, meaning no new themes or additional relevant information emerged. The sample included three decision-makers, who represented the entirety of the actors responsible for the regulation and implementation of telehealth at that time, a nascent service in the Peruvian context. Although three distinct groups participated (users, providers, and decision-makers), their contributions were integrated into the formulation of a single telemental health service, confirming that data saturation had been achieved.

4. **Discussion**

While the implications and future directions are outlined clearly, the discussion is scanty. There is a need to broaden it as, in its current form, it is narrow-focused.

**Response:** More discussion elements were incorporated into the manuscript.

Additional Editor Comments (if provided):

Dear Authors,

PLOS ONE considers qualitative and mixed-methods studies for publication. We recommend that authors use the COREQ checklist, or other relevant checklists listed by the Equator Network, such as the SRQR, to ensure complete reporting (http://journals.plos.org/plosone/s/submission-guidelines#loc-qualitative-research). In general, we would expect qualitative studies to include the following: 1) defined objectives or research questions; 2) description of the sampling strategy, including rationale for the recruitment method, participant inclusion/exclusion criteria and the number of participants recruited; 3) detailed reporting of the data collection procedures; 4) data analysis procedures described in sufficient detail to enable replication; 5) a discussion of potential sources of bias; and 6) a discussion of limitations.

**Response:** We applied the COREQ guidelines for reporting qualitative research in this manuscript. A copy of the checklist is attached with the submission. Furthermore, we believe that the editorial requirements have been met.

We have addressed all submitted observations and believe the manuscript is now ready for further review and to proceed with the publication process.

Best regards,

The Authors.
